# Comparative plastid genomics of *Gastrochilus* (Orchidaceae) with insights into molecular markers and phylogeny

**DOI:** 10.1186/s12864-026-12698-8

**Published:** 2026-06-26

**Authors:** Qi Wu, Ruimin Yu, Xiaoxiang Deng, Yibo Luo, Xiujin Qi, Shubin Dong, Jin Cheng

**Affiliations:** 1 National Engineering Research Center of Tree Breeding and Ecological Restoration, State Key Laboratory of Efficient Production of Forest Resources, College of Biological Sciences and Technology, Beijing Forestry University, Beijing, 100083 China; 2 Forestry Comprehensive Service Center of Xinning County, Shaoyang, 422700 China; 3 State Key Laboratory of Plant Diversity and Specialty Crops, Institute of Botany, Chinese Academy of Sciences, Beijing, 100093 China

**Keywords:** Plastid genomes, Gastrochilus, Comparative analysis, Molecular markers, Phylogeny

## Abstract

**Background:**

*Gastrochilus* (Orchidaceae) is a genus of epiphytic orchids valued for horticultural and medicinal purposes. However, the structural and evolutionary features of its plastid genomes have not been systematically examined. Here, we characterize the complete plastid genomes of 23 *Gastrochilus* species, elucidating structural features and key variations to provide new insights into their evolutionary significance.

**Results:**

The plastid genomes showed a typical quadripartite structure, ranging from 146,183 to 148,666 bp in length. Each genome encodes 120 genes, comprising 74 protein-coding genes, 38 tRNA genes, and 8 rRNA genes. A prominent feature was the recurrent pseudogenization or loss of *ndh* genes. We identified 1,200 simple sequence repeats and 905 dispersed repeats. Codon usage was highly conserved, with a consistent preference for A/U-ending codons and an overall weak codon usage bias. Expansion and contraction of the inverted repeat regions notably affected the position of *ycf1*. Nucleotide diversity analysis revealed ten hypervariable regions and eight genes as potential molecular markers. Evidence of positive selection was detected in *rps16* and *ycf2*. Phylogenetic analysis divided the 23 *Gastrochilus* species into four well-supported clades.

**Conclusions:**

The plastid genomes of *Gastrochilus* display highly conserved architecture. The recurrent loss or pseudogenization of *ndh* genes, along with positive selection in *ycf2*, may reflect adaptations to low-light forest environments. The identified repeat sequences and hypervariable regions provide valuable molecular markers for phylogenetic studies and species identification. This phylogenetic framework, based on complete plastid genomes, offers the most comprehensive resolution for *Gastrochilus* to date. However, the adaptive significance of these genomic features requires further experimental validation to confirm their ecological relevance.

**Supplementary Information:**

The online version contains supplementary material available at https://doi.org/10.1186/s12864-026-12698-8.

## Background

*Gastrochilus* D. Don (Orchidaceae, Aeridinae) comprises approximately 81 species distributed across tropical and subtropical Asia. They are mostly are epiphytes growing on forest tree trunks or rocks [1–4]. Their diverse floral traits and the occurrence of bioactive alkaloids confer both horticultural and medicinal importance [5–8].

Recent systematic studies of *Gastrochilus* have advanced its conservation and evolutionary understanding. Molecular data suggest that the most recent common ancestor of *Gastrochilus* originated in the late Miocene in subtropical East Asia [9]. Integrative morphological and molecular analyses support the division of the genus into six Sects [10, 11]. Nonetheless, existing phylogenies often show topological conflicts and weak nodal support. Phylogenies based on nuclear internal transcribed spacers and plastid markers become inconsistent when additional plastid markers are incorporated [9, 10, 12]. Furthermore, these results contradict topologies derived from complete plastid genomes [4, 11]. Such discrepancies may stem from historical hybridisation or introgression within Aeridinae, leading to distinct evolutionary trajectories for nuclear and plastid genomes [13]. Moreover, plastome-based phylogenies are sensitive to sampling size and composition. With fewer than 20% of species represented in current datasets, inadequate sampling in certain clades likely contributes to topological instability [4, 11].

Compared to nuclear genomes, plastid genomes are characterised by uniparental inheritance, lack of recombination, a low nucleotide substitution rate and structural conservation [14, 15]. Complete plastid genome sequences improve the resolution and accuracy of phylogenetic inference within defined taxonomic groups [16–18]. This utility is also well-illustrated by research on *Gastrochilus* and related genera [4, 19]. Furthermore, attributes of plastid genomes, such as GC composition, structural rearrangements, and genic variation, are increasingly analyzed in conjunction with nuclear genomic data and ecological niche data. This integrated approach sheds light on mechanisms of speciation and adaptive evolution [19–24]. Plastid simple sequence repeats (SSRs) serve as valuable markers for phylogenetic studies, population structure, and genetic diversity assessment [25–27]. Similarly, dispersed repeats (DRs) influence both species evolution and plastome stability [28, 29]. Collectively, plastid genome data offer critical information on maternal lineage and form a solid basis for reconstructing evolutionary histories. In this study, we assembled and annotated the plastid genome of *G. rantabunensis*, and annotated four previously published *Gastrochilus* plastid genomes for which published annotations were lacking. We conducted comparative analyses of plastid genome characteristics and phylogenetic relationships using all available *Gastrochilus* plastid genomes. The objectives were: (1) to characterize the structural features and key variations of *Gastrochilus* plastid genomes and examine their evolutionary patterns; (2) to identify species-specific repeat sequences as potential plastid DNA barcodes, and (3) to reconstruct a robust *Gastrochilus* phylogeny using complete plastid genomes and assess the causes of topological conflicts in traditional markers.

## Methods and materials

### Plant materials and plastid genome sequencing

Fresh leaves of *G. rantabunensis* were collected from a living plant in Xinning County, Shaoyang, Hunan Province, China. The voucher specimen was deposited in the Herbarium of the Plant Biology Department, Beijing Forestry University (BJFU, voucher number: GR20210710). The plant material of *G. rantabunensis* was formally identified by Dr. Yibo Luo of the Institute of Botany, Chinese Academy of Sciences, based on morphological comparing with the pictures in two books [30, 31]. This species is not listed as a nationally protected plant in China, so no collection permits were necessary. Genomic DNA was extracted from these leaves using the Tiangen DNA Kit (TIANGEN, China). Subsequently, sequencing libraries were constructed and subjected to 150 bp paired-end sequencing on the Illumina HiSeq 2500 system (NEB, USA).

### Plastid genome assembly and annotation

The complete plastid genome of *G. rantabunensis* was assembled using GetOrganelle v1.7.5 [32]. The remaining 22 plastid genomes of *Gastrochilus* were obtained from the NCBI database (Table S1). All plastid genomes were annotated using PGA [33], with previously published *Gastrochilus* plastid genomes used as reference sequences (Table S1). All annotations were subsequently inspected and manually corrected in Geneious Prime v2021.1.1 [34]. Given that widespread pseudogenization or loss of *ndh* genes was observed in *Gastrochilus* plastid genomes, annotations of *ndh* genes were further examined using *Musa acuminata* genome (HF677508), a well-studied monocot species with a complete and well-annotated plastid genome, which has been used as a reference in previous plastome studies [4, 22, 35]. Information on *ndh* gene loss and pseudogenization was summarized based on PGA warning logs and visual inspection in Geneious Prime. Finally, plastid genome maps were generated using CPGView (http://www.1kmpg.cn/cpgview/) [36].

### Identification of sequence repeats

SSRs in the plastid genomes of the 23 *Gastrochilus* species were analysed via MISA (https://webblast.ipk-gatersleben.de/misa/index.php? action=1), with minimum number of repeats set to 10, 5, 4, 3, 3 and 3 for mononucleotide, dinucleotide, trinucleotide, tetranucleotide, pentanucleotide and hexanucleotide repeats, respectively [37, 38]. DRs were analyzed using REPuter (https://bibiserv.cebitec.uni-bielefeld.de/reputer/), where forward, reverse, complement and palindromic repeats were defined by a minimum size of 30 bp and a Hamming distance of three [39, 40].

### Codon usage analysis

Both relative synonymous codon usage (RSCU) and the effective number of codons (ENC) were calculated using CodonW v1.4.2 [41]. RSCU analysis included all plastid protein-coding genes, whereas ENC calculation was limited to genes ≥ 300 bp to reduce bias from short sequences. The resulting RSCU values were visualized using the online platform JSHYCloud (http://cloud.genepioneer.com:9929/#/tool/alltool/detail/335). ENC values range from 20 (strong bias) to 61 (no bias) and were used to assess the overall strength of codon usage bias [42].

### Sequence structural variation and selective pressure analyses

Contraction and expansion of IR boundaries among the four major regions (LSC/IRa/SSC/IRb) were visualised for 23 plastid genomes using CPJSdraw [43]. The nucleotide diversity (*π*) was calculated using DnaSP v6.12.03 with a window length of 800 bp and a step size of 200 bp [44]. Pairwise alignments and sequence divergence analyses of 23 *Gastrochilus* species were performed using the Pomatocalpa spicatum (MN124411) plastome as a reference, with mVISTA (https://genome.lbl.gov/cgi-bin/VistaInput? num_seqs=2) in Shuffle-LAGAN mode [45]. Plastome collinearity and rearrangements were analysed using the Mauve plugin v1.1.3 in Geneious Prime [46]. The 68 single-copy coding DNA sequences (CDSs) were extracted with Geneious Prime [34] and aligned with MAFFT v7.490 [47]. Non-synonymous (dN) and synonymous (dS) substitution rates were calculated using DnaSP [44].

### Phylogenetic analysis

A total of 25 complete plastid genomes were used for phylogenetic analysis, including 23 *Gastrochilus* genomes and two outgroup species (*Pomatocalpa spicatum*, MN124411.1 and *Holcoglossum wangii*, NC_041520.1). Phylogenetic analyses were conducted using complete plastome sequences and a concatenated dataset of 68 shared CDSs. Sequence alignments were generated using MAFFT [47]. Maximum-likelihood (ML) analyses were conducted in IQ-TREE v2.3.4, with branch support assessed using 5,000 ultrafast bootstrap (UFBoot) replicates and 5,000 SH-aLRT tests [48, 49]. The best-fitting substitution models were selected using ModelFinder implemented in IQ-TREE under the Bayesian Information Criterion (BIC) [50]: GTR + F+I+R4 for the complete plastome dataset and TVM + F+I+R4 for the concatenated dataset of 68 shared CDSs. Bayesian inference (BI) was performed using MrBayes v3.2.7a under the GTR + I+G4 model selected by ModelTest-NG v0.1.7 [51, 52]. Two independent runs, each with four Markov chains, were executed for 300,000 generations (sampled every 1,000; first 25% discarded as burn-in). Convergence was confirmed, with the average standard deviation of split frequencies approaching zero and all potential scale reduction factors close to 1.0.

## Results

### General features of plastid genomes

**Fig. 1:**
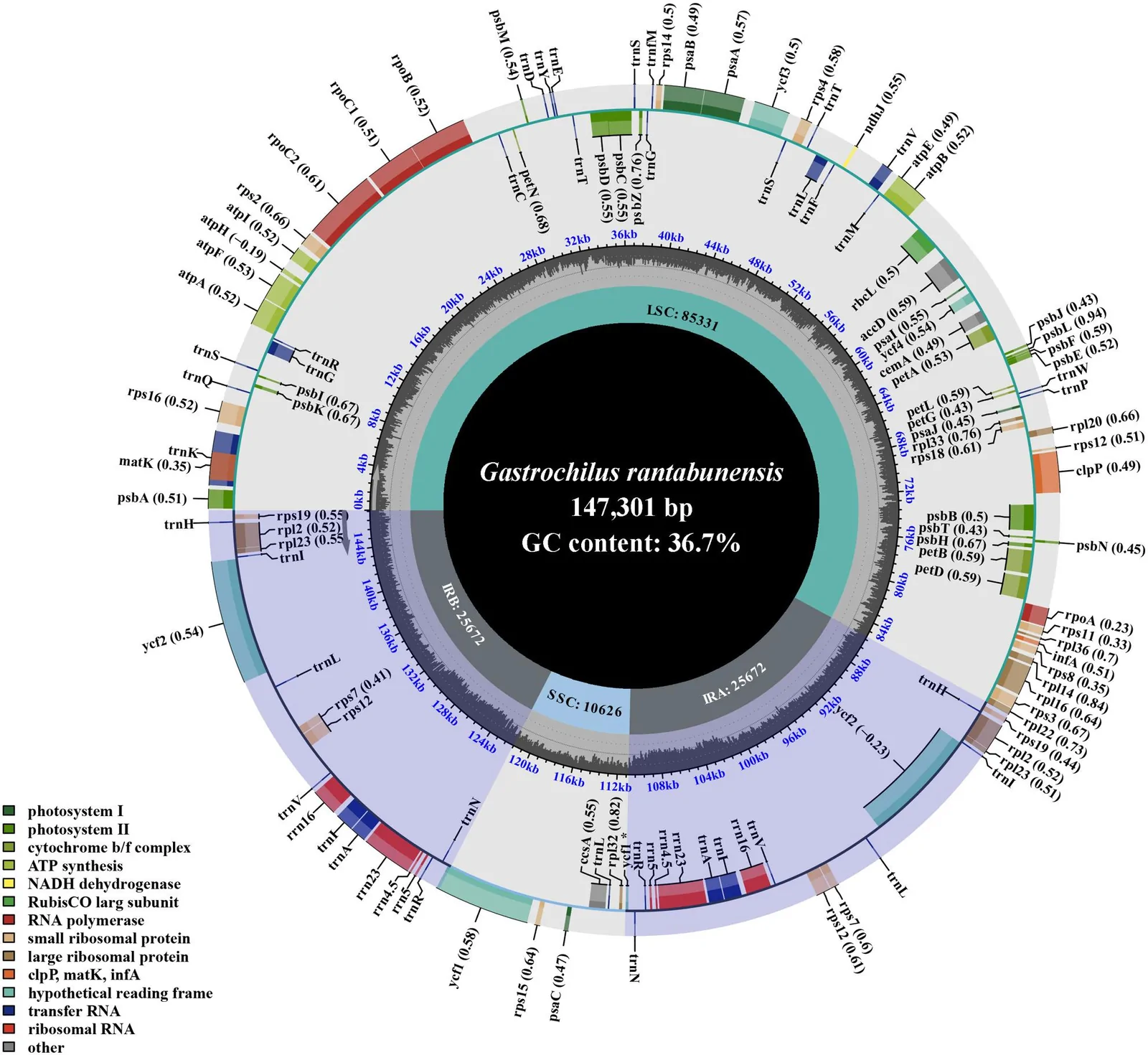
Plastid genome map of *Gastrochilus rantabunensis*. Genes are color-coded according to their functional categories and are plotted on the inner (transcribed counterclockwise) or outer (transcribed clockwise) circle. The inner gray ring visualizes the GC content across the genome. The regions between the circles depict the large single-copy (LSC), small single-copy (SSC), and the two inverted repeat (IRa and IRb) regions. The values in the center of the map represent genome size and overall GC content

The plastid genomes of the 23 *Gastrochilus* species ranged from 146,183 bp (*G. fuscopunctatus*) to 148,666 bp (*G. acinacifolius*), featuring conserved quadripartite architecture with LSC (83,125–85,759 bp), SSC (10,357–11,331 bp), and IR (25,672–26,025 bp) regions (Fig. 1 and Table S1). Universal AT-richness was observed (total GC: 36.6–36.9%), with IR regions exhibiting higher GC content (43.0–43.2%) than both LSC (33.8–34.2%) and SSC (28.0–28.6%) (Table S1).

**Fig. 2:**
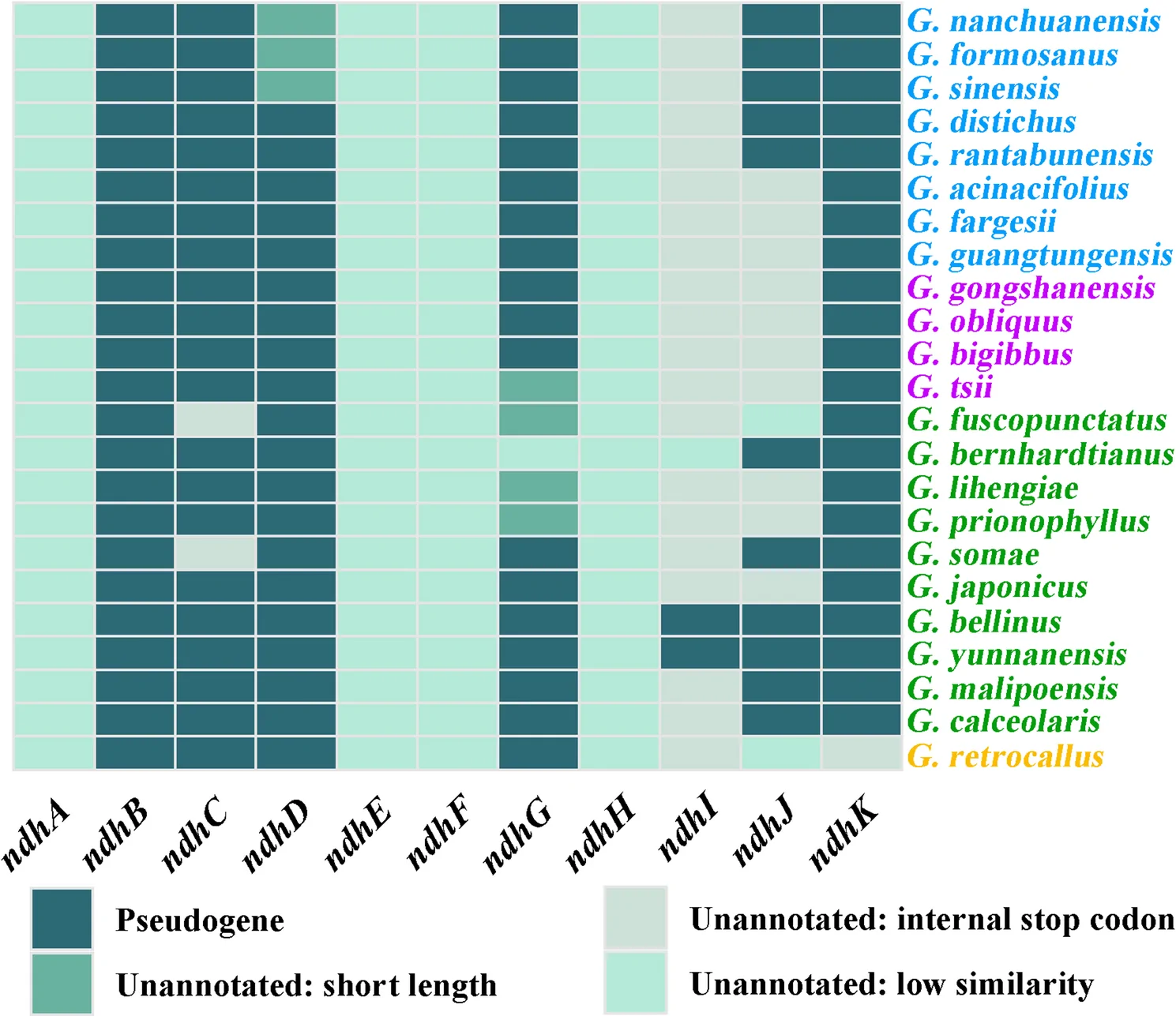
Annotation of *ndh* pseudogenes across 23 *Gastrochilus* species. Each colored box indicates a sequence was annotated as ‘Pseudogene’ or ‘Unannotated’ due to specific disabling features. Species are ordered and color-coded by phylogenetic clade (based on complete plastid genomes) to examine clade-specific patterns in pseudogene presence

Each plastome contained 120 genes, including 74 protein-coding genes, 38 tRNA genes, and 8 rRNA genes, along with 3–7 *ndh* pseudogenes (Table S1  and Fig. 2). The number of *ndh* pseudogenes varied among plastid genomes, with seven detected in two genomes, six in four, five in twelve, four in four, and three in one. Partial fragments of *ndhB*/*C*/*D*/*G*/*K* were retained in most species, whereas other potential *ndh* pseudogenes were unannotated due to low sequence similarity or internal stop codons (Fig. 2 and Table S2). Collinearity analysis also detected no gene rearrangements or inversions (Fig. S2). Overall, genomic structure and functional gene content were conserved across all species, with variation primarily in the number of *ndh* pseudogenes.

### Repeat sequences of the 23 *Gastrochilus* species

**Fig. 3:**
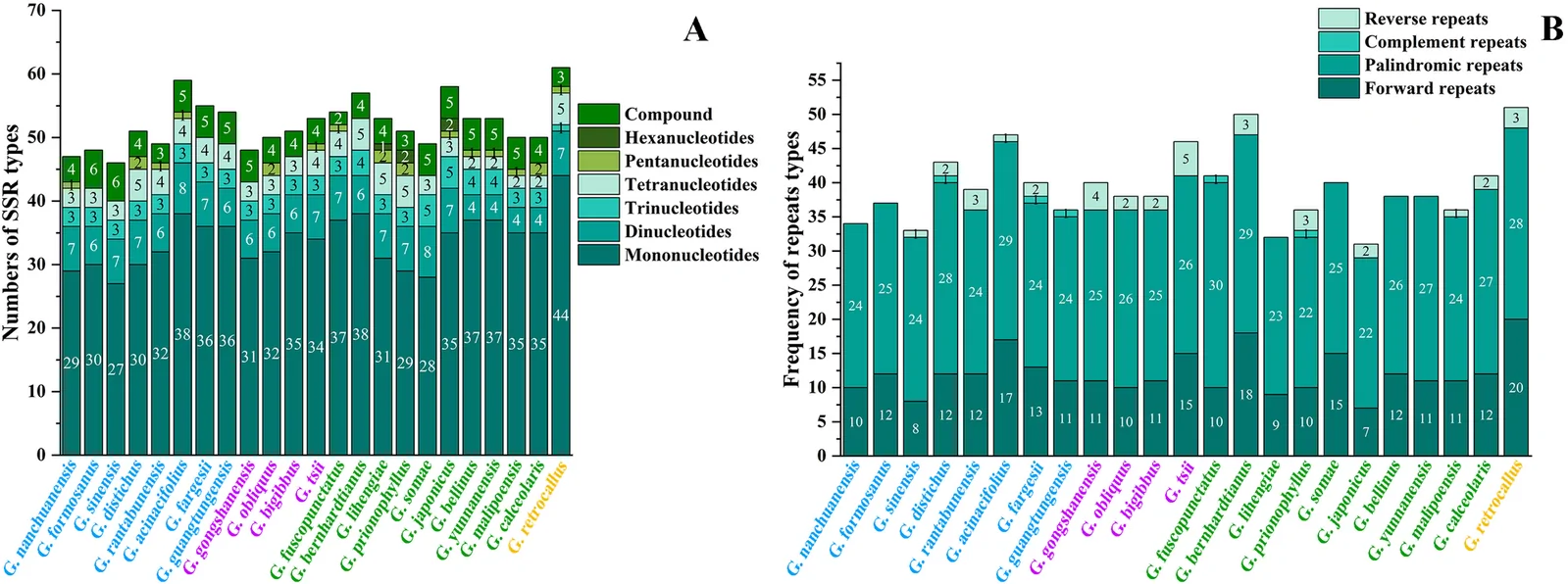
Analysis of repetitive sequences in the plastid genomes of 23 *Gastrochilus* species. **A** Numbers and types of simple sequence repeats (SSRs) in each plastid genome; **B** Numbers of dispersed repeats (DRs) in each plastid genome. Species are ordered and color-coded by phylogenetic clade based on complete plastid genomes

Plastid genome analysis of the 23 *Gastrochilus* species revealed substantial interspecific variation in SSR content. Total SSRs ranged from 46 (*G. sinensis*) to 61 (*G. retrocallus*), with mononucleotide repeats (27–44 per genome) being predominant. Hexanucleotide repeats occurred only in three species. Specifically, *G. lihengiae* contained one hexanucleotide repeat, while *G. japonicus* and *G. prionophyllus* each possessed two hexanucleotide repeats (Fig. 3A). The LSC region consistently showed the highest SSR density. Despite being longer than the SSC region, the IR region had fewer SSRs. Most plastid genomes contained a total of six SSRs across the two IR regions. However, *G*. *bernhardtianus*, *G*. *calceolaris*, *G*. *distichus*, *G*. *prionophyllus* and *G*. *tsii* each contained a total of eight SSRs across the two IR regions. SSRs within the IR regions primarily consisted of mononucleotide, dinucleotide, or trinucleotide repeats. In addition, *G. bernhardtianus* contained one tetranucleotide repeat besides the three common types, whereas *G. retrocallus* contained only mononucleotide and dinucleotide repeats in its IR regions. Compound repeats localized primarily in the LSC region, with only nine species containing such repeats in SSC region. A striking universal feature was the exclusive occurrence of A/T-type mononucleotide repeats across all plastid genomes (Table S3).

Among the 23 *Gastrochilus* plastid genomes, DRs counts ranged from 31 to 51 per species. Palindromic repeats were the most common, accounting for 55%–73% of all DRs, followed by forward repeats. Complement repeats occurred only in *G. distichus*, *G. fargesii*, *G. fuscopunctatus*, *G. guangtungensis* and *G. prionophyllus*, and reverse repeats were unevenly distributed across 15 species. Six plastid genomes contained only palindromic and forward repeats (Fig. 3B and Table S4).

### Codon usage bias in the plastid genomes of *Gastrochilus*

**Fig. 4:**
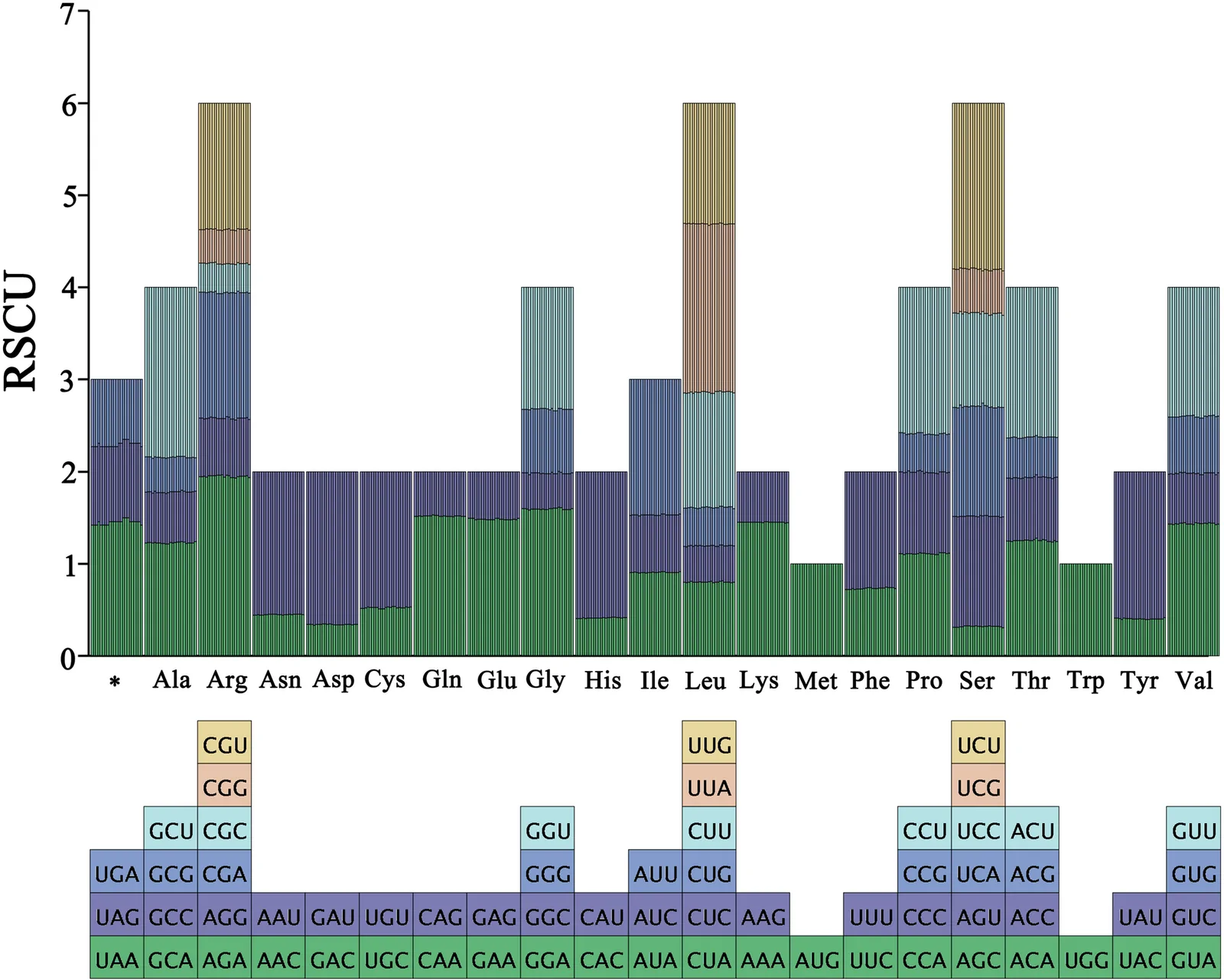
Relative synonymous codon usage (RSCU) analysis of protein-coding genes in 23 *Gastrochilus* plastid genomes. RSCU values for 20 amino acids and the stop codon are shown. Bar colors correspond to specific codons detailed in the legend below. Species are ordered by phylogenetic clade based on complete plastid genomes

Analysis of the 74 CDSs revealed 22,300–22,356 codons in the 23 plastid genomes (Table S5). The number of codons used per amino acid was consistent among these genomes (Fig. 4), indicating overall conservation of codon composition. Codon preference patterns among the 23 species showed minor variations. Five plastid genomes (*G. bernhardtianus*, *G. japonicus*, *G. lihengiae*, *G. prionophyllus* and *G. retrocallus*) exhibited 30 preference codons (RSCU > 1), whereas the remaining 18 species showed 31 preferred codons. This difference was attributable to the codon UCC, which displayed RSCU > 1 in most species but RSCU ≤ 1 in the five aforementioned genomes. With the exception of UUG (ending with G), all preferred codons (RSCU > 1) ended with A or U, while over 90% of non-preferred codons (RSCU < 1) ended with C or G (Table S5).

Codon usage bias was further assessed using the effective number of codons (ENC). The mean ENC across all genes was 46.97 ± 0.05, indicating generally weak codon usage bias across the 23 *Gastrochilus* plastid genomes (Table S6). Among individual genes, ENC values ranged from 34.95 ± 0.73 (*rps8*) to 52.83 ± 0.87 (*ycf4*) (Table S6).

### IR expansion and contraction

**Fig. 5:**
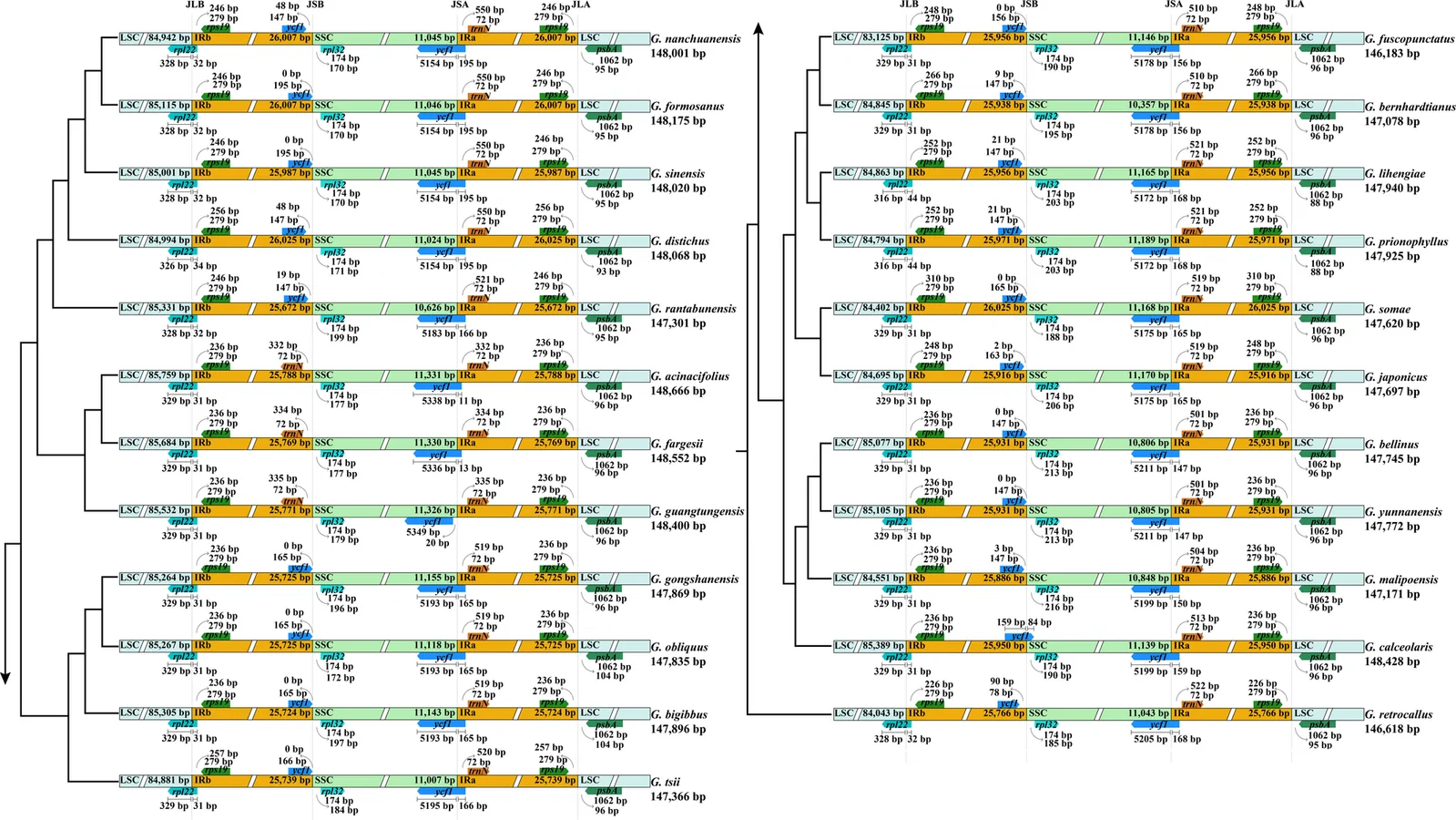
Comparison of boundary regions (LSC, SSC, and IR) among plastid genomes of 23 *Gastrochilus* species. The diagram illustrates variation at the borders of the large single-copy (LSC), small single-copy (SSC), and inverted repeat (IR) regions. Arrows indicate the distance (in base pairs) from each junction to nearby genes, detailing expansion or contraction of the IRs. The four junctions are labeled as JLB (LSC/IRb), JSB (IRb/SSC), JSA (SSC/IRa), and JLA (IRa/LSC). The tree on the left shows the ML phylogeny based on complete plastid genomes

In all 23 *Gastrochilus* plastid genomes, *rpl22* spanned the LSC and IRb regions, with 31 bp within IRb in most cases. However, some species showed shifts that extended further into IRb. These shifts included three different types: a 1-bp shift in *G. rantabunensis*, *G. formosanus*, *G. nanchuanensis*, *G. sinensis* and *G. retrocallus*, a 3-bp shift in *G. distichus*, and a 13-bp shift in *G. lihengiae* and *G. prionophyllus*. Other genes near the four junctions also showed similar positional changes. At the LSC/IRb junction, *rps19* was located 226–310 bp away from the junction. At the IRb/SSC junction, *rps32* was located 170–216 bp away from the junction. At the SSC/IRa junction, *trnN* was located 334–550 bp away from the junction, whereas *psbA* in the LSC region was located 88–104 bp away from the IRa boundary (Fig. 5).

The expansion and contraction of the IR regions had a strong effect on the location of the *ycf1* genes at both the IRb/SSC and SSC/IRa junctions. At the IRb/SSC junction, *ycf1* was annotated as a pseudogene in most species except for *G. acinacifolius*, *G. fargesii* and *G. guangtungensis*, in which this gene was not annotated. Furthermore, in *G. calceolaris*, the *ycf1* pseudogene spanned the IRb/SSC junction, whereas in the other 19 genomes, it was entirely confined to the IRb region. Turning to the SSC/IRa junction, *ycf1* spanned both regions except in *G. guangtungensis*, where it was positioned 20 bp from the IRa region (Fig. 5).

### Analysis of sequences variants 

**Fig. 6:**
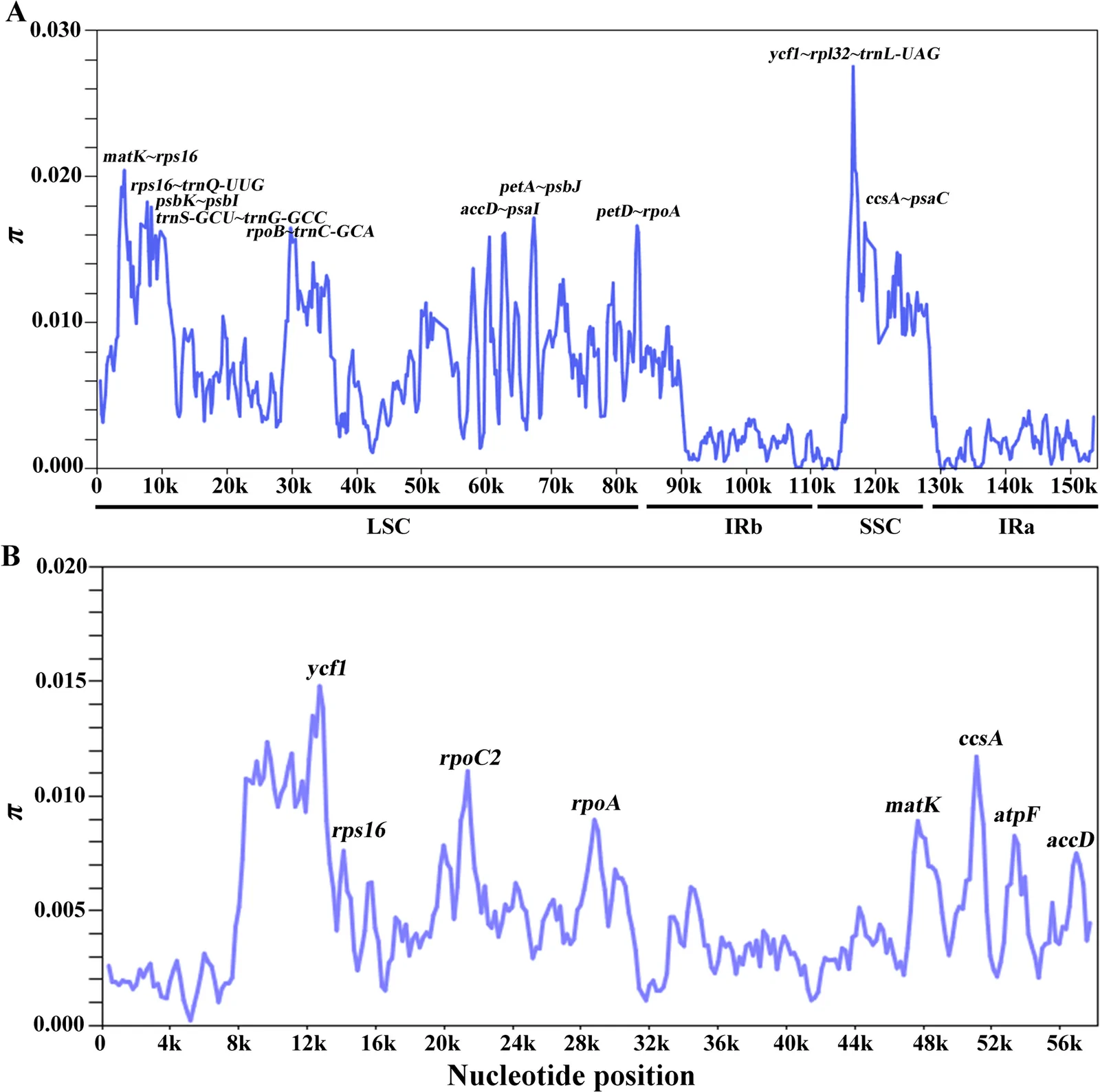
Sliding-window analysis of nucleotide diversity (*π*) in *Gastrochilu*s plastid genomes. **A** Nucleotide diversity of complete plastid genomes; **B** Nucleotide diversity of 68 protein-coding sequences. The X-axis indicates the midpoint position (bp) of each sliding window, and the Y-axis shows the *π* value for each corresponding window. The ten (in A) and eight (in B) most variable regions are annotated, representing candidate markers for phylogenetic studies

**Fig. 7:**
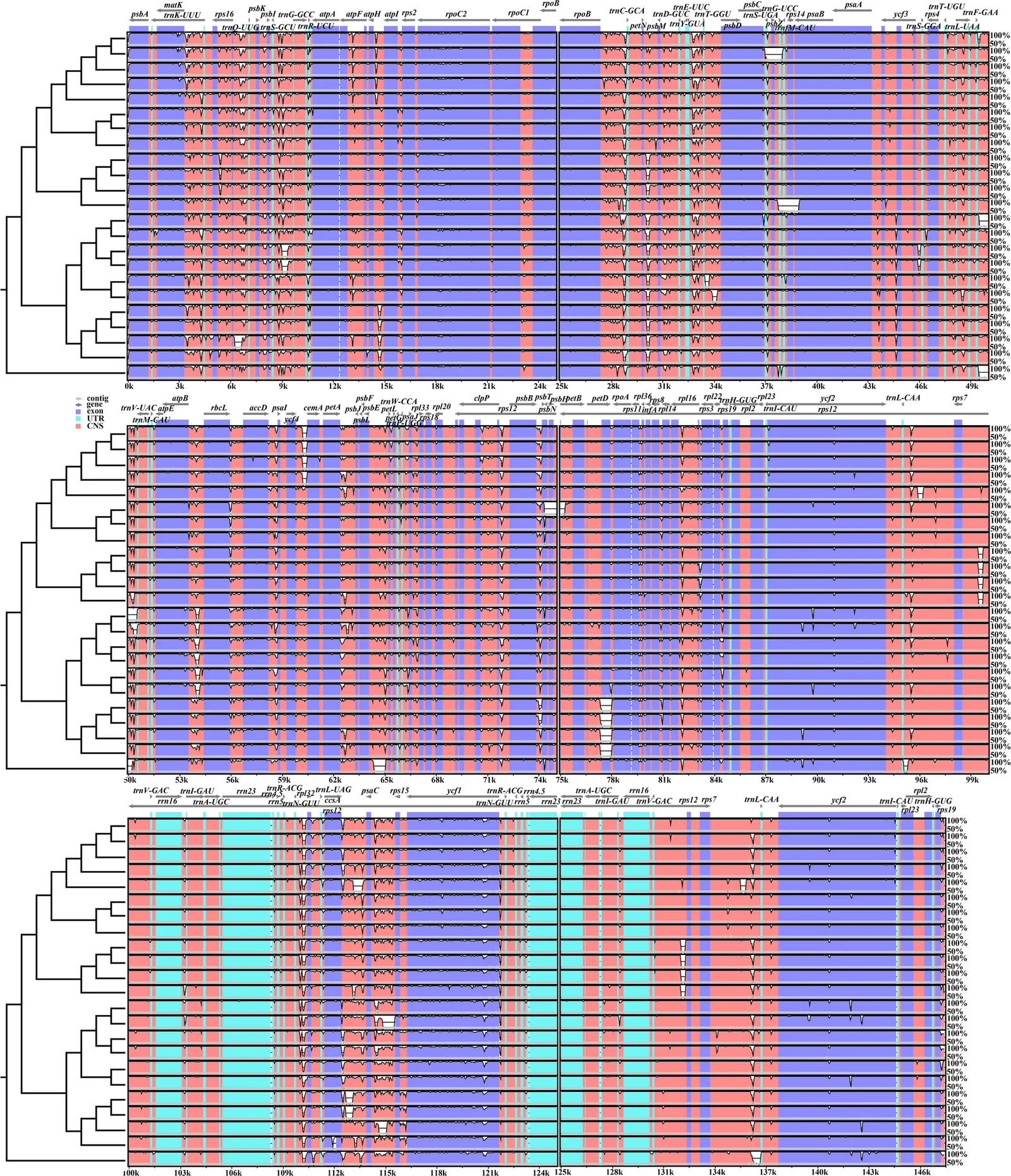
Whole-plastome alignment of 23 *Gastrochilus* species. The horizontal axis represents genome coordinates. Regions are color-coded as exons, untranslated regions (UTRs), conserved non-coding sequences (CNS), and mRNA. The tree on the left shows the ML phylogeny based on complete plastid genomes. The vertical scale indicates the percentage of identity, ranging from 50 to 100%

Comparative *π* value analysis of complete plastid genomes and CDSs across the 23 species revealed higher nucleotide diversity in whole plastid geomes (*π* = 0–0.02755) than in CDS regions (*π* = 0.00022–0.01481). Meanwhile, the LSC and SSC regions (*π* = 0.0078, 0.0085) exhibited higher *π* values than the IR regions (*π* = 0.0025). Ten hypervariable variability regions with *π >* 0.01597 were designated as hotspots. Among them, *ycf1* ~ *rpl32*~*trnL-UAG* and *ccsA*~*psaC* were located in the SSC region, while *matK*~*rps16*, *rps16* ~ *trnQ*-*UUG*, *petA~psbJ*, *petD*~*rpoA*, *rpoB*~*trnC-GCA*, *trnS-GCU*~*trnG-GCC*, *accD*~*psaI* and *psbK*~*psbI* were located in the LSC region. Additionally, eight genes (*π* > 0.00751) were identified, including *ycf1*, *rpoC2*, *ccsA*, *rpoA*, *matK*, *atpF*, *rps16* and *accD* (Fig. 6, Table S7). mVISTA alignments further validated substantial sequence divergence within these high *π* value regions (Fig. 7).

### Selection pressure analysis

**Fig. 8:**
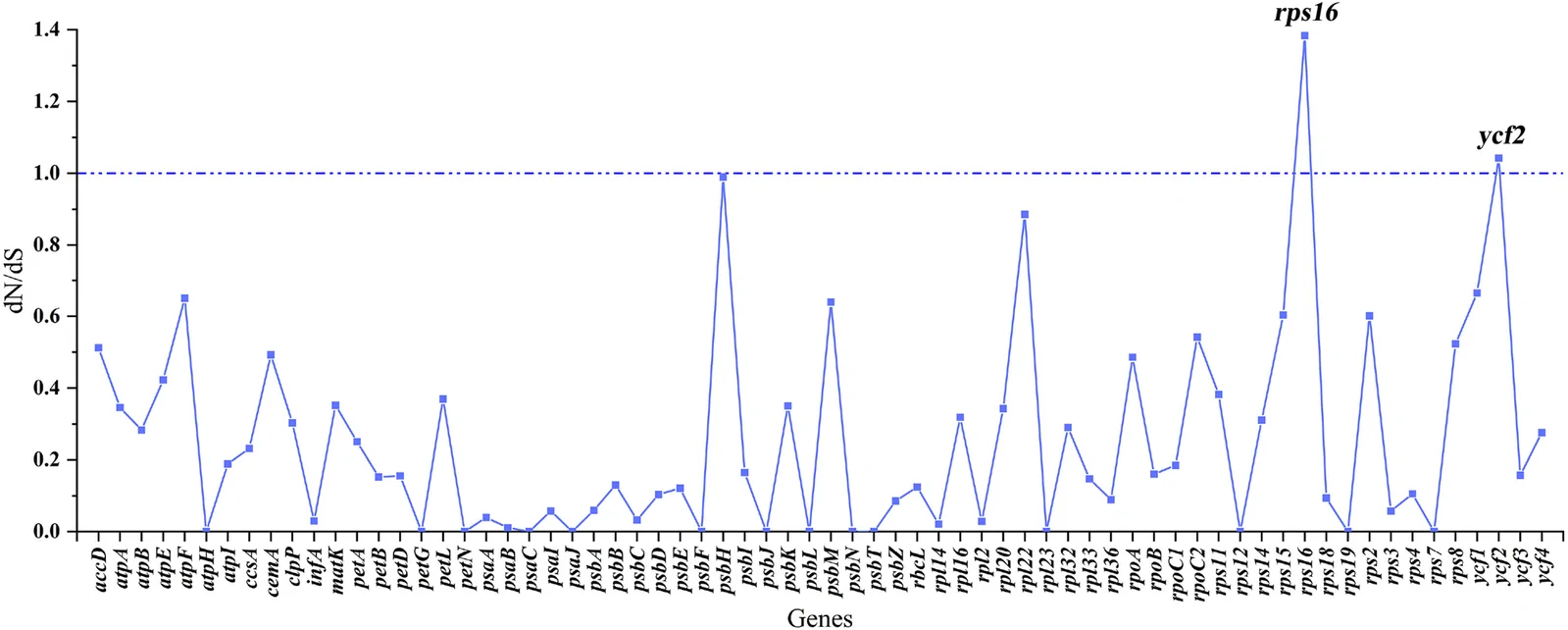
Estimates of selective pressure (dN/dS) for protein-coding genes in the plastid genomes of 23 *Gastrochilus species*. A dashed line at dN/dS = 1 marks the threshold for neutral evolution, with ratios below 1 suggesting purifying (negative) selection and those above 1 indicating positive selection

The average values of dN, dS, and dN/dS for all 68 protein coding genes were 0.00256, 0.01047 and 0.09257, respectively (Table S8). Sixty-six genes had dN/dS values of less than 1, indicating that they were primarily subject to purifying selection. Only *rps16* and *ycf2* had dN/dS values above 1, suggesting that they may have been subject to positive selection (Fig. 8). Furthermore, the results revealed that 8 and 78 positively selected sites in *rps16* and *ycf2*, respectively, of which 3 and 63 altered the amino acid products of translation (Fig. S3 and Fig. S4). In particular, for *rps16*, a premature stop codon was observed in 11 *Gastrochilus* species (Fig. S3).

### Phylogenetic relationships

**Fig. 9:**
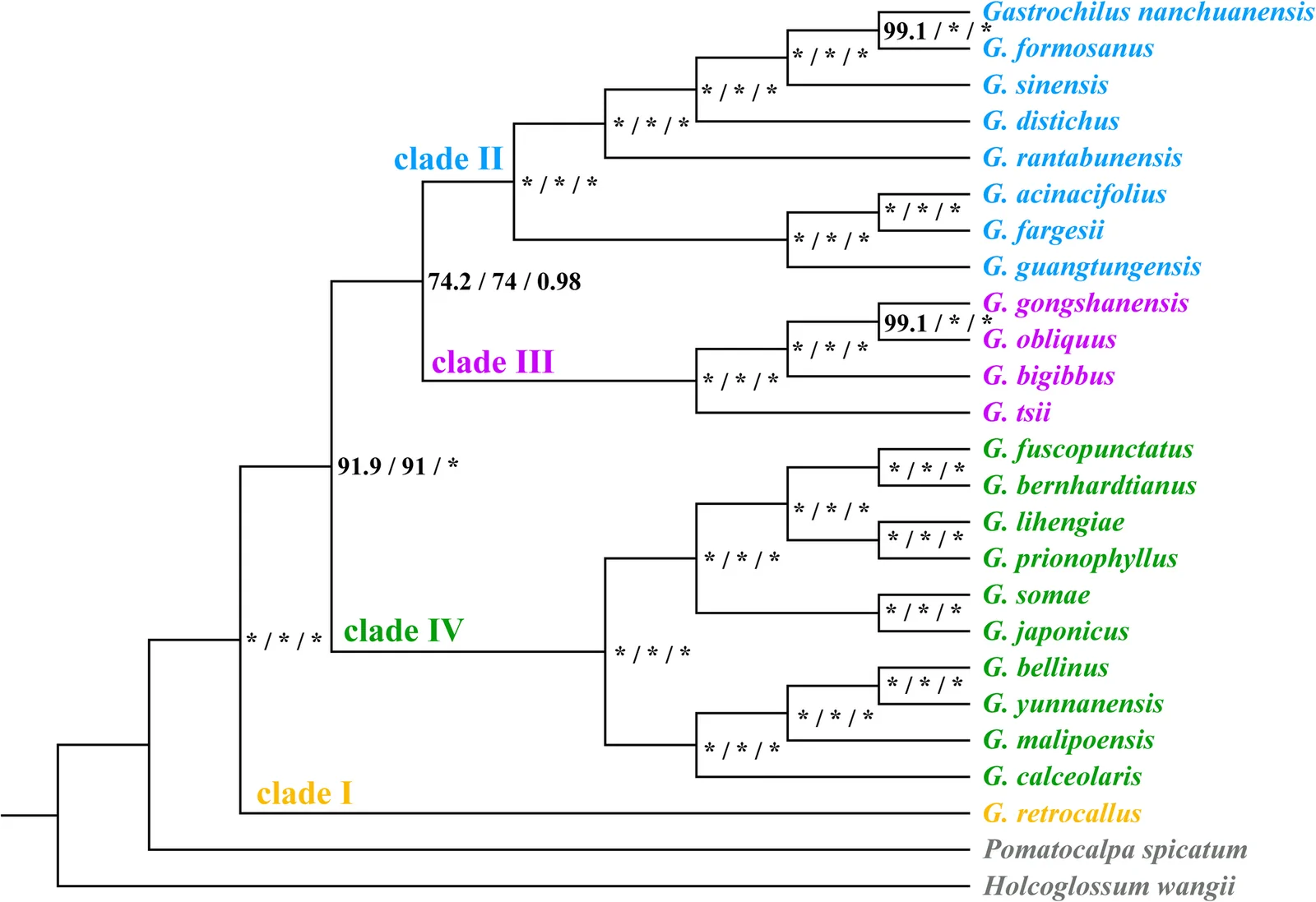
Maximum likelihood (ML) phylogenetic tree of *Gastrochilus* inferred from complete plastid genome sequences. Topology was estimated using the ML method. Nodal support values are shown as UFBoot / SH-aLRT / Bayesian posterior probability (BPP). Asterisks (*) indicate 100% bootstrap or 1.00 BPP

All plastome-based phylogenetic analyses consistently recovered *Gastrochilus* as a monophyletic group, sister to *Pomatocalpa*. Within *Gastrochilus*, *G. retrocallus* was resolved as the earliest-diverging lineage (Clade I) with strong support (Fig. 9). The remaining species were grouped into three major clades (Clades II–IV). The ML and BI analyses of the complete plastome dataset were identical, with most internal nodes receiving high support, although the relationships between the two major clades were only moderately supported (Fig. 9).

In contrast, the ML tree inferred from the concatenated 68 CDSs showed a different topology of Clade III (Fig. S4). Specifically, plastome-wide analyses supported Clade III as sister to Clade II, whereas the CDS-based ML analysis supported Clade III as the sister of Clade IV (Fig. S5). Notably, the corresponding nodes in the CDS-based tree received substantially lower support (UFBoot/SH-aLRT = 72.9/48 and 69.4/49), indicating reduced phylogenetic resolution in the CDS dataset and suggesting that inter-clade relationships are sensitive to data selection (Fig. S5).

## Discussion

### Structural conservation of plastid genomes in *Gastrochilus*

Consistent with other angiosperms, *Gastrochilus* plastid genomes possess the typical quadripartite circular structure (LSC, SSC, and two IR regions) [53]. The highly similar GC content and CDS gene numbers among the 23 species indicate conserved plastid genomes (Table S1). Comparative analysis further revealed highly conserved genomic architecture across *Gastrochilus* species, with no major structural rearrangements detected (Fig. S2). Genome length and GC content did not exhibit any clear patterns of association with phylogenetic relationships, suggesting that these variations may be largely stochastic or influenced by other factors. The lack of distinguishable features in genome length, GC content, and gene number between *Gastrochilus* and its close relatives (*Pomatocalpa*, *Acampe*, *Staurochilus*, and *Trichoglottis*) provide definitive evidence for structural conservation across this lineage [19].

### Loss of *ndh* genes and potential ecological associations

Among the notable genomic features, the universal loss or pseudogenization of *ndh* genes in *Gastrochilus* plastid genomes presents an intriguing evolutionary phenomenon. The functional significance of the NDH complex (encoded by 11 *ndh* genes) in photosystem I cyclic electron transport and chlororespiration renders its widespread loss particularly noteworthy [54–56]. However, the compensatory mechanisms observed or suggested in other orchid and angiosperm lineages are potential strategies, not necessarily universal features across all orchids. First, in *Paphiopedilum* species, mitochondrial transfer of *ndh* genes has been documented [22]. Second, some orchids like *Phalaenopsis equestris* maintain normal physiology despite lacking *ndh* homologs across all genomes [23]. Third, alternative pathways involving PGRL1-PGR5 complexes have been proposed as functional substitutes [57, 58].

This extensive loss of *ndh* genes in orchids may be correlated with their mycorrhizal symbiotic relationships [23, 59], as the loss in leafless *Corallorhiza* and *Gastrodia* plastid genomes exceeding that observed in leafy orchids [60, 61]. Environmental pressures also appear significant. The secondary cause of *ndh* gene loss in *Gastrochilus* plastid genomes could be linked to their epiphytic/lithophytic habitats in forest canopies or understories [1]. During adaptation to these environments, plants may gradually weaken their demand for responding to dynamic light stress, a trait also observed in some heterotrophic orchids [62, 63]. Supporting the role of environmental stress, studies in Cypripedioideae plastid genomes suggest that light and temperature stresses in tropical regions may contribute to the functional loss of the NDH complex [20]. Additionally, a potential link to photosynthetic metabolism exists. Research has suggested that the loss of *ndh* genes in the plastid genomes of *Littorella* could be linked to its partial dependence on the Crassulacean acid metabolism (CAM) pathway [64]. Intriguingly, within the *Cleisostoma*-*Gastrochilus* clade, where *ndh* genes are widely lost or pseudogenized, the majority of species are constitutive CAM plants [19]. However, the specific mechanistic relationship between CAM evolutionary pathways and plastid genomic variations remains unresolved. 

Notably, regarding the phylogenetic distribution within *Gastrochilus*, our analysis revealed no parallel patterns between *ndh* gene loss and the early- or later-diverging positions of clades within the genus. This suggests that within this genus, the loss events may not be strictly tied to its major evolutionary divergences or might have occurred prior to or independently of the diversification events defining the early- or later-diverging groups.

### Repeat sequence variation and plastome mutation hotspots

Our analysis of 23 *Gastrochilus* species identified between 46 and 61 SSRs per plastid genome, highlighting their widespread presence and interspecific variability (Fig. 3). Mononucleotide repeats were the most abundant, accounting for 57%–72% of SSRs, with hexanucleotide repeats observed in only three species. The repeat units were predominantly composed of A or T, with all mononucleotide repeats belonging to the A/T type (Table S3). This distribution likely reflects the AT-rich composition characteristic of *Gastrochilus* plastid genomes. These results were similar to prior SSRs distribution patterns reported in 16 *Gastrochilus* species and related genera such as *Polystachya*, *Phalaenopsis*, *Paphiopedilum*, and *Epidendrum* [65–69]. Within this study, 31 to 51 DRs were identified across the 23 *Gastrochilus* plastid genomes, revealing extensive variation among these repeat sequences (Fig. 3). Palindromic repeats were the most frequent, while complementary repeats showed the lowest frequency, a pattern similar to that observed in species such as *Phalaenopsis*, *Paphiopedilum* and *Epidendrum* species [65, 67, 68]. At the clade level, no clear patterns were detected in SSR or dispersed repeat abundance or composition, with variation primarily occurring at the species level (Fig. 3). For instance, *G. retrocallus* showed abundant SSRs, while hexanucleotides appeared in a small subset of species like *G. lihengiae.* In summary, we have identified 1,200 SSRs and 905 DRs within the plastid genomes of *Gastrochilus* (Table S3 and Table S4). These molecular markers hold potential for application in population genetic analyses and complementary phylogenetic studies, providing useful resources for species-level genetic differentiation.

### Codon usage bias in *Gastrochilus* plastid genomes

Codon usage bias reflects the non-random usage of synonymous codons and is influenced by a combination of nucleotide composition and evolutionary constraints [70]. Across all the 23 *Gastrochilus* species, the preferred codons for each amino acid were identical, with a consistent preference for A/U-ending codons (Table S5). Similar A/U-ending preferences have been widely reported in plastid genomes of Orchidaceae and other angiosperms, and are generally associated with the overall AT-richness of plastid genomes or selection pressure [66, 71–73]. Generally, ENC values ≤ 35 are considered indicative of relatively high codon usage bias [42, 73, 74]. In this study, only *rps8* showed low ENC values, whereas the overall ENC across all genes was 46.97 ± 0.05 (Table S6). The small standard deviation indicates that codon usage patterns are highly similar among the 23 *Gastrochilus* plastid genomes, suggesting limited and conserved codon usage bias across species. Notably, we observed that the codon UCC had RSCU ≤ 1 (0.9954–1) only in *G. retrocallus* and clade IV species (Table S5). Given the overall weak codon usage bias and the highly conserved nature of plastid genomes, the observed variation in UCC usage appears to be limited in scope. Further investigation will be required to clarify the evolutionary factors underlying this pattern.

### Structural variation and IR boundary dynamics

The contraction and expansion of IR regions represent primary mechanisms driving plastid genome size variation in Orchidaceae, a phenomenon extensively documented in the family [75, 76]. For instance, complete IR loss has been observed in *Gastrodia elata* and *Aphyllorchis montana* due to genome reduction, while IR expansion towards the SSC region yielded elongated IRs (29,808–32,683 bp) in *Pogonia* and *Vanilla* [22, 77]. In contrast to these pronounced IR region variations observed in other orchid plastid genomes, *Gastrochilus* species exhibit minimal changes. However, significant boundary shifts were observed at the SSC region, particularly involving the two *ycf1* genes. Phylogenetic analysis revealed that *G. acinacifolius*, *G. fargesii*, and *G. guangtungensis* within Clade II exhibited IR contraction, leading to gradual reduction of the *ycf1* segment in IRa until complete relocation to SSC. Concurrently, the residual *ycf1* pseudogene in IRb became progressively truncated and ultimately lost (Fig. 5). Nevertheless, our findings on IR boundary dynamics in *Gastrochilus* were insufficient to elucidate the underlying mechanisms driving its variation. Therefore, additional morphological and ecological data are necessary for a more precise and targeted investigation.

### Sequence divergence and selection signals in plastid genes

Consistent with previous studies on *Gastrochilus*, IR regions demonstrated higher conservation than LSC and SSC regions, with intergenic spacers exhibiting greater variability than coding sequences [4]. We identified ten hypervariable regions and eight highly polymorphic genes in *Gastrochilus* plastid genomes (Fig. 6). The majority of these 18 variable regions, including *ycf1* ~ *rpl32*~*trnL*-*UAG*, *trnS*-*GCU*~*trnG*-*GCC*, *accD*~*psaI*, *matK*~*rps16*, *ycf1*, *ccsA*, *matK*, *rps16*, and *rps16* ~ *trnQ*-*UUG*, have been similarly reported as mutation hotspots across diverse orchid lineages [69, 78–80]. Notably, all hypervariable regions identified in this study closely matched those previously reported in an analysis of 16 *Gastrochilus* plastid genomes [4]. The consistent interspecific divergence in these regions supports them as promising candidate regions for future species discrimination and population-level studies, pending further validation using broader sampling and intraspecific analyses.

Selection pressure analysis revealed contrasting evolutionary patterns. Specifically, two genes (*rps16*, *ycf2*) exhibited signatures of positive selection (dN/dS > 1), while 66 genes underwent purifying selection (dN/dS < 1) (Fig. 8). The gene *ycf2* encodes a core component of the Ycf2/FtsHi complex, which plays an important role in plastid ATP synthesis, particularly under low-light or non-photosynthetic conditions [81]. This finding aligns with documented positive selection patterns in *ycf2* from shade-adapted *Lysimachia* and *Heliconia* species [82, 83]. Collectively, these data suggest that *ycf2* in *Gastrochilus* has likely undergone adaptive evolution in response to low-light forest environments. Although *rps16* (encoding ribosomal protein S16) is typically conserved in angiosperm plastid genomes, it frequently undergoes loss or pseudogenization in genera such as *Populus*, *Medicago*, and *Hypericum*. This recurrent phenomenon has established *rps16* as a focal gene for studying plastid-nuclear genome coevolution [84, 85]. However, reports of positive selection acting on plastid *rps16* are relatively rare across plants, and the mechanistic understanding remains limited even in documented cases such as Rutaceae [86]. Intriguingly, premature termination codons in *rps16* were exclusively detected within Clade IV and *G. rantabunensis*, exhibiting partial phylogenetic congruence with clade membership.

### Plastome phylogeny and implications for infrageneric relationships

Persistent controversies regarding infrageneric relationships in *Gastrochilus* have been attributed to limited taxon sampling, inconsistent molecular markers, and potential historical interspecific hybridization within Aeridinae [4, 9, 13, 19, 87]. Using complete plastid genome sequences and expanded sampling, both ML and BI analyses consistently resolved the 23 *Gastrochilus* species into four major clades. This topology is congruent with previous plastome-based phylogenies [4], but shows substantially improved nodal support and phylogenetic resolution. Notably, the sister relationship between Clade III and Clade IV, which previously received weak Bayesian support (BPP = 0.55) [4], now shows substantially increased support (UFBoot/SH-aLRT/BPP = 74.2 / 74 / 0.98). Plastome-wide phylogenies provide stronger internal nodes than CDS-based trees, likely reflecting a history of rapid diversification and potential lineage sorting or introgression events [9], which have been widely documented in Orchidaceae and other angiosperm groups. In such evolutionary scenarios, datasets with limited phylogenetically informative sites, such as concatenated CDSs, may be insufficient to resolve short internal branches. Further integration of nuclear genomic data will be essential to disentangle these processes and reconcile organellar and nuclear phylogenetic signals.

At the intra-clade level, relationships within each major clade were generally well supported in the plastome-wide phylogeny. By contrast, the backbone node defining the sister-group relationship of Clade III with other major clades received only moderate support, reflecting uncertainty in the placement of Clade III rather than in its internal structure. Additional taxon sampling, particularly from closely related lineages, may help further stabilize this deeper-level relationship.

This study reports the first complete plastid genome of *G. rantabunensis* and clarifies its phylogenetic position using whole-plastome data. By incorporating all available *Gastrochilus* plastid genomes, we provide the most comprehensive plastome-based phylogeny of the genus to date. Our results are largely consistent with recent plastome-based studies, except for the newly added genomes [4]. Additionally, discrepancies among published phylogenies are mainly due to differences in molecular marker selection and research focus [9, 13].

Phylogenetic relationships, genomic features, and ecological information together provide important insights into historical biogeography [9, 20]. In this study, variation in the *rps16* and IR region contraction are two genomic features with potential associations to phylogenetic position. Future studies that integrate species distribution modelling, expanded taxonomic sampling, and multi-omics datasets will be essential for disentangling the roles of hybridization and adaptive evolution in shaping diversification patterns in *Gastrochilus*.

## Conclusion

This study provides the first comprehensive analysis of plastome evolution across 23 *Gastrochilus* species. Our results demonstrate highly conserved genomic structures in these species with no major structural rearrangements. The loss of *ndh* genes and positive selection in *ycf2* ‌potentially reflect‌ adaptation to low-light forest environments. The identified hypervariable regions and repeat sequences offer powerful molecular markers for species delimitation and phylogenetic inference. Phylogenomic analyses resolved all species into four well-supported clades, offering a robust framework for infrageneric classification. Together, these findings advance the understanding of the evolution of *Gastrochilus* plastid genomes and provide key resources for exploring the ecological and adaptive significance of genomic variation.

## Supplementary information

Supplementary Material 1

## Data Availability

All data supporting this study are publicly available. The complete chloroplast genome sequence of Gastrochilus rantabunensis has been deposited in NCBI. Accession numbers for comparative datasets are provided in Table S1. The datasets supporting the conclusions of this article are included within the article and its additional files.

## Abbreviations

BI: Bayesian inference
BS: Bootstrap values
CDS: Coding DNA sequence
dN: Non-synonymous
DRs: Dispersed repeats
dS: Synonymous
IR: Inverted repeat
LSC: Large single copy
ML: Maximum likelihood
mRNA: Messenger RNA
rRNA: Ribosomal RNA
RSCU: Relative synonymous codon usage
SSC: Small single copy
SSRs: Simple sequence repeats
tRNA: Transfer RNA
*π*: Nucleotide diversity
