# Supplementary material for: Comparative plastid genomics of Gastrochilus (Orchidaceae) with insights into molecular markers and phylogeny
Source: BMC Genomics. 2026 Jun 26;27:777. doi: 10.1186/s12864-026-12698-8 (PMC13599090; doi:10.1186/s12864-026-12698-8)
Supplement: Supplementary file 1 [file 12864_2026_12698_MOESM2_ESM.zip › Supplementary material/Fig. S1.pdf]

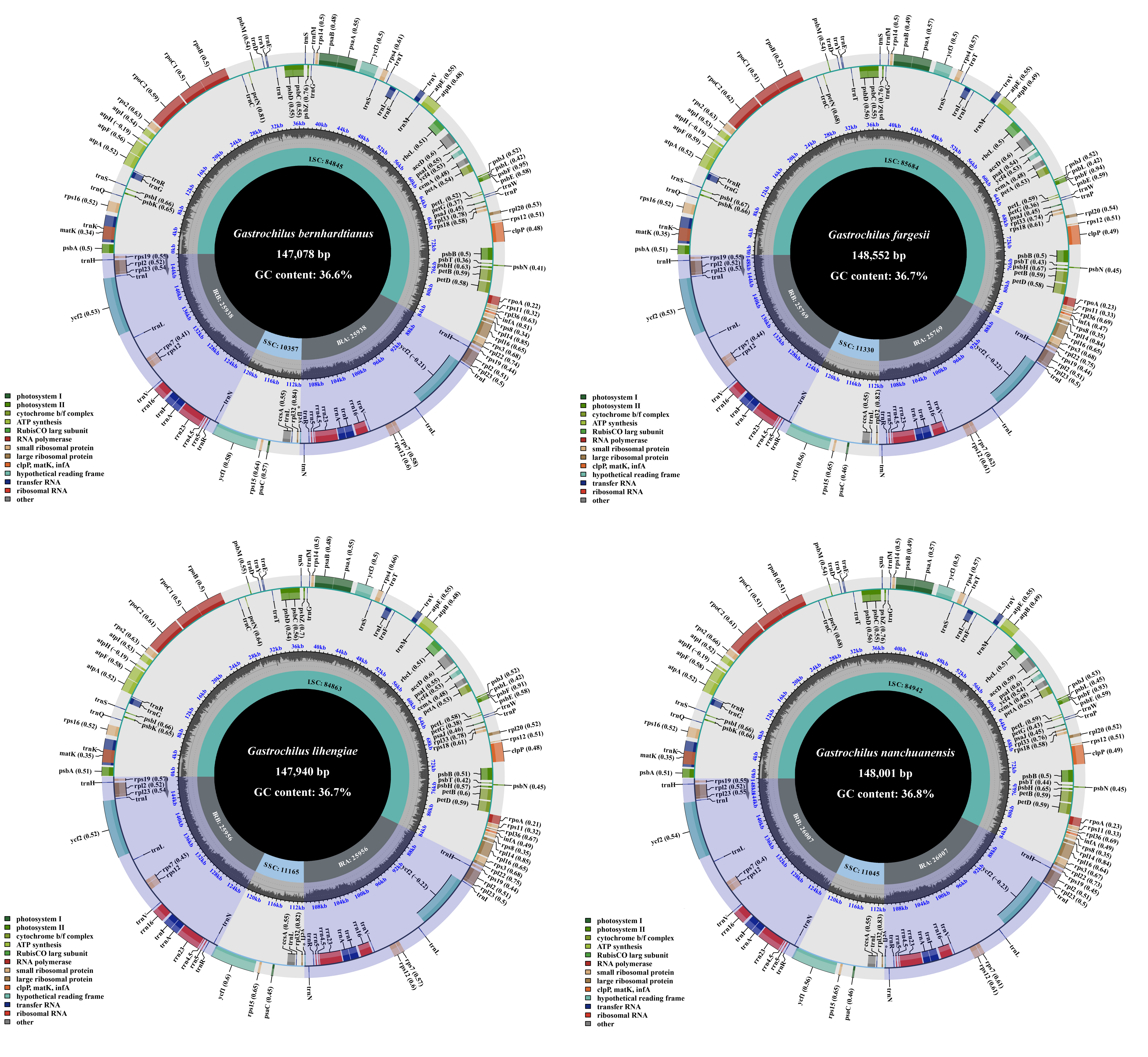

Gastrochilus bernhardtianus
147,078 bp
GC content: 36.6%
LSC: 84845
IRA: 25938
SSC: 10357
IRB: 25938
Gastrochilus fargesii
148,552 bp
GC content: 36.7%
LSC: 85684
IRA: 25769
SSC: 11330
IRB: 25769
Gastrochilus lihengiae
147,940 bp
GC content: 36.7%
LSC: 84863
IRA: 25956
SSC: 11165
IRB: 25956
Gastrochilus nanchuanensis
148,001 bp
GC content: 36.8%
LSC: 84942
IRA: 26007
SSC: 11045
IRB: 26007
photosystem I
photosystem II
cytochrome b/f complex
ATP synthesis
RubisCO larg subunit
RNA polymerase
small ribosomal protein
large ribosomal protein
clpP, matK, infA
hypothetical reading frame
transfer RNA
ribosomal RNA
other
