# Supplementary material for: Comparative plastid genomics of Gastrochilus (Orchidaceae) with insights into molecular markers and phylogeny
Source: BMC Genomics. 2026 Jun 26;27:777. doi: 10.1186/s12864-026-12698-8 (PMC13599090; doi:10.1186/s12864-026-12698-8)
Supplement: Supplementary file 1 [file 12864_2026_12698_MOESM2_ESM.zip › Supplementary material/Fig. S2.pdf]

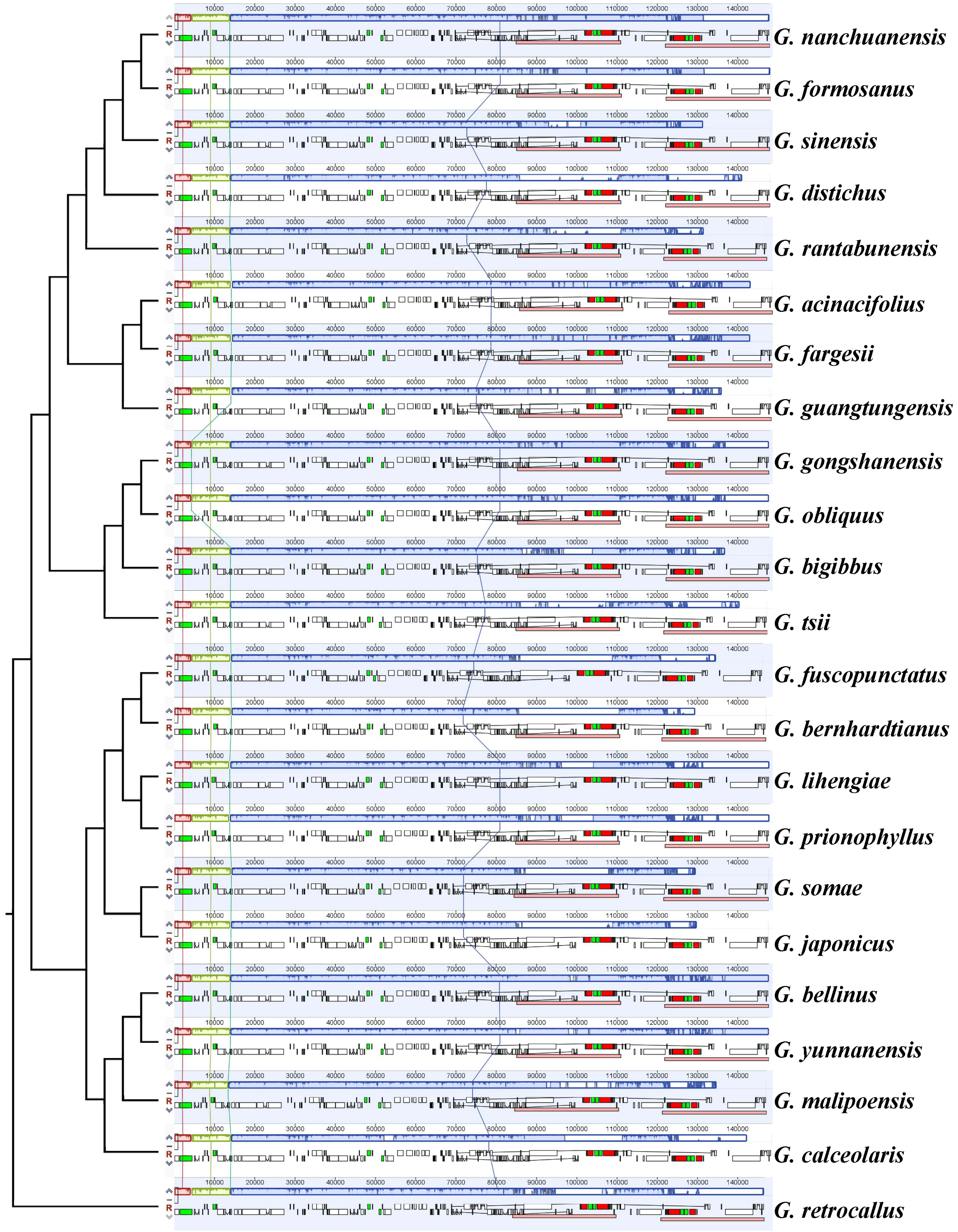

G. nanchuanensis
G. formosanus
G. sinensis
G. distichus
G. rantabunensis
G. acinacifolius
G. fargesii
G. guangtungensis
G. gongshanensis
G. obliquus
G. bigibbus
G. tsii
G. fuscopunctatus
G. bernhardtianus
G. lihengiae
G. prionophyllus
G. somae
G. japonicus
G. bellinus
G. yunnanensis
G. malipoensis
G. calceolaris
G. retrocallus
