# Supplementary material for: Comparative plastid genomics of Gastrochilus (Orchidaceae) with insights into molecular markers and phylogeny
Source: BMC Genomics. 2026 Jun 26;27:777. doi: 10.1186/s12864-026-12698-8 (PMC13599090; doi:10.1186/s12864-026-12698-8)
Supplement: Supplementary file 1 [file 12864_2026_12698_MOESM2_ESM.zip › Supplementary material/Fig. S3.pdf]

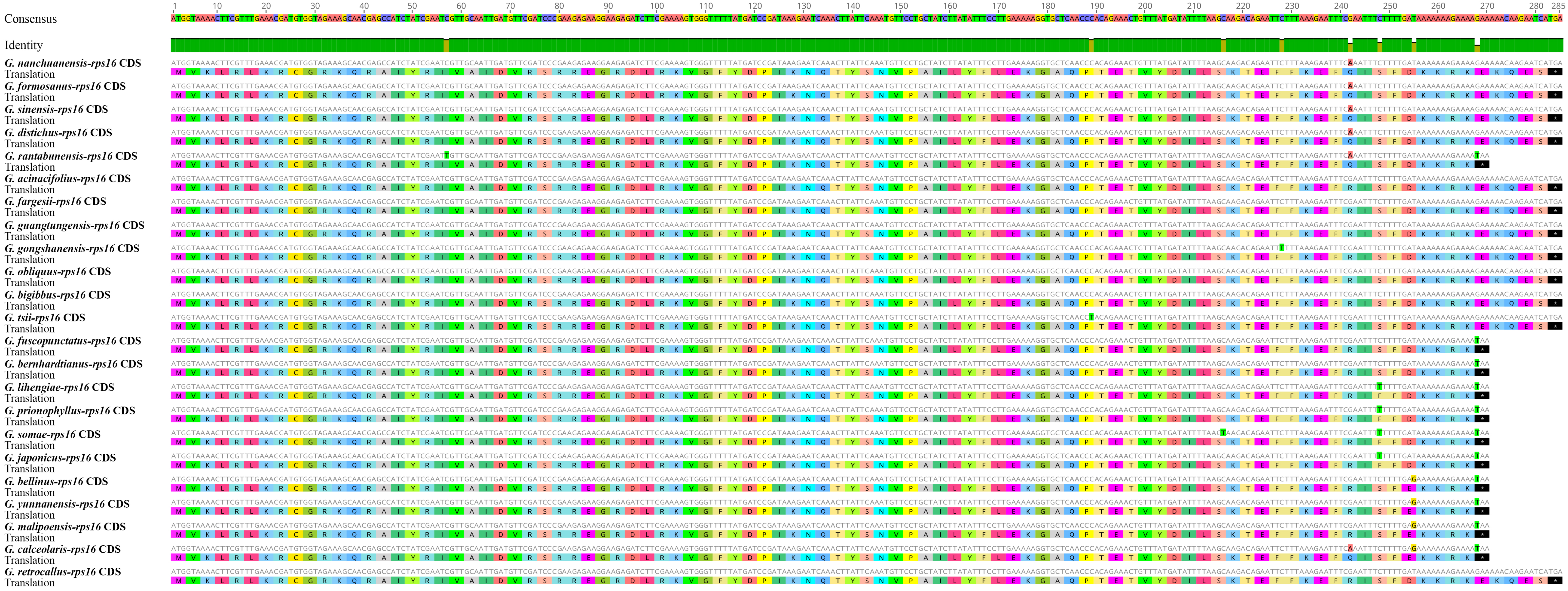
Consensus
Identity
G. nanchuanensis-rps16 CDS
Translation
G. formosanus-rps16 CDS
Translation
G. sinensis-rps16 CDS
Translation
G. distichus-rps16 CDS
Translation
G. rantabunensis-rps16 CDS
Translation
G. acinacifolius-rps16 CDS
Translation
G. fargesii-rps16 CDS
Translation
G. guangtungensis-rps16 CDS
Translation
G. gongshanensis-rps16 CDS
Translation
G. obliquus-rps16 CDS
Translation
G. bigibbus-rps16 CDS
Translation
G. tsii-rps16 CDS
Translation
G. fuscopunctatus-rps16 CDS
Translation
G. bernhardtianus-rps16 CDS
Translation
G. lihengiae-rps16 CDS
Translation
G. prionophyllus-rps16 CDS
Translation
G. somae-rps16 CDS
Translation
G. japonicus-rps16 CDS
Translation
G. bellinus-rps16 CDS
Translation
G. yunnanensis-rps16 CDS
Translation
G. malipoensis-rps16 CDS
Translation
G. calceolaris-rps16 CDS
Translation
G. retrocallus-rps16 CDS
Translation
