# Supplementary material for: Comparative plastid genomics of Gastrochilus (Orchidaceae) with insights into molecular markers and phylogeny
Source: BMC Genomics. 2026 Jun 26;27:777. doi: 10.1186/s12864-026-12698-8 (PMC13599090; doi:10.1186/s12864-026-12698-8)
Supplement: Supplementary file 1 [file 12864_2026_12698_MOESM2_ESM.zip › Supplementary material/fig. S4.pdf]

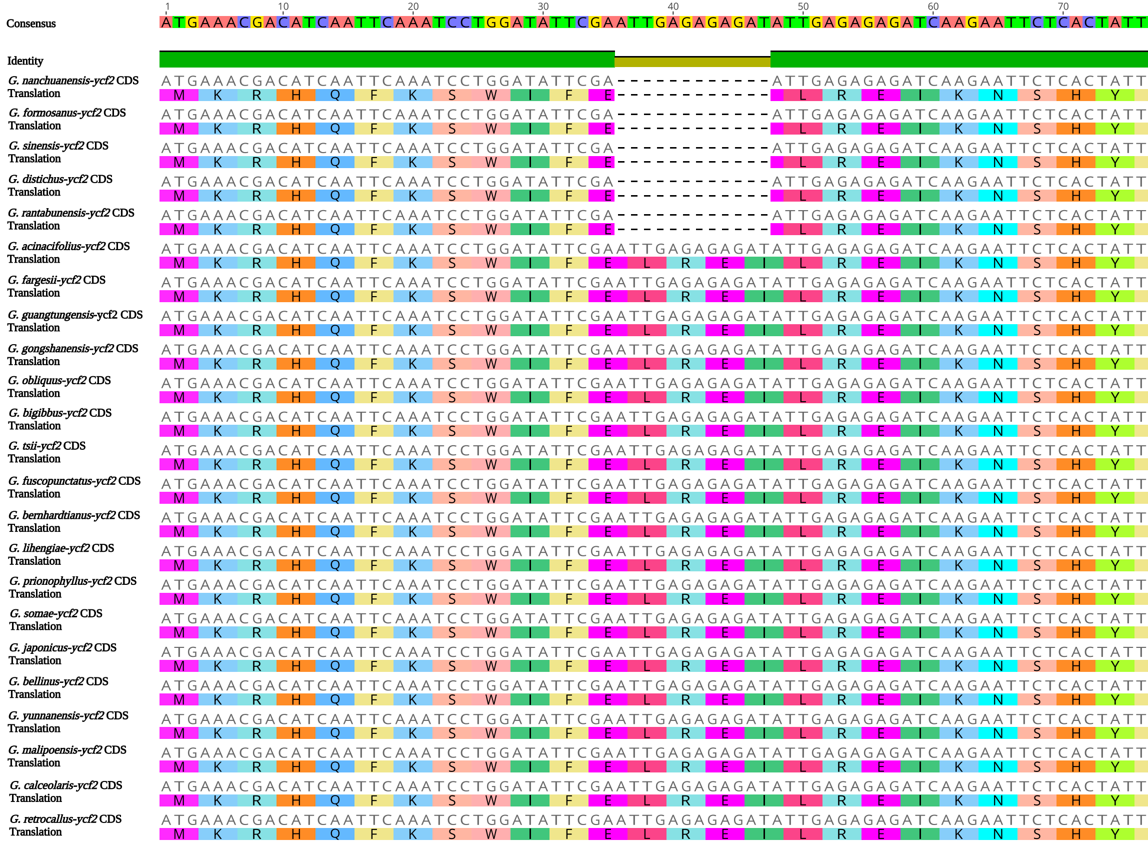
1 10 20 30 40 50 60 70
Consensus
ATGAAACGACATCAATTCAAATCCTGGATATTCGAATTGAGAGAGATATTGAGAGAGATCAAGAATTCTCACTATT
Identity
G. nanchuanensis-ycf2 CDS
ATGAAACGACATCAATTCAAATCCTGGATATTCGA------------ATTGAGAGAGATCAAGAATTCTCACTATT
Translation
M K R H Q F K S W I F E ---- L R E I K N S H Y
G. formosanus-ycf2 CDS
ATGAAACGACATCAATTCAAATCCTGGATATTCGA------------ATTGAGAGAGATCAAGAATTCTCACTATT
Translation
M K R H Q F K S W I F E ---- L R E I K N S H Y
G. sinensis-ycf2 CDS
ATGAAACGACATCAATTCAAATCCTGGATATTCGA------------ATTGAGAGAGATCAAGAATTCTCACTATT
Translation
M K R H Q F K S W I F E ---- L R E I K N S H Y
G. distichus-ycf2 CDS
ATGAAACGACATCAATTCAAATCCTGGATATTCGA------------ATTGAGAGAGATCAAGAATTCTCACTATT
Translation
M K R H Q F K S W I F E ---- L R E I K N S H Y
G. rantabunensis-ycf2 CDS
ATGAAACGACATCAATTCAAATCCTGGATATTCGA------------ATTGAGAGAGATCAAGAATTCTCACTATT
Translation
M K R H Q F K S W I F E ---- L R E I K N S H Y
G. acinacifolius-ycf2 CDS
ATGAAACGACATCAATTCAAATCCTGGATATTCGAATTGAGAGAGATATTGAGAGAGATCAAGAATTCTCACTATT
Translation
M K R H Q F K S W I F E L R E I L R E I K N S H Y
G. fargesii-ycf2 CDS
ATGAAACGACATCAATTCAAATCCTGGATATTCGAATTGAGAGAGATATTGAGAGAGATCAAGAATTCTCACTATT
Translation
M K R H Q F K S W I F E L R E I L R E I K N S H Y
G. guangtungensis-ycf2 CDS
ATGAAACGACATCAATTCAAATCCTGGATATTCGAATTGAGAGAGATATTGAGAGAGATCAAGAATTCTCACTATT
Translation
M K R H Q F K S W I F E L R E I L R E I K N S H Y
G. gongshanensis-ycf2 CDS
ATGAAACGACATCAATTCAAATCCTGGATATTCGAATTGAGAGAGATATTGAGAGAGATCAAGAATTCTCACTATT
Translation
M K R H Q F K S W I F E L R E I L R E I K N S H Y
G. obliquus-ycf2 CDS
ATGAAACGACATCAATTCAAATCCTGGATATTCGAATTGAGAGAGATATTGAGAGAGATCAAGAATTCTCACTATT
Translation
M K R H Q F K S W I F E L R E I L R E I K N S H Y
G. bigibbus-ycf2 CDS
ATGAAACGACATCAATTCAAATCCTGGATATTCGAATTGAGAGAGATATTGAGAGAGATCAAGAATTCTCACTATT
Translation
M K R H Q F K S W I F E L R E I L R E I K N S H Y
G. tsii-ycf2 CDS
ATGAAACGACATCAATTCAAATCCTGGATATTCGAATTGAGAGAGATATTGAGAGAGATCAAGAATTCTCACTATT
Translation
M K R H Q F K S W I F E L R E I L R E I K N S H Y
G. fuscopunctatus-ycf2 CDS
ATGAAACGACATCAATTCAAATCCTGGATATTCGAATTGAGAGAGATATTGAGAGAGATCAAGAATTCTCACTATT
Translation
M K R H Q F K S W I F E L R E I L R E I K N S H Y
G. bernhardtianus-ycf2 CDS
ATGAAACGACATCAATTCAAATCCTGGATATTCGAATTGAGAGAGATATTGAGAGAGATCAAGAATTCTCACTATT
Translation
M K R H Q F K S W I F E L R E I L R E I K N S H Y
G. lihengiae-ycf2 CDS
ATGAAACGACATCAATTCAAATCCTGGATATTCGAATTGAGAGAGATATTGAGAGAGATCAAGAATTCTCACTATT
Translation
M K R H Q F K S W I F E L R E I L R E I K N S H Y
G. prionophyllus-ycf2 CDS
ATGAAACGACATCAATTCAAATCCTGGATATTCGAATTGAGAGAGATATTGAGAGAGATCAAGAATTCTCACTATT
Translation
M K R H Q F K S W I F E L R E I L R E I K N S H Y
G. somae-ycf2 CDS
ATGAAACGACATCAATTCAAATCCTGGATATTCGAATTGAGAGAGATATTGAGAGAGATCAAGAATTCTCACTATT
Translation
M K R H Q F K S W I F E L R E I L R E I K N S H Y
G. japonicus-ycf2 CDS
ATGAAACGACATCAATTCAAATCCTGGATATTCGAATTGAGAGAGATATTGAGAGAGATCAAGAATTCTCACTATT
Translation
M K R H Q F K S W I F E L R E I L R E I K N S H Y
G. bellinus-ycf2 CDS
ATGAAACGACATCAATTCAAATCCTGGATATTCGAATTGAGAGAGATATTGAGAGAGATCAAGAATTCTCACTATT
Translation
M K R H Q F K S W I F E L R E I L R E I K N S H Y
G. yunnanensis-ycf2 CDS
ATGAAACGACATCAATTCAAATCCTGGATATTCGAATTGAGAGAGATATTGAGAGAGATCAAGAATTCTCACTATT
Translation
M K R H Q F K S W I F E L R E I L R E I K N S H Y
G. malipoensis-ycf2 CDS
ATGAAACGACATCAATTCAAATCCTGGATATTCGAATTGAGAGAGATATTGAGAGAGATCAAGAATTCTCACTATT
Translation
M K R H Q F K S W I F E L R E I L R E I K N S H Y
G. calceolaris-ycf2 CDS
ATGAAACGACATCAATTCAAATCCTGGATATTCGAATTGAGAGAGATATTGAGAGAGATCAAGAATTCTCACTATT
Translation
M K R H Q F K S W I F E L R E I L R E I K N S H Y
G. retrocallus-ycf2 CDS
ATGAAACGACATCAATTCAAATCCTGGATATTCGAATTGAGAGAGATATTGAGAGAGATCAAGAATTCTCACTATT
Translation
M K R H Q F K S W I F E L R E I L R E I K N S H Y

80 90 100 110 120 130 140 150 160

TCTTAGATTCATGGATCAAATTCGATTCAGTGGGATCTTTCACTCACATTTTTTTCCACCAAGAACGTTTTATGAAACTCTTTGACCC

TCTTAGATTCATGGATCAAATTCGATTCAGTGGGATCTTTCACTCACATTTTTTTCCACCAAGAACGTTTTATGAAACTCTTTGACCC
F L D S W I K F D S V G S F T H I F F H Q E R F M K L F D P

TCTTAGATTCATGGATCAAATTCGATTCAGTGGGATCTTTCACTCACATTTTTTTCCACCAAGAACGTTTTATGAAACTCTTTGACCC
F L D S W I K F D S V G S F T H I F F H Q E R F M K L F D P

TCTTAGATTCATGGATCAAATTCGATTCAGTGGGATCTTTCACTCACATTTTTTTCCACCAAGAACGTTTTATGAAACTCTTTGACCC
F L D S W I K F D S V G S F T H I F F H Q E R F M K L F D P

TCTTAGATTCATGGATCAAATTCGATTCAGTGGGATCTTTCACTCACATTTTTTTCCACCAAGAACGTTTTATGAAACTCTTTGACCC
F L D S W I K F D S V G S F T H I F F H Q E R F M K L F D P

TCTTAGATTCATGGATCAAATTCGATTCAGTGGGATCTTTCACTCACATTTTTTTCCACCAAGAACGTTTTATGAAACTCTTTGACCC
F L D S W I K F D S V G S F T H I F F H Q E R F M K L F D P

TCTTAGATTCATGGATCAAATTCGATTCAGTGGGATCTTTCACTCACATTTTTTTCCACCAAGAACGTTTTATGAAACTCTTTGACCC
F L D S W I K F D S V G S F T H I F F H Q E R F M K L F D P

TCTTAGATTCATGGATCAAATTCGATTCAGTGGGATCTTTCACTCACATTTTTTTCCACCAAGAACGTTTTATGAAACTCTTTGACCC
F L D S W I K F D S V G S F T H I F F H Q E R F M K L F D P

TCTTAGATTCATGGATCAAATTCGATTCAGTGGGATCTTTCACTCACATTTTTTTCCACCAAGAACGTTTTATGAAACTCTTTGACCC
F L D S W I K F D S V G S F T H I F F H Q E R F M K L F D P

TCTTAGATTCATGGATCAAATTCGATTCAGTGGGATCTTTCACTCACATTTTTTTCCACCAAGAACGTTTTATGAAACTCTTTGACCC
F L D S W I K F D S V G S F T H I F F H Q E R F M K L F D P

TCTTAGATTCATGGATCAAATTCGATTCAGTGGGATCTTTCACTCACATTTTTTTCCACCAAGAACGTTTTATGAAACTCTTTGACCC
F L D S W I K F D S V G S F T H I F F H Q E R F M K L F D P

TCTTAGATTCATGGATCAAATTCGATTCAGTGGGATCTTTCACTCACATTTTTTTCCACCAAGAACGTTTTATGAAACTCTTTGACCC
F L D S W I K F D S V G S F T H I F F H Q E R F M K L F D P

TCTTAGATTCATGGATCAAATTCGATTCAGTGGGATCTTTCACTCACATTTTTTTCCACCAAGAACGTTTTATGAAACTCTTTGACCC
F L D S W I K F D S V G S F T H I F F H Q E R F M K L F D P

TCTTAGATTCATGGATCAAATTCGATTCAGTGGGATCTTTCACTCACATTTTTTTCCACCAAGAACGTTTTATGAAACTCTTTGACCC
F L D S W I K F D S V G S F T H I F F H Q E R F M K L F D P

TCTTAGATTCATGGATCAAATTCGATTCAGTGGGATCTTTCACTCACATTTTTTTCCACCAAGAACGTTTTATGAAACTCTTTGACCC
F L D S W I K F D S V G S F T H I F F H Q E R F M K L F D P

TCTTAGATTCATGGATCAAATTCGATTCAGTGGGATCTTTCAGTCACATTTTTTTCCACCAAGAACGTTTTATGAAACTCTTTGACCC
F L D S W I K F D S V G S F S H I F F H Q E R F M K L F D P

TCTTAGATTCATGGATCAAATTCGATTCAGTGGGATCTTTCAGTCACATTTTTTTCCACCAAGAACGTTTTATGAAACTCTTTGACCC
F L D S W I K F D S V G S F S H I F F H Q E R F M K L F D P

TCTTAGATTCATGGATCAAATTCGATTCAGTGGGATCTTTCACTCACATTTTTTTCCACCAAGAACGTTTTATGAAACTCTTTGACCC
F L D S W I K F D S V G S F T H I F F H Q E R F M K L F D P

TCTTAGATTCATGGATCAAATTCGATTCAGTGGGATCTTTCACTCACATTTTTTTCCACCAAGAACGTTTTATGAAACTCTTTGACCC
F L D S W I K F D S V G S F T H I F F H Q E R F M K L F D P

TCTTAGATTCATGGATCAAATTCGATTCAGTGGGATCTTTCACTCACATTTTTTTCCACCAAGAACGTTTTATGAAACTCTTTGACCC
F L D S W I K F D S V G S F T H I F F H Q E R F M K L F D P

TCTTAGATTCATGGATCAAATTCGATTCAGTGGGATCTTTCACTCACATTTTTTTCCACCAAGAACGTTTTATGAAACTCTTTGACCC
F L D S W I K F D S V G S F T H I F F H Q E R F M K L F D P

TCTTAGATTCATGGATCAAATTCGATTCAGTGGGATCTTTCACTCACATTTTTTTCCACCAAGAACGTTTTATGAAACTCTTTGACCC
F L D S W I K F D S V G S F T H I F F H Q E R F M K L F D P

TCTTAGATTCATGGATCAAATTTGATTCAGTGGGATCTTTCACTCACATTTTTTTCCACCAAGAACGTTTTATGAAACTCTTTGACCC
F L D S W I K F D S V G S F T H I F F H Q E R F M K L F D P

TCTTAGATTCATGGATCAAATTCGATTCAGTGGGATCTTTCACTCACATTTTTTTCCACCAAGAACGTTTTATGAAACTCTTTGACCC
F L D S W I K F D S V G S F T H I F F H Q E R F M K L F D P

```
170       180       190       200       210       220       230       240
CCGAATTTGGAGTATCCTACTTTCACGTGATTCACAGGGTGCAACAAGCAATCGATATTTCACGATCAAAGGTTTAGTACTG

CCGAATTTGGAGTATCCTACTTTCACGTGATTCACAGGGTGCAACAAGCAATCGATATTTCACGATCAAAGGTTTAGTACTG
 R  I  W  S  I  L  L  S  R  D  S  Q  G  A  T  S  N  R  Y  F  T  I  K  G  L  V  L
CCGAATTTGGAGTATCCTACTTTCACGTGATTCACAGGGTGCAACAAGCAATCGATATTTCACGATCAAAGGTTTAGTACTG
 R  I  W  S  I  L  L  S  R  D  S  Q  G  A  T  S  N  R  Y  F  T  I  K  G  L  V  L
CCGAATTTGGAGTATCCTACTTTCACGTGATTCACAGGGTGCAACAAGCAATCGATATTTCACGATCAAAGGTTTAGTACTG
 R  I  W  S  I  L  L  S  R  D  S  Q  G  A  T  S  N  R  Y  F  T  I  K  G  L  V  L
CCGAATTTGGAGTATCCTACTTTCACGTGATTCACAGGGTGCAACAAGCAATCGATATTTCACGATCAAAGGTTTAGTACTG
 R  I  W  S  I  L  L  S  R  D  S  Q  G  A  T  S  N  R  Y  F  T  I  K  G  L  V  L
CCGAATTTGGAGTATCCTACTTTCACGTGATTCACAGGGTGCAACAAGCAATCGATATTTCACGATCAAAGGTTTAGTACTG
 R  I  W  S  I  L  L  S  R  D  S  Q  G  A  T  S  N  R  Y  F  T  I  K  G  L  V  L
CCGAATTTGGAGTATCCTACTTTCACGTGATTCACAGGGTGCAACAAGCAATCGATATTTCACGATCAAAGGTTTAGTACTG
 R  I  W  S  I  L  L  S  R  D  S  Q  G  A  T  S  N  R  Y  F  T  I  K  G  L  V  L
CCGAATTTGGAGTATCCTACTTTCACGTGATTCACAGGGTGCAACAAGCAATCGATATTTCACGATCAAAGGTTTAGTACTG
 R  I  W  S  I  L  L  S  R  D  S  Q  G  A  T  S  N  R  Y  F  T  I  K  G  L  V  L
CCGAATTTGGAGTATCCTACTTTCACGTGATTCACAGGGTGCAACAAGCAATCGATATTTCACGATCAAAGGTTTAGTACTG
 R  I  W  S  I  L  L  S  R  D  S  Q  G  A  T  S  N  R  Y  F  T  I  K  G  L  V  L
CCGAATTTGGAGTATCCTACTTTCACGTGATTCACAGGGTGCAACAAGCAATCGATATTTCACGATCAAAGGTTTAGTACTG
 R  I  W  S  I  L  L  S  R  D  S  Q  G  A  T  S  N  R  Y  F  T  I  K  G  L  V  L
CCGAATTTGGAGTATCCTACTTTCACGTGATTCACAGGGTGCAACAAGCAATCGATATTTCACGATCAAAGGTTTAGTACTG
 R  I  W  S  I  L  L  S  R  D  S  Q  G  A  T  S  N  R  Y  F  T  I  K  G  L  V  L
CCGAATTTGGAGTATCCTACTTTCACGTGATTCACAGGGTGCAACAAGCAATCGATATTTCACGATCAAAGGTTTAGTACTG
 R  I  W  S  I  L  L  S  R  D  S  Q  G  A  T  S  N  R  Y  F  T  I  K  G  L  V  L
CCGAATTTGGAGTATCCTACTTTCACGTGATTCACAGGGTGCAACAAGCAATCGATATTTCACGATCAAAGGTTTAGTACTG
 R  I  W  S  I  L  L  S  R  D  S  Q  G  A  T  S  N  R  Y  F  T  I  K  G  L  V  L
CCGAATTTGGAGTATCCTACTTTCACGTGATTCACAGGGTGCAACAAGCAATCGATATTTCACGATCAAAGGTTTAGTACTG
 R  I  W  S  I  L  L  S  R  D  S  Q  G  A  T  S  N  R  Y  F  T  I  K  G  L  V  L
CCGAATTTGGAGTATCCTACTTTCACGTGATTCACAGGGTGCAACAAGCAATCGATATTTCACGATCAAAGGTTTAGTACTG
 R  I  W  S  I  L  L  S  R  D  S  Q  G  A  T  S  N  R  Y  F  T  I  K  G  L  V  L
CCGAATTTGGAGTATCCTACTTTCACGTGATTCACAGGGTGCAACAAGCAATCGATATTTCACGATCAAAGGTTTAGTACTG
 R  I  W  S  I  L  L  S  R  D  S  Q  G  A  T  S  N  R  Y  F  T  I  K  G  L  V  L
CCGAATTTGGAGTATCCTACTTTCACGTGATTCACAGGGTGCAACAAGCAATCGATATTTCACGATCAAAGGTTTAGTACTG
 R  I  W  S  I  L  L  S  R  D  S  Q  G  A  T  S  N  R  Y  F  T  I  K  G  L  V  L
CCGAATTTGGAGTATCCTACTTTCACGTGATTCACAGGGTGCAACAAGCAATCGATATTTCACGATCAAAGGTTTAGTACTG
 R  I  W  S  I  L  L  S  R  D  S  Q  G  A  T  S  N  R  Y  F  T  I  K  G  L  V  L
CCGAATTTGGAGTATCCTACTTTCACGTGATTCACAGGGTGCAACAAGCAATCGATATTTCACGATCAAAGGTTTAGTACTG
 R  I  W  S  I  L  L  S  R  D  S  Q  G  A  T  S  N  R  Y  F  T  I  K  G  L  V  L
CCGAATTTGGAGTATCCTACTTTCACGTGATTCACAGGGTGCAACAAGCAATCGATATTTCACGATCAAAGGTTTAGTACTG
 R  I  W  S  I  L  L  S  R  D  S  Q  G  A  T  S  N  R  Y  F  T  I  K  G  L  V  L
CCGAATTTGGAGTATCCTACTTTCACGTGATTCACAGGGTGCAACAAGCAATCGATATTTCACGATCAAAGGTTTAGTACTG
 R  I  W  S  I  L  L  S  R  D  S  Q  G  A  T  S  N  R  Y  F  T  I  K  G  L  V  L
CCGAATTTGGAGTATCCTACTTTCACGTGATTCACAGGGTGCAACAAGCAATCGATATTTCACGATCAAAGGTTTAGTACTG
 R  I  W  S  I  L  L  S  R  D  S  Q  G  A  T  S  N  R  Y  F  T  I  K  G  L  V  L
CCGAATTTGGAGTATCCTACTTTCACGTGATTCACAGGGTGCAACAAGCAATCGATATTTCACGATCAAAGGTTTAGTACTG
 R  I  W  S  I  L  L  S  R  D  S  Q  G  A  T  S  N  R  Y  F  T  I  K  G  L  V  L
CCGAATTTGGAGTATCCTACTTTCACGTGATTCACAGGGTGCAACAAGCAATCGATATTTCACGATCAAAGGTTTAGTACTG
 R  I  W  S  I  L  L  S  R  D  S  Q  G  A  T  S  N  R  Y  F  T  I  K  G  L  V  L
CCGAATTTGGAGTATCCTACTTTCACGTGATTCACAGGGTGCAACAAACAATCGATATTTCACGATCAAAGGTTTAGTACTG
 R  I  W  S  I  L  L  S  R  D  S  Q  G  A  T  N  N  R  Y  F  T  I  K  G  L  V  L
```

```
250       260       270       280       290       300       310       320       330
CTTGTAGTAGTGGTCCTTCTATCTCGTATTAACAATCGAAAGATGGTCGAAAGAAAAAATCTCTATTTGATGGGGCTTCTTCCTATAC

CTTGTAGTAGTGGTCCTTCTATCTCGTATTAACAATCGAAAGATGGTCGAAAGAAAAAATCTCTATTTGATGGGGCTTCTTCCTATAC
 L  V  V  V  V  L  L  S  R  I  N  N  R  K  M  V  E  R  K  N  L  Y  L  M  G  L  L  P  I
CTTGTAGTAGTGGTCCTTCTATCTCGTATTAACAATCGAAAGATGGTCGAAAGAAAAAATCTCTATTTGATGGGGCTTCTTCCTATAC
 L  V  V  V  V  L  L  S  R  I  N  N  R  K  M  V  E  R  K  N  L  Y  L  M  G  L  L  P  I
CTTGTAGTAGTGGTCCTTCTATCTCGTATTAACAATCGAAAGATGGTCGAAAGAAAAAATCTCTATTTGATGGGGCTTCTTCCTATAC
 L  V  V  V  V  L  L  S  R  I  N  N  R  K  M  V  E  R  K  N  L  Y  L  M  G  L  L  P  I
CTTGTAGTAGTGGTCCTTCTATCTCGTATTAACAATCGAAAGATGGTCGAAAGAAAAAATCTCTATTTGATGGGGCTTCTTCCTATAC
 L  V  V  V  V  L  L  S  R  I  N  N  R  K  M  V  E  R  K  N  L  Y  L  M  G  L  L  P  I
CTTGTAGTAGTGGTCCTTCTATCTCGTATTAACAATCGAAAGATGGTCGAAAGAAAAAATCTCTATTTGATGGGGCTTCTTCCTATAC
 L  V  V  V  V  L  L  S  R  I  N  N  R  K  M  V  E  R  K  N  L  Y  L  M  G  L  L  P  I
CTTGTAGTAGTGGTCCTTCTATCTCGTATTAACAATCGAAAGATGGTCGAAAGAAAAAATCTCTATTTGATGGGGCTTCTTCCTATAC
 L  V  V  V  V  L  L  S  R  I  N  N  R  K  M  V  E  R  K  N  L  Y  L  M  G  L  L  P  I
CTTGTAGTAGTGGTCCTTCTATCTCGTATTAACAATCGAAAGATGGTCGAAAGAAAAAATCTCTATTTGATGGGGCTTCTTCCTATAC
 L  V  V  V  V  L  L  S  R  I  N  N  R  K  M  V  E  R  K  N  L  Y  L  M  G  L  L  P  I
CTTGTAGTAGTGGTCCTTCTATCTCGTATTAACAATCGAAAGATGGTCGAAAGAAAAAATCTCTATTTGATGGGGCTTCTTCCTATAC
 L  V  V  V  V  L  L  S  R  I  N  N  R  K  M  V  E  R  K  N  L  Y  L  M  G  L  L  P  I
CTTGTAGTAGTGGTCCTTCTATCTCGTATTAACAATCGAAAGATGGTCGAAAGAAAAAATCTCTATTTGATGGGGCTTCTTCCTATAC
 L  V  V  V  V  L  L  S  R  I  N  N  R  K  M  V  E  R  K  N  L  Y  L  M  G  L  L  P  I
CTTGTAGTAGTGGTCCTTCTATCTCGTATTAACAATCGAAAGATGGTCGAAAGAAAAAATCTCTATTTGATGGGGCTTCTTCCTATAC
 L  V  V  V  V  L  L  S  R  I  N  N  R  K  M  V  E  R  K  N  L  Y  L  M  G  L  L  P  I
CTTGTAGTAGTGGTCCTTCTATCTCGTATTAACAATCGAAAGATGGTCGAAAGAAAAAATCTCTATTTGATGGGGCTTCTTCCTATAC
 L  V  V  V  V  L  L  S  R  I  N  N  R  K  M  V  E  R  K  N  L  Y  L  M  G  L  L  P  I
CTTGTAGTAGTGGTCCTTCTATCTCGTATTAACAATCGAAAGATGGTCGAAAGAAAAAATCTCTATTTGATGGGGCTTCTTCCTATAC
 L  V  V  V  V  L  L  S  R  I  N  N  R  K  M  V  E  R  K  N  L  Y  L  M  G  L  L  P  I
CTTGTAGTAGTGGTCCTTCTATCTCGTATTAACAATCGAAAGATTGTCGAAAGAAAAAATCTCTATTTGATGGGGCTTCTTCCTATAC
 L  V  V  V  V  L  L  S  R  I  N  N  R  K  I  V  E  R  K  N  L  Y  L  M  G  L  L  P  I
CTTGTAGTAGTGGTCCTTCTATCTCGTATTAACAATCGAAAGATGGTCGAAAGAAAAAATCTCTATTTGATGGGGCTTCTTCCTATAC
 L  V  V  V  V  L  L  S  R  I  N  N  R  K  M  V  E  R  K  N  L  Y  L  M  G  L  L  P  I
CTTGTAGTAGTGGTCCTTCTATCTCGTATTAACAATCGAAAGATGGTCGAAAGAAAAAATCTCTATTTGATGGGGCTTCTTCCTATAC
 L  V  V  V  V  L  L  S  R  I  N  N  R  K  M  V  E  R  K  N  L  Y  L  M  G  L  L  P  I
CTTGTAGTAGTGGTCCTTCTATCTCGTATTAACAATCGAAAGATGGTCGAAAGAAAAAATCTCTATTTGATGGGGCTTCTTCCTATAC
 L  V  V  V  V  L  L  S  R  I  N  N  R  K  M  V  E  R  K  N  L  Y  L  M  G  L  L  P  I
CTTGTAGTAGTGGTCCTTCTATCTCGTATTAACAATCGAAAGATGGTCGAAAGAAAAAATCTCTATTTGATGGGGCTTCTTCCTATAC
 L  V  V  V  V  L  L  S  R  I  N  N  R  K  M  V  E  R  K  N  L  Y  L  M  G  L  L  P  I
CTTGTAGTAGTGGTCCTTCTATCTCGTATTAACAATCGAAAGATGGTCGAAAGAAAAAATCTCTATTTGATGGGGCTTCTTCCTATAC
 L  V  V  V  V  L  L  S  R  I  N  N  R  K  M  V  E  R  K  N  L  Y  L  M  G  L  L  P  I
CTTGTAGTAGTGGCCCTTCTATCTCGTATTAACAATCGAAAGATGGTCGAAAGAAAAAATCTCTATTTGATGGGGCTTCTTCCTATAC
 L  V  V  V  A  L  L  S  R  I  N  N  R  K  M  V  E  R  K  N  L  Y  L  M  G  L  L  P  I
CTTGTAGTAGTGGCCCTTCTATCTCGTATTAACAATCGAAAGATGGTCGAAAGAAAAAATCTCTATTTGATGGGGCTTCTTCCTATAC
 L  V  V  V  A  L  L  S  R  I  N  N  R  K  M  V  E  R  K  N  L  Y  L  M  G  L  L  P  I
CTTGTAGTAGTGGCCCTTCTATCTCGTATTAACAATCGAAAGATGGTCGAAAGAAAAAATCTCTATTTGATGGGGCTTCTTCCTATAC
 L  V  V  V  A  L  L  S  R  I  N  N  R  K  M  V  E  R  K  N  L  Y  L  M  G  L  L  P  I
CTTGTAGTAGTGGTCCTTCTATCTCGTATTAACAATCGAAAGATGGTCGAAAGAAAAAATCTCTATTTGATGGGGCTTCTTCCTATAC
 L  V  V  V  V  L  L  S  R  I  N  N  R  K  M  V  E  R  K  N  L  Y  L  M  G  L  L  P  I
CTTGTAGTAGTGGTCCTTCTATCTCGTATTAACAATCGAAAGATGGTCGAAAGAAAAAATCTCTATTTGATGGGGCTTCTTCCTATAC
 L  V  V  V  V  L  L  S  R  I  N  N  R  K  M  V  E  R  K  N  L  Y  L  M  G  L  L  P  I
```

```
340        350        360        370        380        390        400        410
CTATGAATTCCATTGGACCCAGAAAGGAGACATTGGAAGAATCTTTTTGGTCTTCCAATAGAAATAGGTTGATTGTTTC

CTATGAATTCCATTGGACCCAGAAAGGAGACATTGGAAGAATCTTTTTGGTCTTCCAATAGAAATAGGTTGATTGTTTC
P M N S I G P R K E T L E E S F W S S N R N R L I V S
CTATGAATTCCATTGGACCCAGAAAGGAGACATTGGAAGAATCTTTTTGGTCTTCCAATAGAAATAGGTTGATTGTTTC
P M N S I G P R K E T L E E S F W S S N R N R L I V S
CTATGAATTCCATTGGACCCAGAAAGGAGACATTGGAAGAATCTTTTTGGTCTTCCAATAGAAATAGGTTGATTGTTTC
P M N S I G P R K E T L E E S F W S S N R N R L I V S
CTATGAATTCCATTGGACCCAGAAAGGAGACATTGGAAGAATCTTTTTGGTCTTCCAATAGAAATAGGTTGATTGTTTC
P M N S I G P R K E T L E E S F W S S N R N R L I V S
CTATGAATTCCATTGGACCCAGAAAGGAGACATTGGAAGAATCTTTTTGGTCTTCCAATAGAAATAGGTTGATTGTTTC
P M N S I G P R K E T L E E S F W S S N R N R L I V S
CTATGAATTCCATTGGACCCAGAAAGGAGACATTGGAAGAATCTTTTTGGTCTTCCAATAGAAATAGGTTGATTGTTTC
P M N S I G P R K E T L E E S F W S S N R N R L I V S
CTATGAATTCCATTGGACCCAGAAAGGAGACATTGGAAGAATCTTTTTGGTCTTCCAATAGAAATAGGTTGATTGTTTC
P M N S I G P R K E T L E E S F W S S N R N R L I V S
CTATGAATTCCATTGGACCCAGAAAGGAGACATTGGAAGAATCTTTTTGGTCTTCCAATAGAAATAGGTTGATTGTTTC
P M N S I G P R K E T L E E S F W S S N R N R L I V S
CTATGAATTCCATTGGACCCAGAAAGGAGACATTGGAAGAATCTTTTTGGTCTTCCAATAGAAATAGGTTGATTGTTTC
P M N S I G P R K E T L E E S F W S S N R N R L I V S
CTATGAATTCCATTGGACCCAGAAAGGAGACATTGGAAGAATCTTTTTGGTCTTCCAATAGAAATAGGTTGATTGTTTC
P M N S I G P R K E T L E E S F W S S N R N R L I V S
CTATGAATTCCATTGGACCCAGAAAGGAGACATTGGAAGAATCTTTTTGGTCTTCCAATAGAAATAGGTTGATTGTTTC
P M N S I G P R K E T L E E S F W S S N R N R L I V S
CTATGAATTCCATTGGACCCAGAAAGGAGACATTGGAAGAATCTTTTTGGTCTTCCAATAGAAATAGGTTGATTGTTTC
P M N S I G P R K E T L E E S F W S S N R N R L I V S
CTATGAATTCCATTGGACCCAGAAAGGAGACATTGGAAGAATCTTTTTGGTCTTCCAATAGAAATAGGTTGATTGTTTC
P M N S I G P R K E T L E E S F W S S N R N R L I V S
CTATGAATTCCATTGGACCCAGAAAGGAGACATTGGAAGAATCTTTTTGGTCTTCCAATAGAAATAGGTTGATTGTTTC
P M N S I G P R K E T L E E S F W S S N R N R L I V S
CTATGAATTCCATTGGACCCAGAAAGGAGACATTGGAAGAATCTTTTTGGTCTTCCAATAGAAATAGGTTGATTGTTTC
P M N S I G P R K E T L E E S F W S S N R N R L I V S
CTATGAATTCCATTGGACCCAGAAAGGAGACATTGGAAGAATCTTTTTGGTCTTCCAATAGAAATAGGTTGATTGTTTC
P M N S I G P R K E T L E E S F W S S N R N R L I V S
CTATGAATTCCATTGGACCCAGAAAGGAGACATTGGAAGAATCTTTTTGGTCTTCCAATAGAAATAGGTTGATTGTTTC
P M N S I G P R K E T L E E S F W S S N R N R L I V S
CTATGAATTCCATTGGACCCAGAAAGGAGACATTGGAAGAATCTTTTTGGTCTTCCAATAGAAATAGGTTGATTGTTTC
P M N S I G P R K E T L E E S F W S S N R N R L I V S
CTATGAATTCCATTGGACCCAGAAAGGAGACATTGGAAGAATCTTTTTGGTCTTCCAATAGAAATAGGTTGATTGTTTC
P M N S I G P R K E T L E E S F W S S N R N R L I V S
CTATGAATTCCATTGGACCCAGAAAGGAGACATTGGAAGAATCTTTTTGGTCTTCCAATAGAAATAGGTTGATTGTTTC
P M N S I G P R K E T L E E S F W S S N R N R L I V S
CTATGAATTCCATTGGACCCAGAAAGGAGACATTGGAAGAATCTTTTTGGTCTTCCAATAGAAATAGGTTGATTGTTTC
P M N S I G P R K E T L E E S F W S S N R N R L I V S
CTATGAATTCCATTGGACCCAGAAAGGAGACATTGGAAGAATCTTTTTGGTCTTCCAATAGAAATAGGTTGATTGTTTC
P M N S I G P R K E T L E E S F W S S N R N R L I V S
CTATGAATTCCATTGGACCCAGAAAGGAGACATTGGAAGAATCTTTTTGGTCTTCCAATAGAAATAGGTTGATTGTTTC
P M N S I G P R K E T L E E S F W S S N R N R L I V S
```

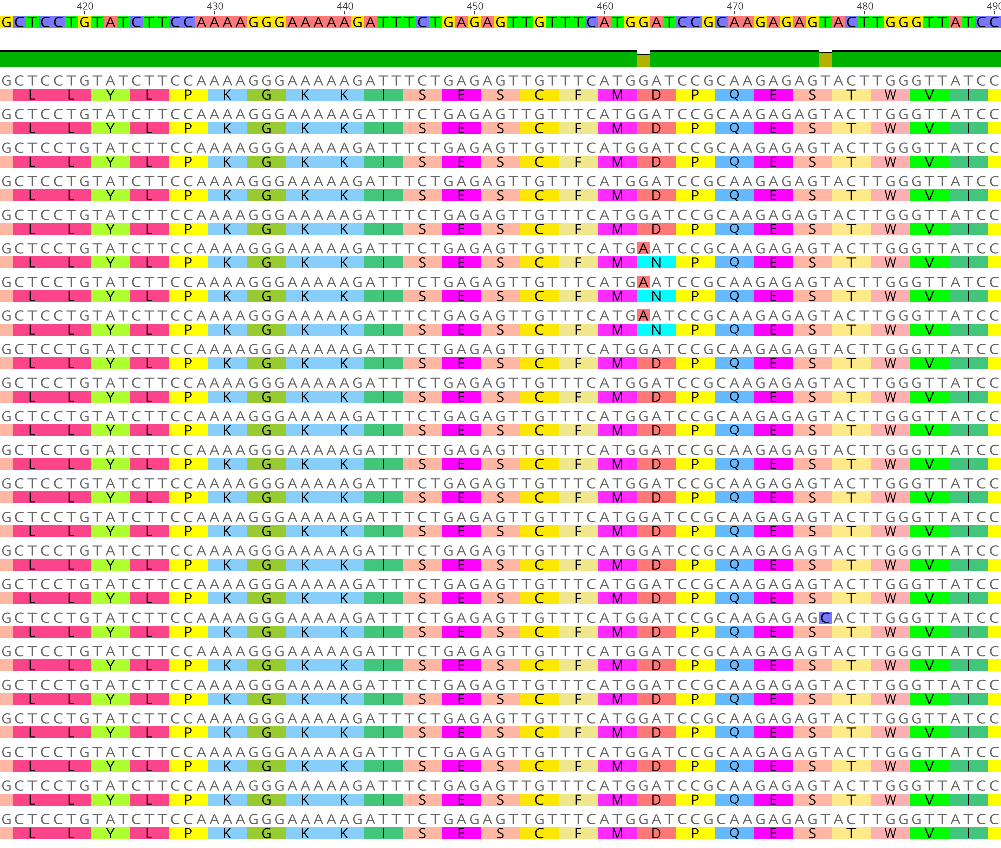
420 430 440 450 460 470 480 490
GCTCCTGTATCTTCCAAAAGGGAAAAAGATTTCTGAGAGTTGTTTCATGGATCCGCAAGAGAGTACTTGGGTTATCC
GCTCCTGTATCTTCCAAAAGGGAAAAAGATTTCTGAGAGTTGTTTCATGGATCCGCAAGAGAGTACTTGGGTTATCC
L L Y L P K G K K I S E S C F M D P Q E S T W V I
GCTCCTGTATCTTCCAAAAGGGAAAAAGATTTCTGAGAGTTGTTTCATGGATCCGCAAGAGAGTACTTGGGTTATCC
L L Y L P K G K K I S E S C F M D P Q E S T W V I
GCTCCTGTATCTTCCAAAAGGGAAAAAGATTTCTGAGAGTTGTTTCATGGATCCGCAAGAGAGTACTTGGGTTATCC
L L Y L P K G K K I S E S C F M D P Q E S T W V I
GCTCCTGTATCTTCCAAAAGGGAAAAAGATTTCTGAGAGTTGTTTCATGGATCCGCAAGAGAGTACTTGGGTTATCC
L L Y L P K G K K I S E S C F M D P Q E S T W V I
GCTCCTGTATCTTCCAAAAGGGAAAAAGATTTCTGAGAGTTGTTTCATGGATCCGCAAGAGAGTACTTGGGTTATCC
L L Y L P K G K K I S E S C F M D P Q E S T W V I
GCTCCTGTATCTTCCAAAAGGGAAAAAGATTTCTGAGAGTTGTTTCATGAATCCGCAAGAGAGTACTTGGGTTATCC
L L Y L P K G K K I S E S C F M N P Q E S T W V I
GCTCCTGTATCTTCCAAAAGGGAAAAAGATTTCTGAGAGTTGTTTCATGAATCCGCAAGAGAGTACTTGGGTTATCC
L L Y L P K G K K I S E S C F M N P Q E S T W V I
GCTCCTGTATCTTCCAAAAGGGAAAAAGATTTCTGAGAGTTGTTTCATGAATCCGCAAGAGAGTACTTGGGTTATCC
L L Y L P K G K K I S E S C F M N P Q E S T W V I
GCTCCTGTATCTTCCAAAAGGGAAAAAGATTTCTGAGAGTTGTTTCATGGATCCGCAAGAGAGTACTTGGGTTATCC
L L Y L P K G K K I S E S C F M D P Q E S T W V I
GCTCCTGTATCTTCCAAAAGGGAAAAAGATTTCTGAGAGTTGTTTCATGGATCCGCAAGAGAGTACTTGGGTTATCC
L L Y L P K G K K I S E S C F M D P Q E S T W V I
GCTCCTGTATCTTCCAAAAGGGAAAAAGATTTCTGAGAGTTGTTTCATGGATCCGCAAGAGAGTACTTGGGTTATCC
L L Y L P K G K K I S E S C F M D P Q E S T W V I
GCTCCTGTATCTTCCAAAAGGGAAAAAGATTTCTGAGAGTTGTTTCATGGATCCGCAAGAGAGTACTTGGGTTATCC
L L Y L P K G K K I S E S C F M D P Q E S T W V I
GCTCCTGTATCTTCCAAAAGGGAAAAAGATTTCTGAGAGTTGTTTCATGGATCCGCAAGAGAGTACTTGGGTTATCC
L L Y L P K G K K I S E S C F M D P Q E S T W V I
GCTCCTGTATCTTCCAAAAGGGAAAAAGATTTCTGAGAGTTGTTTCATGGATCCGCAAGAGAGTACTTGGGTTATCC
L L Y L P K G K K I S E S C F M D P Q E S T W V I
GCTCCTGTATCTTCCAAAAGGGAAAAAGATTTCTGAGAGTTGTTTCATGGATCCGCAAGAGAGTACTTGGGTTATCC
L L Y L P K G K K I S E S C F M D P Q E S T W V I
GCTCCTGTATCTTCCAAAAGGGAAAAAGATTTCTGAGAGTTGTTTCATGGATCCGCAAGAGAGTACTTGGGTTATCC
L L Y L P K G K K I S E S C F M D P Q E S T W V I
GCTCCTGTATCTTCCAAAAGGGAAAAAGATTTCTGAGAGTTGTTTCATGGATCCGCAAGAGAGCACTTGGGTTATCC
L L Y L P K G K K I S E S C F M D P Q E S T W V I
GCTCCTGTATCTTCCAAAAGGGAAAAAGATTTCTGAGAGTTGTTTCATGGATCCGCAAGAGAGTACTTGGGTTATCC
L L Y L P K G K K I S E S C F M D P Q E S T W V I
GCTCCTGTATCTTCCAAAAGGGAAAAAGATTTCTGAGAGTTGTTTCATGGATCCGCAAGAGAGTACTTGGGTTATCC
L L Y L P K G K K I S E S C F M D P Q E S T W V I
GCTCCTGTATCTTCCAAAAGGGAAAAAGATTTCTGAGAGTTGTTTCATGGATCCGCAAGAGAGTACTTGGGTTATCC
L L Y L P K G K K I S E S C F M D P Q E S T W V I
GCTCCTGTATCTTCCAAAAGGGAAAAAGATTTCTGAGAGTTGTTTCATGGATCCGCAAGAGAGTACTTGGGTTATCC
L L Y L P K G K K I S E S C F M D P Q E S T W V I
GCTCCTGTATCTTCCAAAAGGGAAAAAGATTTCTGAGAGTTGTTTCATGGATCCGCAAGAGAGTACTTGGGTTATCC
L L Y L P K G K K I S E S C F M D P Q E S T W V I
GCTCCTGTATCTTCCAAAAGGGAAAAAGATTTCTGAGAGTTGTTTCATGGATCCGCAAGAGAGTACTTGGGTTATCC
L L Y L P K G K K I S E S C F M D P Q E S T W V I

```
        500       510       520       530       540       550       560
CAATAAAGAAAAAGCGTATCATGCCTGAATCTAACCGGGGTTCGCGGTGGTGGAGGAACCGGATCGGAAAAAGAGG

CAATAAAGAAAAAGCGTATCATGCCTGAATCTAACCGGGGTTCGCGGTGGTGGAGGAACCGGATCGGAAAAAGAGG
P  I  K  K  K  R  I  M  P  E  S  N  R  G  S  R  W  W  R  N  R  I  G  K  K  R
CAATAAAGAAAAAGCGTATCATGCCTGAATCTAACCGGGGTTCGCGGTGGTGGAGGAACCGGATCGGAAAAAGAGG
P  I  K  K  K  R  I  M  P  E  S  N  R  G  S  R  W  W  R  N  R  I  G  K  K  R
CAATAAAGAAAAAGCGTATCATGCCTGAATCTAACCGGGGTTCGCGGTGGTGGAGGAACCGGATCGGAAAAAGAGG
P  I  K  K  K  R  I  M  P  E  S  N  R  G  S  R  W  W  R  N  R  I  G  K  K  R
CAATAAAGAAAAAGCGTATCATGCCTGAATCTAACCGGGGTTCGCGGTGGTGGAGGAACCGGATCGGAAAAAGAGG
P  I  K  K  K  R  I  M  P  E  S  N  R  G  S  R  W  W  R  N  R  I  G  K  K  R
CAATAAAGAAAAAGCGTATCATGCCTGAATCTAACCGGGGTTCGCGGTGGTGGAGGAACCGGATCGGAAAAAGAGG
P  I  K  K  K  R  I  M  P  E  S  N  R  G  S  R  W  W  R  N  R  I  G  K  K  R
CAATAAAGAAAAAGCGTATCATGCCTGAATCTAACCGGGGTTCGCGGTGGTGGAGGAACCGGATCGGAAAAAGAGG
P  I  K  K  K  R  I  M  P  E  S  N  R  G  S  R  W  W  R  N  R  I  G  K  K  R
CAATAAAGAAAAAGCGTATCATGCCTGAATCTAACCGGGGTTCGCGGTGGTGGAGGAACCGGATCGGAAAAAGAGG
P  I  K  K  K  R  I  M  P  E  S  N  R  G  S  R  W  W  R  N  R  I  G  K  K  R
CAATAAAGAAAAAGCGTATCATGCCTGAATCTAACCGGGGTTCGCGGTGGTGGAGGAACCGGATCGGAAAAAGAGG
P  I  K  K  K  R  I  M  P  E  S  N  R  G  S  R  W  W  R  N  R  I  G  K  K  R
CAATAAAGAAAAAGCGTATCATGCCTGAATCTAACCGGGGTTCGCGGTGGTGGAGGAACCGGATCGGAAAAAGAGG
P  I  K  K  K  R  I  M  P  E  S  N  R  G  S  R  W  W  R  N  R  I  G  K  K  R
CAATAAAGAAAAAGCGTATCATGCCTGAATCTAACCGGGGTTCGCGGTGGTGGAGGAACCGGATCGGAAAAAGAGG
P  I  K  K  K  R  I  M  P  E  S  N  R  G  S  R  W  W  R  N  R  I  G  K  K  R
CAATAAAGAAAAAGCGTATCATGCCTGAATCTAACCGGGGTTCGCGGTGGTGGAGGAACCGGATCGGAAAAAGAGG
P  I  K  K  K  R  I  M  P  E  S  N  R  G  S  R  W  W  R  N  R  I  G  K  K  R
CAATAAAGAAAAAGCGTATCATGCCTGAATCTAACCGGGGTTCGCGGTGGTGGAGGAACCGGATCGGAAAAAGAGG
P  I  K  K  K  R  I  M  P  E  S  N  R  G  S  R  W  W  R  N  R  I  G  K  K  R
CAATAAAGAAAAAGCGTATCATGCCTGAATCTAACCGGGGTTCGCGGTGGTGGAGGAACCGGATCGGAAAAAGAGG
P  I  K  K  K  R  I  M  P  E  S  N  R  G  S  R  W  W  R  N  R  I  G  K  K  R
CAATAAATAAAAAGCGTATCATGCCTGAATCTAACCGGGGTTCGCGGTGGTGGAGGAACCGGATCGGAAAAAGAGG
P  I  N  K  K  R  I  M  P  E  S  N  R  G  S  R  W  W  R  N  R  I  G  K  K  R
CAATAAAGAAAAAGCGTATCATGCCTGAATCTAACCGGGGTTCGCGGTGGTGGAGGAACCGGATCGGAAAAAGAGG
P  I  K  K  K  R  I  M  P  E  S  N  R  G  S  R  W  W  R  N  R  I  G  K  K  R
CAATAAAGAAAAAGCGTATCATGCCTGAATCTAACCGGGGTTCGCGGTGGTGGAGGAACCGGATCGGAAAAAGAGG
P  I  K  K  K  R  I  M  P  E  S  N  R  G  S  R  W  W  R  N  R  I  G  K  K  R
CAATAAAGAAAAAGCGTATCATGCCTGAATCTAACCGGGGTTCGCGGTGGTGGAGGAACCGGATCGGAAAAAGAGG
P  I  K  K  K  R  I  M  P  E  S  N  R  G  S  R  W  W  R  N  R  I  G  K  K  R
CAATAAAGAAAAAGCGTATCATGCCTGAATCTAACCGGGGTTCGCGGTGGTGGAGGAACCGGATCGGAAAAAGAGG
P  I  K  K  K  R  I  M  P  E  S  N  R  G  S  R  W  W  R  N  R  I  G  K  K  R
CAATAAAGAAAAAGCGTATCATGCCTGAATCTAACCGGGGTTCGCGGTGGTGGAGGAACCGGATCGGAAAAAGAGG
P  I  K  K  K  R  I  M  P  E  S  N  R  G  S  R  W  W  R  N  R  I  G  K  K  R
CAATAAAGAAAAAGCGTATCATGCCTGAATCTAACCGGGGTTCGCGGTGGTGGAGGAACCGGATCGGAAAAAGAGG
P  I  K  K  K  R  I  M  P  E  S  N  R  G  S  R  W  W  R  N  R  I  G  K  K  R
CAATAAAGAAAAAGCGTATCATGCCTGAATCTAACCGGGGTTCGCGGTGGTGGAGGAACCGGATCGGAAAAAGAGG
P  I  K  K  K  R  I  M  P  E  S  N  R  G  S  R  W  W  R  N  R  I  G  K  K  R
CAATAAAGAAAAAGCGTATCATGCCTGAATCTAACCGGGGTTCGCGGTGGTGGAGGAACCGGATCGGAAAAAGAGG
P  I  K  K  K  R  I  M  P  E  S  N  R  G  S  R  W  W  R  N  R  I  G  K  K  R
CAATAAAGAAAAAGCGTATCATGCCTGAATCTAACCGGGGTTCGCGGTGGTGGAGGAACCGGATCGGAAAAAGAGG
P  I  K  K  K  R  I  M  P  E  S  N  R  G  S  R  W  W  R  N  R  I  G  K  K  R
CAATAAAGAAAAAGCGTATCATGCCTGAATCTAACCGGGGTTCGCGGTGGTGGAGGAACCGGATCGGAAAAAGAGG
P  I  K  K  K  R  I  M  P  E  S  N  R  G  S  R  W  W  R  N  R  I  G  K  K  R
```

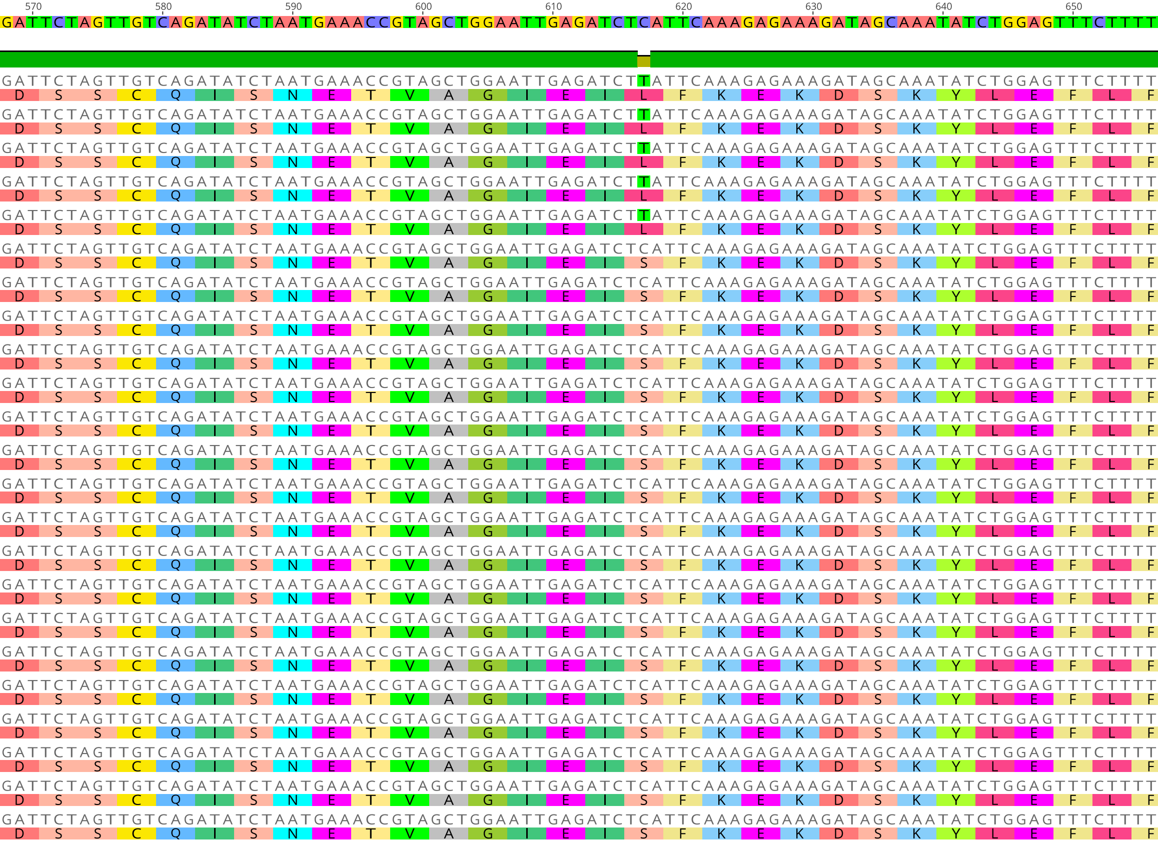
570 580 590 600 610 620 630 640 650
GATTCTAGTTGTCAGATATCTAATGAAACCGTAGCTGGAATTGAGATCTCATTCAAAGAGAAAGATAGCAAATATCTGGAGTTTCTTTT
GATTCTAGTTGTCAGATATCTAATGAAACCGTAGCTGGAATTGAGATCTTATTCAAAGAGAAAGATAGCAAATATCTGGAGTTTCTTTT
D S S C Q I S N E T V A G I E I L F K E K D S K Y L E F L F
GATTCTAGTTGTCAGATATCTAATGAAACCGTAGCTGGAATTGAGATCTTATTCAAAGAGAAAGATAGCAAATATCTGGAGTTTCTTTT
D S S C Q I S N E T V A G I E I L F K E K D S K Y L E F L F
GATTCTAGTTGTCAGATATCTAATGAAACCGTAGCTGGAATTGAGATCTTATTCAAAGAGAAAGATAGCAAATATCTGGAGTTTCTTTT
D S S C Q I S N E T V A G I E I L F K E K D S K Y L E F L F
GATTCTAGTTGTCAGATATCTAATGAAACCGTAGCTGGAATTGAGATCTTATTCAAAGAGAAAGATAGCAAATATCTGGAGTTTCTTTT
D S S C Q I S N E T V A G I E I L F K E K D S K Y L E F L F
GATTCTAGTTGTCAGATATCTAATGAAACCGTAGCTGGAATTGAGATCTTATTCAAAGAGAAAGATAGCAAATATCTGGAGTTTCTTTT
D S S C Q I S N E T V A G I E I L F K E K D S K Y L E F L F
GATTCTAGTTGTCAGATATCTAATGAAACCGTAGCTGGAATTGAGATCTCATTCAAAGAGAAAGATAGCAAATATCTGGAGTTTCTTTT
D S S C Q I S N E T V A G I E I S F K E K D S K Y L E F L F
GATTCTAGTTGTCAGATATCTAATGAAACCGTAGCTGGAATTGAGATCTCATTCAAAGAGAAAGATAGCAAATATCTGGAGTTTCTTTT
D S S C Q I S N E T V A G I E I S F K E K D S K Y L E F L F
GATTCTAGTTGTCAGATATCTAATGAAACCGTAGCTGGAATTGAGATCTCATTCAAAGAGAAAGATAGCAAATATCTGGAGTTTCTTTT
D S S C Q I S N E T V A G I E I S F K E K D S K Y L E F L F
GATTCTAGTTGTCAGATATCTAATGAAACCGTAGCTGGAATTGAGATCTCATTCAAAGAGAAAGATAGCAAATATCTGGAGTTTCTTTT
D S S C Q I S N E T V A G I E I S F K E K D S K Y L E F L F
GATTCTAGTTGTCAGATATCTAATGAAACCGTAGCTGGAATTGAGATCTCATTCAAAGAGAAAGATAGCAAATATCTGGAGTTTCTTTT
D S S C Q I S N E T V A G I E I S F K E K D S K Y L E F L F
GATTCTAGTTGTCAGATATCTAATGAAACCGTAGCTGGAATTGAGATCTCATTCAAAGAGAAAGATAGCAAATATCTGGAGTTTCTTTT
D S S C Q I S N E T V A G I E I S F K E K D S K Y L E F L F
GATTCTAGTTGTCAGATATCTAATGAAACCGTAGCTGGAATTGAGATCTCATTCAAAGAGAAAGATAGCAAATATCTGGAGTTTCTTTT
D S S C Q I S N E T V A G I E I S F K E K D S K Y L E F L F
GATTCTAGTTGTCAGATATCTAATGAAACCGTAGCTGGAATTGAGATCTCATTCAAAGAGAAAGATAGCAAATATCTGGAGTTTCTTTT
D S S C Q I S N E T V A G I E I S F K E K D S K Y L E F L F
GATTCTAGTTGTCAGATATCTAATGAAACCGTAGCTGGAATTGAGATCTCATTCAAAGAGAAAGATAGCAAATATCTGGAGTTTCTTTT
D S S C Q I S N E T V A G I E I S F K E K D S K Y L E F L F
GATTCTAGTTGTCAGATATCTAATGAAACCGTAGCTGGAATTGAGATCTCATTCAAAGAGAAAGATAGCAAATATCTGGAGTTTCTTTT
D S S C Q I S N E T V A G I E I S F K E K D S K Y L E F L F
GATTCTAGTTGTCAGATATCTAATGAAACCGTAGCTGGAATTGAGATCTCATTCAAAGAGAAAGATAGCAAATATCTGGAGTTTCTTTT
D S S C Q I S N E T V A G I E I S F K E K D S K Y L E F L F
GATTCTAGTTGTCAGATATCTAATGAAACCGTAGCTGGAATTGAGATCTCATTCAAAGAGAAAGATAGCAAATATCTGGAGTTTCTTTT
D S S C Q I S N E T V A G I E I S F K E K D S K Y L E F L F
GATTCTAGTTGTCAGATATCTAATGAAACCGTAGCTGGAATTGAGATCTCATTCAAAGAGAAAGATAGCAAATATCTGGAGTTTCTTTT
D S S C Q I S N E T V A G I E I S F K E K D S K Y L E F L F
GATTCTAGTTGTCAGATATCTAATGAAACCGTAGCTGGAATTGAGATCTCATTCAAAGAGAAAGATAGCAAATATCTGGAGTTTCTTTT
D S S C Q I S N E T V A G I E I S F K E K D S K Y L E F L F
GATTCTAGTTGTCAGATATCTAATGAAACCGTAGCTGGAATTGAGATCTCATTCAAAGAGAAAGATAGCAAATATCTGGAGTTTCTTTT
D S S C Q I S N E T V A G I E I S F K E K D S K Y L E F L F
GATTCTAGTTGTCAGATATCTAATGAAACCGTAGCTGGAATTGAGATCTCATTCAAAGAGAAAGATAGCAAATATCTGGAGTTTCTTTT
D S S C Q I S N E T V A G I E I S F K E K D S K Y L E F L F
GATTCTAGTTGTCAGATATCTAATGAAACCGTAGCTGGAATTGAGATCTCATTCAAAGAGAAAGATAGCAAATATCTGGAGTTTCTTTT
D S S C Q I S N E T V A G I E I S F K E K D S K Y L E F L F
GATTCTAGTTGTCAGATATCTAATGAAACCGTAGCTGGAATTGAGATCTCATTCAAAGAGAAAGATAGCAAATATCTGGAGTTTCTTTT
D S S C Q I S N E T V A G I E I S F K E K D S K Y L E F L F

```
660       670       680       690       700       710       720       730
TTTATCCTATACGGATGATCCGATCCGCAAGGACCATGATTGGGAATTGTTTGATCGTCTTTCTCCGAGGAAGAAAC

TTTATCCTATACGGATGATCCGATCCGCAAGGACCATGATTGGGAATTGTTTGATCGTCTTTCTCCGAGGAAGAAAC
 L  S  Y  T  D  D  P  I  R  K  D  H  D  W  E  L  F  D  R  L  S  P  R  K  K
TTTATCCTATACGGATGATCCGATCCGCAAGGACCATGATTGGGAATTGTTTGATCGTCTTTCTCCGAGGAAGAAAC
 L  S  Y  T  D  D  P  I  R  K  D  H  D  W  E  L  F  D  R  L  S  P  R  K  K
TTTATCCTATACGGATGATCCGATCCGCAAGGACCATGATTGGGAATTGTTTGATCGTCTTTCTCCGAGGAAGAAAC
 L  S  Y  T  D  D  P  I  R  K  D  H  D  W  E  L  F  D  R  L  S  P  R  K  K
TTTATCCTATACGGATGATCCGATCCGCAAGGACCATGATTGGGAATTGTTTGATCGTCTTTCTCCGAGGAAGAAAC
 L  S  Y  T  D  D  P  I  R  K  D  H  D  W  E  L  F  D  R  L  S  P  R  K  K
TTTATCCTATACGGATGATCCGATCCGCAAGGACCATGATTGGGAATTGTTTGATCGTCTTTCTCCGAGGAAGAAAC
 L  S  Y  T  D  D  P  I  R  K  D  H  D  W  E  L  F  D  R  L  S  P  R  K  K
TTTATCCTATACGGATGATCCGATCCGCAAGGACCATGATTGGGAATTGTTTGATCGTCTTTCTCCGAGGAAGAAAC
 L  S  Y  T  D  D  P  I  R  K  D  H  D  W  E  L  F  D  R  L  S  P  R  K  K
TTTATCCTATACGGATGATCCGATCCGCAAGGACCATGATTGGGAATTGTTTGATCGTCTTTCTCCGAGGAAGAAAC
 L  S  Y  T  D  D  P  I  R  K  D  H  D  W  E  L  F  D  R  L  S  P  R  K  K
TTTATCCTATACGGATGATCCGATCCGCAAGGACCATGATTGGGAATTGTTTGATCGTCTTTCTCCGAGGAAGAAAC
 L  S  Y  T  D  D  P  I  R  K  D  H  D  W  E  L  F  D  R  L  S  P  R  K  K
TTTATCCTATACGGATGATCCGATCCGCAAGGACCATGATTGGGAATTGTTTGATCGTCTTTCTCCGAGGAAGAAAC
 L  S  Y  T  D  D  P  I  R  K  D  H  D  W  E  L  F  D  R  L  S  P  R  K  K
TTTATCCTATACGGATGATCCGATCCGCAAGGACCATGATTGGGAATTGTTTGATCGTCTTTCTCCGAGGAAGAAAC
 L  S  Y  T  D  D  P  I  R  K  D  H  D  W  E  L  F  D  R  L  S  P  R  K  K
TTTATCCTATACGGATGATCCGATCCGCAAGGACCATGATTGGGAATTGTTTGATCGTCTTTCTCCGAGGAAGAAAC
 L  S  Y  T  D  D  P  I  R  K  D  H  D  W  E  L  F  D  R  L  S  P  R  K  K
TTTATCCTATACGGATGATCCGATCCGCAAGGACCATGATTGGGAATTGTTTGATCGTCTTTCTCCGAGGAAGAAAC
 L  S  Y  T  D  D  P  I  R  K  D  H  D  W  E  L  F  D  R  L  S  P  R  K  K
TTTATCCTATACGGATGATCCGATCCGCAAGGACCATGATTGGGAATTGTTTGATCGTCTTTCTCCGAGGAAGAAAC
 L  S  Y  T  D  D  P  I  R  K  D  H  D  W  E  L  F  D  R  L  S  P  R  K  K
TTTATCCTATACGGATGATCCGATCCGCAAGGACCATGATTGGGAATTGTTTGATCGTCTTTCTCCGAGGAAGAAAC
 L  S  Y  T  D  D  P  I  R  K  D  H  D  W  E  L  F  D  R  L  S  P  R  K  K
TTTATCCTATACGGATGATCCGATCCGCAAGGACCATGATTGGGAATTGTTTGATCGTCTTTCTCCGAGGAATAAAC
 L  S  Y  T  D  D  P  I  R  K  D  H  D  W  E  L  F  D  R  L  S  P  R  N  K
TTTATCCTATACGGATGATCCGATCCGCAAGGACCATGATTGGGAATTGTTTGATCGTCTTTCTCCGAGGAAGAAAC
 L  S  Y  T  D  D  P  I  R  K  D  H  D  W  E  L  F  D  R  L  S  P  R  K  K
TTTATCCTATACGGATGATCCGATCCGCAAGGACCATGATTGGGAATTGTTTGATCGTCTTTCTCCGAGGAAGAAAC
 L  S  Y  T  D  D  P  I  R  K  D  H  D  W  E  L  F  D  R  L  S  P  R  K  K
TTTATCCTATACGGATGATCCGATCCGCAAGGACCATGATTGGGAATTGTTTGATCGTCTTTCTCCGAGGAAGAAAC
 L  S  Y  T  D  D  P  I  R  K  D  H  D  W  E  L  F  D  R  L  S  P  R  K  K
TTTATCCTATACGGATGATCCGATCCGCAAGGACCATGATTGGGAATTGTTTGATCGTCTTTCTCCGAGGAAGAAAC
 L  S  Y  T  D  D  P  I  R  K  D  H  D  W  E  L  F  D  R  L  S  P  R  K  K
TTTATCCTATACGGATGATCCGATCCGCAAGGACCATGATTGGGAATTGTTTGATCGTCTTTCTCCGAGGAAGAAAC
 L  S  Y  T  D  D  P  I  R  K  D  H  D  W  E  L  F  D  R  L  S  P  R  K  K
TTTATCCTATACGGATGATCCGATCCGCAAGGACCATGATTGGGAATTGTTTGATCGTCTTTCTCCGAGGAAGAAAC
 L  S  Y  T  D  D  P  I  R  K  D  H  D  W  E  L  F  D  R  L  S  P  R  K  K
TTTATCCTATACGGATGATCCGATCCGCAAGGACCATGATTGGGAATTGTTTGATCGTCTTTCTCCGAGGAAGAAAC
 L  S  Y  T  D  D  P  I  R  K  D  H  D  W  E  L  F  D  R  L  S  P  R  K  K
TTTATCCTATACGGATGATCCGATCCGCAAGGACCATGATTGGGAATTGTTTGATCGTCTTTCTCCGAGGAAGAAAC
 L  S  Y  T  D  D  P  I  R  K  D  H  D  W  E  L  F  D  R  L  S  P  R  K  K
TTTATCCTATACGGATGATCCGATCCGCAAGGACCATGATTGGGAATTGTTTGATCGTCTTTCTCCGAGGAAGAAAC
 L  S  Y  T  D  D  P  I  R  K  D  H  D  W  E  L  F  D  R  L  S  P  R  K  K
TTTATCCTATACGGATGATCCGATCCGCAAGGACCATGATTGGGAATTGTTTGATCGTCTTTCTCCGAGGAAGAAAC
 L  S  Y  T  D  D  P  I  R  K  D  H  D  W  E  L  F  D  R  L  S  P  R  K  K
```

```
740 750 760 770 780 790 800 810
GAAACATAATCAACTTGAATTCGGGACAGCTATTCGAAATCTTAGGGAAAGACTTGATTTGTTATCTCATGTCTGCTTT

GAAACATAATCAACTTGAATTCGGGACAGCTATTCGAAATCTTAGGGAAAGACTTGATTTGTTATCTCATGTCTGCTTT
R N I I N L N S G Q L F E I L G K D L I C Y L M S A F
GAAACATAATCAACTTGAATTCGGGACAGCTATTCGAAATCTTAGGGAAAGACTTGATTTGTTATCTCATGTCTGCTTT
R N I I N L N S G Q L F E I L G K D L I C Y L M S A F
GAAACATAATCAACTTGAATTCGGGACAGCTATTCGAAATCTTAGGGAAAGACTTGATTTGTTATCTCATGTCTGCTTT
R N I I N L N S G Q L F E I L G K D L I C Y L M S A F
GAAACATAATCAACTTGAATTCGGGACAGCTATTCGAAATCTTAGGGAAAGACTTGATTTGTTATCTCATGTCTGCTTT
R N I I N L N S G Q L F E I L G K D L I C Y L M S A F
GAAACATAATCAACTTGAATTCGGGACAGCTATTCGAAATCTTAGGGAAAGACTTGATTTGTTATCTCATGTCTGCTTT
R N I I N L N S G Q L F E I L G K D L I C Y L M S A F
GAAACATAATCAACTTGAATTCGGGACAGCTATTCGAAATCTTAGGGAAAGACTTGATTTGTTATCTCATGTCTGCTTT
R N I I N L N S G Q L F E I L G K D L I C Y L M S A F
GAAACATAATCAACTTGAATTCGGGACAGCTATTCGAAATCTTAGGGAAAGACTTGATTTGTTATCTCATGTCTGCTTT
R N I I N L N S G Q L F E I L G K D L I C Y L M S A F
GAAACATAATCAACTTGAATTCGGGACAGCTATTCGAAATCTTAGGGAAAGACTTGATTTGTTATCTCATGTCTGCTTT
R N I I N L N S G Q L F E I L G K D L I C Y L M S A F
GAAACATAATCAACTTGAATTCGGGACAGCTATTCGAAATCTTAGGGAAAGACTTGATTTGTTATCTCATGTCTGCTTT
R N I I N L N S G Q L F E I L G K D L I C Y L M S A F
GAAACATAATCAACTTGAATTCGGGACAGCTATTCGAAATCTTAGGGAAAGACTTGATTTGTTATCTCATGTCTGCTTT
R N I I N L N S G Q L F E I L G K D L I C Y L M S A F
GAAACATAATCAACTTGAATTCGGGACAGCTATTCGAAATCTTAGGGAAAGACTTGATTTGTTATCTCATGTCTGCTTT
R N I I N L N S G Q L F E I L G K D L I C Y L M S A F
GAAACATAATCAACTTGAATTCGGGACAGCTATTCGAAATCTTAGGGAAAGACTTGATTTGTTATCTCATGTCTGCTTT
R N I I N L N S G Q L F E I L G K D L I C Y L M S A F
GAAACATAATCAACTTGAATTCGGGACAGCTATTCGAAATCTTAGGGAAAGACTTGATTTGTTATCTCATGTCTGCTTT
R N I I N L N S G Q L F E I L G K D L I C Y L M S A F
GAAACATAATCAACTTGAATTCGGGACAGCTATTCGAAATCTTAGGGAAAGACTTGATTTGTTATCTCATGTCTGCTTT
R N I I N L N S G Q L F E I L G K D L I C Y L M S A F
GAAACATAATCAACTTGAATTCGGGACAGCTATTCGAAATCTTAGGGAAAGACTTGATTTGTTATCTCATGTCTGCTTT
R N I I N L N S G Q L F E I L G K D L I C Y L M S A F
GAAACATAATCAACTTGAATTCGGGACAGCTATTCGAAATCTTAGGGAAAGACTTGATTTGTTATCTCATGTCTGCTTT
R N I I N L N S G Q L F E I L G K D L I C Y L M S A F
GAAACATAATCAACTTGAATTCGGGACAGCTATTCGAAATCTTAGGGAAAGACTTGATTTGTTATCTCATGTCTGCTTT
R N I I N L N S G Q L F E I L G K D L I C Y L M S A F
GAAACATAATCAACTTGAATTCGGGACAGCTATTCGAAATCTTAGGGAAAGACTTGATTTGTTATCTCATGTCTGCTTT
R N I I N L N S G Q L F E I L G K D L I C Y L M S A F
GAAACATAATCAACTTGAATTCGGGACAGCTATTCGAAATCTTAGGGAAAGACTTGATTTGTTATCTCATGTCTGCTTT
R N I I N L N S G Q L F E I L G K D L I C Y L M S A F
GAAACATAATCAACTTGAATTCGGGACAGCTATTCGAAATCTTAGGGAAAGACTTGATTTGTTATCTCATGTCTGCTTT
R N I I N L N S G Q L F E I L G K D L I C Y L M S A F
GAAACATAATCAACTTGAATTCGGGACAGCTATTCGAAATCTTAGGGAAAGACTTGATTTGTTATCTCATGTCTGCTTT
R N I I N L N S G Q L F E I L G K D L I C Y L M S A F
GAAACATAATCAACTTGAATTCGGGACAGCTATTCGAAATCTTAGGGAAAGACTTGATTTGTTATCTCATGTCTGCTTT
R N I I N L N S G Q L F E I L G K D L I C Y L M S A F
GAAACATAATCAACTTGAATTCGGGACAGCTATTCGAAATCTTAGGGAAAGACTTGATTTGTTATCTCATGTCTGCTTT
R N I I N L N S G Q L F E I L G K D L I C Y L M S A F
```

820 830 840 850 860 870 880 890

TCGTGAAAAAGACCAATTCAGGGGGAGAGTTTCTTCAAACAACAAGGAGCTGGGCAACTATGCAATCCAATGATATTGAG

TCGTGAAAAAGACCAATTCAGGGGGAGAGTTTCTTCAAACAACAAGGAGCTGGGCAACTATGCAATCCAATGATATTGAG
R E K R P I Q G E S F F K Q Q G A G A T M Q S N D I E

TCGTGAAAAAGACCAATTCAGGGGGAGAGTTTCTTCAAACAACAAGGAGCTGGGCAACTATGCAATCCAATGATATTGAG
R E K R P I Q G E S F F K Q Q G A G A T M Q S N D I E
TCGTGAAAAAGACCAATTCAGGGGGAGAGTTTCTTCAAACAACAAGGAGCTGGGCAACTATGCAATCCAATGATATTGAG
R E K R P I Q G E S F F K Q Q G A G A T M Q S N D I E
TCGTGAAAAAGACCAATTCAGGGGGAGAGTTTCTTCAAACAACAAGGAGCTGGGCAACTATGCAATCCAATGATATTGAG
R E K R P I Q G E S F F K Q Q G A G A T M Q S N D I E
TCGTGAAAAAGACCAATTCAGGGGGAGAGTTTCTTCAAACAACAAGGAGCTGGGCAACTATGCAATCCAATGATATTGAG
R E K R P I Q G E S F F K Q Q G A G A T M Q S N D I E
TCGTGAAAAAGACCAATTCAGGGGGAGAGTTTCTTCAAACAACAAGGAGCTGGGCAACTATGCAATCCAATGATATTGAG
R E K R P I Q G E S F F K Q Q G A G A T M Q S N D I E
TCGTGAAAAAGACCAATTCAGGGGGAGAGTTTCTTCAAACAACAAGGAGCTGGGCAACTATGCAATCCAATGATATTGAG
R E K R P I Q G E S F F K Q Q G A G A T M Q S N D I E
TCGTGAAAAAGACCAATTCAGGGGGAGAGTTTCTTCAAACAACAAGGAGCTGGGCAACTATGCAATCCAATGATATTGAG
R E K R P I Q G E S F F K Q Q G A G A T M Q S N D I E
TCGTGAAAAAGACCAATTCAGGGGGAGAGTTTCTTCAAACAACAAGGAGCTGGGCAACTATGCAATCCAATGATATTGAG
R E K R P I Q G E S F F K Q Q G A G A T M Q S N D I E
TCGTGAAAAAGACCAATTCAGGGGGAGAGTTTCTTCAAACAACAAGGAGCTGGGCAACTATGCAATCCAATGATATTGAG
R E K R P I Q G E S F F K Q Q G A G A T M Q S N D I E
TCGTGAAAAAGACCAATTCAGGGGGAGAGTTTCTTCAAACAACAAGGAGCTGGGCAACTATGCAATCCAATGATATTGAG
R E K R P I Q G E S F F K Q Q G A G A T M Q S N D I E
TCGTGAAAAAGACCAATTCAGGGGGAGAGTTTCTTCAAACAACAAGGAGCTGGGCAACTATGCAATCCAATGATATTGAG
R E K R P I Q G E S F F K Q Q G A G A T M Q S N D I E
TCGTGAAAAAGACCAATTCAGGGGGAGAGTTTCTTCAAACAACAAGGAGCTGGGCAACTATGCAATCCAATGATATTGAG
R E K R P I Q G E S F F K Q Q G A G A T M Q S N D I E
TCGTGAAAAAGACCAATTCAGGGGGAGAGTTTCTTCAAACAACAAGGAGCTGGGCAACTATGCAATCCAATGATATTGAG
R E K R P I Q G E S F F K Q Q G A G A T M Q S N D I E
TCGTGAAAAAGACCAATTCAGGGGGAGAGTTTCTTCAAACAACAAGGAGCTGGGCAACTATGCAATCCAATGATATTGAG
R E K R P I Q G E S F F K Q Q G A G A T M Q S N D I E
TCGTGAAAAAGACCAATTCAGGGGGAGAGTTTCTTCAAACAACAAGGAGCTGGGCAACTATGCAATCCAATGATATTGAG
R E K R P I Q G E S F F K Q Q G A G A T M Q S N D I E
TCGTGAAAAAGACCAATTCAGGGGGAGAGTTTCTTCAAACAACAAGGAGCTGGGCAACTATGCAATCCAATGATATTGAG
R E K R P I Q G E S F F K Q Q G A G A T M Q S N D I E
TCGTGAAAAAGACCAATTCAGGGGGAGAGTTTCTTCAAACAACAAGGAGCTGGGCAACTATGCAATCCAATGATATTGAG
R E K R P I Q G E S F F K Q Q G A G A T M Q S N D I E
TCGTGAAAAAGACCAATTCAGGGGGAGAGTTTCTTCAAACAACAAGGAGCTGGGCAACTATGCAATCCAATGATATTGAG
R E K R P I Q G E S F F K Q Q G A G A T M Q S N D I E
TCGTGAAAAAGACCAATTCAGGGGGAGAGTTTCTTCAAACAACAAGGAGCTGGGCAACTATGCAATCCAATGATATTGAG
R E K R P I Q G E S F F K Q Q G A G A T M Q S N D I E
TCGTGAAAAAGACCAATTCAGGGGGAGAGTTTCTTCAAACAACAAGGAGCTGGGCAACTATGCAATCCAATGATATTGAG
R E K R P I Q G E S F F K Q Q G A G A T M Q S N D I E
TCGTGAAAAAGACCAATTCAGGGGGAGAGTTTCTTCAAACAACAAGGAGCTGGGCAACTATGCAATCCAATGATATTGAG
R E K R P I Q G E S F F K Q Q G A G A T M Q S N D I E
TCGTGAAAAAGACCAATTCAGGGGGAGAGTTTCTTCAAACAACAAGGAGCTGGGCAACTATGCAATCCAATGATATTGAG
R E K R P I Q G E S F F K Q Q G A G A T M Q S N D I E

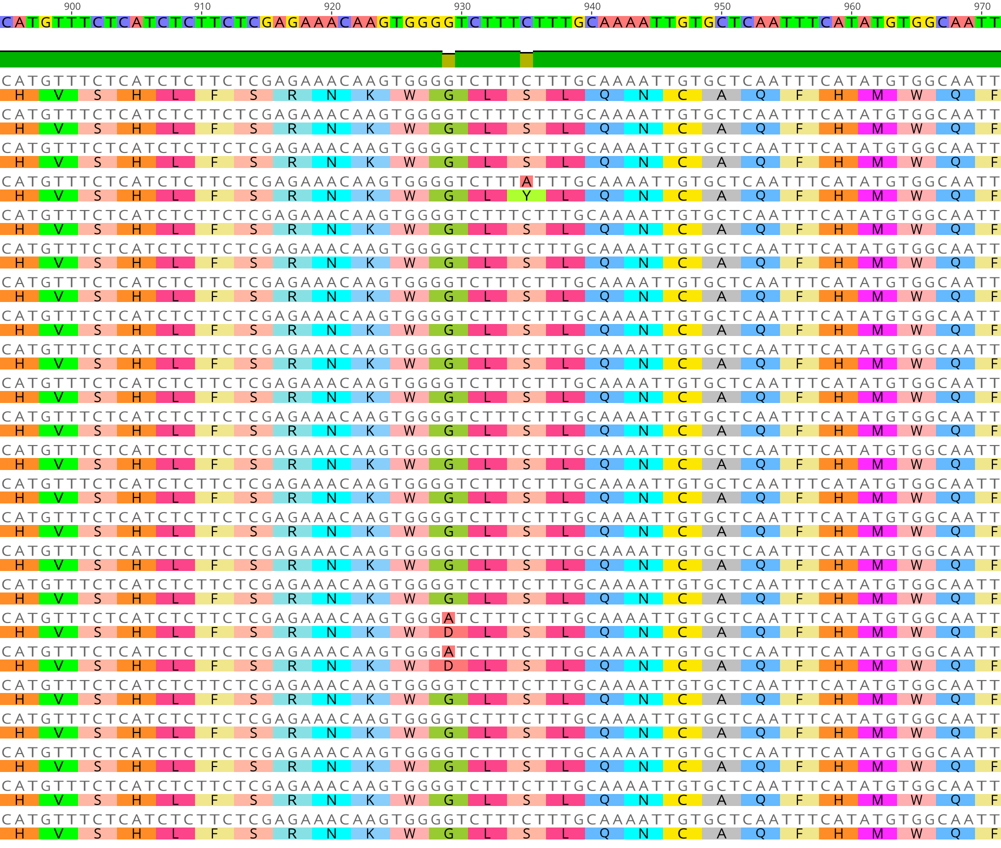
900 910 920 930 940 950 960 970
CATGTTTCTCATCTCTTCTCGAGAAACAAGTGGGGTCTTTCTTTGCAAAATTGTGCTCAATTTCATATGTGGCAATT
CATGTTTCTCATCTCTTCTCGAGAAACAAGTGGGGTCTTTCTTTGCAAAATTGTGCTCAATTTCATATGTGGCAATT
H V S H L F S R N K W G L S L Q N C A Q F H M W Q F
CATGTTTCTCATCTCTTCTCGAGAAACAAGTGGGGTCTTTCTTTGCAAAATTGTGCTCAATTTCATATGTGGCAATT
H V S H L F S R N K W G L S L Q N C A Q F H M W Q F
CATGTTTCTCATCTCTTCTCGAGAAACAAGTGGGGTCTTTCTTTGCAAAATTGTGCTCAATTTCATATGTGGCAATT
H V S H L F S R N K W G L S L Q N C A Q F H M W Q F
CATGTTTCTCATCTCTTCTCGAGAAACAAGTGGGGTCTTTATTTGCAAAATTGTGCTCAATTTCATATGTGGCAATT
H V S H L F S R N K W G L Y L Q N C A Q F H M W Q F
CATGTTTCTCATCTCTTCTCGAGAAACAAGTGGGGTCTTTCTTTGCAAAATTGTGCTCAATTTCATATGTGGCAATT
H V S H L F S R N K W G L S L Q N C A Q F H M W Q F
CATGTTTCTCATCTCTTCTCGAGAAACAAGTGGGGTCTTTCTTTGCAAAATTGTGCTCAATTTCATATGTGGCAATT
H V S H L F S R N K W G L S L Q N C A Q F H M W Q F
CATGTTTCTCATCTCTTCTCGAGAAACAAGTGGGGTCTTTCTTTGCAAAATTGTGCTCAATTTCATATGTGGCAATT
H V S H L F S R N K W G L S L Q N C A Q F H M W Q F
CATGTTTCTCATCTCTTCTCGAGAAACAAGTGGGGTCTTTCTTTGCAAAATTGTGCTCAATTTCATATGTGGCAATT
H V S H L F S R N K W G L S L Q N C A Q F H M W Q F
CATGTTTCTCATCTCTTCTCGAGAAACAAGTGGGGTCTTTCTTTGCAAAATTGTGCTCAATTTCATATGTGGCAATT
H V S H L F S R N K W G L S L Q N C A Q F H M W Q F
CATGTTTCTCATCTCTTCTCGAGAAACAAGTGGGGTCTTTCTTTGCAAAATTGTGCTCAATTTCATATGTGGCAATT
H V S H L F S R N K W G L S L Q N C A Q F H M W Q F
CATGTTTCTCATCTCTTCTCGAGAAACAAGTGGGGTCTTTCTTTGCAAAATTGTGCTCAATTTCATATGTGGCAATT
H V S H L F S R N K W G L S L Q N C A Q F H M W Q F
CATGTTTCTCATCTCTTCTCGAGAAACAAGTGGGGTCTTTCTTTGCAAAATTGTGCTCAATTTCATATGTGGCAATT
H V S H L F S R N K W G L S L Q N C A Q F H M W Q F
CATGTTTCTCATCTCTTCTCGAGAAACAAGTGGGGTCTTTCTTTGCAAAATTGTGCTCAATTTCATATGTGGCAATT
H V S H L F S R N K W G L S L Q N C A Q F H M W Q F
CATGTTTCTCATCTCTTCTCGAGAAACAAGTGGGGTCTTTCTTTGCAAAATTGTGCTCAATTTCATATGTGGCAATT
H V S H L F S R N K W G L S L Q N C A Q F H M W Q F
CATGTTTCTCATCTCTTCTCGAGAAACAAGTGGGGTCTTTCTTTGCAAAATTGTGCTCAATTTCATATGTGGCAATT
H V S H L F S R N K W G L S L Q N C A Q F H M W Q F
CATGTTTCTCATCTCTTCTCGAGAAACAAGTGGGGTCTTTCTTTGCAAAATTGTGCTCAATTTCATATGTGGCAATT
H V S H L F S R N K W G L S L Q N C A Q F H M W Q F
CATGTTTCTCATCTCTTCTCGAGAAACAAGTGGGATCTTTCTTTGCAAAATTGTGCTCAATTTCATATGTGGCAATT
H V S H L F S R N K W D L S L Q N C A Q F H M W Q F
CATGTTTCTCATCTCTTCTCGAGAAACAAGTGGGATCTTTCTTTGCAAAATTGTGCTCAATTTCATATGTGGCAATT
H V S H L F S R N K W D L S L Q N C A Q F H M W Q F
CATGTTTCTCATCTCTTCTCGAGAAACAAGTGGGGTCTTTCTTTGCAAAATTGTGCTCAATTTCATATGTGGCAATT
H V S H L F S R N K W G L S L Q N C A Q F H M W Q F
CATGTTTCTCATCTCTTCTCGAGAAACAAGTGGGGTCTTTCTTTGCAAAATTGTGCTCAATTTCATATGTGGCAATT
H V S H L F S R N K W G L S L Q N C A Q F H M W Q F
CATGTTTCTCATCTCTTCTCGAGAAACAAGTGGGGTCTTTCTTTGCAAAATTGTGCTCAATTTCATATGTGGCAATT
H V S H L F S R N K W G L S L Q N C A Q F H M W Q F
CATGTTTCTCATCTCTTCTCGAGAAACAAGTGGGGTCTTTCTTTGCAAAATTGTGCTCAATTTCATATGTGGCAATT
H V S H L F S R N K W G L S L Q N C A Q F H M W Q F
CATGTTTCTCATCTCTTCTCGAGAAACAAGTGGGGTCTTTCTTTGCAAAATTGTGCTCAATTTCATATGTGGCAATT
H V S H L F S R N K W G L S L Q N C A Q F H M W Q F

```
        980       990       1,000     1,010     1,020     1,030     1,040     1,050     1
CCGCCAAGATCTCTTCGTTAGTTGGGGGAAGAATCAGCACGAATCGGATTTTTTGAAGAACGTCTCGAGAGAGAATTGGATTTGGTTA

CCGCCAAGATCTCTTCGTTAGTTGGGGGAAGAATCAGCACGAATCGGATTTTTTGAAGAACGTCTCGAGAGAGAATTGGATTTGGTTA
 R  Q  D  L  F  V  S  W  G  K  N  Q  H  E  S  D  F  L  K  N  V  S  R  E  N  W  I  W  L
CCGCCAAGATCTCTTCGTTAGTTGGGGGAAGAATCAGCACGAATCGGATTTTTTGAAGAACGTCTCGAGAGAGAATTGGATTTGGTTA
 R  Q  D  L  F  V  S  W  G  K  N  Q  H  E  S  D  F  L  K  N  V  S  R  E  N  W  I  W  L
CCGCCAAGATCTCTTCGTTAGTTGGGGGAAGAATCAGCACGAATCGGATTTTTTGAAGAACGTCTCGAGAGAGAATTGGATTTGGTTA
 R  Q  D  L  F  V  S  W  G  K  N  Q  H  E  S  D  F  L  K  N  V  S  R  E  N  W  I  W  L
CCGCCAAGATCTCTTCGTTAGTTGGGGGAAGAATCAGCACGAATCGGATTTTTTGAAGAACGTCTCGAGAGAGAATTGGATTTGGTTA
 R  Q  D  L  F  V  S  W  G  K  N  Q  H  E  S  D  F  L  K  N  V  S  R  E  N  W  I  W  L
CCGCCAAGATCTCTTCGTTAGTTGGGGGAAGAATCAGCACGAATCGGATTTTTTGAAGAACGTCTCGAGAGAGAATTGGATTTGGTTA
 R  Q  D  L  F  V  S  W  G  K  N  Q  H  E  S  D  F  L  K  N  V  S  R  E  N  W  I  W  L
CCGCCAAGATCTCTTCGTTAGTTGGGGGAAGAATCAGCACGAATCGGATTTTTTGAAGAACGTCTCGAGAGAGAATTGGATTTGGTTA
 R  Q  D  L  F  V  S  W  G  K  N  Q  H  E  S  D  F  L  K  N  V  S  R  E  N  W  I  W  L
CCGCCAAGATCTCTTCGTTAGTTGGGGGAAGAATCAGCACGAATCGGATTTTTTGAAGAACGTCTCGAGAGAGAATTGGATTTGGTTA
 R  Q  D  L  F  V  S  W  G  K  N  Q  H  E  S  D  F  L  K  N  V  S  R  E  N  W  I  W  L
CCGCCAAGATCTCTTCGTTAGTTGGGGGAAGAATCAGCACGAATCGGATTTTTTGAAGAACGTCTCGAGAGAGAATTGGATTTGGTTA
 R  Q  D  L  F  V  S  W  G  K  N  Q  H  E  S  D  F  L  K  N  V  S  R  E  N  W  I  W  L
CCGCCAAGATCTCTTCGTTAGTTGGGGGAAGAATCAGCACGAATCGGATTTTTTGAAGAACGTCTCGAGAGAGAATTGGATTTGGTTA
 R  Q  D  L  F  V  S  W  G  K  N  Q  H  E  S  D  F  L  K  N  V  S  R  E  N  W  I  W  L
CCGCCAAGATCTCTTCGTTAGTTGGGGGAAGAATCAGCACGAATCGGATTTTTTGAAGAACGTCTCGAGAGAGAATTGGATTTGGTTA
 R  Q  D  L  F  V  S  W  G  K  N  Q  H  E  S  D  F  L  K  N  V  S  R  E  N  W  I  W  L
CCGCCAAGATCTCTTCGTTAGTTGGGGGAAGAATCAGCACGAATCGGATTTTTTGAAGAACGTCTCGAGAGAGAATTGGATTTGGTTA
 R  Q  D  L  F  V  S  W  G  K  N  Q  H  E  S  D  F  L  K  N  V  S  R  E  N  W  I  W  L
CCGCCAAGATCTCTTCGTTAGTTGGGGGAAGAATCAGCACGAATCGGATTTTTTGAAGAACGTCTCGAGAGAGAATTGGATTTGGTTA
 R  Q  D  L  F  V  S  W  G  K  N  Q  H  E  S  D  F  L  K  N  V  S  R  E  N  W  I  W  L
CCGCCAAGATCTCTTCGTTAGTTGGGGGAAGAATCAGCACGAATCGGATTTTTTGAAGAACGTCTCGAGAGAGAATTGGATTTGGTTA
 R  Q  D  L  F  V  S  W  G  K  N  Q  H  E  S  D  F  L  K  N  V  S  R  E  N  W  I  W  L
CCGCCAAGATCTCTTCGTTAGTTGGGGGAAGAATCAGCACGAATCGGATTTTTTGAAGAACGTCTCGAGAGAGAATTGGATTTGGTTA
 R  Q  D  L  F  V  S  W  G  K  N  Q  H  E  S  D  F  L  K  N  V  S  R  E  N  W  I  W  L
CCGCCAAGATCTCTTCGTTAGTTGGGGGAAGAATCAGCACGAATCGGATTTTTTGAAGAACGTCTCGAGAGAGAATTGGATTTGGTTA
 R  Q  D  L  F  V  S  W  G  K  N  Q  H  E  S  D  F  L  K  N  V  S  R  E  N  W  I  W  L
CCGCCAAGATCTCTTCGTTAGTTGGGGGAAGAATCAGCACGAATCGGATTTTTTGAAGAACGTCTCGAGAGAGAATTGGATTTGGTTA
 R  Q  D  L  F  V  S  W  G  K  N  Q  H  E  S  D  F  L  K  N  V  S  R  E  N  W  I  W  L
CCGCCAAGATCTCTTCGTTAGTTGGGGGAAGAATCAGCACGAATCGGATTTTTTGAAGAACGTCTCGAGAGAGAATTGGATTTGGTTA
 R  Q  D  L  F  V  S  W  G  K  N  Q  H  E  S  D  F  L  K  N  V  S  R  E  N  W  I  W  L
CCGCCAAGATCTCTTCGTTAGTTGGGGGAAGAATCAGCACGAATCGGATTTTTTGAAGAACGTCTCGAGAGAGAATTGGATTTGGTTA
 R  Q  D  L  F  V  S  W  G  K  N  Q  H  E  S  D  F  L  K  N  V  S  R  E  N  W  I  W  L
CCGCCAAGATCTCTTCGTTAGTTGGGGGAAGAATCAGCACGAATCGGATTTTTTGAAGAACGTCTCGAGAGAGAATTGGATTTGGTTA
 R  Q  D  L  F  V  S  W  G  K  N  Q  H  E  S  D  F  L  K  N  V  S  R  E  N  W  I  W  L
CCGCCAAGATCTCTTCGTTAGTTGGGGGAAGAATCAGCACGAATCGGATTTTTTGAAGAACGTCTCGAGAGAGAATTGGATTTGGTTA
 R  Q  D  L  F  V  S  W  G  K  N  Q  H  E  S  D  F  L  K  N  V  S  R  E  N  W  I  W  L
CCGCCAAGATCTCTTCGTTAGTTGGGGGAAGAATCAGCACGAATCGGATTTTTTGAAGAACGTCTCGAGAGAGAATTGGATTTGGTTA
 R  Q  D  L  F  V  S  W  G  K  N  Q  H  E  S  D  F  L  K  N  V  S  R  E  N  W  I  W  L
CCGCCAAGATCTCTTCGTTAGTTGGGGGAAGAATCAGCACGAATCGGATTTTTTGAAGAACGTCTCGAGAGAGAATTGGATTTGGTTA
 R  Q  D  L  F  V  S  W  G  K  N  Q  H  E  S  D  F  L  K  N  V  S  R  E  N  W  I  W  L
CCGCCAAGATCTCTTCGTTAGTTGGGGGAAGAATCAGCACGAATCGGATTTTTTGAAGAACGTCTCGAGAGAGAATTGGATTTGGTTA
 R  Q  D  L  F  V  S  W  G  K  N  Q  H  E  S  D  F  L  K  N  V  S  R  E  N  W  I  W  L
```

```
,060      1,070      1,080      1,090      1,100      1,110      1,120      1,130      1
GACAATGTGTGGTTGGTAAACAAGGATCGGTTTTTTAGCAAGGTACGGAATGTATTGTCAAATATTCAATATGATTCCAC

GACAATGTGTGGTTGGTAAACAAGGATCGGTTTTTTAGCAAGGTACGGAATGTATTGTCAAATATTCAATATGATTCCAC
 D  N  V  W  L  V  N  K  D  R  F  F  S  K  V  R  N  V  L  S  N  I  Q  Y  D  S  T
GACAATGTGTGGTTGGTAAACAAGGATCGGTTTTTTAGCAAGGTACGGAATGTATTGTCAAATATTCAATATGATTCCAC
 D  N  V  W  L  V  N  K  D  R  F  F  S  K  V  R  N  V  L  S  N  I  Q  Y  D  S  T
GACAATGTGTGGTTGGTAAACAAGGATCGGTTTTTTAGCAAGGTACGGAATGTATTGTCAAATATTCAATATGATTCCAC
 D  N  V  W  L  V  N  K  D  R  F  F  S  K  V  R  N  V  L  S  N  I  Q  Y  D  S  T
GACAATGTGTGGTTGGTAAACAAGGATCGGTTTTTTAGCAAGGTACGGAATGTATTGTCAAATATTCAATATGATTCCAC
 D  N  V  W  L  V  N  K  D  R  F  F  S  K  V  R  N  V  L  S  N  I  Q  Y  D  S  T
GACAATGTGTGGTTGGTAAACAAGGATCGGTTTTTTAGCAAGGTACGGAATGTATTGTCAAATATTCAATATGATTCCAC
 D  N  V  W  L  V  N  K  D  R  F  F  S  K  V  R  N  V  L  S  N  I  Q  Y  D  S  T
GACAATGTGTGGTTGGTAAACAAGGATCGGTTTTTTAGCAAGGTACGGAATGTATTGTCAAATATTCAATATGATTCCAC
 D  N  V  W  L  V  N  K  D  R  F  F  S  K  V  R  N  V  L  S  N  I  Q  Y  D  S  T
GACAATGTGTGGTTGGTAAACAAGGATCGGTTTTTTAGCAAGGTACGGAATGTATTGTCAAATATTCAATATGATTCCAC
 D  N  V  W  L  V  N  K  D  R  F  F  S  K  V  R  N  V  L  S  N  I  Q  Y  D  S  T
GACAATGTGTGGTTGGTAAACAAGGATCGGTTTTTTAGCAAGGTACGGAATGTATTGTCAAATATTCAATATGATTCCAC
 D  N  V  W  L  V  N  K  D  R  F  F  S  K  V  R  N  V  L  S  N  I  Q  Y  D  S  T
GACAATGTGTGGTTGGTAAACAAGGATCGGTTTTTTAGCAAGGTACGGAATGTATTGTCAAATATTCAATATGATTCCAC
 D  N  V  W  L  V  N  K  D  R  F  F  S  K  V  R  N  V  L  S  N  I  Q  Y  D  S  T
GACAATGTGTGGTTGGTAAACAAGGATCGGTTTTTTAGCAAGGTACGGAATGTATTGTCAAATATTCAATATGATTCCAC
 D  N  V  W  L  V  N  K  D  R  F  F  S  K  V  R  N  V  L  S  N  I  Q  Y  D  S  T
GACAATGTGTGGTTGGTAAACAAGGATCGGTTTTTTAGCAAGGTACGGAATGTATTGTCAAATATTCAATATGATTCCAC
 D  N  V  W  L  V  N  K  D  R  F  F  S  K  V  R  N  V  L  S  N  I  Q  Y  D  S  T
GACAATGTGTGGTTGGTAAACAAGGATCGGTTTTTTAGCAAGGTACGGAATGTATTGTCAAATATTCAATATGATTCCAC
 D  N  V  W  L  V  N  K  D  R  F  F  S  K  V  R  N  V  L  S  N  I  Q  Y  D  S  T
GACAATGTGTGGTTGGTAAACAAGGATCGGTTTTTTAGCAAGGTACGGAATGTATTGTCAAATATTCAATATGATTCCAC
 D  N  V  W  L  V  N  K  D  R  F  F  S  K  V  R  N  V  L  S  N  I  Q  Y  D  S  T
GACAATGTGTGGTTGGTAAACAAGGATCGGTTTTTTAGCAAGGTACGGAATGTATTGTCAAATATTCAATATGATTCCAC
 D  N  V  W  L  V  N  K  D  R  F  F  S  K  V  R  N  V  L  S  N  I  Q  Y  D  S  T
GACAATGTGTGGTTGGTAAACAAGGATCGGTTTTTTAGCAAGGTACGGAATGTATTGTCAAATATTCAATATGATTCCAC
 D  N  V  W  L  V  N  K  D  R  F  F  S  K  V  R  N  V  L  S  N  I  Q  Y  D  S  T
GACAATGTGTGGTTGGTAAACAAGGATCGGTTTTTTAGCAAGGTACGGAATGTATTGTCAAATATTCAATATGATTCCAC
 D  N  V  W  L  V  N  K  D  R  F  F  S  K  V  R  N  V  L  S  N  I  Q  Y  D  S  T
GACAATGTGTGGTTGGTAAACAAGGATCGGTTTTTTAGCAAGGTACGGAATGTATTTTCAAATATTCAATATGATTCCAC
 D  N  V  W  L  V  N  K  D  R  F  F  S  K  V  R  N  V  F  S  N  I  Q  Y  D  S  T
GACAATGTGTGGTTGGTAAACAAGGATCGGTTTTTTAGCAAGGTACGGAATGTATTGTCAAATATTCAATATGATTCCAC
 D  N  V  W  L  V  N  K  D  R  F  F  S  K  V  R  N  V  L  S  N  I  Q  Y  D  S  T
GACAATGTGTGGTTGGTAAACAAGGATCGGTTTTTTAGCAAGGTACGGAATGTATTGTCAAATATTCAATATGATTCCAC
 D  N  V  W  L  V  N  K  D  R  F  F  S  K  V  R  N  V  L  S  N  I  Q  Y  D  S  T
GACAATGTGTGGTTGGTAAACAAGGATCGGTTTTTTAGCAAGGTACGGAATGTATTGTCAAATATTCAATATGATTCCAC
 D  N  V  W  L  V  N  K  D  R  F  F  S  K  V  R  N  V  L  S  N  I  Q  Y  D  S  T
GACAATGTGTGGTTGGTAAACAAGGATCGGTTTTTTAGCAAGGTACGGAATGTATTGTCAAATATTCAATATGATTCCAC
 D  N  V  W  L  V  N  K  D  R  F  F  S  K  V  R  N  V  L  S  N  I  Q  Y  D  S  T
GACAATGTGTGGTTGGTAAACAAGGATCGGTTTTTTAGCAAGGTACGGAATGTATTGTCAAATATTCAATATGATTCCAC
 D  N  V  W  L  V  N  K  D  R  F  F  S  K  V  R  N  V  L  S  N  I  Q  Y  D  S  T
GACAATGTGTGGTTGGTAAACAAGGATCGGTTTTTTAGCAAGGTACGGAATGTATTGTCAAATATTCAATATGATTCCAC
 D  N  V  W  L  V  N  K  D  R  F  F  S  K  V  R  N  V  L  S  N  I  Q  Y  D  S  T
GACAATGTCTGGTTGGTAAACAAGGATCGGTTTTTTAGCAAGGTACGGAATGTATTGTCAAATATTCAATATGATTCCAC
 D  N  V  W  L  V  N  K  D  R  F  F  S  K  V  R  N  V  L  S  N  I  Q  Y  D  S  T
```

```
1,140     1,150     1,160     1,170     1,180     1,190     1,200     1,210
AAGATCTATTTTCGTTCAAGTAACGGATTCTAGCCAATGGAAAGGATCTTCTTCTGATCAATCCAGAGATCATT

AAGATCTATTTTCGTTCAAGTAACGGATTCTAGCCAATGGAAAGGATCTTCTTCTGATCAATCCAGAGATCATT
  R  S  I  F  V  Q  V  T  D  S  S  Q  W  K  G  S  S  S  D  Q  S  R  D  H
AAGATCTATTTTCGTTCAAGTAACGGATTCTAGCCAATGGAAAGGATCTTCTTCTGATCAATCCAGAGATCATT
  R  S  I  F  V  Q  V  T  D  S  S  Q  W  K  G  S  S  S  D  Q  S  R  D  H
AAGATCTATTTTCGTTCAAGTAACGGATTCTAGCCAATGGAAAGGATCTTCTTCTGATCAATCCAGAGATCATT
  R  S  I  F  V  Q  V  T  D  S  S  Q  W  K  G  S  S  S  D  Q  S  R  D  H
AAGATCTATTTTCGTTCAAGTAACGGATTCTAGCCAATGGAAAGGATCTTCTTCTGATCAATCCAGAGATCATT
  R  S  I  F  V  Q  V  T  D  S  S  Q  W  K  G  S  S  S  D  Q  S  R  D  H
AAGATCTATTTTCGTTCAAGTAACGGATTCTAGCCAATGGAAAGGATCTTCTTCTGATCAATCCAGAGATCATT
  R  S  I  F  V  Q  V  T  D  S  S  Q  W  K  G  S  S  S  D  Q  S  R  D  H
AAGATCTATTTTCGTTCAAGTAACGGATTCTAGCCAATGGAAAGGATCTTCTTCTGATCAATCCAGAGATCATT
  R  S  I  F  V  Q  V  T  D  S  S  Q  W  K  G  S  S  S  D  Q  S  R  D  H
AAGATCTATTTTCGTTCAAGTAACGGATTCTAGCCAATGGAAAGGATCTTCTTCTGATCAATCCAGAGATCATT
  R  S  I  F  V  Q  V  T  D  S  S  Q  W  K  G  S  S  S  D  Q  S  R  D  H
AAGATCTATTTTCGTTCAAGTAACGGATTCTAGCCAATGGAAAGGATCTTCTTCTGATCAATCCAGAGATCATT
  R  S  I  F  V  Q  V  T  D  S  S  Q  W  K  G  S  S  S  D  Q  S  R  D  H
AAGATCTATTTTCGTTCAAGTAACGGATTCTAGCCAATGGAAAGGATCTTCTTCTGATCAATCCAGAGATCATT
  R  S  I  F  V  Q  V  T  D  S  S  Q  W  K  G  S  S  S  D  Q  S  R  D  H
AAGATCTATTTTCGTTCAAGTAACGGATTCTAGCCAATGGAAAGGATCTTCTTCTGATCAATCCAGAGATCATT
  R  S  I  F  V  Q  V  T  D  S  S  Q  W  K  G  S  S  S  D  Q  S  R  D  H
AAGATCTATTTTCGTTCAAGTAACGGATTCTAGCCAATGGAAAGGATCTTCTTCTGATCAATCCAGAGATCATT
  R  S  I  F  V  Q  V  T  D  S  S  Q  W  K  G  S  S  S  D  Q  S  R  D  H
AAGATCTATTTTCGTTCAAGTAACGGATTCTAGCCAATGGAAAGGATCTTCTTCTGATCAATCCAGAGATCATT
  R  S  I  F  V  Q  V  T  D  S  S  Q  W  K  G  S  S  S  D  Q  S  R  D  H
AAGATCTATTTTCGTTCAAGTAACGGATTCTAGCCAATGGAAAGGATCTTCTTCTGATCAATCCAGAGATCATT
  R  S  I  F  V  Q  V  T  D  S  S  Q  W  K  G  S  S  S  D  Q  S  R  D  H
AAGATCTATTTTCGTTCAAGTAACGGATTCTAGCCAATGGAAAGGATCTTCTTCTGATCAATCCAGAGATCATT
  R  S  I  F  V  Q  V  T  D  S  S  Q  W  K  G  S  S  S  D  Q  S  R  D  H
AAGATCTATTTTCGTTCAAGTAACGGATTCTAGCCAATGGAAAGGATCTTCTTCTGATCAATCCAGAGATCATT
  R  S  I  F  V  Q  V  T  D  S  S  Q  W  K  G  S  S  S  D  Q  S  R  D  H
AAGATCTATTTTCGTTCAAGTAACGGATTCTAGCCAATGGAAAGGATCTTCTTCTGATCAATCCAGAGATCATT
  R  S  I  F  V  Q  V  T  D  S  S  Q  W  K  G  S  S  S  D  Q  S  R  D  H
AAGATCTATTTTCGTTCAAGTAACGGATTCTAGCCAATGGAAAGGATCTTCTTCTGATCAATCCAGAGATCATT
  R  S  I  F  V  Q  V  T  D  S  S  Q  W  K  G  S  S  S  D  Q  S  R  D  H
AAGATCTATTTTCGTTCAAGTAACGGATTCTAGCCAATGGAAAGGATCTTCTTCTGATCAATCCAGAGATCATT
  R  S  I  F  V  Q  V  T  D  S  S  Q  W  K  G  S  S  S  D  Q  S  R  D  H
AAGATCTATTTTCGTTCAAGTAACGGATTCTAGCCAATGGAAAGGATCTTCTTCTGATCAATCCAGAGATCATT
  R  S  I  F  V  Q  V  T  D  S  S  Q  W  K  G  S  S  S  D  Q  S  R  D  H
AAGATCTATTTTCGTTCAAGTAACGGATTCTAGCCAATGGAAAGGATCTTCTTCTGATCAATCCAGAGATCATT
  R  S  I  F  V  Q  V  T  D  S  S  Q  W  K  G  S  S  S  D  Q  S  R  D  H
AAGATCTATTTTCGTTCAAGTAACGGATTCTAGCCAATGGAAAGGATCTTCTTCTGATCAATCCAGAGATCATT
  R  S  I  F  V  Q  V  T  D  S  S  Q  W  K  G  S  S  S  D  Q  S  R  D  H
AAGATCTATTTTCGTTCAAGTAACGGATTCTAGCCAATGGAAAGGATCTTCTTCTGATCAATCCAGAGATCATT
  R  S  I  F  V  Q  V  T  D  S  S  Q  W  K  G  S  S  S  D  Q  S  R  D  H
AAGATCTATTTTCGTTCAAGTAACGGATTCTAGCCAATGGAAAGGATCTTCTTCTGATCAATCCAGAGATCATT
  R  S  I  F  V  Q  V  T  D  S  S  Q  W  K  G  S  S  S  D  Q  S  R  D  H
AAG-------TTTCGTTCAAGTAACGGATTCTAGCCAATGGAAAGGATCTTCTTCTGATCAATCCAGAGATCATT
  S -------  F  V  Q  V  T  D  S  S  Q  W  K  G  S  S  S  D  Q  S  R  D  H
```

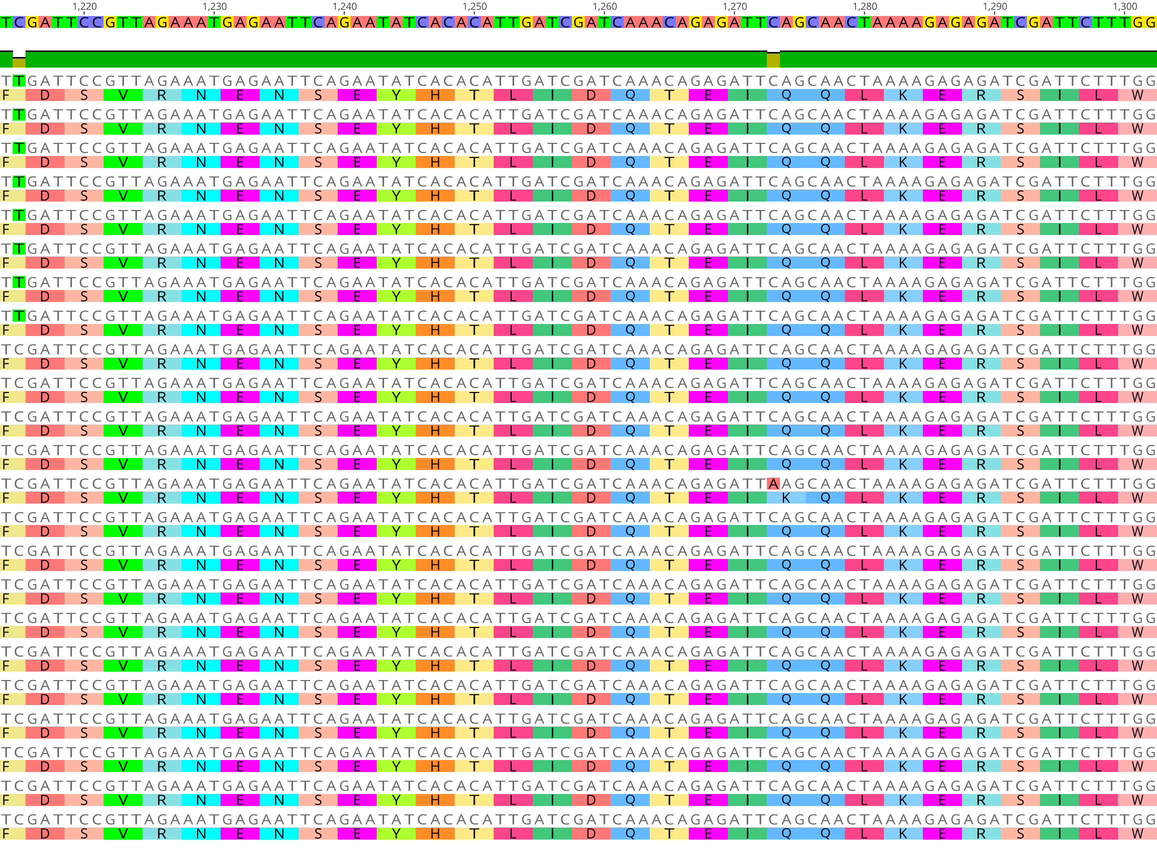

1,220 1,230 1,240 1,250 1,260 1,270 1,280 1,290 1,300
TCGATTCCGTTAGAAATGAGAATTCAGAATATCACACATTGATCGATCAAACAGAGATTCAGCAACTAAAAGAGAGATCGATTCTTTGG
TTGATTCCGTTAGAAATGAGAATTCAGAATATCACACATTGATCGATCAAACAGAGATTCAGCAACTAAAAGAGAGATCGATTCTTTGG
F D S V R N E N S E Y H T L I D Q T E I Q Q L K E R S I L W
TTGATTCCGTTAGAAATGAGAATTCAGAATATCACACATTGATCGATCAAACAGAGATTCAGCAACTAAAAGAGAGATCGATTCTTTGG
F D S V R N E N S E Y H T L I D Q T E I Q Q L K E R S I L W
TTGATTCCGTTAGAAATGAGAATTCAGAATATCACACATTGATCGATCAAACAGAGATTCAGCAACTAAAAGAGAGATCGATTCTTTGG
F D S V R N E N S E Y H T L I D Q T E I Q Q L K E R S I L W
TTGATTCCGTTAGAAATGAGAATTCAGAATATCACACATTGATCGATCAAACAGAGATTCAGCAACTAAAAGAGAGATCGATTCTTTGG
F D S V R N E N S E Y H T L I D Q T E I Q Q L K E R S I L W
TTGATTCCGTTAGAAATGAGAATTCAGAATATCACACATTGATCGATCAAACAGAGATTCAGCAACTAAAAGAGAGATCGATTCTTTGG
F D S V R N E N S E Y H T L I D Q T E I Q Q L K E R S I L W
TTGATTCCGTTAGAAATGAGAATTCAGAATATCACACATTGATCGATCAAACAGAGATTCAGCAACTAAAAGAGAGATCGATTCTTTGG
F D S V R N E N S E Y H T L I D Q T E I Q Q L K E R S I L W
TTGATTCCGTTAGAAATGAGAATTCAGAATATCACACATTGATCGATCAAACAGAGATTCAGCAACTAAAAGAGAGATCGATTCTTTGG
F D S V R N E N S E Y H T L I D Q T E I Q Q L K E R S I L W
TTGATTCCGTTAGAAATGAGAATTCAGAATATCACACATTGATCGATCAAACAGAGATTCAGCAACTAAAAGAGAGATCGATTCTTTGG
F D S V R N E N S E Y H T L I D Q T E I Q Q L K E R S I L W
TTGATTCCGTTAGAAATGAGAATTCAGAATATCACACATTGATCGATCAAACAGAGATTCAGCAACTAAAAGAGAGATCGATTCTTTGG
F D S V R N E N S E Y H T L I D Q T E I Q Q L K E R S I L W
TCGATTCCGTTAGAAATGAGAATTCAGAATATCACACATTGATCGATCAAACAGAGATTCAGCAACTAAAAGAGAGATCGATTCTTTGG
F D S V R N E N S E Y H T L I D Q T E I Q Q L K E R S I L W
TCGATTCCGTTAGAAATGAGAATTCAGAATATCACACATTGATCGATCAAACAGAGATTCAGCAACTAAAAGAGAGATCGATTCTTTGG
F D S V R N E N S E Y H T L I D Q T E I Q Q L K E R S I L W
TCGATTCCGTTAGAAATGAGAATTCAGAATATCACACATTGATCGATCAAACAGAGATTCAGCAACTAAAAGAGAGATCGATTCTTTGG
F D S V R N E N S E Y H T L I D Q T E I Q Q L K E R S I L W
TCGATTCCGTTAGAAATGAGAATTCAGAATATCACACATTGATCGATCAAACAGAGATTCAGCAACTAAAAGAGAGATCGATTCTTTGG
F D S V R N E N S E Y H T L I D Q T E I Q Q L K E R S I L W
TCGATTCCGTTAGAAATGAGAATTCAGAATATCACACATTGATCGATCAAACAGAGATTAAGCAACTAAAAGAGAGATCGATTCTTTGG
F D S V R N E N S E Y H T L I D Q T E I K Q L K E R S I L W
TCGATTCCGTTAGAAATGAGAATTCAGAATATCACACATTGATCGATCAAACAGAGATTCAGCAACTAAAAGAGAGATCGATTCTTTGG
F D S V R N E N S E Y H T L I D Q T E I Q Q L K E R S I L W
TCGATTCCGTTAGAAATGAGAATTCAGAATATCACACATTGATCGATCAAACAGAGATTCAGCAACTAAAAGAGAGATCGATTCTTTGG
F D S V R N E N S E Y H T L I D Q T E I Q Q L K E R S I L W
TCGATTCCGTTAGAAATGAGAATTCAGAATATCACACATTGATCGATCAAACAGAGATTCAGCAACTAAAAGAGAGATCGATTCTTTGG
F D S V R N E N S E Y H T L I D Q T E I Q Q L K E R S I L W
TCGATTCCGTTAGAAATGAGAATTCAGAATATCACACATTGATCGATCAAACAGAGATTCAGCAACTAAAAGAGAGATCGATTCTTTGG
F D S V R N E N S E Y H T L I D Q T E I Q Q L K E R S I L W
TCGATTCCGTTAGAAATGAGAATTCAGAATATCACACATTGATCGATCAAACAGAGATTCAGCAACTAAAAGAGAGATCGATTCTTTGG
F D S V R N E N S E Y H T L I D Q T E I Q Q L K E R S I L W
TCGATTCCGTTAGAAATGAGAATTCAGAATATCACACATTGATCGATCAAACAGAGATTCAGCAACTAAAAGAGAGATCGATTCTTTGG
F D S V R N E N S E Y H T L I D Q T E I Q Q L K E R S I L W
TCGATTCCGTTAGAAATGAGAATTCAGAATATCACACATTGATCGATCAAACAGAGATTCAGCAACTAAAAGAGAGATCGATTCTTTGG
F D S V R N E N S E Y H T L I D Q T E I Q Q L K E R S I L W
TCGATTCCGTTAGAAATGAGAATTCAGAATATCACACATTGATCGATCAAACAGAGATTCAGCAACTAAAAGAGAGATCGATTCTTTGG
F D S V R N E N S E Y H T L I D Q T E I Q Q L K E R S I L W
TCGATTCCGTTAGAAATGAGAATTCAGAATATCACACATTGATCGATCAAACAGAGATTCAGCAACTAAAAGAGAGATCGATTCTTTGG
F D S V R N E N S E Y H T L I D Q T E I Q Q L K E R S I L W
TCGATTCCGTTAGAAATGAGAATTCAGAATATCACACATTGATCGATCAAACAGAGATTCAGCAACTAAAAGAGAGATCGATTCTTTGG
F D S V R N E N S E Y H T L I D Q T E I Q Q L K E R S I L W

1,310 1,320 1,330 1,340 1,350 1,360 1,370 1,380 1,390

GATCCTTCCTTTCTTCAAACGGAACGAACAGAGATAGAATCAGATCGATTCCCGAAATGCCTTTTTGGATCTTCCTCCATGTCCTGGCTAT

GATCCTTCCTTTCTTCAAACGGAACGAACAGAGATAGAATCAGATCGATTCCCGAAATGCCTTTTTGGATCTTCCTCCATGTCCTGGCTAT
D P S F L Q T E R T E I E S D R F P K C L F G S S S M S W L

GATCCTTCCTTTCTTCAAACGGAACGAACAGAGATAGAATCAGATCGATTCCCGAAATGCCTTTTTGGATCTTCCTCCATGTCCTGGCTAT
D P S F L Q T E R T E I E S D R F P K C L F G S S S M S W L

GATCCTTCCTTTCTTCAAACGGAACGAACAGAGATAGAATCAGATCGATTCCCGAAATGCCTTTTTGGATCTTCCTCCATGTCCTGGCTAT
D P S F L Q T E R T E I E S D R F P K C L F G S S S M S W L

GATCCTTCCTTTCTTCAAACGGAACGAACAGAGATAGAATCAGATCGATTCCCGAAATGCCTTTTTGGATCTTCCTCCATGTCCTGGCTAT
D P S F L Q T E R T E I E S D R F P K C L F G S S S M S W L

GATCCTTCCTTTCTTCAAACGGAACGAACAGAGATAGAATCAGATCGATTCCCGAAATGCCTTTTTGGATCTTCCTCCATGTCCTGGCTAT
D P S F L Q T E R T E I E S D R F P K C L F G S S S M S W L

GATCCTTCCTTTCTTCAAACGGAACGAACAGAGATAGAATCAGATCGATTCCCGAAATGCCTTTTTGGATCTTCCTCCATGTCCTGGCTAT
D P S F L Q T E R T E I E S D R F P K C L F G S S S M S W L

GATCCTTCCTTTCTTCAAACGGAACGAACAGAGATAGAATCAGATCGATTCCCGAAATGCCTTTTTGGATCTTCCTCCATGTCCTGGCTAT
D P S F L Q T E R T E I E S D R F P K C L F G S S S M S W L

GATCCTTCCTTTCTTCAAACGGAACGAACAGAGATAGAATCAGATCGATTCCCGAAATGCCTTTTTGGATCTTCCTCCATGTCCTGGCTAT
D P S F L Q T E R T E I E S D R F P K C L F G S S S M S W L

GATCCTTCCTTTCTTCAAACGGAACGAACAGAGATAGAATCAGATCGATTCCCGAAATGCCTTTTTGGATCTTCCTCCATGTCCTGGCTAT
D P S F L Q T E R T E I E S D R F P K C L F G S S S M S W L

GATCCTTCCTTTCTTCAAACGGAACGAACAGAGATAGAATCAGATCGATTCCCGAAATGCCTTTTTGGATCTTCCTCCATGTCCTGGCTAT
D P S F L Q T E R T E I E S D R F P K C L F G S S S M S W L

GATCCTTCCTTTCTTCAAACGGAACGAACAGAGATAGAATCAGATCGATTCCCGAAATGCCTTTTTGGATCTTCCTCCATGTCCTGGCTAT
D P S F L Q T E R T E I E S D R F P K C L F G S S S M S W L

GATCCTTCCTTTCTTCAAACGGAACGAACAGAGATAGAATCAGATCGATTCCCGAAATGCCTTTTTGGATCTTCCTCCATATCCTGGCTAT
D P S F L Q T E R T E I E S D R F P K C L F G S S S I S W L

GATCCTTCCTTTCTTCAAACGGAACGAACAGAGATAGAATCAGATCGATTCCCGAAATGCCTTTTTGGATCTTCCTCCATGTCCTGGCTAT
D P S F L Q T E R T E I E S D R F P K C L F G S S S M S W L

GATCCTTCCTTTCTTCAAACGGAACGAACAGAGATAGAATCAGATCGATTCCCGAAATGCCTTTTTGGATCTTCCTCCATGTCCTGGCTAT
D P S F L Q T E R T E I E S D R F P K C L F G S S S M S W L

GATCCTTCCTTTCTTCAAACGGAACGAACAGAGATAGAATCAGATCGATTCCCGAAATGCCTTTTTGGATCTTCCTCCATGTCCTGGCTAT
D P S F L Q T E R T E I E S D R F P K C L F G S S S M S W L

GATCCTTCCTTTCTTCAAACGGAACGAACAGAGATAGAATCAGATCGATTCCCGAAATGCCTTTTTGGATCTTCCTCCATGTCCTGGCTAT
D P S F L Q T E R T E I E S D R F P K C L F G S S S M S W L

GATCCTTCCTTTCTTCAAACGGAACGAACAGAGATAGAATCAGATCGATTCCCGAAATGCCTTTTTGGATCTTCCTCCATGTCCTGGCTAT
D P S F L Q T E R T E I E S D R F P K C L F G S S S M S W L

GATCCTTCCTTTCTTCAAACGGAACGAACAGAGATAGAATCAGATCGATTCCCGAAATGCCTTTTTGGATCTTCCTCCATGTCCTGGCTAT
D P S F L Q T E R T E I E S D R F P K C L F G S S S M S W L

GATCCTTCCTTTCTTCAAACGGAACGAACAGAGATAGAATCAGATCGATTCCCGAAATGCCTTTTTGGATCTTCCTCCATGTCCTGGCTAT
D P S F L Q T E R T E I E S D R F P K C L F G S S S M S W L

GATCCTTCCTTTCTTCAAACGGAACGAACAGAGATAGAATCAGATCGATTCCCGAAATGCCTTTTTGGATCTTCCTCCATGTCCTGGCTAT
D P S F L Q T E R T E I E S D R F P K C L F G S S S M S W L

GATCCTTCCTTTCTTCAAACGGAACGAACAGAGATAGAATCAGATCGATTCCCGAAATGCCTTTTTGGATCTTCCTCCATGTCCTGGCTAT
D P S F L Q T E R T E I E S D R F P K C L F G S S S M S W L

GATCCTTCCTTTCTTCAAACGGAACGAACAGAGATAGAATCAGATCGATTCCCGAAATGCCTTTTTGGATCTTCCTCCATGTCCTGGCTAT
D P S F L Q T E R T E I E S D R F P K C L F G S S S M S W L

GATCCTTCCTTTCTTCAAACGGAACGAACAGAGATAGAATCAGATCGATTCCCGAAATGCCTTTTTGGATCTTCCTCCATGTCCTGGCTAT
D P S F L Q T E R T E I E S D R F P K C L F G S S S M S W L

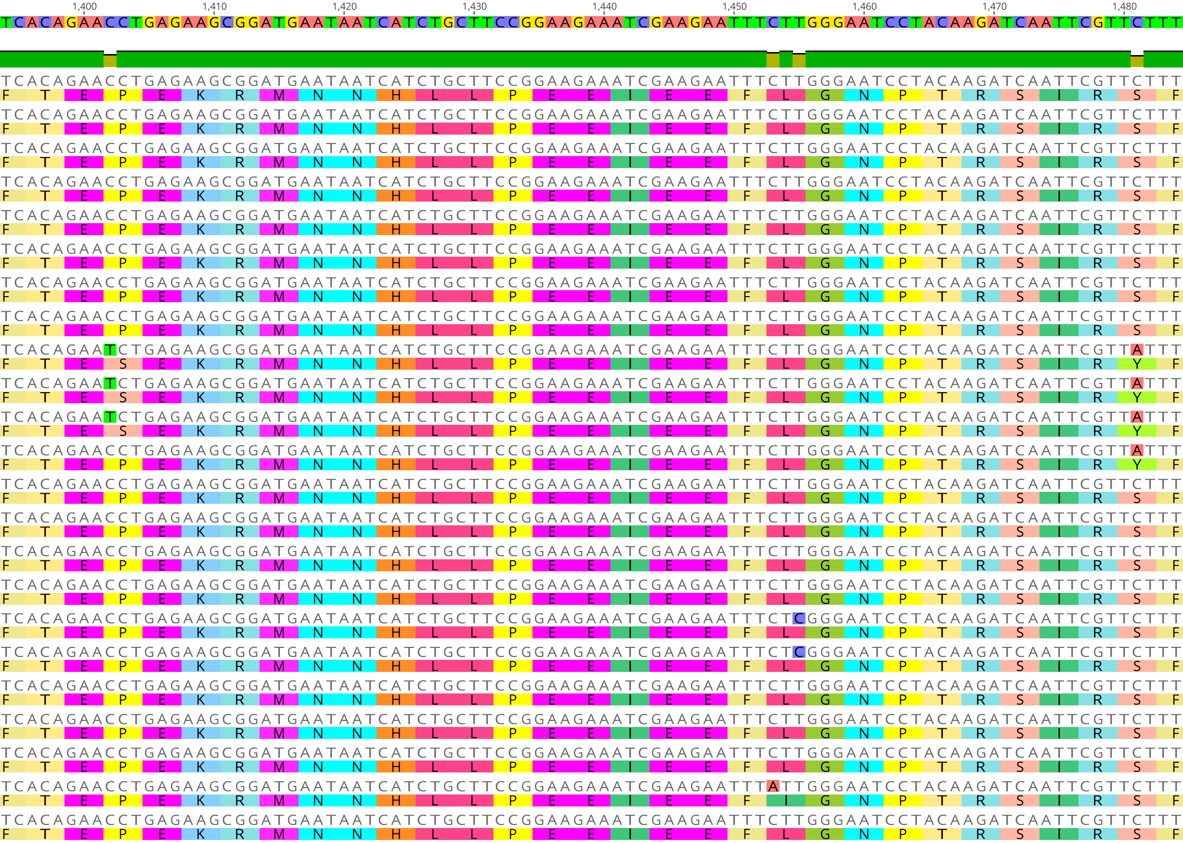
1,400 1,410 1,420 1,430 1,440 1,450 1,460 1,470 1,480
TCACAGAACCTGAGAAGCGGATGAATAATCATCTGCTTCCGGAAGAAATCGAAGAATTTCTTGGGAATCCTACAAGATCAATTCGTTCTTT
TCACAGAACCTGAGAAGCGGATGAATAATCATCTGCTTCCGGAAGAAATCGAAGAATTTCTTGGGAATCCTACAAGATCAATTCGTTCTTT
F T E P E K R M N N H L L P E E I E E F L G N P T R S I R S F
TCACAGAACCTGAGAAGCGGATGAATAATCATCTGCTTCCGGAAGAAATCGAAGAATTTCTTGGGAATCCTACAAGATCAATTCGTTCTTT
F T E P E K R M N N H L L P E E I E E F L G N P T R S I R S F
TCACAGAACCTGAGAAGCGGATGAATAATCATCTGCTTCCGGAAGAAATCGAAGAATTTCTTGGGAATCCTACAAGATCAATTCGTTCTTT
F T E P E K R M N N H L L P E E I E E F L G N P T R S I R S F
TCACAGAACCTGAGAAGCGGATGAATAATCATCTGCTTCCGGAAGAAATCGAAGAATTTCTTGGGAATCCTACAAGATCAATTCGTTCTTT
F T E P E K R M N N H L L P E E I E E F L G N P T R S I R S F
TCACAGAACCTGAGAAGCGGATGAATAATCATCTGCTTCCGGAAGAAATCGAAGAATTTCTTGGGAATCCTACAAGATCAATTCGTTCTTT
F T E P E K R M N N H L L P E E I E E F L G N P T R S I R S F
TCACAGAACCTGAGAAGCGGATGAATAATCATCTGCTTCCGGAAGAAATCGAAGAATTTCTTGGGAATCCTACAAGATCAATTCGTTCTTT
F T E P E K R M N N H L L P E E I E E F L G N P T R S I R S F
TCACAGAACCTGAGAAGCGGATGAATAATCATCTGCTTCCGGAAGAAATCGAAGAATTTCTTGGGAATCCTACAAGATCAATTCGTTCTTT
F T E P E K R M N N H L L P E E I E E F L G N P T R S I R S F
TCACAGAACCTGAGAAGCGGATGAATAATCATCTGCTTCCGGAAGAAATCGAAGAATTTCTTGGGAATCCTACAAGATCAATTCGTTCTTT
F T E P E K R M N N H L L P E E I E E F L G N P T R S I R S F
TCACAGAATCTGAGAAGCGGATGAATAATCATCTGCTTCCGGAAGAAATCGAAGAATTTCTTGGGAATCCTACAAGATCAATTCGTTATTT
F T E S E K R M N N H L L P E E I E E F L G N P T R S I R Y F
TCACAGAATCTGAGAAGCGGATGAATAATCATCTGCTTCCGGAAGAAATCGAAGAATTTCTTGGGAATCCTACAAGATCAATTCGTTATTT
F T E S E K R M N N H L L P E E I E E F L G N P T R S I R Y F
TCACAGAATCTGAGAAGCGGATGAATAATCATCTGCTTCCGGAAGAAATCGAAGAATTTCTTGGGAATCCTACAAGATCAATTCGTTATTT
F T E S E K R M N N H L L P E E I E E F L G N P T R S I R Y F
TCACAGAACCTGAGAAGCGGATGAATAATCATCTGCTTCCGGAAGAAATCGAAGAATTTCTTGGGAATCCTACAAGATCAATTCGTTATTT
F T E P E K R M N N H L L P E E I E E F L G N P T R S I R Y F
TCACAGAACCTGAGAAGCGGATGAATAATCATCTGCTTCCGGAAGAAATCGAAGAATTTCTTGGGAATCCTACAAGATCAATTCGTTCTTT
F T E P E K R M N N H L L P E E I E E F L G N P T R S I R S F
TCACAGAACCTGAGAAGCGGATGAATAATCATCTGCTTCCGGAAGAAATCGAAGAATTTCTTGGGAATCCTACAAGATCAATTCGTTCTTT
F T E P E K R M N N H L L P E E I E E F L G N P T R S I R S F
TCACAGAACCTGAGAAGCGGATGAATAATCATCTGCTTCCGGAAGAAATCGAAGAATTTCTTGGGAATCCTACAAGATCAATTCGTTCTTT
F T E P E K R M N N H L L P E E I E E F L G N P T R S I R S F
TCACAGAACCTGAGAAGCGGATGAATAATCATCTGCTTCCGGAAGAAATCGAAGAATTTCTTGGGAATCCTACAAGATCAATTCGTTCTTT
F T E P E K R M N N H L L P E E I E E F L G N P T R S I R S F
TCACAGAACCTGAGAAGCGGATGAATAATCATCTGCTTCCGGAAGAAATCGAAGAATTTCTCGGGAATCCTACAAGATCAATTCGTTCTTT
F T E P E K R M N N H L L P E E I E E F L G N P T R S I R S F
TCACAGAACCTGAGAAGCGGATGAATAATCATCTGCTTCCGGAAGAAATCGAAGAATTTCTCGGGAATCCTACAAGATCAATTCGTTCTTT
F T E P E K R M N N H L L P E E I E E F L G N P T R S I R S F
TCACAGAACCTGAGAAGCGGATGAATAATCATCTGCTTCCGGAAGAAATCGAAGAATTTCTTGGGAATCCTACAAGATCAATTCGTTCTTT
F T E P E K R M N N H L L P E E I E E F L G N P T R S I R S F
TCACAGAACCTGAGAAGCGGATGAATAATCATCTGCTTCCGGAAGAAATCGAAGAATTTCTTGGGAATCCTACAAGATCAATTCGTTCTTT
F T E P E K R M N N H L L P E E I E E F L G N P T R S I R S F
TCACAGAACCTGAGAAGCGGATGAATAATCATCTGCTTCCGGAAGAAATCGAAGAATTTCTTGGGAATCCTACAAGATCAATTCGTTCTTT
F T E P E K R M N N H L L P E E I E E F L G N P T R S I R S F
TCACAGAACCTGAGAAGCGGATGAATAATCATCTGCTTCCGGAAGAAATCGAAGAATTTATTGGGAATCCTACAAGATCAATTCGTTCTTT
F T E P E K R M N N H L L P E E I E E F I G N P T R S I R S F
TCACAGAACCTGAGAAGCGGATGAATAATCATCTGCTTCCGGAAGAAATCGAAGAATTTCTTGGGAATCCTACAAGATCAATTCGTTCTTT
F T E P E K R M N N H L L P E E I E E F L G N P T R S I R S F

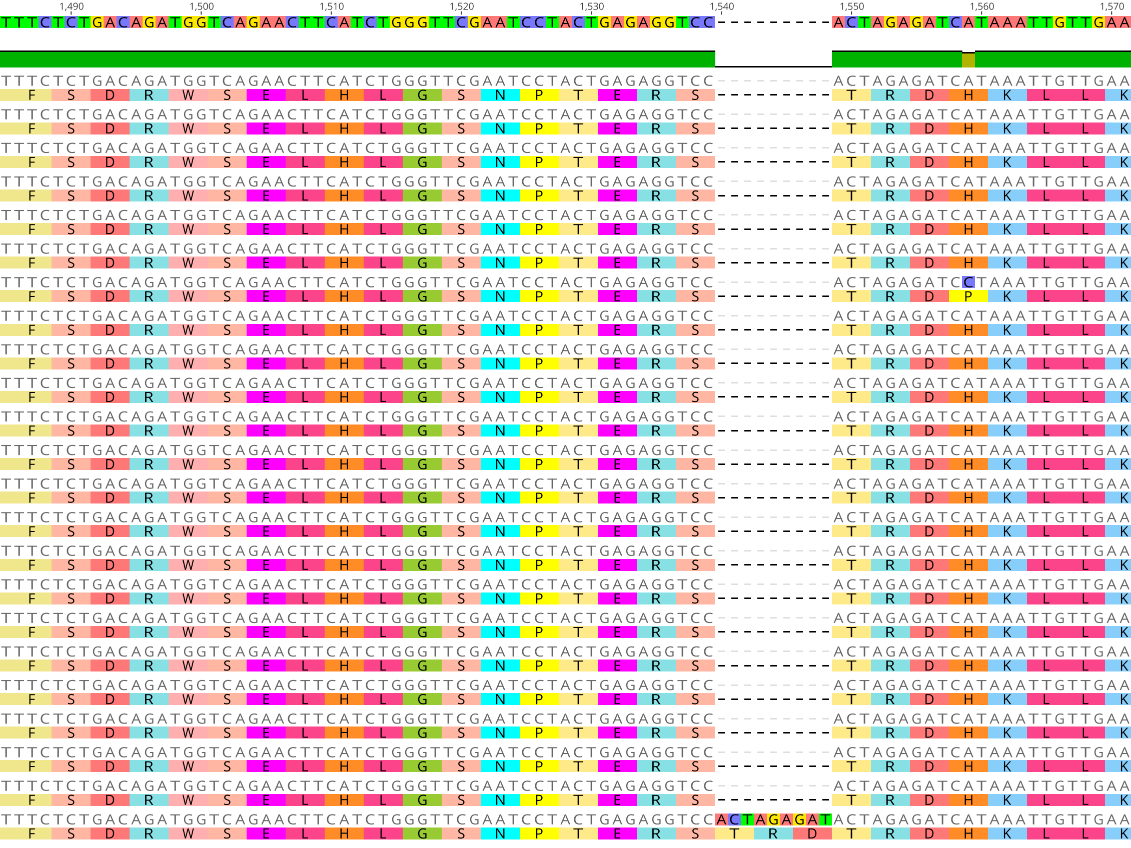
1,490 1,500 1,510 1,520 1,530 1,540 1,550 1,560 1,570
TTTCTCTGACAGATGGTCAGAACTTCATCTGGGTTCGAATCCTACTGAGAGGTCC---------ACTAGAGATCATAAATTGTTGAA
TTTCTCTGACAGATGGTCAGAACTTCATCTGGGTTCGAATCCTACTGAGAGGTCC---------ACTAGAGATCATAAATTGTTGAA
F S D R W S E L H L G S N P T E R S --------- T R D H K L L K
TTTCTCTGACAGATGGTCAGAACTTCATCTGGGTTCGAATCCTACTGAGAGGTCC---------ACTAGAGATCATAAATTGTTGAA
F S D R W S E L H L G S N P T E R S --------- T R D H K L L K
TTTCTCTGACAGATGGTCAGAACTTCATCTGGGTTCGAATCCTACTGAGAGGTCC---------ACTAGAGATCATAAATTGTTGAA
F S D R W S E L H L G S N P T E R S --------- T R D H K L L K
TTTCTCTGACAGATGGTCAGAACTTCATCTGGGTTCGAATCCTACTGAGAGGTCC---------ACTAGAGATCATAAATTGTTGAA
F S D R W S E L H L G S N P T E R S --------- T R D H K L L K
TTTCTCTGACAGATGGTCAGAACTTCATCTGGGTTCGAATCCTACTGAGAGGTCC---------ACTAGAGATCATAAATTGTTGAA
F S D R W S E L H L G S N P T E R S --------- T R D H K L L K
TTTCTCTGACAGATGGTCAGAACTTCATCTGGGTTCGAATCCTACTGAGAGGTCC---------ACTAGAGATCATAAATTGTTGAA
F S D R W S E L H L G S N P T E R S --------- T R D H K L L K
TTTCTCTGACAGATGGTCAGAACTTCATCTGGGTTCGAATCCTACTGAGAGGTCC---------ACTAGAGATCCTAAATTGTTGAA
F S D R W S E L H L G S N P T E R S --------- T R D P K L L K
TTTCTCTGACAGATGGTCAGAACTTCATCTGGGTTCGAATCCTACTGAGAGGTCC---------ACTAGAGATCATAAATTGTTGAA
F S D R W S E L H L G S N P T E R S --------- T R D H K L L K
TTTCTCTGACAGATGGTCAGAACTTCATCTGGGTTCGAATCCTACTGAGAGGTCC---------ACTAGAGATCATAAATTGTTGAA
F S D R W S E L H L G S N P T E R S --------- T R D H K L L K
TTTCTCTGACAGATGGTCAGAACTTCATCTGGGTTCGAATCCTACTGAGAGGTCC---------ACTAGAGATCATAAATTGTTGAA
F S D R W S E L H L G S N P T E R S --------- T R D H K L L K
TTTCTCTGACAGATGGTCAGAACTTCATCTGGGTTCGAATCCTACTGAGAGGTCC---------ACTAGAGATCATAAATTGTTGAA
F S D R W S E L H L G S N P T E R S --------- T R D H K L L K
TTTCTCTGACAGATGGTCAGAACTTCATCTGGGTTCGAATCCTACTGAGAGGTCC---------ACTAGAGATCATAAATTGTTGAA
F S D R W S E L H L G S N P T E R S --------- T R D H K L L K
TTTCTCTGACAGATGGTCAGAACTTCATCTGGGTTCGAATCCTACTGAGAGGTCC---------ACTAGAGATCATAAATTGTTGAA
F S D R W S E L H L G S N P T E R S --------- T R D H K L L K
TTTCTCTGACAGATGGTCAGAACTTCATCTGGGTTCGAATCCTACTGAGAGGTCC---------ACTAGAGATCATAAATTGTTGAA
F S D R W S E L H L G S N P T E R S --------- T R D H K L L K
TTTCTCTGACAGATGGTCAGAACTTCATCTGGGTTCGAATCCTACTGAGAGGTCC---------ACTAGAGATCATAAATTGTTGAA
F S D R W S E L H L G S N P T E R S --------- T R D H K L L K
TTTCTCTGACAGATGGTCAGAACTTCATCTGGGTTCGAATCCTACTGAGAGGTCC---------ACTAGAGATCATAAATTGTTGAA
F S D R W S E L H L G S N P T E R S --------- T R D H K L L K
TTTCTCTGACAGATGGTCAGAACTTCATCTGGGTTCGAATCCTACTGAGAGGTCC---------ACTAGAGATCATAAATTGTTGAA
F S D R W S E L H L G S N P T E R S --------- T R D H K L L K
TTTCTCTGACAGATGGTCAGAACTTCATCTGGGTTCGAATCCTACTGAGAGGTCC---------ACTAGAGATCATAAATTGTTGAA
F S D R W S E L H L G S N P T E R S --------- T R D H K L L K
TTTCTCTGACAGATGGTCAGAACTTCATCTGGGTTCGAATCCTACTGAGAGGTCC---------ACTAGAGATCATAAATTGTTGAA
F S D R W S E L H L G S N P T E R S --------- T R D H K L L K
TTTCTCTGACAGATGGTCAGAACTTCATCTGGGTTCGAATCCTACTGAGAGGTCC---------ACTAGAGATCATAAATTGTTGAA
F S D R W S E L H L G S N P T E R S --------- T R D H K L L K
TTTCTCTGACAGATGGTCAGAACTTCATCTGGGTTCGAATCCTACTGAGAGGTCC---------ACTAGAGATCATAAATTGTTGAA
F S D R W S E L H L G S N P T E R S --------- T R D H K L L K
TTTCTCTGACAGATGGTCAGAACTTCATCTGGGTTCGAATCCTACTGAGAGGTCC---------ACTAGAGATCATAAATTGTTGAA
F S D R W S E L H L G S N P T E R S --------- T R D H K L L K
TTTCTCTGACAGATGGTCAGAACTTCATCTGGGTTCGAATCCTACTGAGAGGTCC---------ACTAGAGATCATAAATTGTTGAA
F S D R W S E L H L G S N P T E R S --------- T R D H K L L K
TTTCTCTGACAGATGGTCAGAACTTCATCTGGGTTCGAATCCTACTGAGAGGTCCACTAGAGATACTAGAGATCATAAATTGTTGAA
F S D R W S E L H L G S N P T E R S T R D T R D H K L L K

```
1,580 1,590 1,600 1,610 1,620 1,630 1,640 1
GAAAAAACAAGATGTTTCTTTTGTCCCTTCCAGGCGAGCGGAAAAGAAAGAAATGGTTGATATATTCAAGATAATTAC

GAAAAAACAAGATGTTTCTTTTGTCCCTTCCAGGCGAGCGGAAAAGAAAGAAATGGTTGATATATTCAAGATAATTAC
K K Q D V S F V P S R R A E K K E M V D I F K I I T
GAAAAAACAAGATGTTTCTTTTGTCCCTTCCAGGCGAGCGGAAAAGAAAGAAATGGTTGATATATTCAAGATAATTAC
K K Q D V S F V P S R R A E K K E M V D I F K I I T
GAAAAAACAAGATGTTTCTTTTGTCCCTTCCAGGCGAGCGGAAAAGAAAGAAATGGTTGATATATTCAAGATAATTAC
K K Q D V S F V P S R R A E K K E M V D I F K I I T
GAAAAAACAAGATGTTTCTTTTGTCCCTTCCAGGCGAGCGGAAAAGAAAGAAATGGTTGATATATTCAAGATAATTAC
K K Q D V S F V P S R R A E K K E M V D I F K I I T
GAAAAAACAAGATGTTTCTTTTGTCCCTTCCAGGCGAGCGGAAAAGAAAGAAATGGTTGATATATTCAAGATAATTAC
K K Q D V S F V P S R R A E K K E M V D I F K I I T
GAAAAAACAAGATGTTTCTTTTGTCCCTTCCAGGCGAGCGGAAAAGAAAGAAATGGTTGATATATTCAAGATAATTAC
K K Q D V S F V P S R R A E K K E M V D I F K I I T
GAAAAAACAAGATGTTTCTTTTGTCCCTTCCAGGCGAGCGGAAAAGAAAGAAATGGTTGATATATTCAAGATAATTAC
K K Q D V S F V P S R R A E K K E M V D I F K I I T
GAAAAAACAAGATGTTTCTTTTGTCCCTTCCAGGCGAGCGGAAAAGAAAGAAATGGTTGATATATTCAAGATAATTAC
K K Q D V S F V P S R R A E K K E M V D I F K I I T
GAAAAAACAAGATGTTTCTTTTGTCCCTTCCAGGCGAGCGGAAAAGAAAGAAATGGTTGATATATTCAAGATAATTAC
K K Q D V S F V P S R R A E K K E M V D I F K I I T
GAAAAAACAAGATGTTTCTTTTGTCCCTTCCAGGCGAGCGGAAAAGAAAGAAATGGTTGATATATTCAAGATAATTAC
K K Q D V S F V P S R R A E K K E M V D I F K I I T
GAAAAAACAAGATGTTTCTTTTGTCCCTTCCAGGCGAGCGGAAAAGAAAGAAATGGTTGATATATTCAAGATAATTAC
K K Q D V S F V P S R R A E K K E M V D I F K I I T
GAAAAAACAAGATGTTTCTTTTGTCCCTTCCAGGCGAGCGGAAAAGAAAGAAATGGTTGATATATTCAAGATAATTAC
K K Q D V S F V P S R R A E K K E M V D I F K I I T
GAAAAAACAAGATGTTTCTTTTGTCCCTTCCAGGCGAGCGGAAAAGAAAGAAATGGTTGATATATTCAAGATAATTAC
K K Q D V S F V P S R R A E K K E M V D I F K I I T
GAAAAAACAAGATGTTTCTTTTGTCCCTTCCAGGCGAGCGGAAAAGAAAGAAATGGTTGATATATTCAAGATAATTAC
K K Q D V S F V P S R R A E K K E M V D I F K I I T
GAAAAAACAAGATGTTTCTTTTGTCCCTTCCAGGCGAGCGGAAAAGAAAGAAATGGTTGATATATTCAAGATAATTAC
K K Q D V S F V P S R R A E K K E M V D I F K I I T
GAAAAAACAAGATGTTTCTTTTGTCCCTTCCAGGCGAGCGGAAAAGAAAGAAATGGTTGATATATTCAAGATAATTAC
K K Q D V S F V P S R R A E K K E M V D I F K I I T
GAAAAAACAAGATGTTTCTTTTGTCCCTTCCAGGCGAGCGGAAAAGAAAGAAATGGTTGATATATTCAAGATAATTAC
K K Q D V S F V P S R R A E K K E M V D I F K I I T
GAAAAAACAAGATGTTTCTTTTGTCCCTTCCAGGCGAGCGGAAAAGAAAGAAATGGTTGATATATTCAAGATAATTAC
K K Q D V S F V P S R R A E K K E M V D I F K I I T
GAAAAAACAAGATGTTTCTTTTGTCCCTTCCAGGCGAGCGGAAAAGAAAGAAATGGTTGATATATTCAAGATAATTAC
K K Q D V S F V P S R R A E K K E M V D I F K I I T
GAAAAAACAAGATGTTTCTTTTGTCCCTTCCAGGCGAGCGGAAAAGAAAGAAATGGTTGATATATTCAAGATAATTAC
K K Q D V S F V P S R R A E K K E M V D I F K I I T
GAAAAAACAAGATGTTTCTTTTGTCCCTTCCAGGCGAGCGGAAAAGAAAGAAATGGTTGATATATTCAAGATAATTAC
K K Q D V S F V P S R R A E K K E M V D I F K I I T
GAAAAAACAAGATGTTTCTTTTGTCCCTTCCAGGCGAGCGGAAAAGAAAGAAATGGTTGATATATTCAAGATAATTAC
K K Q D V S F V P S R R A E K K E M V D I F K I I T
GAAAAAACAAGATGTTTCTTTTGTCCCTTCCAGGCGAGCGGAAAAGAAAGAAATGGTTGATATATTCAAGATAATTAC
K K Q D V S F V P S R R A E K K E M V D I F K I I T
```

```
,650      1,660     1,670     1,680     1,690     1,700     1,710     1,720
GTATTTACAAGATACCGTCTCAATTCATCCTTCGGAACCATATCACATCCCGAATCTGGTTCCGAAGGATGAACC

GTATTTACAAGATACCGTCTCAATTCATCCTTCGGAACCATATCACATCCCGAATCTGGTTCCGAAGGATGAACC
 Y  L  Q  D  T  V  S  I  H  P  S  E  P  Y  H  I  P  N  L  V  P  K  D  E  P
GTATTTACAAGATACCGTCTCAATTCATCCTTCGGAACCATATCACATCCCGAATCTGGTTCCGAAGGATGAACC
 Y  L  Q  D  T  V  S  I  H  P  S  E  P  Y  H  I  P  N  L  V  P  K  D  E  P
GTATTTACAAGATACCGTCTCAATTCATCCTTCGGAACCATATCACATCCCGAATCTGGTTCCGAAGGATGAACC
 Y  L  Q  D  T  V  S  I  H  P  S  E  P  Y  H  I  P  N  L  V  P  K  D  E  P
GTATTTACAAGATACCGTCTCAATTCATCCTTCGGAACCATATCACATCCCGAATCTGGTTCCGAAGGATGAACC
 Y  L  Q  D  T  V  S  I  H  P  S  E  P  Y  H  I  P  N  L  V  P  K  D  E  P
GTATTTACAAGATACCGTCTCAATTCATCCTTCGGAACCATATCACATCCCGAATCTGGTTCCGAAGGATGAACC
 Y  L  Q  D  T  V  S  I  H  P  S  E  P  Y  H  I  P  N  L  V  P  K  D  E  P
GTATTTACAAGATACCGTCTCAATTCATCCTTCGGAACCATATCACATCCCGAATCTGGTTCCGAAGGATGAACC
 Y  L  Q  D  T  V  S  I  H  P  S  E  P  Y  H  I  P  N  L  V  P  K  D  E  P
GTATTTACAAGATACCGTCTCAATTCATCCTTCGGAACCATATCACATCCCGAATCTGGTTCCGAAGGATGAACC
 Y  L  Q  D  T  V  S  I  H  P  S  E  P  Y  H  I  P  N  L  V  P  K  D  E  P
GTATTTACAAGATACCGTCTCAATTCATCCTTCGGAACCATATCACATCCCGAATCTGGTTCCGAAGGATGAACC
 Y  L  Q  D  T  V  S  I  H  P  S  E  P  Y  H  I  P  N  L  V  P  K  D  E  P
GTATTTACAAGATACCGTCTCAATTCATCCTTCGGAACCATATCACATCCCGAATCTGGTTCCGAAGGATGAACC
 Y  L  Q  D  T  V  S  I  H  P  S  E  P  Y  H  I  P  N  L  V  P  K  D  E  P
GTATTTACAAGATACCGTCTCAATTCATCCTTCGGAACCATATCACATCCCGAATCTGGTTCCGAAGGATGAACC
 Y  L  Q  D  T  V  S  I  H  P  S  E  P  Y  H  I  P  N  L  V  P  K  D  E  P
GTATTTACAAGATACCGTCTCAATTCATCCTTCGGAACCATATCACATCCCGAATCTGGTTCCGAAGGATGAACC
 Y  L  Q  D  T  V  S  I  H  P  S  E  P  Y  H  I  P  N  L  V  P  K  D  E  P
GTATTTACAAGATACCGTCTCAATTCATCCTTCGGAACCATATCACATCCCGAATCTGGTTCCGAAGGATGAACC
 Y  L  Q  D  T  V  S  I  H  P  S  E  P  Y  H  I  P  N  L  V  P  K  D  E  P
GTATTTACAAGATACCGTCTCAATTCATCCTTCGGAACCATATCACATCCCGAATCTGGTTCCGAAGGATGAACC
 Y  L  Q  D  T  V  S  I  H  P  S  E  P  Y  H  I  P  N  L  V  P  K  D  E  P
GTATTTACAAGATACCGTCTCAATTCATCCTTCGGAACCATATCACATCCCGAATCTGGTTCCGAAGGATGAACC
 Y  L  Q  D  T  V  S  I  H  P  S  E  P  Y  H  I  P  N  L  V  P  K  D  E  P
GTATTTACAAGATACCGTCTCAATTCATCCTTCGGAACCATATCACATCCCGAATCTGGTTCCGAAGGATGAACC
 Y  L  Q  D  T  V  S  I  H  P  S  E  P  Y  H  I  P  N  L  V  P  K  D  E  P
GTATTTACAAGATACCGTCTCAATTCATCCTTCGGAACCATATCACATCCCGAATCTGGTTCCGAAGGATGAACC
 Y  L  Q  D  T  V  S  I  H  P  S  E  P  Y  H  I  P  N  L  V  P  K  D  E  P
GTATTTACAAGATACCGTCTCAATTCATCCTTCGGAACCATATCACATCCCGAATCTGGTTCCGAAGGATGAACC
 Y  L  Q  D  T  V  S  I  H  P  S  E  P  Y  H  I  P  N  L  V  P  K  D  E  P
GTATTTACAAGATACCGTCTCAATTCATCCTTCGGAACCATATCACATCCCGAATCTGGTTCCGAAGGATGAACC
 Y  L  Q  D  T  V  S  I  H  P  S  E  P  Y  H  I  P  N  L  V  P  K  D  E  P
GTATTTACAAGATACCGTCTCAATTCATCCTTCGGAACCATATCACATCCCGAATCTGGTTCCGAAGGATGAACC
 Y  L  Q  D  T  V  S  I  H  P  S  E  P  Y  H  I  P  N  L  V  P  K  D  E  P
GTATTTACAAGATACCGTCTCAATTCATCCTTCGGAACCATATCACATCCCGAATCTGGTTCCGAAGGATGAACC
 Y  L  Q  D  T  V  S  I  H  P  S  E  P  Y  H  I  P  N  L  V  P  K  D  E  P
GTATTTACAAGATACCGTCTCAATTCATCCTTCGGAACCATATCACATCCCGAATCTGGTTCCGAAGGATGAACC
 Y  L  Q  D  T  V  S  I  H  P  S  E  P  Y  H  I  P  N  L  V  P  K  D  E  P
GTATTTACAAGATACCGTCTCAATTCATCCTTCGGAACCATATCACATCCCGAATCTGGTTCCGAAGGATGAACC
 Y  L  Q  D  T  V  S  I  H  P  S  E  P  Y  H  I  P  N  L  V  P  K  D  E  P
GTATTTACAAGATACCGTCTCAATTCATCCTTCGGAACCATATCACATCCCGAATCTGGTTCCGAAGGATGAACC
 Y  L  Q  D  T  V  S  I  H  P  S  E  P  Y  H  I  P  N  L  V  P  K  D  E  P
```

1,730 1,740 1,750 1,760 1,770 1,780 1,790 1,800 1,810

GGATATGGACAGTTCCAATAAGATTTCATTCTTGAACAAAAATCCATTTTTTGATTTCTTTCATCTATTCCATGACCGGAACAAAGGGG

GGATATGGACAGTTCCAATAAGATTTCATTCTTGAACAAAAATCCATTTTTTGATTTCTTTCATCTATTCCATGACCGGAACAAAGGGG
D M D S S N K I S F L N K N P F F D F F H L F H D R N K G

GGATATGGACAGTTCCAATAAGATTTCATTCTTGAACAAAAATCCATTTTTTGATTTCTTTCATCTATTCCATGACCGGAACAAAGGGG
D M D S S N K I S F L N K N P F F D F F H L F H D R N K G

GGATATGGACAGTTCCAATAAGATTTCATTCTTGAACAAAAATCCATTTTTTGATTTCTTTCATCTATTCCATGACCGGAACAAAGGGG
D M D S S N K I S F L N K N P F F D F F H L F H D R N K G

GGATATGGACAGTTCCAATAAGATTTCATTCTTGAACAAAAATCCATTTTTTGATTTCTTTCATCTATTCCATGACCGGAACAAAGGGG
D M D S S N K I S F L N K N P F F D F F H L F H D R N K G

GGATATGGACAGTTCCAATAAGATTTCATTCTTGAACAAAAATCCATTTTTTGATTTCTTTCATCTATTCCATGACCGGAACAAAGGGG
D M D S S N K I S F L N K N P F F D F F H L F H D R N K G

GGATATGGACAGTTCCAATAAGATTTCATTCTTGAACAAAAATCCATTTTTTGATTTCTTTCATCTATTCCATGACCGGAACAAAGGGG
D M D S S N K I S F L N K N P F F D F F H L F H D R N K G

GGATATGGACAGTTCCAATAAGATTTCATTCTTGAACAAAAATCCATTTTTTGATTTCTTTCATCTATTCCATGACCGGAACAAAGGGG
D M D S S N K I S F L N K N P F F D F F H L F H D R N K G

GGATATGGACAGTTCCAATAAGATTTCATTCTTGAACAAAAATCCATTTTTTGATTTCTTTCATCTATTCCATGACCGGAACAAAGGGG
D M D S S N K I S F L N K N P F F D F F H L F H D R N K G

GGATATGGACAGTTCCAATAAGATTTCATTCTTGAACAAAAATCCATTTTTTGATTTCTTTCATCTATTCCATGACCGGAACAAAGGGG
D M D S S N K I S F L N K N P F F D F F H L F H D R N K G

GGATATGGACAGTTCCAATAAGATTTCATTCTTGAACAAAAATCCATTTTTTGATTTCTTTCATCTATTCCATGACCGGAACAAAGGGG
D M D S S N K I S F L N K N P F F D F F H L F H D R N K G

GGATATGGACAGTTCCAATAAGATTTCATTCTTGAACAAAAATCCATTTTTTGATTTCTTTCATCTATTCCATGACCGGAACAAAGGGG
D M D S S N K I S F L N K N P F F D F F H L F H D R N K G

GGATATGGACAGTTCCAATAAGATTTCATTCTTGAACAAAAATCCATTTTTTGATTTCTTTCATCTATTCCATGACCGGAACAAAGGGG
D M D S S N K I S F L N K N P F F D F F H L F H D R N K G

GGATATGGACAGTTCCAATAAGATTTCATTCTTGAACAAAAATCCATTTTTTGATTTCTTTCATCTATTCCATGACCGGAACAAAGGGG
D M D S S N K I S F L N K N P F F D F F H L F H D R N K G

GGATATGGACAGTTCCAATAAGATTTCATTCTTGAACAAAAATCCATTTTTTGATTTCTTTCATCTATTCCATGACCGGAACAAAGGGG
D M D S S N K I S F L N K N P F F D F F H L F H D R N K G

GGATATGGACAGTTCCAATAAGATTTCATTCTTGAACAAAAATCCATTTTTTGATTTCTTTCATCTATTCCATGACCGGAACAAAGGGG
D M D S S N K I S F L N K N P F F D F F H L F H D R N K G

GGATATGGACAGTTCCAATAAGATTTCATTCTTGAACAAAAATCCATTTTTTGATTTCTTTCATCTATTCCATGACCGGAACAAAGGGG
D M D S S N K I S F L N K N P F F D F F H L F H D R N K G

GGATATGGACAGTTCCAATAAGATTTCATTCTTGAACAAAAATCCATTTTTTGATTTCTTTCATCTATTCCATGACCGGAACAAAGGGG
D M D S S N K I S F L N K N P F F D F F H L F H D R N K G

GGATATGGACAGTTCCAATAAGATTTCATTCTTGAACAAAAATCCATTTTTTGATTTCTTTCATCTATTCCATGACCGGAACAAAGGGG
D M D S S N K I S F L N K N P F F D F F H L F H D R N K G

GGATATGGACAGTTCCAATAAGATTTCATTCTTGAACAAAAATCCATTTTTTGATTTCTTTCATCTATTCCATGACCGGAACAAAGGGG
D M D S S N K I S F L N K N P F F D F F H L F H D R N K G

GGATATGGACAGTTCCAATAAGATTTCATTCTTGAACAAAAATCCATTTTTTGATTTCTTTCATCTATTCCATGACCGGAACAAAGGGG
D M D S S N K I S F L N K N P F F D F F H L F H D R N K G

GGATATGGACAGTTCCAATAAGATTTCATTCTTGAACAAAAATCCATTTTTTGATTTCTTTCATCTATTCCATGACCGGAACAAAGGGG
D M D S S N K I S F L N K N P F F D F F H L F H D R N K G

GGATATGGACAGTTCCAATAAGATTTCATTCTTGAACAAAAATCCATTTTTTGATTTCTTTCATCTATTCCATGACCGGAACAAAGGGG
D M D S S N K I S F L N K N P F F D F F H L F H D R N K G

GGATATGGACAGTTCCAATAAGATTTCATTCTTGAACAAAAATCCATTTTTTGATTTCTTTCATCTATTCCATGACCGGAACAAAGGGG
D M D S S N K I S F L N K N P F F D F F H L F H D R N K G

```
1,820 1,830 1,840 1,850 1,860 1,870 1,880 1,890
GATACGCGTTACGCCACGATTTTTTTGAATCAGAAGAGAGATTCCCAGAAATGGCGGATCTATTCACTCTATCAATAACCGAGCC

GATACGCGTTACGCCACGATTTTTTTGAATCAGAAGAGAGATTCCCAGAAATGGCGGATCTATTCACTCTATCAATAACCGAGCC
G Y A L R H D F F E S E E R F P E M A D L F T L S I T E P
GATACGCGTTACGCCACGATTTTTTTGAATCAGAAGAGAGATTCCCAGAAATGGCGGATCTATTCACTCTATCAATAACCGAGCC
G Y A L R H D F F E S E E R F P E M A D L F T L S I T E P
GATACGCGTTACGCCACGATTTTTTTGAATCAGAAGAGAGATTCCCAGAAATGGCGGATCTATTCACTCTATCAATAACCGAGCC
G Y A L R H D F F E S E E R F P E M A D L F T L S I T E P
GATACGCGTTACGCCACGATTTTTTTGAATCAGAAGAGAGATTCCCAGAAATGGCGGATCTATTCACTCTATCAATAACCGAGCC
G Y A L R H D F F E S E E R F P E M A D L F T L S I T E P
GATACGCGTTACGCCACGATTTTTTTGAATCAGAAGAGAGATTCCCAGAAATGGCGGATCTATTCACTCTATCAATAACCGAGCC
G Y A L R H D F F E S E E R F P E M A D L F T L S I T E P
GATACGCGTTACGCCACGATTTTTTTGAATCAGAAGAGAGATTCCCAGAAATGGCGGATCTATTCACTCTATCAATAACCGAGCC
G Y A L R H D F F E S E E R F P E M A D L F T L S I T E P
GATACGCGTTACGCCACGATTTTTTTGAATCAGAAGAGAGATTCCCAGAAATGGCGGATCTATTCACTCTATCAATAACCGAGCC
G Y A L R H D F F E S E E R F P E M A D L F T L S I T E P
GATACGCGTTACGCCACGATTTTTTTGAATCAGAAGAGAGATTCCCAGAAATGGCGGATCTATTCACTCTATCAATAACCGAGCC
G Y A L R H D F F E S E E R F P E M A D L F T L S I T E P
GATACGCGTTACGCCACGATTTTTTTGAATCAGAAGAGAGATTCCCAGAAATGGCGGATCTATTCACTCTATCAATAACCGAGCC
G Y A L R H D F F E S E E R F P E M A D L F T L S I T E P
GATACGCGTTACGCCACGATTTTTTTGAATCAGAAGAGAGATTCCCAGAAATGGCGGATCTATTCACTCTATCAATAACCGAGCC
G Y A L R H D F F E S E E R F P E M A D L F T L S I T E P
GATACGCGTTACGCCACGATTTTTTTGAATCAGAAGAGAGATTCCCAGAAATGGCGGATCTATTCACTCTATCAATAACCGAGCC
G Y A L R H D F F E S E E R F P E M A D L F T L S I T E P
GATACGCGTTACGCCACGATTTTTTTGAATCAGAAGAGAGATTCCCAGAAATGGCGGATCTATTCACTCTATCAATAACCGAGCC
G Y A L R H D F F E S E E R F P E M A D L F T L S I T E P
GATACGCGTTACGCCACGATTTTTTTGAATCAGAAGAGAGATTCCCAGAAATGGCGGATCTATTCACTCTATCAATAACCGAGCC
G Y A L R H D F F E S E E R F P E M A D L F T L S I T E P
GATACGCGTTACGCCACGATTTTTTTGAATCAGAAGAGAGATTACCAGAAATGGCGGATCTATTCACTCTATCAATAACCGAGCC
G Y A L R H D F F E S E E R L P E M A D L F T L S I T E P
GATACGCGTTACGCCACGATTTTTTTGAATCAGAAGAGAGATTCCCAGAAATGGCGGATCTATTCACTCTATCAATAACCGAGCC
G Y A L R H D F F E S E E R F P E M A D L F T L S I T E P
GATACGCGTTACGCCACGATTTTTTTGAATCAGAAGAGAGATTCCCAGAAATGGCGGATCTATTCACTCTATCAATAACCGAGCC
G Y A L R H D F F E S E E R F P E M A D L F T L S I T E P
GATACGCGTTACGCCACGATTTTTTTGAATCAGAAGAGAGATTCCCAGAAATGGCGGATCTATTCACTCTATCAATAACCGAGCC
G Y A L R H D F F E S E E R F P E M A D L F T L S I T E P
GATACGCGTTACGCCACGATTTTTTTGAATCAGAAGAGAGATTCCCAGAAATGGCGGATCTATTCACTCTATCAATAACCGAGCC
G Y A L R H D F F E S E E R F P E M A D L F T L S I T E P
GATACGCGTTACGCCACGATTTTTTTGAATCAGAAGAGAGATTCCCAGAAATGGCGGATCTATTCACTCTATCAATAACCGAGCC
G Y A L R H D F F E S E E R F P E M A D L F T L S I T E P
GATACGCGTTACGCCACGATTTTTTTGAATCAGAAGAGAGATTCCCAGAAATGGCGGATCTATTCACTCTATCAATAACCGAGCC
G Y A L R H D F F E S E E R F P E M A D L F T L S I T E P
GATACGCGTTACGCCACGATTTTTTTGAATCAGAAGAGAGATTCCCAGAAATGGCGGATCTATTCACTCTATCAATAACCGAGCC
G Y A L R H D F F E S E E R F P E M A D L F T L S I T E P
GATACGCGTTACGCCACGATTTTTTTGAATCAGAAGAGAGATTCCCAGAAATGGCGGATCTATTCACTCTATCAATAACCGAGCC
G Y A L R H D F F E S E E R F P E M A D L F T L S I T E P
GATACGCGTTACGCCACGATTTTTTTGAATCAGAAGAGAGATTCCCAGAAATGGCGGATCTATTCACTCTATCAATAACCGAGCC
G Y A L R H D F F E S E E R F P E M A D L F T L S I T E P
```

```
1,900     1,910     1,920     1,930     1,940     1,950     1,960     1,970     1
GGATCTGGTGTTTCATAGGGGATTTGCCTTTTCTATTGATTCCTACGGGTTGGATCAAAAAAGATTCTTGAATGAGGTATT

GGGTCTGGTGTTTCATAGGGGATTTGCCTTTTCTATTGATTCCTACGGGTTGGATCAAAAAAGATTCTTGAATGAGGTATT
 G  L  V  F  H  R  G  F  A  F  S  I  D  S  Y  G  L  D  Q  K  R  F  L  N  E  V  F
GGGTCTGGTGTTTCATAGGGGATTTGCCTTTTCTATTGATTCCTACGGGTTGGATCAAAAAAGATTCTTGAATGAGGTATT
 G  L  V  F  H  R  G  F  A  F  S  I  D  S  Y  G  L  D  Q  K  R  F  L  N  E  V  F
GGGTCTGGTGTTTCATAGGGGATTTGCCTTTTCTATTGATTCCTACGGGTTGGATCAAAAAAGATTCTTGAATGAGGTATT
 G  L  V  F  H  R  G  F  A  F  S  I  D  S  Y  G  L  D  Q  K  R  F  L  N  E  V  F
GGGTCTGGTGTTTCATAGGGGATTTGCCTTTTCTATTGATTCCTACGGGTTGGATCAAAAAAGATTCTTGAATGAGGTATT
 G  L  V  F  H  R  G  F  A  F  S  I  D  S  Y  G  L  D  Q  K  R  F  L  N  E  V  F
GGGTCTGGTGTTTCATAGGGGATTTGCCTTTTCTATTGATTCCTACGGGTTGGATCAAAAAAGATTCTTGAATGAGGTATT
 G  L  V  F  H  R  G  F  A  F  S  I  D  S  Y  G  L  D  Q  K  R  F  L  N  E  V  F
GGATCTGGTGTTTCATAGGGGATTTGCCTTTTCTATTGATTCCTACGGGTTGGATCAAAAAAGATTCTTGAATGAGGTATT
 D  L  V  F  H  R  G  F  A  F  S  I  D  S  Y  G  L  D  Q  K  R  F  L  N  E  V  F
GGATCTGGTGTTTCATAGGGGATTTGCCTTTTCTATTGATTCCTACGGGTTGGATCAAAAAAGATTCTTGAATGAGGTATT
 D  L  V  F  H  R  G  F  A  F  S  I  D  S  Y  G  L  D  Q  K  R  F  L  N  E  V  F
GGATCTGGTGTTTCATAGGGGATTTGCCTTTTCTATTGATTCCTACGGGTTGGATCAAAAAAGATTCTTGAATGAGGTATT
 D  L  V  F  H  R  G  F  A  F  S  I  D  S  Y  G  L  D  Q  K  R  F  L  N  E  V  F
GGATCTGGTGTTTCATAGGGGATTTGCCTTTTCTATTGATTCCTACGGGTTGGATCAAAAAAGATTCTTGAATGAGGTATT
 D  L  V  F  H  R  G  F  A  F  S  I  D  S  Y  G  L  D  Q  K  R  F  L  N  E  V  F
GGATCTGGTGTTTCATAGGGGATTTGCCTTTTCTATTGATTCCTACGGGTTGGATCAAAAAAGATTCTTGAATGAGGTATT
 D  L  V  F  H  R  G  F  A  F  S  I  D  S  Y  G  L  D  Q  K  R  F  L  N  E  V  F
GGATCTGGTGTTTCATAGGGGATTTGCCTTTTCTATTGATTCCTACGGGTTGGATCAAAAAAGATTCTTGAATGAGGTATT
 D  L  V  F  H  R  G  F  A  F  S  I  D  S  Y  G  L  D  Q  K  R  F  L  N  E  V  F
GGATCTGGTGTTTCATAGGGGATTTGCCTTTTCTATTGATTCCTACGGGTTGGATCAAAAAAGATTCTTGAATGAGGTATT
 D  L  V  F  H  R  G  F  A  F  S  I  D  S  Y  G  L  D  Q  K  R  F  L  N  E  V  F
GGATCTGGTGTTTCATAGGGGATTTGCCTTTTCTATTGATTCCTACGGGTTGGATCAAAAAAGATTCTTGAATGAGGTATT
 D  L  V  F  H  R  G  F  A  F  S  I  D  S  Y  G  L  D  Q  K  R  F  L  N  E  V  F
GGATCTGGTGTTTCATAGGGGATTTGCCTTTTCTATTGATTCCTACGGGTTGGATCAAAAAAGATTCTTGAATGAGGTATT
 D  L  V  F  H  R  G  F  A  F  S  I  D  S  Y  G  L  D  Q  K  R  F  L  N  E  V  F
GGATCTGGTGTTTCATAGGGGATTTGCCTTTTCTATTGATTCCTACGGGTTGGATCAAAAAAGATTCTTGAATGAGGTATT
 D  L  V  F  H  R  G  F  A  F  S  I  D  S  Y  G  L  D  Q  K  R  F  L  N  E  V  F
GGATCTGGTGTTTCATAGGGGATTTGCCTTTTCTATTGATTCCTACGGGTTGGATCAAAAAAGATTCTTGAATGAGGTATT
 D  L  V  F  H  R  G  F  A  F  S  I  D  S  Y  G  L  D  Q  K  R  F  L  N  E  V  F
GGATCTGGTGTTTCATAGGGGATTTGCCTTTTCTATTGATTCCTACGGGTTGGATCAAAAAAGATTCTTGAATGAGGTATT
 D  L  V  F  H  R  G  F  A  F  S  I  D  S  Y  G  L  D  Q  K  R  F  L  N  E  V  F
GGATCTGGTGTTTCATAGGGGATTTGCCTTTTCTATTGATTCCTACGGGTTGGATCAAAAAAGATTCTTGAATGAGGTATT
 D  L  V  F  H  R  G  F  A  F  S  I  D  S  Y  G  L  D  Q  K  R  F  L  N  E  V  F
GGATCTGGTGTTTCATAGGGGATTTGCCTTTTCTATTGATTCCTACGGGTTGGATCAAAAAAGATTCTTGAATGAGGTATT
 D  L  V  F  H  R  G  F  A  F  S  I  D  S  Y  G  L  D  Q  K  R  F  L  N  E  V  F
GGATCTGGTGTTTCATAGGGGATTTGCCTTTTCTATTGATTCCTACGGGTTGGATCAAAAAAGATTCTTGAATGAGGTATT
 D  L  V  F  H  R  G  F  A  F  S  I  D  S  Y  G  L  D  Q  K  R  F  L  N  E  V  F
GGATCTGGTGTTTCATAGGGGATTTGCCTTTTCTATTGATTCCTACGGGTTGGATCAAAAAAGATTCTTGAATGAGGTATT
 D  L  V  F  H  R  G  F  A  F  S  I  D  S  Y  G  L  D  Q  K  R  F  L  N  E  V  F
GGATCTGGTGTTTCATAGGGGATTTGCCTTTTCTATTGATTCCTACGGGTTGGATCAAAAAAGATTCTTGAATGAGGTATT
 D  L  V  F  H  R  G  F  A  F  S  I  D  S  Y  G  L  D  Q  K  R  F  L  N  E  V  F
GGATCTGGTGTTTCATAGGGGATTTGCCTTTTCTATTGATTCCTACGGGTTGGATCAAAAAAGATTCTTGAATGAGGTATT
 D  L  V  F  H  R  G  F  A  F  S  I  D  S  Y  G  L  D  Q  K  R  F  L  N  E  V  F
```

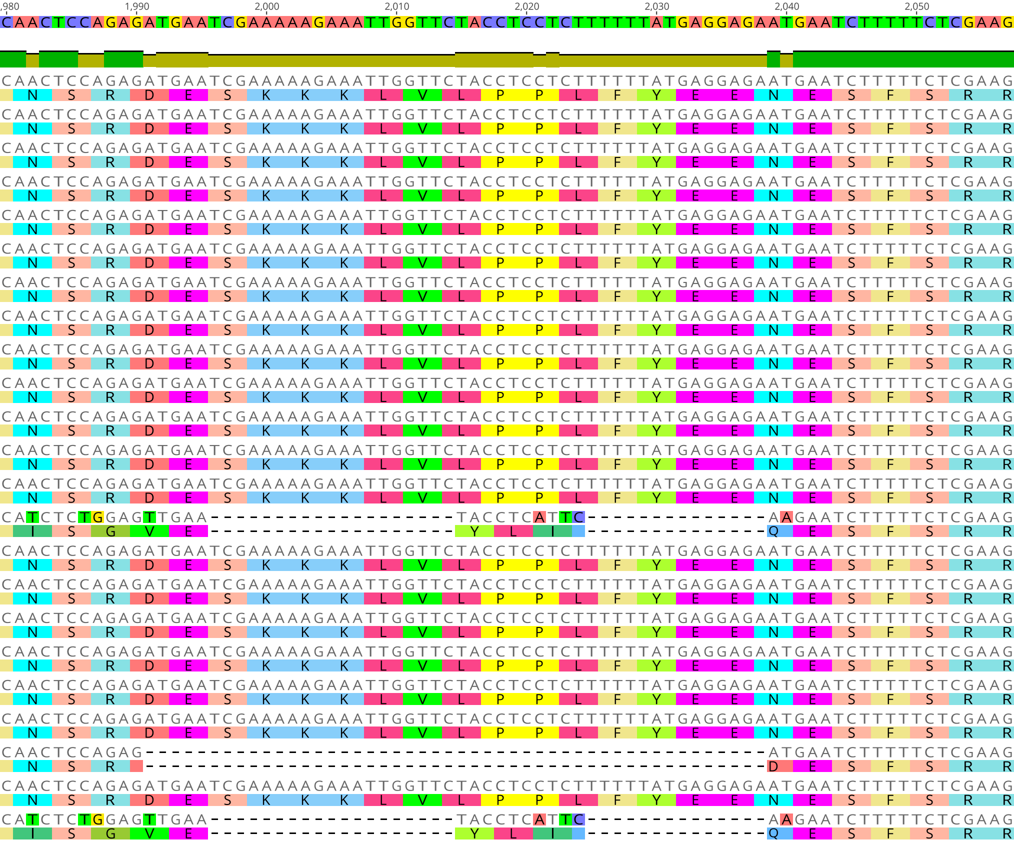

1,980 1,990 2,000 2,010 2,020 2,030 2,040 2,050
CAACTCCAGAGATGAATCGAAAAAGAAATTGGTTCTACCTCCTCTTTTTTATGAGGAGAATGAATCTTTTTCTCGAAG

```
2,060     2,070     2,080     2,090     2,100     2,110     2,120     2,130     2,140
GATCAGAAAAAAATCGGTCCGGATCTACTGCGGGAATGAGTTGGAAGATCCCAAACTAAAAACAGCGGTATTTGCTAGCAACAACATAATG

GATCAGAAAAAAATCGGTCCGGATCTACTGCGGGAATGAGTTGGAAGATCCCAAACTAAAAACAGCGGTATTTGCTAGCAACAACATAATG
 I  R  K  K  S  V  R  I  Y  C  G  N  E  L  E  D  P  K  L  K  T  A  V  F  A  S  N  N  I  M
GATCAGAAAAAAATCGGTCCGGATCTACTGCGGGAATGAGTTGGAAGATCCCAAACTAAAAACAGCGGTATTTGCTAGCAACAACATAATG
 I  R  K  K  S  V  R  I  Y  C  G  N  E  L  E  D  P  K  L  K  T  A  V  F  A  S  N  N  I  M
GATCAGAAAAAAATCGGTCCGGATCTACTGCGGGAATGAGTTGGAAGATCCCAAACTAAAAACAGCGGTATTTGCTAGCAACAACATAATG
 I  R  K  K  S  V  R  I  Y  C  G  N  E  L  E  D  P  K  L  K  T  A  V  F  A  S  N  N  I  M
GATCAGAAAAAAATCGGTCCGGATCTACTGCGGGAATGAGTTGGAAGATCCCAAACTAAAAACAGCGGTATTTGCTAGCAACAACATAATG
 I  R  K  K  S  V  R  I  Y  C  G  N  E  L  E  D  P  K  L  K  T  A  V  F  A  S  N  N  I  M
GATCAGAAAAAAATCGGTCCGGATCTACTGCGGGAATGAGTTGGAAGATCCCAAACTAAAAACAGCGGTATTTGCTAGCAACAACATAATG
 I  R  K  K  S  V  R  I  Y  C  G  N  E  L  E  D  P  K  L  K  T  A  V  F  A  S  N  N  I  M
GATCAGAAAAAAATCGGTCCGGATCTACTGCGGGAATGAGTTGGAAGATCCCAAACTAAAAACAGCGGTATTTGCTAGCAACAACATAATG
 I  R  K  K  S  V  R  I  Y  C  G  N  E  L  E  D  P  K  L  K  T  A  V  F  A  S  N  N  I  M
GATCAGAAAAAAATCGGTCCGGATCTACTGCGGGAATGAGTTGGAAGATCCCAAACTAAAAACAGCGGTATTTGCTAGCAACAACATAATG
 I  R  K  K  S  V  R  I  Y  C  G  N  E  L  E  D  P  K  L  K  T  A  V  F  A  S  N  N  I  M
GATCAGAAAAAAATCGGTCCGGATCTACTGCGGGAATGAGTTGGAAGATCCCAAACTAAAAACAGCGGTATTTGCTAGCAACAACATAATG
 I  R  K  K  S  V  R  I  Y  C  G  N  E  L  E  D  P  K  L  K  T  A  V  F  A  S  N  N  I  M
GATCAGAAAAAAATCGGTCCGGATCTACTGCGGGAATGAGTTGGAAGATCCCAAACTAAAAACAGCGGTATTTGCTAGCAACAACATAATG
 I  R  K  K  S  V  R  I  Y  C  G  N  E  L  E  D  P  K  L  K  T  A  V  F  A  S  N  N  I  M
GATCAGAAAAAAATCGGTCCGGATCTACTGCGGGAATGAGTTGGAAGATCCCAAACTAAAAACAGCGGTATTTGCTAGCAACAACATAATG
 I  R  K  K  S  V  R  I  Y  C  G  N  E  L  E  D  P  K  L  K  T  A  V  F  A  S  N  N  I  M
GATCAGAAAAAAATCGGTCCGGATCTACTGCGGGAATGAGTTGGAAGATCCCAAACTAAAAACAGCGGTATTTGCTAGCAACAACATAATG
 I  R  K  K  S  V  R  I  Y  C  G  N  E  L  E  D  P  K  L  K  T  A  V  F  A  S  N  N  I  M
GATCAGAAAAAAATCGGTCCGGATCTACTGCGGGAATGAGTTGGAAGATCCCAAACTAAAAACAGCGGTATTTGCTAGCAACAACATAATG
 I  R  K  K  S  V  R  I  Y  C  G  N  E  L  E  D  P  K  L  K  T  A  V  F  A  S  N  N  I  M
GATCAGAAAAAAATCGGTCCGGATCTACTGCGGGAATGAGTTGGAAGATCCCAAACTAAAAACAGCGGTATTTGCTAGCAACAACATAATG
 I  R  K  K  S  V  R  I  Y  C  G  N  E  L  E  D  P  K  L  K  T  A  V  F  A  S  N  N  I  M
GATCAGAAAAAAATTGGTCCGGATCTACTGCGGGAATGAGTTGGAAGATCCCAAACTAAAAACAGCGGTATTTGCTAGCAACAACATAATG
 I  R  K  K  L  V  R  I  Y  C  G  N  E  L  E  D  P  K  L  K  T  A  V  F  A  S  N  N  I  M
GATCAGAAAAAAATCGGTCCGGATCTACTGCGGGAATGAGTTGGAAGATCCCAAACTAAAAACAGCGGTATTTGCTAGCAACAACATAATG
 I  R  K  K  S  V  R  I  Y  C  G  N  E  L  E  D  P  K  L  K  T  A  V  F  A  S  N  N  I  M
GATCAGAAAAAAATCGGTCCGGATCTACTGCGGGAATGAGTTGGAAGATCCCAAACTAAAAACAGCGGTATTTGCTAGCAACAACATAATG
 I  R  K  K  S  V  R  I  Y  C  G  N  E  L  E  D  P  K  L  K  T  A  V  F  A  S  N  N  I  M
GATCAGAAAAAAATCGGTCCGGATCTACTGCGGGAATGAGTTGGAAGATCCCAAACTAAAAACAGCGGTATTTGCTAGCAACAACATAATG
 I  R  K  K  S  V  R  I  Y  C  G  N  E  L  E  D  P  K  L  K  T  A  V  F  A  S  N  N  I  M
GATCAGAAAAAAATCGGTCCGGATCTACTGCGGGAATGAGTTGGAAGATCCCAAACTAAAAACAGCGGTATTTGCTAGCAACAACATAATG
 I  R  K  K  S  V  R  I  Y  C  G  N  E  L  E  D  P  K  L  K  T  A  V  F  A  S  N  N  I  M
GATCAGAAAAAAATCGGTCCGGATCTACTGCGGGAATGAGTTGGAAGATCCCAAACTAAAAACAGCGGTATTTGCTAGCAACAACATAATG
 I  R  K  K  S  V  R  I  Y  C  G  N  E  L  E  D  P  K  L  K  T  A  V  F  A  S  N  N  I  M
GATCAGAAAAAAATCGGTCCGGATCTACTGCGGGAATGAGTTGGAAGATCCCAAACTAAAAACAGCGGTATTTGCTAGCAACAACATAATG
 I  R  K  K  S  V  R  I  Y  C  G  N  E  L  E  D  P  K  L  K  T  A  V  F  A  S  N  N  I  M
GATCAGAAAAAAATCGGTCCGGATCTACTGCGGGAATGAGTTGGAAGATCCCAAACTAAAAACAGCGGTATTTGCTAGCAACAACATAATG
 I  R  K  K  S  V  R  I  Y  C  G  N  E  L  E  D  P  K  L  K  T  A  V  F  A  S  N  N  I  M
GATCAGAAAAAAATCGGTCCGGATCTACTGCGGGAATGAGTTGGAAGATCCCAAACTAAAAACAGCGGTATTTGCTAGCAACAACATAATG
 I  R  K  K  S  V  R  I  Y  C  G  N  E  L  E  D  P  K  L  K  T  A  V  F  A  S  N  N  I  M
GATCAGAAAAAAATCGGTCCGGATCTACTGCGGGAATGAGTTGGAAGATCCCAAACTAAAAACAGCGGTATTTGCTAGCAACAACATAATG
 I  R  K  K  S  V  R  I  Y  C  G  N  E  L  E  D  P  K  L  K  T  A  V  F  A  S  N  N  I  M
```

2,150 2,160 2,170 2,180 2,190 2,200 2,210 2,220

GAGGCAGTCAATCAATATAGATTGATCCGAAATCTCCAATATTATGGGTACATAAGAAATGTATCGAATCGATT

GAGGCAGTCAATCAATATAGATTGATCCGAAATCTCCAATATTATGGGTACATAAGAAATGTATCGAATCGATT
E A V N Q Y R L I R N L Q Y Y G Y I R N V S N R F

GAGGCAGTCAATCAATATAGATTGATCCGAAATCTCCAATATTATGGGTACATAAGAAATGTATCGAATCGATT
E A V N Q Y R L I R N L Q Y Y G Y I R N V S N R F

GAGGCAGTCAATCAATATAGATTGATCCGAAATCTCCAATATTATGGGTACATAAGAAATGTATCGAATCGATT
E A V N Q Y R L I R N L Q Y Y G Y I R N V S N R F

GAGGCAGTCAATCAATATAGATTGATCCGAAATCTCCAATATTATGGGTACATAAGAAATGTATCGAATCGATT
E A V N Q Y R L I R N L Q Y Y G Y I R N V S N R F

GAGGCAGTCAATCAATATAGATTGATCCGAAATCTCCAATATTATGGGTACATAAGAAATGTATCGAATCGATT
E A V N Q Y R L I R N L Q Y Y G Y I R N V S N R F

GAGGCAGTCAATCAATATAGATTGATCCGAAATCTCCAATATTATGGGTACATAAGAAATGTATCGAATCGATT
E A V N Q Y R L I R N L Q Y Y G Y I R N V S N R F

GAGGCAGTCAATCAATATAGATTGATCCGAAATCTCCAATATTATGGGTACATAAGAAATGTATCGAATCGATT
E A V N Q Y R L I R N L Q Y Y G Y I R N V S N R F

GAGGCAGTCAATCAATATAGATTGATCCGAAATCTCCAATATTATGGGTACATAAGAAATGTATCGAATCGATT
E A V N Q Y R L I R N L Q Y Y G Y I R N V S N R F

GAGGCAGTCAATCAATATAGATTGATCCGAAATCTCCAATATTATGGGTACATAAGAAATGTATCGAATCGATT
E A V N Q Y R L I R N L Q Y Y G Y I R N V S N R F

GAGGCAGTCAATCAATATAGATTGATCCGAAATCTCCAATATTATGGGTACATAAGAAATGTATCGAATCGATT
E A V N Q Y R L I R N L Q Y Y G Y I R N V S N R F

GAGGCAGTCAATCAATATAGATTGATCCGAAATCTCCAATATTATGGGTACATAAGAAATGTATCGAATCGATT
E A V N Q Y R L I R N L Q Y Y G Y I R N V S N R F

GAGGCAGTCAATCAATATAGATTGATCCGAAATCTCCAATATTATGGGTACATAAGAAATGTATCGAATCGATT
E A V N Q Y R L I R N L Q Y Y G Y I R N V S N R F

GAGGCAGTCAATCAATATAGATTGATCCGAAATCTCCAATATTATGGGTACATAAGAAATGTATCGAATCGATT
E A V N Q Y R L I R N L Q Y Y G Y I R N V S N R F

GAGGCAGTCAATCAATATAGATTGATCCGAAATCTCCAATATTATGGGTACATAAGAAATGTATCGAATCGATT
E A V N Q Y R L I R N L Q Y Y G Y I R N V S N R F

GAGGCAGTCAATCAATATAGATTGATCCGAAATCTCCAATATTATGGGTACATAAGAAATGTATCGAATCGATT
E A V N Q Y R L I R N L Q Y Y G Y I R N V S N R F

GAGGCAGTCAATCAATATAGATTGATCCGAAATCTCCAATATTATGGGTACATAAGAAATGTATCGAATCGATT
E A V N Q Y R L I R N L Q Y Y G Y I R N V S N R F

GAGGCAGTCAATCAATATAGATTGATCCGAAATCTCCAATATTATGGGTACATAAGAAATGTATCGAATCGATT
E A V N Q Y R L I R N L Q Y Y G Y I R N V S N R F

GAGGCAGTCAATCAATATAGATTGATCCGAAATCTCCAATATTATGGGTACATAAGAAATGTATCGAATCGATT
E A V N Q Y R L I R N L Q Y Y G Y I R N V S N R F

GAGGCAGTCAATCAATATAGATTGATCCGAAATCTCCAATATTATGGGTACATAAGAAATGTATCGAATCGATT
E A V N Q Y R L I R N L Q Y Y G Y I R N V S N R F

GAGGCAGTCAATCAATATAGATTGATCCGAAATCTCCAATATTATGGGTACATAAGAAATGTATCGAATCGATT
E A V N Q Y R L I R N L Q Y Y G Y I R N V S N R F

GAGGCAGTCAATCAATATAGATTGATCCGAAATCTCCAATATTATGGGTACATAAGAAATGTATCGAATCGATT
E A V N Q Y R L I R N L Q Y Y G Y I R N V S N R F

GAGGCAGTCAATCAATATAGATTGATCCGAAATCTCCAATATTATGGGTACATAAGAAATGTATCGAATCGATT
E A V N Q Y R L I R N L Q Y Y G Y I R N V S N R F

GAGGCAGTCAATCAATATAGATTGATCCGAAATCTCCAATATTATGGGTACATAAGAAATGTATCGAATCGATT
E A V N Q Y R L I R N L Q Y Y G Y I R N V S N R F

2,230 2,240 2,250 2,260 2,270 2,280 2,290 2,300

CTTTTTAATGAATAGATCCGATCGCAACTTCGAATATGGAATTCAAAGGGATCAAATAGGAAATGAGACTCTGAATCATATAAC

CTTTTTAATGAATAGATCCGATCGCAACTTCGAATATGGAATTCAAAGGGATCAAATAGGAAATGAGACTCTGAATCATATAAC
F L M N R S D R N F E Y G I Q R D Q I G N E T L N H I T

CTTTTTAATGAATAGATCCGATCGCAACTTCGAATATGGAATTCAAAGGGATCAAATAGGAAATGAGACTCTGAATCATATAAC
F L M N R S D R N F E Y G I Q R D Q I G N E T L N H I T

CTTTTTAATGAATAGATCCGATCGCAACTTCGAATATGGAATTCAAAGGGATCAAATAGGAAATGAGACTCTGAATCATATAAC
F L M N R S D R N F E Y G I Q R D Q I G N E T L N H I T

CTTTTTAATGAATAGATCCGATCGCAACTTCGAATATGGAATTCAAAGGGATCAAATAGGAAATGAGACTCTGAATCATATAAC
F L M N R S D R N F E Y G I Q R D Q I G N E T L N H I T

CTTTTTAATGAATAGATCCGATCGCAACTTCGAATATGGAATTCAAAGGGATCAAATAGGAAATGAGACTCTGAATCATATAAC
F L M N R S D R N F E Y G I Q R D Q I G N E T L N H I T

CTTTTTAATGAATAGATCCGATCGCAACTTCGAATATGGAATTCAAAGGGATCAAATAGGAAATGAGACTCTGAATCATATAAC
F L M N R S D R N F E Y G I Q R D Q I G N E T L N H I T

CTTTTTAATGAATAGATCCGATCGCAACTTCGAATATGGAATTCAAAGGGATCAAATAGGAAATGAGACTCTGAATCATATAAC
F L M N R S D R N F E Y G I Q R D Q I G N E T L N H I T

CTTTTTAATGAATAGATCCGATCGCAACTTCGAATATGGAATTCAAAGGGATCAAATAGGAAATGAGACTCTGAATCATATAAC
F L M N R S D R N F E Y G I Q R D Q I G N E T L N H I T

CTTTTTAATGAATAGATCCGATCGCAACTTCGAATATGGAATTCAAAGGGATCAAATAGGAAATGAGACTCTGAATCATATAAC
F L M N R S D R N F E Y G I Q R D Q I G N E T L N H I T

CTTTTTAATGAATAGATCCGATCGCAACTTCGAATATGGAATTCAAAGGGATCAAATAGGAAATGAGACTCTGAATCATATAAC
F L M N R S D R N F E Y G I Q R D Q I G N E T L N H I T

CTTTTTAATGAATAGATCCGATCGCAACTTCGAATATGGAATTCAAAGGGATCAAATAGGAAATGAGACTCTGAATCATATAAC
F L M N R S D R N F E Y G I Q R D Q I G N E T L N H I T

CTTTTTAATGAATAGATCCGATCGCAACTTCGAATATGGAATTCAAAGGGATCAAATAGGAAATGAGACTCTGAATCATATAAC
F L M N R S D R N F E Y G I Q R D Q I G N E T L N H I T

CTTTTTAATGAATAGATCCGATCGCAACTTCGAATATGGAATTCAAAGGGATCAAATAGGAAATGAGACTCTGAATCATATAAC
F L M N R S D R N F E Y G I Q R D Q I G N E T L N H I T

CTTTTTAATGAATAGATCCGATCGCAACTTCGAATATGGAATTCAAAGGGATCAAATAGGAAATGAGACTCTGAATCATATAAC
F L M N R S D R N F E Y G I Q R D Q I G N E T L N H I T

CTTTTTAATGAATAGATCCGATCGCAACTTCGAATATGGAATTCAAAGGGATCAAATAGGAAATGAGACTCTGAATCATATAAC
F L M N R S D R N F E Y G I Q R D Q I G N E T L N H I T

CTTTTTAATGAATAGATCCGATCGCAACTTCGAATATGGAATTCAAAGGGATCAAATAGGAAATGAGACTCTGAATCATATAAC
F L M N R S D R N F E Y G I Q R D Q I G N E T L N H I T

CTTTTTAATGAATAGATCCGATCGCAACTTCGAATATGGAATTCAAAGGGATCAAATAGGAAATGAGACTCTGAATCATATAAC
F L M N R S D R N F E Y G I Q R D Q I G N E T L N H I T

CTTTTTAATGAATAGATCCGATCGCAACTTCGAATATGGAATTCAAAGGGATCAAATAGGAAATGAGACTCTGAATCATATAAC
F L M N R S D R N F E Y G I Q R D Q I G N E T L N H I T

CTTTTTAATGAATAGATCCGATCGCAACTTCGAATATGGAATTCAAAGGGATCAAATAGGAAATGAGACTCTGAATCATATAAC
F L M N R S D R N F E Y G I Q R D Q I G N E T L N H I T

CTTTTTAATGAATAGATCCGATCGCAACTTCGAATATGGAATTCAAAGGGATCAAATAGGAAATGAGACTCTGAATCATATAAC
F L M N R S D R N F E Y G I Q R D Q I G N E T L N H I T

CTTTTTAATGAATAGATCCGATCGCAACTTCGAATATGGAATTCAAAGGGATCAAATAGGAAATGAGACTCTGAATCATATAAC
F L M N R S D R N F E Y G I Q R D Q I G N E T L N H I T

CTTTTTAATGAATAGATCCGATCGCAACTTCGAATATGGAATTCAAAGGGATCAAATAGGAAATGAGACTCTGAATCATATAAC
F L M N R S D R N F E Y G I Q R D Q I G N E T L N H I T

```
2,310     2,320     2,330     2,340     2,350     2,360     2,370     2,380     2,39
TATAATGAAATATACGATCAACCAACATTTATCGAATTTGAAAAAGAGTCAGAAGAAATGGTTTGATCCTCTTATTTCTCGAAC

TATAATGAAATATACGATCAACCAACATTTATCGAATTTGAAAAAGAGTCAGAAGAAATGGTTTGATCCTCTTATTTCTCGAAC
 I  M  K  Y  T  I  N  Q  H  L  S  N  L  K  K  S  Q  K  K  W  F  D  P  L  I  S  R  T
TATAATGAAATATACGATCAACCAACATTTATCGAATTTGAAAAAGAGTCAGAAGAAATGGTTTGATCCTCTTATTTCTCGAAC
 I  M  K  Y  T  I  N  Q  H  L  S  N  L  K  K  S  Q  K  K  W  F  D  P  L  I  S  R  T
TATAATGAAATATACGATCAACCAACATTTATCGAATTTGAAAAAGAGTCAGAAGAAATGGTTTGATCCTCTTATTTCTCGAAC
 I  M  K  Y  T  I  N  Q  H  L  S  N  L  K  K  S  Q  K  K  W  F  D  P  L  I  S  R  T
TATAATGAAATATACGATCAACCAACATTTATCGAATTTGAAAAAGAGTCAGAAGAAATGGTTTGATCCTCTTATTTCTCGAAC
 I  M  K  Y  T  I  N  Q  H  L  S  N  L  K  K  S  Q  K  K  W  F  D  P  L  I  S  R  T
TATAATGAAATATACGATCAATCAACATTTATCGAATTTGAAAAAGAGTCAGAAGAAATGGTTTGATCCTCTTATTTCTCGAAC
 I  M  K  Y  T  I  N  Q  H  L  S  N  L  K  K  S  Q  K  K  W  F  D  P  L  I  S  R  T
TATAATGAAATATACGATCAACCAACATTTATCGAATTTGAAAAAGAGTCAGAAGAAATGGTTTGATCCTCTTATTTCTCGAAC
 I  M  K  Y  T  I  N  Q  H  L  S  N  L  K  K  S  Q  K  K  W  F  D  P  L  I  S  R  T
TATAATGAAATATACGATCAACCAACATTTATCGAATTTGAAAAAGAGTCAGAAGAAATGGTTTGATCCTCTTATTTCTCGAAC
 I  M  K  Y  T  I  N  Q  H  L  S  N  L  K  K  S  Q  K  K  W  F  D  P  L  I  S  R  T
TATAATGAAATATACGATCAACCAACATTTATCGAATTTGAAAAAGAGTCAGAAGAAATGGTTTGATCCTCTTATTTCTCGAAC
 I  M  K  Y  T  I  N  Q  H  L  S  N  L  K  K  S  Q  K  K  W  F  D  P  L  I  S  R  T
TATAATGAAATATATGATCAACCAACATTTATCGAATTTGAAAAAGAGTCAGAAGAAATGGTTTGATCCTCTTATTTCTCGAAC
 I  M  K  Y  M  I  N  Q  H  L  S  N  L  K  K  S  Q  K  K  W  F  D  P  L  I  S  R  T
TATAATGAAATATACGATCAACCAACATTTATCGAATTTGAAAAAGAGTCAGAAGAAATGGTTTGATCCTCTTATTTCTCGAAC
 I  M  K  Y  T  I  N  Q  H  L  S  N  L  K  K  S  Q  K  K  W  F  D  P  L  I  S  R  T
TATAATGAAATATACGATCAACCAACATTTATCGAATTTGAAAAAGAGTCAGAAGAAATGGTTTGATCCTCTTATTTCTCGAAC
 I  M  K  Y  T  I  N  Q  H  L  S  N  L  K  K  S  Q  K  K  W  F  D  P  L  I  S  R  T
TATAATGAAATATACGATCAACCAACATTTATCGAATTTGAAAAAGAGTCAGAAGAAATGGTTTGATCCTCTTATTTCTCGAAC
 I  M  K  Y  T  I  N  Q  H  L  S  N  L  K  K  S  Q  K  K  W  F  D  P  L  I  S  R  T
TATAATGAAATATACGATCAACCAACATTTATCGAATTTGAAAAAGAGTCAGAAGAAATGGTTTGATCCTCTTATTTCTCGAAC
 I  M  K  Y  T  I  N  Q  H  L  S  N  L  K  K  S  Q  K  K  W  F  D  P  L  I  S  R  T
TATAATGAAATATACGATCAACCAACATTTATCGAATTTGAAAAAGAGTCAGAAGAAATGGTTTGATCCTCTTATTTCTCGAAC
 I  M  K  Y  T  I  N  Q  H  L  S  N  L  K  K  S  Q  K  K  W  F  D  P  L  I  S  R  T
TATAATGAAATATACGATCAACCAACATTTATCGAATTTGAAAAAGAGTCAGAAGAAATGGTTTGATCCTCTTATTTCTCGAAC
 I  M  K  Y  T  I  N  Q  H  L  S  N  L  K  K  S  Q  K  K  W  F  D  P  L  I  S  R  T
TATAATGAAATATACGATCAACCAACATTTATCGAATTTGAAAAAGAGTCAGAAGAAATGGTTTGATCCTCTTATTTCTCGAAC
 I  M  K  Y  T  I  N  Q  H  L  S  N  L  K  K  S  Q  K  K  W  F  D  P  L  I  S  R  T
TATAATGAAATATACGATCAACCAACATTTATCGAATTTGAAAAAGAGTCAGAAGAAATGGTTTGATCCTCTTATTTCTCGAAC
 I  M  K  Y  T  I  N  Q  H  L  S  N  L  K  K  S  Q  K  K  W  F  D  P  L  I  S  R  T
TATAATGAAATATACGATCAACCAACATTTATCGAATTTGAAAAAGAGTCAGAAGAAATGGTTTGATCCTCTTATTTCTCGAAC
 I  M  K  Y  T  I  N  Q  H  L  S  N  L  K  K  S  Q  K  K  W  F  D  P  L  I  S  R  T
TATAATGAAATATACGATCAACCAACATTTATCGAATTTGAAAAAGAGTCAGAAGAAATGGTTTGATCCTCTTATTTCTCGAAC
 I  M  K  Y  T  I  N  Q  H  L  S  N  L  K  K  S  Q  K  K  W  F  D  P  L  I  S  R  T
TATAATGAAATATACGATCAACCAACATTTATCGAATTTGAAAAAGAGTCAGAAGAAATGGTTTGATCCTCTTATTTCTCGAAC
 I  M  K  Y  T  I  N  Q  H  L  S  N  L  K  K  S  Q  K  K  W  F  D  P  L  I  S  R  T
TATAATGAAATATACGATCAACCAACATTTATCGAATTTGAAAAAGAGTCAGAAGAAATGGTTTGATCCTCTTATTTCTCGAAC
 I  M  K  Y  T  I  N  Q  H  L  S  N  L  K  K  S  Q  K  K  W  F  D  P  L  I  S  R  T
TATAATGAAATATACGATCAACCAACATTTATCGAATTTGAAAAAGAGTCAGAAGAAATGGTTTGATCCTCTTATTTCTCGAAC
 I  M  K  Y  T  I  N  Q  H  L  S  N  L  K  K  S  Q  K  K  W  F  D  P  L  I  S  R  T
TATAATGAAATATACGATCAACCAACATTTATCGAATTTGAAAAAGAGTCAGAAGAAATGGTTTGATCCTCTTATTTCTCGAAC
 I  M  K  Y  T  I  N  Q  H  L  S  N  L  K  K  S  Q  K  K  W  F  D  P  L  I  S  R  T
```

```
0        2,400     2,410     2,420     2,430     2,440     2,450     2,460     2,470
TGAGAGATCCATGAATCAGGATCCTGATGCATATAGATACAAATGGTCCGATGGGAGCAAGAATTTCCAGGAACATTTGGAACATTT

TGAGAGATCCATGAATCAGGATCCTGATGCATATAGATACAAATGGTCCGATGGGAGCAAGAATTTCCAGGAACATTTGGAACATTT
 E  R  S  M  N  Q  D  P  D  A  Y  R  Y  K  W  S  D  G  S  K  N  F  Q  E  H  L  E  H  F
TGAGAGATCCATGAATCAGGATCCTGATGCATATAGATACAAATGGTCCGATGGGAGCAAGAATTTCCAGGAACATTTGGAACATTT
 E  R  S  M  N  Q  D  P  D  A  Y  R  Y  K  W  S  D  G  S  K  N  F  Q  E  H  L  E  H  F
TGAGAGATCCATGAATCAGGATCCTGATGCATATAGATACAAATGGTCCGATGGGAGCAAGAATTTCCAGGAACATTTGGAACATTT
 E  R  S  M  N  Q  D  P  D  A  Y  R  Y  K  W  S  D  G  S  K  N  F  Q  E  H  L  E  H  F
TGAGAGATCCATGAATCAGGATCCTGATGCATATAGATACAAATGGTCCGATGGGAGCAAGAATTTCCAGGAACATTTGGAACATTT
 E  R  S  M  N  Q  D  P  D  A  Y  R  Y  K  W  S  D  G  S  K  N  F  Q  E  H  L  E  H  F
TGAGAGATCCATGAATCAGGATCCTGATGCATATAGATACAAATGGTCCGATGGGAGCAAGAATTTCCAGGAACATTTGGAACATTT
 E  R  S  M  N  Q  D  P  D  A  Y  R  Y  K  W  S  D  G  S  K  N  F  Q  E  H  L  E  H  F
TGAGAGATCCATGAATCAGGATCCTGATGCATATAGATACAAATGGTCCGATGGGAGCAAGAATTTCCAGGAACATTTGGAACATTT
 E  R  S  M  N  Q  D  P  D  A  Y  R  Y  K  W  S  D  G  S  K  N  F  Q  E  H  L  E  H  F
TGAGAGATCCATGAATCAGGATCCTGATGCATATAGATACAAATGGTCCGATGGGAGCAAGAATTTCCAGGAACATTTGGAACATTT
 E  R  S  M  N  Q  D  P  D  A  Y  R  Y  K  W  S  D  G  S  K  N  F  Q  E  H  L  E  H  F
TGAGAGATCCATGAATCAGGATCCTGATGCATATAGATACAAATGGTCCGATGGGAGCAAGAATTTCCAGGAACATTTGGAACATTT
 E  R  S  M  N  Q  D  P  D  A  Y  R  Y  K  W  S  D  G  S  K  N  F  Q  E  H  L  E  H  F
TGAGAGATCCATGAATCAGGATCCTGATGCATATAGATACAAATGGTCCGATGGGAGCAAGAATTTCCAGGAACATTTGGAACATTT
 E  R  S  M  N  Q  D  P  D  A  Y  R  Y  K  W  S  D  G  S  K  N  F  Q  E  H  L  E  H  F
TGAGAGATCCATGAATCAGGATCCTGATGCATATAGATACAAATGGTCCGATGGGAGCAAGAATTTCCAGGAACATTTGGAACATTT
 E  R  S  M  N  Q  D  P  D  A  Y  R  Y  K  W  S  D  G  S  K  N  F  Q  E  H  L  E  H  F
TGAGAGATCCATGAATCAGGATCCTGATGCATATAGATACAAATGGTCCGATGGGAGCAAGAATTTCCAGGAACATTTGGAACATTT
 E  R  S  M  N  Q  D  P  D  A  Y  R  Y  K  W  S  D  G  S  K  N  F  Q  E  H  L  E  H  F
TGAGAGATCCATGAATCAGGATCCTGATGCATATAGATACAAATGGTCCGATGGGAGCAAGAATTTCCAGGAACATTTGGAACATTT
 E  R  S  M  N  Q  D  P  D  A  Y  R  Y  K  W  S  D  G  S  K  N  F  Q  E  H  L  E  H  F
TGAGAGATCCATGAATCAGGATCCTGATGCATATAGATACAAATGGTCCGATGGGAGCAAGAATTTCCAGGAACATTTGGAACATTT
 E  R  S  M  N  Q  D  P  D  A  Y  R  Y  K  W  S  D  G  S  K  N  F  Q  E  H  L  E  H  F
TGAGAGATCCATGAATCAGGATCCTGATGCATATAGATACAAATGGTCCGATGGGAGCAAGAATTTCCAGGAACATTTGGAACATTT
 E  R  S  M  N  Q  D  P  D  A  Y  R  Y  K  W  S  D  G  S  K  N  F  Q  E  H  L  E  H  F
TGAGAGATCCATGAATCAGGATCCTGATGCATATAGATACAAATGGTCCGATGGGAGCAAGAATTTCCAGGAACATTTGGAACATTT
 E  R  S  M  N  Q  D  P  D  A  Y  R  Y  K  W  S  D  G  S  K  N  F  Q  E  H  L  E  H  F
TGAGAGATCCATGAATCAGGATCCTGATGCATATAGATACAAATGGTCCGATGGGAGCAAGAATTTCCAGGAACATTTGGAACATTT
 E  R  S  M  N  Q  D  P  D  A  Y  R  Y  K  W  S  D  G  S  K  N  F  Q  E  H  L  E  H  F
TGAGAGATCCATGAATCAGGATCCTGATGCATATAGATACAAATGGTCCGATGGGAGCAAGAATTTCCAGGAACATTTGGAACATTT
 E  R  S  M  N  Q  D  P  D  A  Y  R  Y  K  W  S  D  G  S  K  N  F  Q  E  H  L  E  H  F
TGAGAGATCCATGAATCAGGATCCTGATGCATATAGATACAAATGGTCCGATGGGAGCAAGAATTTCCAGGAACATTTGGAACATTT
 E  R  S  M  N  Q  D  P  D  A  Y  R  Y  K  W  S  D  G  S  K  N  F  Q  E  H  L  E  H  F
TGAGAGATCCATGAATCAGGATCCTGATGCATATAGATACAAATGGTCCGATGGGAGCAAGAATTTCCAGGAACATTTGGAACATTT
 E  R  S  M  N  Q  D  P  D  A  Y  R  Y  K  W  S  D  G  S  K  N  F  Q  E  H  L  E  H  F
TGAGAGATCCATGAATCAGGATCCTGATGCATATAGATACAAATGGTCCGATGGGAGCAAGAATTTCCAGGAACATTTGGAACATTT
 E  R  S  M  N  Q  D  P  D  A  Y  R  Y  K  W  S  D  G  S  K  N  F  Q  E  H  L  E  H  F
TGAGAGATCCATGAATCAGGATCCTGATGCATATAGATACAAATGGTCCGATGGGAGCAAGAATTTCCAGGAACATTTGGAACATTT
 E  R  S  M  N  Q  D  P  D  A  Y  R  Y  K  W  S  D  G  S  K  N  F  Q  E  H  L  E  H  F
TGAGAGATCCATGAATCAGGATCCTGATGCATATAGATACAAATGGTCCGATGGGAGCAAGAATTTCCAGGAACATTTGGAACATTT
 E  R  S  M  N  Q  D  P  D  A  Y  R  Y  K  W  S  D  G  S  K  N  F  Q  E  H  L  E  H  F
TGAGAGATCCATGAATCAGGATCCTGATGCATATAGATACAAATGGTCCGATGGGACCAAGAATTTCCAGGAACATTTGGAACATTT
 E  R  S  M  N  Q  D  P  D  A  Y  R  Y  K  W  S  D  G  T  K  N  F  Q  E  H  L  E  H  F
```

2,480 2,490 2,500 2,510 2,520 2,530 2,540 2,550 2,560

CGTTTCTGAACAGAAGAATCCTTTTCAAGTAGTGCAAGTAGTGTTCGATCAATTACGTATTCATCAATATTCGATTGATTGGTCCGAG

CGTTTCTGAACAGAAGAATCCTTTTCAAGTAGTGCAAGTAGTGTTCGATCAATTACGTATTCATCAATATTCGATTGATTGGTCCGAG
V S E Q K N P F Q V V Q V V F D Q L R I H Q Y S I D W S E

CGTTTCTGAACAGAAGAATCCTTTTCAAGTAGTGCAAGTAGTGTTCGATCAATTACGTATTCATCAATATTCGATTGATTGGTCCGAG
V S E Q K N P F Q V V Q V V F D Q L R I H Q Y S I D W S E

CGTTTCTGAACAGAAGAATCCTTTTCAAGTAGTGCAAGTAGTGTTCGATCAATTACGTATTCATCAATATTCGATTGATTGGTCCGAG
V S E Q K N P F Q V V Q V V F D Q L R I H Q Y S I D W S E

CGTTTCTGAACAGAAGAATCCTTTTCAAGTAGTGCAAGTAGTGTTCGATCAATTACGTATTCATCAATATTCGATTGATTGGTCCGAG
V S E Q K N P F Q V V Q V V F D Q L R I H Q Y S I D W S E

CGTTTCTGAACAGAAGAATCCTTTTCAAGTAGTGCAAGTAGTGTTCGATCAATTACGTATTCATCAATATTCGATTGATTGGTCCGAG
V S E Q K N P F Q V V Q V V F D Q L R I H Q Y S I D W S E

CGTTTCTGAACAGAAGAATCCTTTTCAAGTAGTGCAAGTAGTGTTCGATCAATTACGTATTCATCAATATTCGATTGATTGGTCCGAG
V S E Q K N P F Q V V Q V V F D Q L R I H Q Y S I D W S E

CGTTTCTGAACAGAAGAATCCTTTTCAAGTAGTGCAAGTAGTGTTCGATCAATTACGTATTCATCAATATTCGATTGATTGGTCCGAG
V S E Q K N P F Q V V Q V V F D Q L R I H Q Y S I D W S E

CGTTTCTGAACAGAAGAATCCTTTTCAAGTAGTGCAAGTAGTGTTCGATCAATTACGTATTCATCAATATTCGATTGATTGGTCCGAG
V S E Q K N P F Q V V Q V V F D Q L R I H Q Y S I D W S E

CGTTTCTGAACAGAAGAATCCTTTTCAAGTAGTGCAAGTAGTGTTCGATCAATTACGTATTCATCAATATTCGATTGATTGGTCCGAG
V S E Q K N P F Q V V Q V V F D Q L R I H Q Y S I D W S E

CGTTTCTGAACAGAAGAATCCTTTTCAAGTAGTGCAAGTAGTGTTCGATCAATTACGTATTCATCAATATTCGATTGATTGGTCCGAG
V S E Q K N P F Q V V Q V V F D Q L R I H Q Y S I D W S E

CGTTTCTGAACAGAAGAATCCTTTTCAAGTAGTGCAAGTAGTGTTCGATCAATTACGTATTCATCAATATTCGATTGATTGGTCCGAG
V S E Q K N P F Q V V Q V V F D Q L R I H Q Y S I D W S E

CGTTTCTGAACAGAAGAATCCTTTTCAAGTAGTGCAAGTAGTGTTCGATCAATTACGTATTCATCAATATTCGATTGATTGGTCCGAG
V S E Q K N P F Q V V Q V V F D Q L R I H Q Y S I D W S E

CGTTTCTGAACAGAAGAATCCTTTTCAAGTAGTGCAAGTAGTGTTCGATCAATTACGTATTCATCAATATTCGATTGATTGGTCCGAG
V S E Q K N P F Q V V Q V V F D Q L R I H Q Y S I D W S E

CGTTTCTGAACAGAAGAATCCTTTTCAAGTAGTGCAAGTAGTGTTCGATCAATTACGTATTCATCAATATTCGATTGATTGGTCCGAG
V S E Q K N P F Q V V Q V V F D Q L R I H Q Y S I D W S E

CGTTTCTGAACAGAAGAATCCTTTTCAAGTAGTGCAAGTAGTGTTCGATCAATTACGTATTCATCAATATTCGATTGATTGGTCCGAG
V S E Q K N P F Q V V Q V V F D Q L R I H Q Y S I D W S E

CGTTTCTGAACAGAAGAATCCTTTTCAAGTAGTGCAAGTAGTGTTCGATCAATTACGTATTCATCAATATTCGATTGATTGGTCCGAG
V S E Q K N P F Q V V Q V V F D Q L R I H Q Y S I D W S E

CGTTTCTGAACAGAAGAATCCTTTTCAAGTAGTGCAAGTAGTGTTCGATCAATTACGTATTCATCAATATTCGATTGATTGGTCCGAG
V S E Q K N P F Q V V Q V V F D Q L R I H Q Y S I D W S E

CGTTTCTGAACAGAAGAATCCTTTTCAAGTAGTGCAAGTAGTGTTCGATCAATTACGTATTCATCAATATTCGATTGATTGGTCCGAG
V S E Q K N P F Q V V Q V V F D Q L R I H Q Y S I D W S E

CGTTTCTGAACAGAAGAATCCTTTTCAAGTAGTGCAAGTAGTGTTCGATCAATTACGTATTCATCAATATTCGATTGATTGGTCCGAG
V S E Q K N P F Q V V Q V V F D Q L R I H Q Y S I D W S E

CGTTTCTGAACAGAAGAATCCTTTTCAAGTAGTGCAAGTAGTGTTCGATCAATTACGTATTCATCAATATTCGATTGATTGGTCCGAG
V S E Q K N P F Q V V Q V V F D Q L R I H Q Y S I D W S E

CGTTTCTGAACAGAAGAATCCTTTTCAAGTAGTGCAAGTAGTGTTCGATCAATTACGTATTCATCAATATTCGATTGATTGGTCCGAG
V S E Q K N P F Q V V Q V V F D Q L R I H Q Y S I D W S E

CGTTTCTGAACAGAAGAATCCTTTTCAAGTAGTGCAAGTAGTGTTCGATCAATTACGTATTCATCAATATTCGATTGATTGGTCCGAG
V S E Q K N P F Q V V Q V V F D Q L R I H Q Y S I D W S E

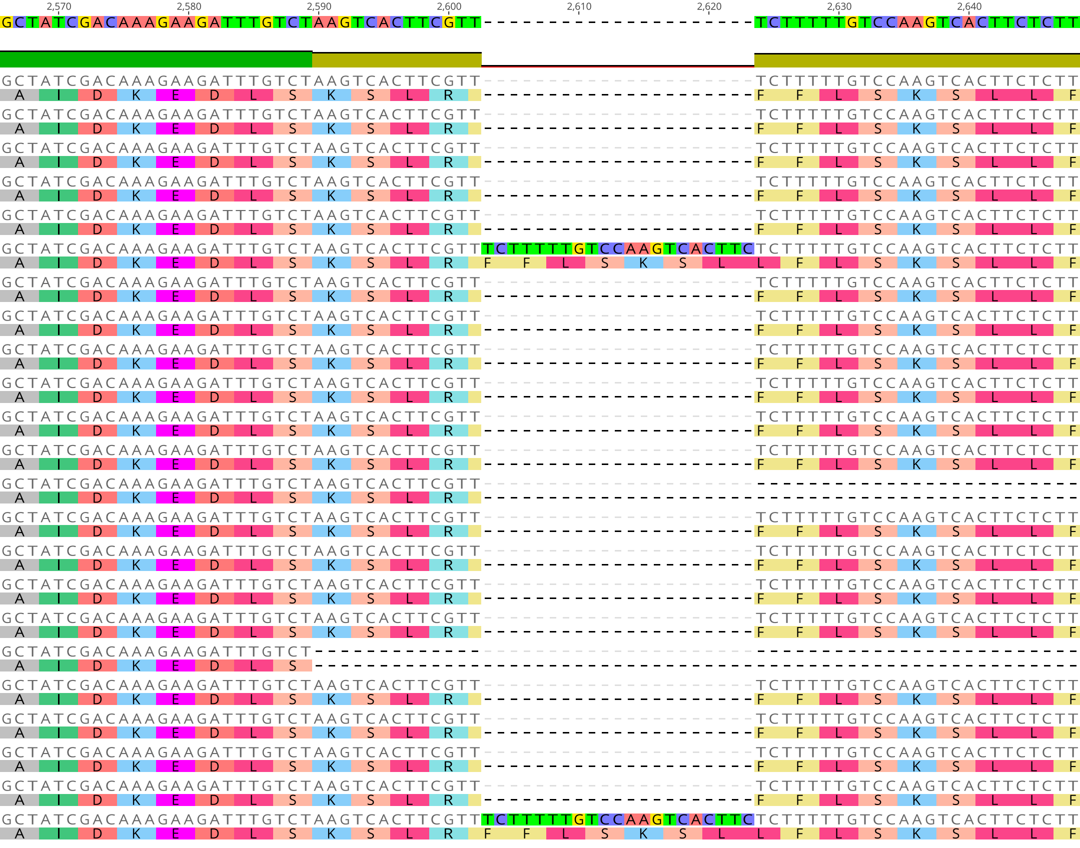

2,570
2,580
2,590
2,600
2,610
2,620
2,630
2,640
GCTATCGACAAAGAAGATTTGTCTAAGTCACTTCGTT
TCTTTTTGTCCAAGTCACTTCTCTT
A I D K E D L S K S L R
F F L S K S L L F

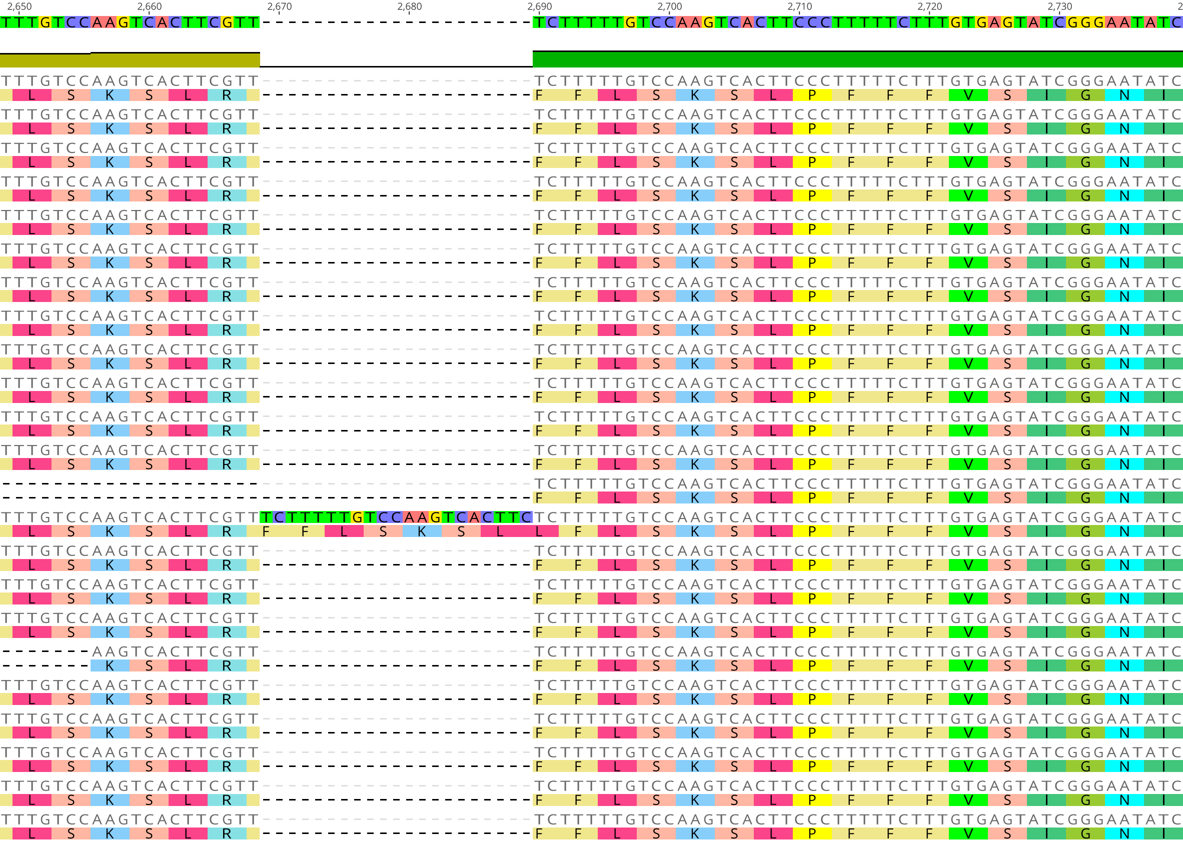
2,650
2,660
2,670
2,680
2,690
2,700
2,710
2,720
2,730
TTTGTCCAAGTCACTTCGTT
TCTTTTTGTCCAAGTCACTTCCCTTTTTCTTTGTGAGTATCGGGAATATC
L S K S L R
F F L S K S L P F F F V S I G N I
TTTGTCCAAGTCACTTCGTTTCTTTTTGTCCAAGTCACTTCTCTTTTTGTCCAAGTCACTTCCCTTTTTCTTTGTGAGTATCGGGAATATC
AAGTCACTTCGTT

,740 2,750 2,760 2,770 2,780 2,790 2,800 2,810 2,820

CCCATTCATAGGTCCGAGATCCACATCTATGAATTGAAAGGTCCGAATGATCAACTCTGCAATCAGTTGTTAGAATCCATAG

CCCATTCATAGGTCCGAGATCCACATCTATGAATTGAAAGGTCCGAATGATCAACTCTGCAATCAGTTGTTAGAATCCATAG
P I H R S E I H I Y E L K G P N D Q L C N Q L L E S I

CCCATTCATAGGTCCGAGATCCACATCTATGAATTGAAAGGTCCGAATGATCAACTCTGCAATCAGTTGTTAGAATCCATAG
P I H R S E I H I Y E L K G P N D Q L C N Q L L E S I

CCCATTCATAGGTCCGAGATCCACATCTATGAATTGAAAGGTCCGAATGATCAACTCTGCAATCAGTTGTTAGAATCCATAG
P I H R S E I H I Y E L K G P N D Q L C N Q L L E S I

CCCATTCATAGGTCCGAGATCCACATCTATGAATTGAAAGGTCCGAATGATCAACTCTGCAATCAGTTGTTAGAATCCATAG
P I H R S E I H I Y E L K G P N D Q L C N Q L L E S I

CCCATTCATAGGTCCGAGATCCACATCTATGAATTGAAAGGTCCGAATGATCAACTCTGCAATCAGTTGTTAGAATCCATAG
P I H R S E I H I Y E L K G P N D Q L C N Q L L E S I

CCCATTCATAGGTCCGAGATCCACATCTATGAATTGAAAGGTCCGAATGATCAACTCTGCAATCAGTTGTTAGAATCCATAG
P I H R S E I H I Y E L K G P N D Q L C N Q L L E S I

CCCATTCATAGGTCCGAGATCCACATCTATGAATTGAAAGGTCCGAATGATCAACTCTGCAATCAGTTGTTAGAATCCATAG
P I H R S E I H I Y E L K G P N D Q L C N Q L L E S I

CCCATTCATAGGTCCGAGATCCACATCTATGAATTGAAAGGTCCGAATGATCAACTCTGCAATCAGTTGTTAGAATCCATAG
P I H R S E I H I Y E L K G P N D Q L C N Q L L E S I

CCCATTCATAGGTCCGAGATCCACATCTATGAATTGAAAGGTCCGAATGATCAACTCTGCAATCAGTTGTTAGAATCCATAG
P I H R S E I H I Y E L K G P N D Q L C N Q L L E S I

CCCATTCATAGGTCCGAGATCCACATCTATGAATTGAAAGGTCCGAATGATCAACTCTGCAATCAGTTGTTAGAATCCATAG
P I H R S E I H I Y E L K G P N D Q L C N Q L L E S I

CCCATTCATAGGTCCGAGATCCACATCTATGAATTGAAAGGTCCGAATGATCAACTCTGCAATCAGTTGTTAGAATCCATAG
P I H R S E I H I Y E L K G P N D Q L C N Q L L E S I

CCCATTCATAGGTCCGAGATCCACATCTATGAATTGAAAGGTCCGAATGATCAACTCTGCAATCAGTTGTTAGAATCCATAG
P I H R S E I H I Y E L K G P N D Q L C N Q L L E S I

CCCATTCATAGGTCCGAGATCCACATCTATGAATTGAAAGGTCCGAATGATCAACTCTGCAATCAGTTGTTAGAATCCATAG
P I H R S E I H I Y E L K G P N D Q L C N Q L L E S I

CCCATTCATAGGTCCGAGATCCACATCTATGAATTGAAAGGTCCGAATGATCAACTCTGCAATCAGTTGTTAGAATCCATAG
P I H R S E I H I Y E L K G P N D Q L C N Q L L E S I

CCCATTCATAGGTCCGAGATCCACATCTATGAATTGAAAGGTCCGAATGATCAACTCTGCAATCAGTTGTTAGAATCCATAG
P I H R S E I H I Y E L K G P N D Q L C N Q L L E S I

CCCATTCATAGGTCCGAGATCCACATCTATGAATTGAAAGGTCCGAATGATCAACTCTGCAATCAGTTGTTAGAATCCATAG
P I H R S E I H I Y E L K G P N D Q L C N Q L L E S I

CCCATTCATAGGTCCGAGATCCACATCTATGAATTGAAAGGTCCGAATGATCAACTCTGCAATCAGTTGTTAGAATCCATAG
P I H R S E I H I Y E L K G P N D Q L C N Q L L E S I

CCCATTCATAGGTCCGAGATCCACATCTATGAATTGAAAGGTCCGAATGATCAACTCTGCAATCAGTTGTTAGAATCCATAG
P I H R S E I H I Y E L K G P N D Q L C N Q L L E S I

CCCATTCATAGGTCCGAGATCCACATCTATGAATTGAAAGGTCCGAATGATCAACTCTGCAATCAGTTGTTAGAATCCATAG
P I H R S E I H I Y E L K G P N D Q L C N Q L L E S I

CCCATTCATAGGTCCGAGATCCACATCTATGAATTGAAAGGTCCGAATGATCAACTCTGCAATCAGTTGTTAGAATCCATAG
P I H R S E I H I Y E L K G P N D Q L C N Q L L E S I

CCCATTCATAGGTCCGAGATCCACATCTATGAATTGAAAGGTCCGAATGATCAACTCTGCAATCAGTTGTTAGAATCCATAG
P I H R S E I H I Y E L K G P N D Q L C N Q L L E S I

CCCATTCATAGGTCCGAGATCCACATCTATGAATTGAAAGGTCCGAATGATCAACTCTGCAATCAGTTGTTAGAATCCATAG
P I H R S E I H I Y E L K G P N D Q L C N Q L L E S I

CCCATTCATAGGTCCGAGATCCACATCTATGAATTGAAAGGTCCGAATGATCAACTCTGCAATCAGTTGTTAGAATCCATAG
P I H R S E I H I Y E L K G P N D Q L C N Q L L E S I

```
2,830  2,840  2,850  2,860  2,870  2,880  2,890
GTGTTCAAATCGTTCATTTGAAGAAATTGAAACCCTTCTTATTGGATGATCATGATACTTCCCAAAGACCAAAATT

GTGTTCAAATCGTTCATTTGAAGAAATTGAAACCCTTCTTATTGGATGATCATGATACTTCCCAAAGACCAAAATT
G V Q I V H L K K L K P F L L D D H D T S Q R P K F
GTGTTCAAATCGTTCATTTGAAGAAATTGAAACCCTTCTTATTGGATGATCATGATACTTCCCAAAGACCAAAATT
G V Q I V H L K K L K P F L L D D H D T S Q R P K F
GTGTTCAAATCGTTCATTTGAAGAAATTGAAACCCTTCTTATTGGATGATCATGATACTTCCCAAAGACCAAAATT
G V Q I V H L K K L K P F L L D D H D T S Q R P K F
GTGTTCAAATCGTTCATTTGAAGAAATTGAAACCCTTCTTATTGGATGATCATGATACTTCCCAAAGACCAAAATT
G V Q I V H L K K L K P F L L D D H D T S Q R P K F
GTGTTCAAATCGTTCATTTGAAGAAATTGAAACCCTTCTTATTGGATGATCATGATACTTCCCAAAGACCAAAATT
G V Q I V H L K K L K P F L L D D H D T S Q R P K F
GTGTTCAAATCGTTCATTTGAAGAAATTGAAACCCTTCTTATTGGATGATCATGATACTTCCCAAAGACCAAAATT
G V Q I V H L K K L K P F L L D D H D T S Q R P K F
GTGTTCAAATCGTTCATTTGAAGAAATTGAAACCCTTCTTATTGGATGATCATGATACTTCCCAAAGACCAAAATT
G V Q I V H L K K L K P F L L D D H D T S Q R P K F
GTGTTCAAATCGTTCATTTGAAGAAATTGAAACCCTTCTTATTGGATGATCATGATACTTCCCAAAGACCAAAATT
G V Q I V H L K K L K P F L L D D H D T S Q R P K F
GTGTTCAAATCGTTCATTTGAAGAAATTGAAACCCTTCTTATTGGATGATCATGATACTTCCCAAAGACCAAAATT
G V Q I V H L K K L K P F L L D D H D T S Q R P K F
GTGTTCAAATCGTTCATTTGAAGAAATTGAAACCCTTCTTATTGGATGATCATGATACTTCCCAAAGACCAAAATT
G V Q I V H L K K L K P F L L D D H D T S Q R P K F
GTGTTCAAATCGTTCATTTGAAGAAATTGAAACCCTTCTTATTGGATGATCATGATACTTCCCAAAGACCAAAATT
G V Q I V H L K K L K P F L L D D H D T S Q R P K F
GTGTTCAAATCGTTCATTTGAAGAAATTGAAACCCTTCTTATTGGATGATCATGATACTTCCCAAAGACCAAAATT
G V Q I V H L K K L K P F L L D D H D T S Q R P K F
GTGTTCAAATCGTTCATTTGAAGAAATTGAAACCCTTCTTATTGGATGATCATGATACTTCCCAAAGACCAAAATT
G V Q I V H L K K L K P F L L D D H D T S Q R P K F
GTGTTCAAATCGTTCATTTGAAGAAATTGAAACCCTTCTTATTGGATGATCATGATACTTCCCAAAGACCAAAATT
G V Q I V H L K K L K P F L L D D H D T S Q R P K F
GTGTTCAAATCGTTCATTTGAAGAAATTGAAACCCTTCTTATTGGATGATCATGATACTTCCCAAAGACCAAAATT
G V Q I V H L K K L K P F L L D D H D T S Q R P K F
GTGTTCAAATCGTTCATTTGAAGAAATTGAAACCCTTCTTATTGGATGATCATGATACTTCCCAAAGACCAAAATT
G V Q I V H L K K L K P F L L D D H D T S Q R P K F
GTGTTCAAATCGTTCATTTGAAGAAATTGAAACCCTTCTTATTGGATGATCATGATACTTCCCAAAGACCAAAATT
G V Q I V H L K K L K P F L L D D H D T S Q R P K F
GTGTTCAAATCGTTCATTTGAAGAAATTGAAACCCTTCTTATTGGATGATCATGATACTTCCCAAAGACCAAAATT
G V Q I V H L K K L K P F L L D D H D T S Q R P K F
GTGTTCAAATCGTTCATTTGAAGAAATTGAAACCCTTCTTATTGGATGATCATGATACTTCCCAAAGACCAAAATT
G V Q I V H L K K L K P F L L D D H D T S Q R P K F
GTGTTCAAATCGTTCATTTGAAGAAATTGAAACCCTTCTTATTGGATGATCATGATACTTCCCAAAGACCAAAATT
G V Q I V H L K K L K P F L L D D H D T S Q R P K F
GTGTTCAAATCGTTCATTTGAAGAAATTGAAACCCTTCTTATTGGATGATCATGATACTTCCCAAAGACCAAAATT
G V Q I V H L K K L K P F L L D D H D T S Q R P K F
GTGTTCAAATCGTTCATTTGAAGAAATTGAAACCCTTCTTATTGGATGATCATGATACTTCCCAAAGACCAAAATT
G V Q I V H L K K L K P F L L D D H D T S Q R P K F
GTGTTCAAATCGTTCATTTGAAGAAATTGAAACCCTTCTTATTGGATGATCATGATACTTCCCAAAGACCAAAATT
G V Q I V H L K K L K P F L L D D H D T S Q R P K F
```

2,900 2,910 2,920 2,930 2,940 2,950 2,960 2,970

CTTGATCAATGGAGGAACAATATTACCATTTTTGTTCAAAAAGATACCAAAGCGGGTGATTGACTCATTCCATAC

CTTGATCAATGGAGGAACAATATTACCATTTTTGTTCAAAAAGATACCAAAGCGGGTGATTGACTCATTCCATAC
L I N G G T I L P F L F K K I P K R V I D S F H T

CTTGATCAATGGAGGAACAATATTACCATTTTTGTTCAAAAAGATACCAAAGCGGGTGATTGACTCATTCCATAC
L I N G G T I L P F L F K K I P K R V I D S F H T

CTTGATCAATGGAGGAACAATATTACCATTTTTGTTCAAAAAGATACCAAAGCGGGTGATTGACTCATTCCATAC
L I N G G T I L P F L F K K I P K R V I D S F H T

CTTGATCAATGGAGGAACAATATTACCATTTTTGTTCAAAAAGATACCAAAGCGGGTGATTGACTCATTCCATAC
L I N G G T I L P F L F K K I P K R V I D S F H T

CTTGATCAATGGAGGAACAATATTACCATTTTTGTTCAAAAAGATACCAAAGCGGGTGATTGACTCATTCCATAC
L I N G G T I L P F L F K K I P K R V I D S F H T

CTTGATCAATGGAGGAACAATATTACCATTTTTGTTCAAAAAGATACCAAAGCGGGTGATTGACTCATTCCATAC
L I N G G T I L P F L F K K I P K R V I D S F H T

CTTGATCAATGGAGGAACAATATTACCATTTTTGTTCAAAAAGATACCAAAGCGGGTGATTGACTCATTCCATAC
L I N G G T I L P F L F K K I P K R V I D S F H T

CTTGATCAATGGAGGAACAATATTACCATTTTTGTTCAAAAAGATACCAAAGCGGGTGATTGACTCATTCCATAC
L I N G G T I L P F L F K K I P K R V I D S F H T

CTTGATCAATGGAGGAACAATATTACCATTTTTGTTCAAAAAGATACCAAAGCGGGTGATTGACTCATTCCATAC
L I N G G T I L P F L F K K I P K R V I D S F H T

CTTGATCAATGGAGGAACAATATTACCATTTTTGTTCAAAAAGATACCAAAGCGGGTGATTGACTCATTCCATAC
L I N G G T I L P F L F K K I P K R V I D S F H T

CTTGATCAATGGAGGAACAATATTACCATTTTTGTTCAAAAAGATACCAAAGCGGGTGATTGACTCATTCCATAC
L I N G G T I L P F L F K K I P K R V I D S F H T

CTTGATCAATGGAGGAACAATATTACCATTTTTGTTCAAAAAGATACCAAAGCGGGTGATTGACTCATTCCATAC
L I N G G T I L P F L F K K I P K R V I D S F H T

CTTGATCAATGGAGGAACAATATTACCATTTTTGTTCAAAAAGATACCAAAGCGGGTGATTGACTCATTCCATAC
L I N G G T I L P F L F K K I P K R V I D S F H T

CTTGATCAATGGAGGAACAATATTACCATTTTTGTTCAAAAAGATACCAAAGCGGGTGATTGACTCATTCCATAC
L I N G G T I L P F L F K K I P K R V I D S F H T

CTTGATCAATGGAGGAACAATATTACCATTTTTGTTCAAAAAGATACCAAAGCGGGTGATTGACTCATTCCATAC
L I N G G T I L P F L F K K I P K R V I D S F H T

CTTGATCAATGGAGGAACAATATTACCATTTTTGTTCAAAAAGATACCAAAGCGGGTGATTGACTCATTCCATAC
L I N G G T I L P F L F K K I P K R V I D S F H T

CTTGATCAATGGAGGAACAATATTACCATTTTTGTTCAAAAAGATACCAAAGCGGGTGATTGACTCATTCCATAC
L I N G G T I L P F L F K K I P K R V I D S F H T

CTTGATCAATGGAGGAACAATATTACCATTTTTGTTCAAAAAGATACCAAAGCGGGTGATTGACTCATTCCATAC
L I N G G T I L P F L F K K I P K R V I D S F H T

CTTGATCAATGGAGGAACAATATTACCATTTTTGTTCAAAAAGATACCAAAGCGGGTGATTGACTCATTCCATAC
L I N G G T I L P F L F K K I P K R V I D S F H T

CTTGATCAATGGAGGAACAATATTACCATTTTTGTTCAAAAAGATACCAAAGCGGGTGATTGACTCATTCCATAC
L I N G G T I L P F L F K K I P K R V I D S F H T

CTTGATCAATGGAGGAACAATATTACCATTTTTGTTCAAAAAGATACCAAAGCGGGTGATTGACTCATTCCATAC
L I N G G T I L P F L F K K I P K R V I D S F H T

CTTGATCAATGGAGGAACAATATTACCATTTTTGTTCAAAAAGATACCAAAGCGGGTGATTGACTCATTCCATAC
L I N G G T I L P F L F K K I P K R V I D S F H T

CTTGATCAATGGAGGAACAATATTACCATTTTTGTTCAAAAAGATACCAAAGCGGGTGATTGACTCATTCCATAC
L I N G G T I L P F L F K K I P K R V I D S F H T

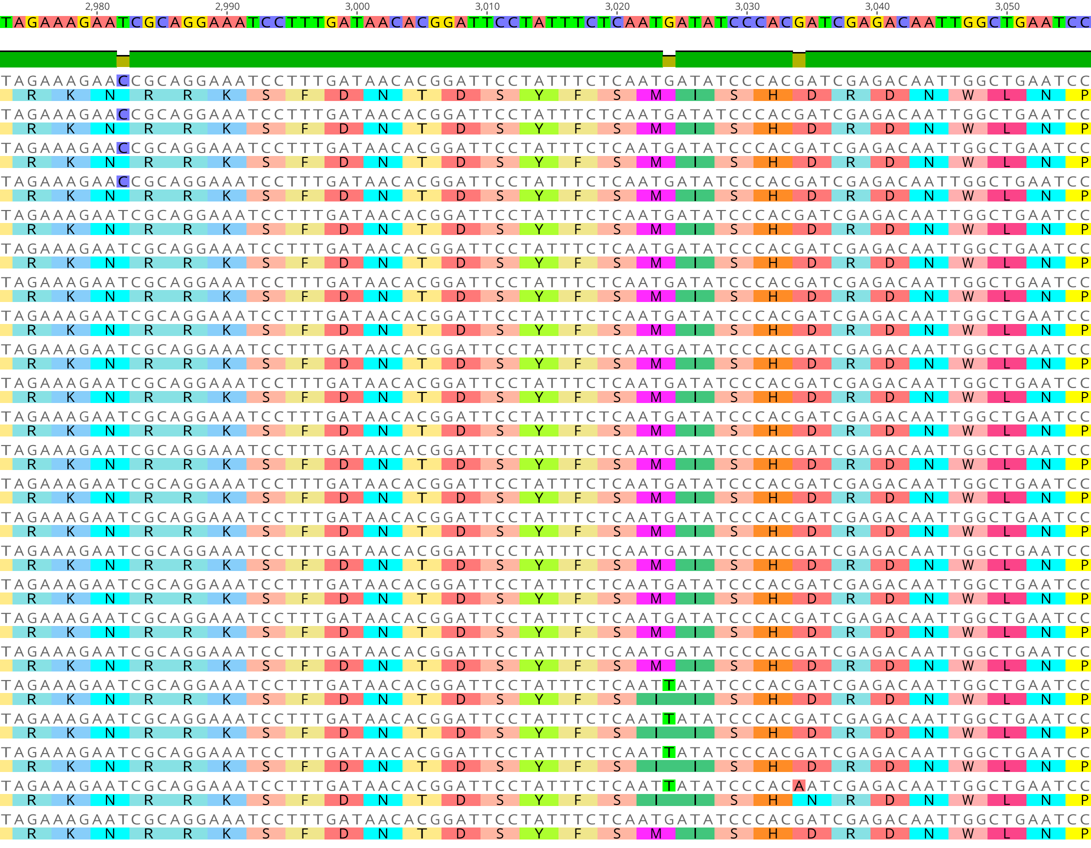

2,980 2,990 3,000 3,010 3,020 3,030 3,040 3,050
TAGAAAGAATCGCAGGAAATCCTTTGATAACACGGATTCCTATTTCTCAATGATATCCCACGATCGAGACAATTGGCTGAATCC
TAGAAAGAACCGCAGGAAATCCTTTGATAACACGGATTCCTATTTCTCAATGATATCCCACGATCGAGACAATTGGCTGAATCC
R K N R R K S F D N T D S Y F S M I S H D R D N W L N P
TAGAAAGAACCGCAGGAAATCCTTTGATAACACGGATTCCTATTTCTCAATGATATCCCACGATCGAGACAATTGGCTGAATCC
R K N R R K S F D N T D S Y F S M I S H D R D N W L N P
TAGAAAGAACCGCAGGAAATCCTTTGATAACACGGATTCCTATTTCTCAATGATATCCCACGATCGAGACAATTGGCTGAATCC
R K N R R K S F D N T D S Y F S M I S H D R D N W L N P
TAGAAAGAACCGCAGGAAATCCTTTGATAACACGGATTCCTATTTCTCAATGATATCCCACGATCGAGACAATTGGCTGAATCC
R K N R R K S F D N T D S Y F S M I S H D R D N W L N P
TAGAAAGAATCGCAGGAAATCCTTTGATAACACGGATTCCTATTTCTCAATGATATCCCACGATCGAGACAATTGGCTGAATCC
R K N R R K S F D N T D S Y F S M I S H D R D N W L N P
TAGAAAGAATCGCAGGAAATCCTTTGATAACACGGATTCCTATTTCTCAATGATATCCCACGATCGAGACAATTGGCTGAATCC
R K N R R K S F D N T D S Y F S M I S H D R D N W L N P
TAGAAAGAATCGCAGGAAATCCTTTGATAACACGGATTCCTATTTCTCAATGATATCCCACGATCGAGACAATTGGCTGAATCC
R K N R R K S F D N T D S Y F S M I S H D R D N W L N P
TAGAAAGAATCGCAGGAAATCCTTTGATAACACGGATTCCTATTTCTCAATGATATCCCACGATCGAGACAATTGGCTGAATCC
R K N R R K S F D N T D S Y F S M I S H D R D N W L N P
TAGAAAGAATCGCAGGAAATCCTTTGATAACACGGATTCCTATTTCTCAATGATATCCCACGATCGAGACAATTGGCTGAATCC
R K N R R K S F D N T D S Y F S M I S H D R D N W L N P
TAGAAAGAATCGCAGGAAATCCTTTGATAACACGGATTCCTATTTCTCAATGATATCCCACGATCGAGACAATTGGCTGAATCC
R K N R R K S F D N T D S Y F S M I S H D R D N W L N P
TAGAAAGAATCGCAGGAAATCCTTTGATAACACGGATTCCTATTTCTCAATGATATCCCACGATCGAGACAATTGGCTGAATCC
R K N R R K S F D N T D S Y F S M I S H D R D N W L N P
TAGAAAGAATCGCAGGAAATCCTTTGATAACACGGATTCCTATTTCTCAATGATATCCCACGATCGAGACAATTGGCTGAATCC
R K N R R K S F D N T D S Y F S M I S H D R D N W L N P
TAGAAAGAATCGCAGGAAATCCTTTGATAACACGGATTCCTATTTCTCAATGATATCCCACGATCGAGACAATTGGCTGAATCC
R K N R R K S F D N T D S Y F S M I S H D R D N W L N P
TAGAAAGAATCGCAGGAAATCCTTTGATAACACGGATTCCTATTTCTCAATGATATCCCACGATCGAGACAATTGGCTGAATCC
R K N R R K S F D N T D S Y F S M I S H D R D N W L N P
TAGAAAGAATCGCAGGAAATCCTTTGATAACACGGATTCCTATTTCTCAATGATATCCCACGATCGAGACAATTGGCTGAATCC
R K N R R K S F D N T D S Y F S M I S H D R D N W L N P
TAGAAAGAATCGCAGGAAATCCTTTGATAACACGGATTCCTATTTCTCAATGATATCCCACGATCGAGACAATTGGCTGAATCC
R K N R R K S F D N T D S Y F S M I S H D R D N W L N P
TAGAAAGAATCGCAGGAAATCCTTTGATAACACGGATTCCTATTTCTCAATGATATCCCACGATCGAGACAATTGGCTGAATCC
R K N R R K S F D N T D S Y F S M I S H D R D N W L N P
TAGAAAGAATCGCAGGAAATCCTTTGATAACACGGATTCCTATTTCTCAATGATATCCCACGATCGAGACAATTGGCTGAATCC
R K N R R K S F D N T D S Y F S M I S H D R D N W L N P
TAGAAAGAATCGCAGGAAATCCTTTGATAACACGGATTCCTATTTCTCAATTATATCCCACGATCGAGACAATTGGCTGAATCC
R K N R R K S F D N T D S Y F S I I S H D R D N W L N P
TAGAAAGAATCGCAGGAAATCCTTTGATAACACGGATTCCTATTTCTCAATTATATCCCACGATCGAGACAATTGGCTGAATCC
R K N R R K S F D N T D S Y F S I I S H D R D N W L N P
TAGAAAGAATCGCAGGAAATCCTTTGATAACACGGATTCCTATTTCTCAATTATATCCCACGATCGAGACAATTGGCTGAATCC
R K N R R K S F D N T D S Y F S I I S H D R D N W L N P
TAGAAAGAATCGCAGGAAATCCTTTGATAACACGGATTCCTATTTCTCAATTATATCCCACAATCGAGACAATTGGCTGAATCC
R K N R R K S F D N T D S Y F S I I S H N R D N W L N P
TAGAAAGAATCGCAGGAAATCCTTTGATAACACGGATTCCTATTTCTCAATGATATCCCACGATCGAGACAATTGGCTGAATCC
R K N R R K S F D N T D S Y F S M I S H D R D N W L N P

```
3,060      3,070      3,080      3,090      3,100      3,110      3,120      3,130
CGTGAAACCATTTCATAGAAGTTCATTGATATCTTCTTTTTATAAAGCAAATCGACTTCGATTCTTGAATGATCC

CGTGAAACCATTTCATAGAAGTTCATTGATATCTTCTTTTTATAAAGCAAATCGACTTCGATTCTTGAATGATCC
 V  K  P  F  H  R  S  S  L  I  S  S  F  Y  K  A  N  R  L  R  F  L  N  D  P
CGTGAAACCATTTCATAGAAGTTCATTGATATCTTCTTTTTATAAAGCAAATCGACTTCGATTCTTGAATGATCC
 V  K  P  F  H  R  S  S  L  I  S  S  F  Y  K  A  N  R  L  R  F  L  N  D  P
CGTGAAACCATTTCATAGAAGTTCATTGATATCTTCTTTTTATAAAGCAAATCGACTTCGATTCTTGAATGATCC
 V  K  P  F  H  R  S  S  L  I  S  S  F  Y  K  A  N  R  L  R  F  L  N  D  P
CGTGAAACCATTTCATAGAAGTTCATTGATATCTTCTTTTTATAAAGCAAATCGACTTCGATTCTTGAATGATCC
 V  K  P  F  H  R  S  S  L  I  S  S  F  Y  K  A  N  R  L  R  F  L  N  D  P
CGTGAAACCATTTCATAGAAGTTCATTGATATCTTCTTTTTATAAAGCAAATCGACTTCGATTCTTGAATGATCC
 V  K  P  F  H  R  S  S  L  I  S  S  F  Y  K  A  N  R  L  R  F  L  N  D  P
CGTGAAACCATTTCATAGAAGTTCATTGATATCTTCTTTTTATAAAGCAAATCGACTTCGATTCTTGAATGATCC
 V  K  P  F  H  R  S  S  L  I  S  S  F  Y  K  A  N  R  L  R  F  L  N  D  P
CGTGAAACCATTTCATAGAAGTTCATTGATATCTTCTTTTTATAAAGCAAATCGACTTCGATTCTTGAATGATCC
 V  K  P  F  H  R  S  S  L  I  S  S  F  Y  K  A  N  R  L  R  F  L  N  D  P
CGTGAAACCATTTCATAGAAGTTCATTGATATCTTCTTTTTATAAAGCAAATCGACTTCGATTCTTGAATGATCC
 V  K  P  F  H  R  S  S  L  I  S  S  F  Y  K  A  N  R  L  R  F  L  N  D  P
CGTGAAACCATTTCATAGAAGTTCATTGATATCTTCTTTTTATAAAGCAAATCGACTTCGATTCTTGAATGATCC
 V  K  P  F  H  R  S  S  L  I  S  S  F  Y  K  A  N  R  L  R  F  L  N  D  P
CGTGAAACCATTTCATAGAAGTTCATTGATATCTTCTTTTTATAAAGCAAATCGACTTCGATTCTTGAATGATCC
 V  K  P  F  H  R  S  S  L  I  S  S  F  Y  K  A  N  R  L  R  F  L  N  D  P
CGTGAAACCATTTCATAGAAGTTCATTGATATCTTCTTTTTATAAAGCAAATCGACTTCGATTCTTGAATGATCC
 V  K  P  F  H  R  S  S  L  I  S  S  F  Y  K  A  N  R  L  R  F  L  N  D  P
CGTGAAACCATTTCATAGAAGTTCATTGATATCTTCTTTTTATAAAGCAAATCGACTTCGATTCTTGAATGATCC
 V  K  P  F  H  R  S  S  L  I  S  S  F  Y  K  A  N  R  L  R  F  L  N  D  P
CGTGAAACCATTTCATAGAAGTTCATTGATATCTTCTTTTTATAAAGCAAATCGACTTCGATTCTTGAATGATCC
 V  K  P  F  H  R  S  S  L  I  S  S  F  Y  K  A  N  R  L  R  F  L  N  D  P
CGTGAAACCATTTCATAGAAGTTCATTGATATCTTCTTTTTATAAAGCAAATCGACTTCGATTCTTGAATGATCC
 V  K  P  F  H  R  S  S  L  I  S  S  F  Y  K  A  N  R  L  R  F  L  N  D  P
CGTGAAACCATTTCATAGAAGTTCATTGATATCTTCTTTTTATAAAGCAAATCGACTTCGATTCTTGAATGATCC
 V  K  P  F  H  R  S  S  L  I  S  S  F  Y  K  A  N  R  L  R  F  L  N  D  P
CGTGAAACCATTTCATAGAAGTTCATTGATATCTTCTTTTTATAAAGCAAATCGACTTCGATTCTTGAATGATCC
 V  K  P  F  H  R  S  S  L  I  S  S  F  Y  K  A  N  R  L  R  F  L  N  D  P
CGTGAAACCATTTCATAGAAGTTCATTGATATCTTCTTTTTATAAAGCAAATCGACTTCGATTCTTGAATGATCC
 V  K  P  F  H  R  S  S  L  I  S  S  F  Y  K  A  N  R  L  R  F  L  N  D  P
CGTGAAACCATTTCATAGAAGTTCATTGATATCTTCTTTTTATAAAGCAAATCGACTTCGATTCTTGAATGATCC
 V  K  P  F  H  R  S  S  L  I  S  S  F  Y  K  A  N  R  L  R  F  L  N  D  P
CGTGAAACCATTTCATAGAAGTTCATTGATATCTTCTTTTTATAAAGCAAATCGACTTCGATTCTTGAATGATCC
 V  K  P  F  H  R  S  S  L  I  S  S  F  Y  K  A  N  R  L  R  F  L  N  D  P
CGTGAAACCATTTCATAGAAGTTCATTGATATCTTCTTTTTATAAAGCAAATCGACTTCGATTCTTGAATGATCC
 V  K  P  F  H  R  S  S  L  I  S  S  F  Y  K  A  N  R  L  R  F  L  N  D  P
CGTGAAACCATTTCATAGAAGTTCATTGATATCTTCTTTTTATAAAGCAAATCGACTTCGATTCTTGAATGATCC
 V  K  P  F  H  R  S  S  L  I  S  S  F  Y  K  A  N  R  L  R  F  L  N  D  P
CGTGAAACCATTTCATAGAAGTTCATTGATATCTTCTTTTTATAAAGCAAATCGACTTCGATTCTTGAATGATCC
 V  K  P  F  H  R  S  S  L  I  S  S  F  Y  K  A  N  R  L  R  F  L  N  D  P
CGTGAAACCATTTCATAGAAGTTCATTGATATCTTCTTTTTATAAAGCAAATCGACTTCGATTCTTGAATGATCC
 V  K  P  F  H  R  S  S  L  I  S  S  F  Y  K  A  N  R  L  R  F  L  N  D  P
CGTGAAACCATTTCATAGAAGTTCATTGATATCTTCTTTTTATAAAGCAAATCGACTTCGATTCTTGAATCATCC
 V  K  P  F  H  R  S  S  L  I  S  S  F  Y  K  A  N  R  L  R  F  L  N  H  P
```

```
3,140 3,150 3,160 3,170 3,180 3,190 3,200 3,210 3,220
ACATCACTTCTGGTTCTATTGTAACAAAAGATTCCCCTTTGATGTGGAAAAGACCCGTATCAATAATTATGATCTTACATATGGACAATT

ACATCACTTCTGGTTCTATTGTAACAAAAGATTCCCCTTTGATGTGGAAAAGACCCGTATCAATAATTATGATCTTACATATGGACAATT
 H  H  F  W  F  Y  C  N  K  R  F  P  F  D  V  E  K  T  R  I  N  N  Y  D  L  T  Y  G  Q  F
ACATCACTTCTGGTTCTATTGTAACAAAAGATTCCCCTTTGATGTGGAAAAGACCCGTATCAATAATTATGATCTTACATATGGACAATT
 H  H  F  W  F  Y  C  N  K  R  F  P  F  D  V  E  K  T  R  I  N  N  Y  D  L  T  Y  G  Q  F
ACATCACTTCTGGTTCTATTGTAACAAAAGATTCCCCTTTGATGTGGAAAAGACCCGTATCAATAATTATGATCTTACATATGGACAATT
 H  H  F  W  F  Y  C  N  K  R  F  P  F  D  V  E  K  T  R  I  N  N  Y  D  L  T  Y  G  Q  F
ACATCACTTCTGGTTCTATTGTAACAAAAGATTCCCCTTTGATGTGGAAAAGACCCGTATCAATAATTATGATCTTACATATGGACAATT
 H  H  F  W  F  Y  C  N  K  R  F  P  F  D  V  E  K  T  R  I  N  N  Y  D  L  T  Y  G  Q  F
ACATCACTTCTGGTTCTATTGTAACAAAAGATTCCCCTTTGATGTGGAAAAGACCCGTATCAATAATTATGATCTTACATATGGACAATT
 H  H  F  W  F  Y  C  N  K  R  F  P  F  D  V  E  K  T  R  I  N  N  Y  D  L  T  Y  G  Q  F
ACATCACTTCTGGTTCTATTGTAACAAAAGATTCCCCTTTGATGTGGAAAAGACCCGTATCAATAATTATGATCTTACATATGGACAATT
 H  H  F  W  F  Y  C  N  K  R  F  P  F  D  V  E  K  T  R  I  N  N  Y  D  L  T  Y  G  Q  F
ACATCACTTCTGGTTCTATTGTAACAAAAGATTCCCCTTTGATGTGGAAAAGACCCGTATCAATAATTATGATCTTACATATGGACAATT
 H  H  F  W  F  Y  C  N  K  R  F  P  F  D  V  E  K  T  R  I  N  N  Y  D  L  T  Y  G  Q  F
ACATCACTTCTGGTTCTATTGTAACAAAAGATTCCCCTTTGATGTGGAAAAGACCCGTATCAATAATTATGATCTTACATATGGACAATT
 H  H  F  W  F  Y  C  N  K  R  F  P  F  D  V  E  K  T  R  I  N  N  Y  D  L  T  Y  G  Q  F
ACATCACTTCTGGTTCTATTGTAACAAAAGATTCCCCTTTGATGTGGAAAAGACCCGTATCAATAATTATGATCTTACATATGGACAATT
 H  H  F  W  F  Y  C  N  K  R  F  P  F  D  V  E  K  T  R  I  N  N  Y  D  L  T  Y  G  Q  F
ACATCACTTCTGGTTCTATTGTAACAAAAGATTCCCCTTTGATGTGGAAAAGACCCGTATCAATAATTATGATCTTACATATGGACAATT
 H  H  F  W  F  Y  C  N  K  R  F  P  F  D  V  E  K  T  R  I  N  N  Y  D  L  T  Y  G  Q  F
ACATCACTTCTGGTTCTATTGTAACAAAAGATTCCCCTTTGATGTGGAAAAGACCCGTATCAATAATTATGATCTTACATATGGACAATT
 H  H  F  W  F  Y  C  N  K  R  F  P  F  D  V  E  K  T  R  I  N  N  Y  D  L  T  Y  G  Q  F
ACATCACTTCTGGTTCTATTGTAACAAAAGATTCCCCTTTGATGTGGAAAAGACCCGTATCAATAATTATGATCTTACATATGGACAATT
 H  H  F  W  F  Y  C  N  K  R  F  P  F  D  V  E  K  T  R  I  N  N  Y  D  L  T  Y  G  Q  F
ACATCACTTCTGGTTCTATTGTAACAAAAGATTCCCCTTTGATGTGGAAAAGACCCGTATCAATAATTATGATCTTACATATGGACAATT
 H  H  F  W  F  Y  C  N  K  R  F  P  F  D  V  E  K  T  R  I  N  N  Y  D  L  T  Y  G  Q  F
ACATCACTTCTGGTTCTATTGTAACAAAAGATTCCCCTTTGATGTGGAAAAGACCCGTATCAATAATTATGATCTTACATATGGACAATT
 H  H  F  W  F  Y  C  N  K  R  F  P  F  D  V  E  K  T  R  I  N  N  Y  D  L  T  Y  G  Q  F
ACATCACTTCTGGTTCTATTGTAACAAAAGATTCCCCTTTGATGTGGAAAAGACCCGTATCAATAATTATGATCTTACATATGGACAATT
 H  H  F  W  F  Y  C  N  K  R  F  P  F  D  V  E  K  T  R  I  N  N  Y  D  L  T  Y  G  Q  F
ACATCACTTCTGGTTCTATTGTAACAAAAGATTCCCCTTTGATGTGGAAAAGACCCGTATCAATAATTATGATCTTACATATGGACAATT
 H  H  F  W  F  Y  C  N  K  R  F  P  F  D  V  E  K  T  R  I  N  N  Y  D  L  T  Y  G  Q  F
ACATCACTTCTGGTTCTATTGTAACAAAAGATTCCCCTTTGATGTGGAAAAAACCCGTATCAATAATTATGATCTTACATATGGACAATT
 H  H  F  W  F  Y  C  N  K  R  F  P  F  D  V  E  K  T  R  I  N  N  Y  D  L  T  Y  G  Q  F
ACATCACTTCTGGTTCTATTGTAACAAAAGATTCCCCTTTGATGTGGAAAAAACCCGTATCAATAATTATGATCTTACATATGGACAATT
 H  H  F  W  F  Y  C  N  K  R  F  P  F  D  V  E  K  T  R  I  N  N  Y  D  L  T  Y  G  Q  F
ACATCACTTCTGGTTCTATTGTAACAAAAGATTCCCCTTTGATGTGGAAAAGACCCGTATCAATAATTATGATCTTACATATGGACAATT
 H  H  F  W  F  Y  C  N  K  R  F  P  F  D  V  E  K  T  R  I  N  N  Y  D  L  T  Y  G  Q  F
ACATCACTTCTGGTTCTATTGTAACAAAAGATTCCCCTTTGATGTGGAAAAGACCCGTATCAATAATTATGATCTTACATATGGACAATT
 H  H  F  W  F  Y  C  N  K  R  F  P  F  D  V  E  K  T  R  I  N  N  Y  D  L  T  Y  G  Q  F
ACATCACTTCTGGTTCTATTGTAACAAAAGATTCCCCTTTGATGTGGAAAAGACCCGTATCAATAATTATGATCTTACATATGGACAATT
 H  H  F  W  F  Y  C  N  K  R  F  P  F  D  V  E  K  T  R  I  N  N  Y  D  L  T  Y  G  Q  F
ACATCACTTCTGGTTCTATTGTAACAAAAGATTCCCCTTTGATGTGGAAAAGACCCGTATCAATAATTATGATCTTACATATGGACAATT
 H  H  F  W  F  Y  C  N  K  R  F  P  F  D  V  E  K  T  R  I  N  N  Y  D  L  T  Y  G  Q  F
ACATCACTTCTGGTTCTATTGTAACAAAAGATTCCCCTTTGATGTGGAAAAGACCCGTATCAATAATTATGATCTTACATATGGACAATT
 H  H  F  W  F  Y  C  N  K  R  F  P  F  D  V  E  K  T  R  I  N  N  Y  D  L  T  Y  G  Q  F
```

```
3,230 3,240 3,250 3,260 3,270 3,280 3,290 3,300 3,31
CCTCAATATCTTGTCCATTCGCAACAAAATCTTTTCTTTGTGCGTCGGTAAAAAAGGATCTCTTTTTTTGGAGAGAGAGACTATTTCAC

CCTCAATATCTTGTCCATTCGCAACAAAATCTTTTCTTTGTGCGTCGGTAAAAAAGGATCTCTTTTTTTGGAGAGAGAGACTATTTCAC
 L  N  I  L  S  I  R  N  K  I  F  S  L  C  V  G  K  K  G  S  L  F  L  E  R  E  T  I  S
CCTCAATATCTTGTCCATTCGCAACAAAATCTTTTCTTTGTGCGTCGGTAAAAAAGGATCTCTTTTTTTGGAGAGAGAGACTATTTCAC
 L  N  I  L  S  I  R  N  K  I  F  S  L  C  V  G  K  K  G  S  L  F  L  E  R  E  T  I  S
CCTCAATATCTTGTCCATTCGCAACAAAATCTTTTCTTTGTGCGTCGGTAAAAAAGGATCTCTTTTTTTGGAGAGAGAGACTATTTCAC
 L  N  I  L  S  I  R  N  K  I  F  S  L  C  V  G  K  K  G  S  L  F  L  E  R  E  T  I  S
CCTCAATATCTTGTCCATTCGCAACAAAATCTTTTCTTTGTGCGTCGGTAAAAAAGGATCTCTTTTTTTGGAGAGAGAGACTATTTCAC
 L  N  I  L  S  I  R  N  K  I  F  S  L  C  V  G  K  K  G  S  L  F  L  E  R  E  T  I  S
CCTCAATATCTTGTCCATTCGCAACAAAATCTTTTCTTTGTGCGTCGGTAAAAAAGGATCTCTTTTTTTGGAGAGAGAGACTATTTCAC
 L  N  I  L  S  I  R  N  K  I  F  S  L  C  V  G  K  K  G  S  L  F  L  E  R  E  T  I  S
CCTCAATATCTTGTCCATTCGCAACAAAATCTTTTCTTTGTGCGTCGGTAAAAAAGGATCTCTTTTTTTGGAGAGAGAGACTATTTCAC
 L  N  I  L  S  I  R  N  K  I  F  S  L  C  V  G  K  K  G  S  L  F  L  E  R  E  T  I  S
CCTCAATATCTTGTCCATTCGCAACAAAATCTTTTCTTTGTGCGTCGGTAAAAAAGGATCTCTTTTTTTGGAGAGAGAGACTATTTCAC
 L  N  I  L  S  I  R  N  K  I  F  S  L  C  V  G  K  K  G  S  L  F  L  E  R  E  T  I  S
CCTCAATATCTTGTCCATTCGCAACAAAATCTTTTCTTTGTGCGTCGGTAAAAAAGGATCTCTTTTTTTGGAGAGAGAGACTATTTCAC
 L  N  I  L  S  I  R  N  K  I  F  S  L  C  V  G  K  K  G  S  L  F  L  E  R  E  T  I  S
CCTCAATATCTTGTCCATTCGCAACAAAATCTTTTCTTTGTGCGTCGGTAAAAAAGGATCTCTTTTTTTGGAGAGAGAGACTATTTCAC
 L  N  I  L  S  I  R  N  K  I  F  S  L  C  V  G  K  K  G  S  L  F  L  E  R  E  T  I  S
CCTCAATATCTTGTCCATTCGCAACAAAATCTTTTCTTTGTGCGTCGGTAAAAAAGGATCTCTTTTTTTGGAGAGAGAGACTATTTCAC
 L  N  I  L  S  I  R  N  K  I  F  S  L  C  V  G  K  K  G  S  L  F  L  E  R  E  T  I  S
CCTCAATATCTTGTCCATTCGCAACAAAATCTTTTCTTTGTGCGTCGGTAAAAAAGGATCTCTTTTTTTGGAGAGAGAGACTATTTCAC
 L  N  I  L  S  I  R  N  K  I  F  S  L  C  V  G  K  K  G  S  L  F  L  E  R  E  T  I  S
CCTCAATATCTTGTCCATTCGCAACAAAATCTTTTCTTTGTGCGTCGGTAAAAAAGGATCTCTTTTTTTGGAGAGAGAGACTATTTCAC
 L  N  I  L  S  I  R  N  K  I  F  S  L  C  V  G  K  K  G  S  L  F  L  E  R  E  T  I  S
CCTCAATATCTTGTCCATTCGCAACAAAATCTTTTCTTTGTGCGTCGGTAAAAAAGGATCTCTTTTTTTGGAGAGAGAGACTCTTTCAC
 L  N  I  L  S  I  R  N  K  I  F  S  L  C  V  G  K  K  G  S  L  F  L  E  R  E  T  L  S
CCTCAATATCTTGTCCATTCGCAACAAAATCTTTTCTTTGTGCGTCGGTAAAAAAGGATCTCTTTTTTTGGAGAGAGAGACTCTTTCAC
 L  N  I  L  S  I  R  N  K  I  F  S  L  C  V  G  K  K  G  S  L  F  L  E  R  E  T  L  S
CCTCAATATCTTGTCCATTCGCAACAAAATCTTTTCTTTGTGCGTCGGTAAAAAAGGATCTCTTTTTTTGGAGAGAGAGACTCTTTCAC
 L  N  I  L  S  I  R  N  K  I  F  S  L  C  V  G  K  K  G  S  L  F  L  E  R  E  T  L  S
CCTCAATATCTTGTCCATTCGCAACAAAATCTTTTCTTTGTGCGTCGGTAAAAAAGGATCTCTTTTTTTGGAGAGAGAGACTCTTTCAC
 L  N  I  L  S  I  R  N  K  I  F  S  L  C  V  G  K  K  G  S  L  F  L  E  R  E  T  L  S
CCTCAATATCTTGTCCATTCGCAACAAAATCTTTTCTTTGTGCGTCGGTAAAAAAGGATCTCTTTTTTTGGAGAGAGAGACTCTTTCAC
 L  N  I  L  S  I  R  N  K  I  F  S  L  C  V  G  K  K  G  S  L  F  L  E  R  E  T  L  S
CCTCAATATCTTGTCCATTCGCAACAAAATCTTTTCTTTGTGCGTCGGTAAAAAAGGATCTCTTTTTTTGGAGAGAGAGACTCTTTCAC
 L  N  I  L  S  I  R  N  K  I  F  S  L  C  V  G  K  K  G  S  L  F  L  E  R  E  T  L  S
CCTCAATATCTTGTCCATTCGCAACAAAATCTTTTCTTTGTGCGTCGGTAAAAAAGGATCTCTTTTTTTGGAGAGAGAGACTCTTTCAC
 L  N  I  L  S  I  R  N  K  I  F  S  L  C  V  G  K  K  G  S  L  F  L  E  R  E  T  L  S
CCTCAATATCTTGTCCATTCGCAACAAAATCTTTTCTTTGTGCGTCGGTAAAAAAGGATCTCTTTTTTTGGAGAGAGAGACTCTTTCAC
 L  N  I  L  S  I  R  N  K  I  F  S  L  C  V  G  K  K  G  S  L  F  L  E  R  E  T  L  S
CCTCAATATCTTGTCCATTCGCAACAAAATCTTTTCTTTGTGCGTCGGTAAAAAAGGATCTCTTTTTTTGGAGAGAGAGACTCTTTCAC
 L  N  I  L  S  I  R  N  K  I  F  S  L  C  V  G  K  K  G  S  L  F  L  E  R  E  T  L  S
CCTCAATATCTTGTCCATTCGCAACAAAATCTTTTCTTTGTGCGTCGGTAAAAAAGGATCTCTTTTTTTGGAGAGAGAGACTCTTTCAC
 L  N  I  L  S  I  R  N  K  I  F  S  L  C  V  G  K  K  G  S  L  F  L  E  R  E  T  L  S
CCTCAATATCTTGTCCATTCGCAACAAAATCTTTTCTTTGTGCGTCGGTAAAAAAGGATCTCTTTTTTTGGAGAGAGAGACTATTTCAC
 L  N  I  L  S  I  R  N  K  I  F  S  L  C  V  G  K  K  G  S  L  F  L  E  R  E  T  I  S
```

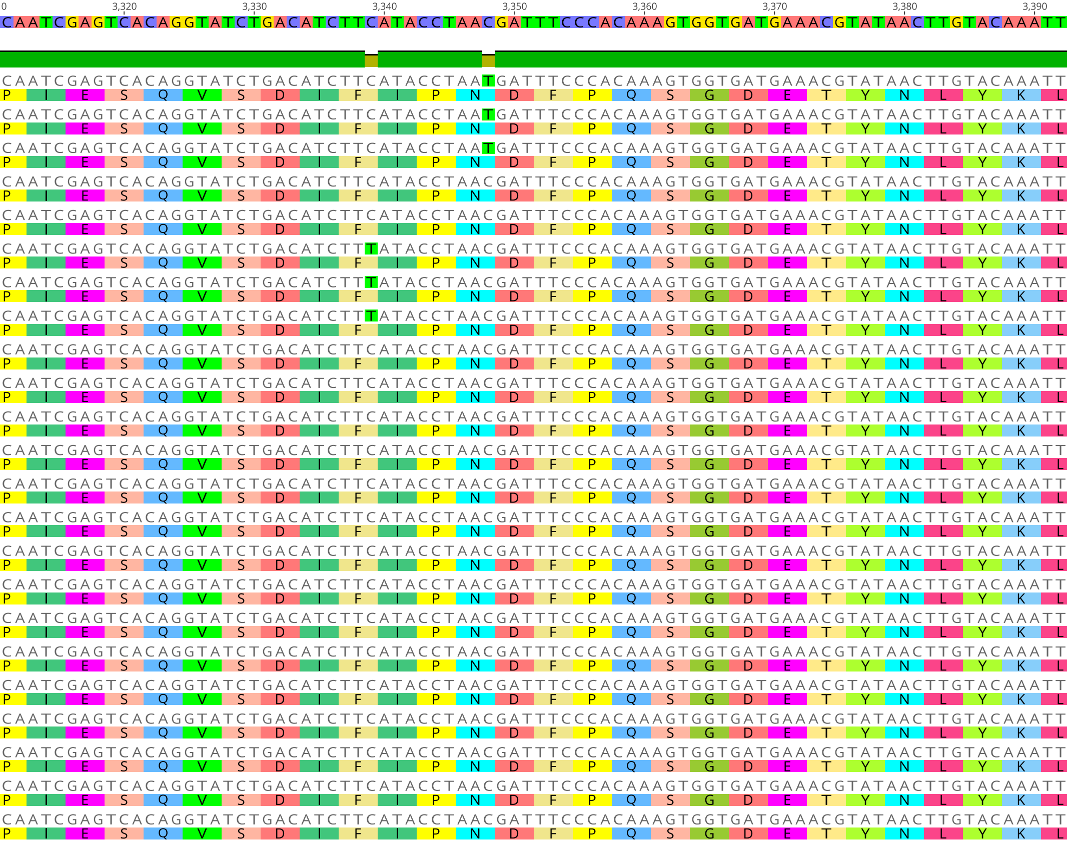

0
3,320
3,330
3,340
3,350
3,360
3,370
3,380
3,390
CAATCGAGTCACAGGTATCTGACATCTTCATACCTAACGATTTCCCACAAAGTGGTGATGAAACGTATAACTTGTACAAATT
CAATCGAGTCACAGGTATCTGACATCTTCATACCTAATGATTTCCCACAAAGTGGTGATGAAACGTATAACTTGTACAAATT
P I E S Q V S D I F I P N D F P Q S G D E T Y N L Y K L
CAATCGAGTCACAGGTATCTGACATCTTCATACCTAATGATTTCCCACAAAGTGGTGATGAAACGTATAACTTGTACAAATT
P I E S Q V S D I F I P N D F P Q S G D E T Y N L Y K L
CAATCGAGTCACAGGTATCTGACATCTTCATACCTAATGATTTCCCACAAAGTGGTGATGAAACGTATAACTTGTACAAATT
P I E S Q V S D I F I P N D F P Q S G D E T Y N L Y K L
CAATCGAGTCACAGGTATCTGACATCTTCATACCTAACGATTTCCCACAAAGTGGTGATGAAACGTATAACTTGTACAAATT
P I E S Q V S D I F I P N D F P Q S G D E T Y N L Y K L
CAATCGAGTCACAGGTATCTGACATCTTCATACCTAACGATTTCCCACAAAGTGGTGATGAAACGTATAACTTGTACAAATT
P I E S Q V S D I F I P N D F P Q S G D E T Y N L Y K L
CAATCGAGTCACAGGTATCTGACATCTTTATACCTAACGATTTCCCACAAAGTGGTGATGAAACGTATAACTTGTACAAATT
P I E S Q V S D I F I P N D F P Q S G D E T Y N L Y K L
CAATCGAGTCACAGGTATCTGACATCTTTATACCTAACGATTTCCCACAAAGTGGTGATGAAACGTATAACTTGTACAAATT
P I E S Q V S D I F I P N D F P Q S G D E T Y N L Y K L
CAATCGAGTCACAGGTATCTGACATCTTTATACCTAACGATTTCCCACAAAGTGGTGATGAAACGTATAACTTGTACAAATT
P I E S Q V S D I F I P N D F P Q S G D E T Y N L Y K L
CAATCGAGTCACAGGTATCTGACATCTTCATACCTAACGATTTCCCACAAAGTGGTGATGAAACGTATAACTTGTACAAATT
P I E S Q V S D I F I P N D F P Q S G D E T Y N L Y K L
CAATCGAGTCACAGGTATCTGACATCTTCATACCTAACGATTTCCCACAAAGTGGTGATGAAACGTATAACTTGTACAAATT
P I E S Q V S D I F I P N D F P Q S G D E T Y N L Y K L
CAATCGAGTCACAGGTATCTGACATCTTCATACCTAACGATTTCCCACAAAGTGGTGATGAAACGTATAACTTGTACAAATT
P I E S Q V S D I F I P N D F P Q S G D E T Y N L Y K L
CAATCGAGTCACAGGTATCTGACATCTTCATACCTAACGATTTCCCACAAAGTGGTGATGAAACGTATAACTTGTACAAATT
P I E S Q V S D I F I P N D F P Q S G D E T Y N L Y K L
CAATCGAGTCACAGGTATCTGACATCTTCATACCTAACGATTTCCCACAAAGTGGTGATGAAACGTATAACTTGTACAAATT
P I E S Q V S D I F I P N D F P Q S G D E T Y N L Y K L
CAATCGAGTCACAGGTATCTGACATCTTCATACCTAACGATTTCCCACAAAGTGGTGATGAAACGTATAACTTGTACAAATT
P I E S Q V S D I F I P N D F P Q S G D E T Y N L Y K L
CAATCGAGTCACAGGTATCTGACATCTTCATACCTAACGATTTCCCACAAAGTGGTGATGAAACGTATAACTTGTACAAATT
P I E S Q V S D I F I P N D F P Q S G D E T Y N L Y K L
CAATCGAGTCACAGGTATCTGACATCTTCATACCTAACGATTTCCCACAAAGTGGTGATGAAACGTATAACTTGTACAAATT
P I E S Q V S D I F I P N D F P Q S G D E T Y N L Y K L
CAATCGAGTCACAGGTATCTGACATCTTCATACCTAACGATTTCCCACAAAGTGGTGATGAAACGTATAACTTGTACAAATT
P I E S Q V S D I F I P N D F P Q S G D E T Y N L Y K L
CAATCGAGTCACAGGTATCTGACATCTTCATACCTAACGATTTCCCACAAAGTGGTGATGAAACGTATAACTTGTACAAATT
P I E S Q V S D I F I P N D F P Q S G D E T Y N L Y K L
CAATCGAGTCACAGGTATCTGACATCTTCATACCTAACGATTTCCCACAAAGTGGTGATGAAACGTATAACTTGTACAAATT
P I E S Q V S D I F I P N D F P Q S G D E T Y N L Y K L
CAATCGAGTCACAGGTATCTGACATCTTCATACCTAACGATTTCCCACAAAGTGGTGATGAAACGTATAACTTGTACAAATT
P I E S Q V S D I F I P N D F P Q S G D E T Y N L Y K L
CAATCGAGTCACAGGTATCTGACATCTTCATACCTAACGATTTCCCACAAAGTGGTGATGAAACGTATAACTTGTACAAATT
P I E S Q V S D I F I P N D F P Q S G D E T Y N L Y K L
CAATCGAGTCACAGGTATCTGACATCTTCATACCTAACGATTTCCCACAAAGTGGTGATGAAACGTATAACTTGTACAAATT
P I E S Q V S D I F I P N D F P Q S G D E T Y N L Y K L
CAATCGAGTCACAGGTATCTGACATCTTCATACCTAACGATTTCCCACAAAGTGGTGATGAAACGTATAACTTGTACAAATT
P I E S Q V S D I F I P N D F P Q S G D E T Y N L Y K L

```
3,400     3,410     3,420     3,430     3,440     3,450     3,460     3,470
GTACAAATCTTTCCATTTTCCAATTCGATCCGATCCATTCGTTCGTGGAGCTATTTACTCGATCGCAGACATTTCTGCAACAC

GTACAAATCTTTCCATTTTCCAATTCGATCCGATCCATTCGTTCGTGGAGCTATTTACTCGATCGCAGACATTTCTGCAACAC
 Y  K  S  F  H  F  P  I  R  S  D  P  F  V  R  G  A  I  Y  S  I  A  D  I  S  A  T
GTACAAATCTTTCCATTTTCCAATTCGATCCGATCCATTCGTTCGTGGAGCTATTTACTCGATCGCAGACATTTCTGCAACAC
 Y  K  S  F  H  F  P  I  R  S  D  P  F  V  R  G  A  I  Y  S  I  A  D  I  S  A  T
GTACAAATCTTTCCATTTTCCAATTCGATCCGATCCATTCGTTCGTGGAGCTATTTACTCGATCGCAGACATTTCTGCAACAC
 Y  K  S  F  H  F  P  I  R  S  D  P  F  V  R  G  A  I  Y  S  I  A  D  I  S  A  T
GTACAAATCTTTCCATTTTCCAATTCGATCCGATCCATTCGTTCGTGGAGCTATTTACTCGATCGCAGACATTTCTGCAACAC
 Y  K  S  F  H  F  P  I  R  S  D  P  F  V  R  G  A  I  Y  S  I  A  D  I  S  A  T
GTACAAATCTTTCCATTTTCCAATTCGATCCGATCCATTCGTTCGTGGAGCTATTTACTCGATCGCAGACATTTCTGCAACAC
 Y  K  S  F  H  F  P  I  R  S  D  P  F  V  R  G  A  I  Y  S  I  A  D  I  S  A  T
GTACAAATCTTTCCATTTTCCAATTCGATCCGATCCATTCGTTCGTGGAGCTATTTACTCGATCGCAGACATTTCTGCAACAC
 Y  K  S  F  H  F  P  I  R  S  D  P  F  V  R  G  A  I  Y  S  I  A  D  I  S  A  T
GTACAAATCTTTCCATTTTCCAATTCGATCCGATCCATTCGTTCGTGGAGCTATTTACTCGATCGCAGACATTTCTGCAACAC
 Y  K  S  F  H  F  P  I  R  S  D  P  F  V  R  G  A  I  Y  S  I  A  D  I  S  A  T
GTACAAATCTTTCCATTTTCCAATTCGATCCGATCCATTCGTTCGTGGAGCTATTTACTCGATCGCAGACATTTCTGCAACAC
 Y  K  S  F  H  F  P  I  R  S  D  P  F  V  R  G  A  I  Y  S  I  A  D  I  S  A  T
GTACAAATCTTTCCATTTTCCAATTCGATCCGATCCATTCGTTCGTGGAGCTATTTACTCGATCGCAGACATTTCTGCAACAC
 Y  K  S  F  H  F  P  I  R  S  D  P  F  V  R  G  A  I  Y  S  I  A  D  I  S  A  T
GTACAAATCTTTCCATTTTCCAATTCGATCCGATCCATTCGTTCGTGGAGCTATTTACTCGATCGCAGACATTTCTGCAACAC
 Y  K  S  F  H  F  P  I  R  S  D  P  F  V  R  G  A  I  Y  S  I  A  D  I  S  A  T
GTACAAATCTTTCCATTTTCCAATTCGATCCGATCCATTCGTTCGTGGAGCTATTTACTCGATCGCAGACATTTCTGCAACAC
 Y  K  S  F  H  F  P  I  R  S  D  P  F  V  R  G  A  I  Y  S  I  A  D  I  S  A  T
GTACAAATCTTTCCATTTTCCAATTCGATCCGATCCATTCGTTCGTGGAGCTATTTACTCGATCGCAGACATTTCTGCAACAC
 Y  K  S  F  H  F  P  I  R  S  D  P  F  V  R  G  A  I  Y  S  I  A  D  I  S  A  T
GTACAAATCTTTCCATTTTCCAATTCGATCCGATCCATTCGTTCGTGGAGCTATTTACTCGATCGCAGACATTTCTGCAACAC
 Y  K  S  F  H  F  P  I  R  S  D  P  F  V  R  G  A  I  Y  S  I  A  D  I  S  A  T
GTACAAATCTTTCCATTTTCCAATTCGATCCGATCCATTCGTTCGTGGAGCTATTTACTCGATCGCAGACATTTCTGCAACAC
 Y  K  S  F  H  F  P  I  R  S  D  P  F  V  R  G  A  I  Y  S  I  A  D  I  S  A  T
GTACAAATCTTTCCATTTTCCAATTCGATCCGATCCATTCGTTCGTGGAGCTATTTACTCGATCGCAGACATTTCTGCAACAC
 Y  K  S  F  H  F  P  I  R  S  D  P  F  V  R  G  A  I  Y  S  I  A  D  I  S  A  T
GTACAAATCTTTCCATTTTCCAATTCGATCCGATCCATTCGTTCGTGGAGCTATTTACTCGATCGCAGACATTTCTGCAACAC
 Y  K  S  F  H  F  P  I  R  S  D  P  F  V  R  G  A  I  Y  S  I  A  D  I  S  A  T
GTACAAATCTTTCCATTTTCCAATTCGATCCGATCCATTCGTTCGTGGAGCTATTTACTCGATCGCAGACATTTCTGCAACAC
 Y  K  S  F  H  F  P  I  R  S  D  P  F  V  R  G  A  I  Y  S  I  A  D  I  S  A  T
GTACAAATCTTTCCATTTTCCAATTCGATCCGATCCATTCGTTCGTGGAGCTATTTACTCGATCGCAGACATTTCTGCAACAC
 Y  K  S  F  H  F  P  I  R  S  D  P  F  V  R  G  A  I  Y  S  I  A  D  I  S  A  T
GTACAAATCTTTCCATTTTCCAATTCGATCCGATCCATTCGTTCGTGGAGCTATTTACTCGATCGCAGACATTTCTGCAACAC
 Y  K  S  F  H  F  P  I  R  S  D  P  F  V  R  G  A  I  Y  S  I  A  D  I  S  A  T
GTACAAATCTTTCCATTTTCCAATTCGATCCGATCCATTCGTTCGTGGAGCTATTTACTCGATCGCAGACATTTCTGCAACAC
 Y  K  S  F  H  F  P  I  R  S  D  P  F  V  R  G  A  I  Y  S  I  A  D  I  S  A  T
GTACAAATCTTTCCATTTTCCAATTCGATCCGATCCATTCGTTCGTGGAGCTATTTACTCGATCGCAGACATTTCTGCAACAC
 Y  K  S  F  H  F  P  I  R  S  D  P  F  V  R  G  A  I  Y  S  I  A  D  I  S  A  T
GTACAAATCTTTCCATTTTCCAATTCGATCCGATCCATTCGTTCGTGGAGCTATTTACTCGATCGCAGACATTTCTGCAACAC
 Y  K  S  F  H  F  P  I  R  S  D  P  F  V  R  G  A  I  Y  S  I  A  D  I  S  A  T
GTACAAATCTTTCCATTTTCCAATTCGATCCGATCCATTCGTTCGTGGAGCTATTTACTCGATCGCAGACATTTCTGCAACAC
 Y  K  S  F  H  F  P  I  R  S  D  P  F  V  R  G  A  I  Y  S  I  A  D  I  S  A  T
```

3,480 3,490 3,500 3,510 3,520 3,530 3,540 3,550

CTCTAACAGAGGAACAAATAGTCAATTTGGAAAAACTTATTGTCAGCCTCTTTCAGATATGAATCTATCTGATTCAGAAGG

CTCTAACAGAGGAACAAATAGTCAATTTGGAAAAACTTATTGTCAGCCTCTTTCAGATATGAATCTATCTGATTCAGAAGG
P L T E E Q I V N L E K T Y C Q P L S D M N L S D S E G

CTCTAACAGAGGAACAAATAGTCAATTTGGAAAAACTTATTGTCAGCCTCTTTCAGATATGAATCTATCTGATTCAGAAGG
P L T E E Q I V N L E K T Y C Q P L S D M N L S D S E G

CTCTAACAGAGGAACAAATAGTCAATTTGGAAAAACTTATTGTCAGCCTCTTTCAGATATGAATCTATCTGATTCAGAAGG
P L T E E Q I V N L E K T Y C Q P L S D M N L S D S E G

CTCTAACAGAGGAACAAATAGTCAATTTGGAAAAACTTATTGTCAGCCTCTTTCAGATATGAATCTATCTGATTCAGAAGG
P L T E E Q I V N L E K T Y C Q P L S D M N L S D S E G

CTCTAACAGAGGAACAAATAGTCAATTTGGAAAAACTTATTGTCAGCCTCTTTCAGATATGAATCTATCTGATTCAGAAGG
P L T E E Q I V N L E K T Y C Q P L S D M N L S D S E G

CTCTAACAGAGGAACAAATAGTCAATTTGGAAAAACTTATTGTCAGCCTCTTTCAGATATGAATCTATCTGATTCAGAAGG
P L T E E Q I V N L E K T Y C Q P L S D M N L S D S E G

CTCTAACAGAGGAACAAATAGTCAATTTGGAAAAACTTATTGTCAGCCTCTTTCAGATATGAATCTATCTGATTCAGAAGG
P L T E E Q I V N L E K T Y C Q P L S D M N L S D S E G

CTCTAACAGAGGAACAAATAGTCAATTTGGAAAAACTTATTGTCAGCCTCTTTCAGATATGAATCTATCTGATTCAGAAGG
P L T E E Q I V N L E K T Y C Q P L S D M N L S D S E G

CTCTAACAGAGGAACAAATAGTCAATTTGGAAAAACTTATTGTCAGCCTCTTTCAGATATGAATCTATCTGATTCAGAAGG
P L T E E Q I V N L E K T Y C Q P L S D M N L S D S E G

CTCTAACAGAGGAACAAATAGTCAATTTGGAAAAACTTATTGTCAGCCTCTTTCAGATATGAATCTATCTGATTCAGAAGG
P L T E E Q I V N L E K T Y C Q P L S D M N L S D S E G

CTCTAACAGAGGAACAAATAGTCAATTTGGAAAAACTTATTGTCAGCCTCTTTCAGATATGAATCTATCTGATTCAGAAGG
P L T E E Q I V N L E K T Y C Q P L S D M N L S D S E G

CTCTAACAGAGGAACAAATAGTCAATTTGGAAAAACTTATTGTCAGCCTCTTTCAGATATGAATCTATCTGATTCAGAAGG
P L T E E Q I V N L E K T Y C Q P L S D M N L S D S E G

CTCTAACAGAGGAACAAATAGTCAATTTGGAAAAACTTATTGTCAGCCTCTTTCAGATATGAATCTATCTGATTCAGAAGG
P L T E E Q I V N L E K T Y C Q P L S D M N L S D S E G

CTCTAACAGAGGAACAAATAGTCAATTTGGAAAAACTTATTGTCAGCCTCTTTCAGATATGAATCTATCTGATTCAGAAGG
P L T E E Q I V N L E K T Y C Q P L S D M N L S D S E G

CTCTAACAGAGGAACAAATAGTCAATTTGGAAAAACTTATTGTCAGCCTCTTTCAGATATGAATCTATCTGATTCAGAAGG
P L T E E Q I V N L E K T Y C Q P L S D M N L S D S E G

CTCTAACAGAGGAACAAATAGTCAATTTGGAAAAACTTATTGTCAGCCTCTTTCAGATATGAATCTATCTGATTCAGAAGG
P L T E E Q I V N L E K T Y C Q P L S D M N L S D S E G

CTCTAACAGAGGAACAAATAGTCAATTTGGAAAAACTTATTGTCAGCCTCTTTCAGATATGAATCTATCTGATTCAGAAGG
P L T E E Q I V N L E K T Y C Q P L S D M N L S D S E G

CTCTAACAGAGGAACAAATAGTCAATTTGGAAAAACTTATTGTCAGCCTCTTTCAGATATGAATCTATCTGATTCAGAAGG
P L T E E Q I V N L E K T Y C Q P L S D M N L S D S E G

CTCTAACAGAGGAACAAATAGTCAATTTGGAAAAACTTATTGTCAGCCTCTTTCAGATATGAATCTATCTGATTCAGAAGG
P L T E E Q I V N L E K T Y C Q P L S D M N L S D S E G

CTCTAACAGAGGAACAAATAGTCAATTTGGAAAAACTTATTGTCAGCCTCTTTCAGATATGAATCTATCTGATTCAGAAGG
P L T E E Q I V N L E K T Y C Q P L S D M N L S D S E G

CTCTAACAGAGGAACAAATAGTCAATTTGGAAAAACTTATTGTCAGCCTCTTTCAGATATGAATCTATCTGATTCAGAAGG
P L T E E Q I V N L E K T Y C Q P L S D M N L S D S E G

CTCTAACAGAGGAACAAATAGTCAATTTGGAAAAACTTATTGTCAGCCTCTTTCAGATATGAATCTATCTGATTCAGAAGG
P L T E E Q I V N L E K T Y C Q P L S D M N L S D S E G

CTCTAACAGAGGAACAAATAGTCAATTTGGAAAAACTTATTGTCAGCCTCTTTCAGATATGAATCTATCTGATTCAGAAGG
P L T E E Q I V N L E K T Y C Q P L S D M N L S D S E G

3,560 3,570 3,580 3,590 3,600 3,610 3,620 3,630

GAATAACTTGCATCAGTATCTCAGTTTCAATTCAAACATGGGTTTGATTCACACTCCATGTTCTGAGAAGTATTTAC

GAATAACTTGCATCAGTATCTCAGTTTCAATTCAAACATGGGTTTGATTCACACTCCATGTTCTGAGAAGTATTTAC
N N L H Q Y L S F N S N M G L I H T P C S E K Y L

GAATAACTTGCATCAGTATCTCAGTTTCAATTCAAACATGGGTTTGATTCACACTCCATGTTCTGAGAAGTATTTAC
N N L H Q Y L S F N S N M G L I H T P C S E K Y L

GAATAACTTGCATCAGTATCTCAGTTTCAATTCAAACATGGGTTTGATTCACACTCCATGTTCTGAGAAGTATTTAC
N N L H Q Y L S F N S N M G L I H T P C S E K Y L

GAATAACTTGCATCAGTATCTCAGTTTCAATTCAAACATGGGTTTGATTCACACTCCATGTTCTGAGAAGTATTTAC
N N L H Q Y L S F N S N M G L I H T P C S E K Y L

GAATAACTTGCATCAGTATCTCAGTTTCAATTCAAACATGGGTTTGATTCACACTCCATGTTCTGAGAAGTATTTAC
N N L H Q Y L S F N S N M G L I H T P C S E K Y L

GAATAACTTGCATCAGTATCTCAGTTTCAATTCAAACATGGGTTTGATTCACACTCCATGTTCTGAGAAGTATTTAC
N N L H Q Y L S F N S N M G L I H T P C S E K Y L

GAATAACTTGCATCAGTATCTCAGTTTCAATTCAAACATGGGTTTGATTCACACTCCATGTTCTGAGAAGTATTTAC
N N L H Q Y L S F N S N M G L I H T P C S E K Y L

GAATAACTTGCATCAGTATCTCAGTTTCAATTCAAACATGGGTTTGATTCACACTCCATGTTCTGAGAAGTATTTAC
N N L H Q Y L S F N S N M G L I H T P C S E K Y L

GAATAACTTGCATCAGTATCTCAGTTTCAATTCAAACATGGGTTTGATTCACACTCCATGTTCTGAGAAGTATTTAC
N N L H Q Y L S F N S N M G L I H T P C S E K Y L

GAATAACTTGCATCAGTATCTCAGTTTCAATTCAAACATGGGTTTGATTCACACTCCATGTTCTGAGAAGTATTTAC
N N L H Q Y L S F N S N M G L I H T P C S E K Y L

GAATAACTTGCATCAGTATCTCAGTTTCAATTCAAACATGGGTTTGATTCACACTCCATGTTCTGAGAAGTATTTAC
N N L H Q Y L S F N S N M G L I H T P C S E K Y L

GAATAACTTGCATCAGTATCTCAGTTTCAATTCAAACATGGGTTTGATTCACACTCCATGTTCTGAGAAGTATTTAC
N N L H Q Y L S F N S N M G L I H T P C S E K Y L

GAATAACTTGCATCAGTATCTCAGTTTCAATTCAAACATGGGTTTGATTCACACTCCATGTTCTGAGAAGTATTTAC
N N L H Q Y L S F N S N M G L I H T P C S E K Y L

GAATAACTTGCATCAGTATCTCAGTTTCAATTCAAACATGGGTTTGATTCACACTCCATGTTCTGAGAAGTCTTTAC
N N L H Q Y L S F N S N M G L I H T P C S E K S L

GAATAACTTGCATCAGTATCTCAGTTTCAATTCAAACATGGGTTTGATTCACACTCCATGTTCTGAGAAGTATTTAC
N N L H Q Y L S F N S N M G L I H T P C S E K Y L

GAATAACTTGCATCAGTATCTCAGTTTCAATTCAAACATGGGTTTGATTCACACTCCATGTTCTGAGAAGTATTTAC
N N L H Q Y L S F N S N M G L I H T P C S E K Y L

GAATAACTTGCATCAGTATCTCAGTTTCAATTCAAACATGGGTTTGATTCACACTCCATGTTCTGAGAAGTCTTTAC
N N L H Q Y L S F N S N M G L I H T P C S E K S L

GAATAACTTGCATCAGTATCTCAGTTTCAATTCAAACATGGGTTTGATTCACACTCCATGTTCTGAGAAGTCTTTAC
N N L H Q Y L S F N S N M G L I H T P C S E K S L

GAATAACTTGCATCAGTATCTCAGTTTCAATTCAAACATGGGTTTGATTCACACTCCATGTTCTGAGAAGTATTTAC
N N L H Q Y L S F N S N M G L I H T P C S E K Y L

GAATAACTTGCATCAGTATCTCAGTTTCAATTCAAACATGGGTTTGATTCACACTCCATGTTCTGAGAAGTATTTAC
N N L H Q Y L S F N S N M G L I H T P C S E K Y L

GAATAACTTGCATCAGTATCTCAGTTTCAATTCAAACATGGGTTTGATTCACACTCCATGTTCTGAGAAGTATTTAC
N N L H Q Y L S F N S N M G L I H T P C S E K Y L

GAATAACTTGCATCAGTATCTCAGTTTCAATTCAAACATGGGTTTGATTCACACTCCATGTTCTGAGAAGTATTTAC
N N L H Q Y L S F N S N M G L I H T P C S E K Y L

GAATAACTTGCATCAGTATCTCAGTTTCAATTCAAACATGGGTTTGATTCACACTCCATGTTCTGAGAAGTATTTAC
N N L H Q Y L S F N S N M G L I H T P C S E K Y L

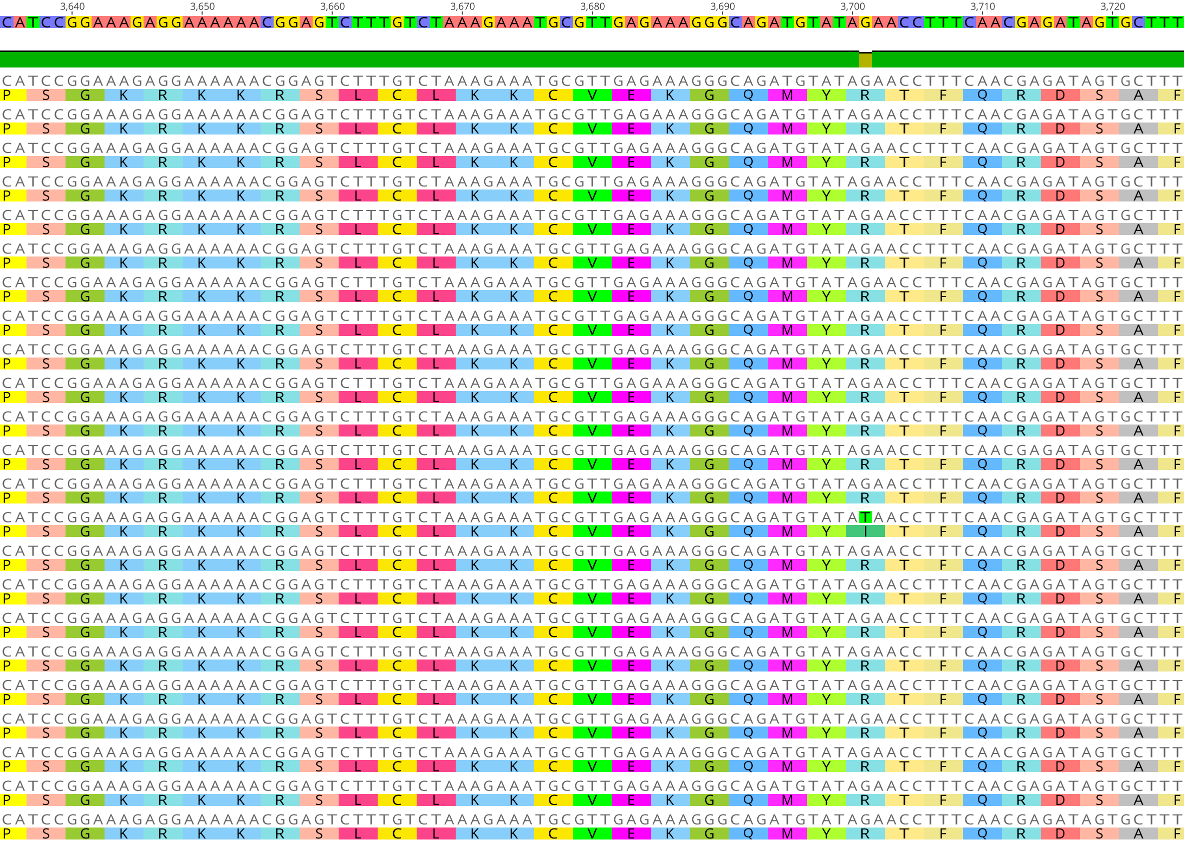
3,640 3,650 3,660 3,670 3,680 3,690 3,700 3,710 3,720
CATCCGGAAAGAGGAAAAAACGGAGTCTTTGTCTAAAGAAATGCGTTGAGAAAGGGCAGATGTATAGAACCTTTCAACGAGATAGTGCTTT
CATCCGGAAAGAGGAAAAAACGGAGTCTTTGTCTAAAGAAATGCGTTGAGAAAGGGCAGATGTATAGAACCTTTCAACGAGATAGTGCTTT
P S G K R K K R S L C L K K C V E K G Q M Y R T F Q R D S A F
CATCCGGAAAGAGGAAAAAACGGAGTCTTTGTCTAAAGAAATGCGTTGAGAAAGGGCAGATGTATAGAACCTTTCAACGAGATAGTGCTTT
P S G K R K K R S L C L K K C V E K G Q M Y R T F Q R D S A F
CATCCGGAAAGAGGAAAAAACGGAGTCTTTGTCTAAAGAAATGCGTTGAGAAAGGGCAGATGTATAGAACCTTTCAACGAGATAGTGCTTT
P S G K R K K R S L C L K K C V E K G Q M Y R T F Q R D S A F
CATCCGGAAAGAGGAAAAAACGGAGTCTTTGTCTAAAGAAATGCGTTGAGAAAGGGCAGATGTATAGAACCTTTCAACGAGATAGTGCTTT
P S G K R K K R S L C L K K C V E K G Q M Y R T F Q R D S A F
CATCCGGAAAGAGGAAAAAACGGAGTCTTTGTCTAAAGAAATGCGTTGAGAAAGGGCAGATGTATAGAACCTTTCAACGAGATAGTGCTTT
P S G K R K K R S L C L K K C V E K G Q M Y R T F Q R D S A F
CATCCGGAAAGAGGAAAAAACGGAGTCTTTGTCTAAAGAAATGCGTTGAGAAAGGGCAGATGTATAGAACCTTTCAACGAGATAGTGCTTT
P S G K R K K R S L C L K K C V E K G Q M Y R T F Q R D S A F
CATCCGGAAAGAGGAAAAAACGGAGTCTTTGTCTAAAGAAATGCGTTGAGAAAGGGCAGATGTATAGAACCTTTCAACGAGATAGTGCTTT
P S G K R K K R S L C L K K C V E K G Q M Y R T F Q R D S A F
CATCCGGAAAGAGGAAAAAACGGAGTCTTTGTCTAAAGAAATGCGTTGAGAAAGGGCAGATGTATAGAACCTTTCAACGAGATAGTGCTTT
P S G K R K K R S L C L K K C V E K G Q M Y R T F Q R D S A F
CATCCGGAAAGAGGAAAAAACGGAGTCTTTGTCTAAAGAAATGCGTTGAGAAAGGGCAGATGTATAGAACCTTTCAACGAGATAGTGCTTT
P S G K R K K R S L C L K K C V E K G Q M Y R T F Q R D S A F
CATCCGGAAAGAGGAAAAAACGGAGTCTTTGTCTAAAGAAATGCGTTGAGAAAGGGCAGATGTATAGAACCTTTCAACGAGATAGTGCTTT
P S G K R K K R S L C L K K C V E K G Q M Y R T F Q R D S A F
CATCCGGAAAGAGGAAAAAACGGAGTCTTTGTCTAAAGAAATGCGTTGAGAAAGGGCAGATGTATAGAACCTTTCAACGAGATAGTGCTTT
P S G K R K K R S L C L K K C V E K G Q M Y R T F Q R D S A F
CATCCGGAAAGAGGAAAAAACGGAGTCTTTGTCTAAAGAAATGCGTTGAGAAAGGGCAGATGTATAGAACCTTTCAACGAGATAGTGCTTT
P S G K R K K R S L C L K K C V E K G Q M Y R T F Q R D S A F
CATCCGGAAAGAGGAAAAAACGGAGTCTTTGTCTAAAGAAATGCGTTGAGAAAGGGCAGATGTATAGAACCTTTCAACGAGATAGTGCTTT
P S G K R K K R S L C L K K C V E K G Q M Y R T F Q R D S A F
CATCCGGAAAGAGGAAAAAACGGAGTCTTTGTCTAAAGAAATGCGTTGAGAAAGGGCAGATGTATAGAACCTTTCAACGAGATAGTGCTTT
P S G K R K K R S L C L K K C V E K G Q M Y R T F Q R D S A F
CATCCGGAAAGAGGAAAAAACGGAGTCTTTGTCTAAAGAAATGCGTTGAGAAAGGGCAGATGTATATAACCTTTCAACGAGATAGTGCTTT
P S G K R K K R S L C L K K C V E K G Q M Y I T F Q R D S A F
CATCCGGAAAGAGGAAAAAACGGAGTCTTTGTCTAAAGAAATGCGTTGAGAAAGGGCAGATGTATAGAACCTTTCAACGAGATAGTGCTTT
P S G K R K K R S L C L K K C V E K G Q M Y R T F Q R D S A F
CATCCGGAAAGAGGAAAAAACGGAGTCTTTGTCTAAAGAAATGCGTTGAGAAAGGGCAGATGTATAGAACCTTTCAACGAGATAGTGCTTT
P S G K R K K R S L C L K K C V E K G Q M Y R T F Q R D S A F
CATCCGGAAAGAGGAAAAAACGGAGTCTTTGTCTAAAGAAATGCGTTGAGAAAGGGCAGATGTATAGAACCTTTCAACGAGATAGTGCTTT
P S G K R K K R S L C L K K C V E K G Q M Y R T F Q R D S A F
CATCCGGAAAGAGGAAAAAACGGAGTCTTTGTCTAAAGAAATGCGTTGAGAAAGGGCAGATGTATAGAACCTTTCAACGAGATAGTGCTTT
P S G K R K K R S L C L K K C V E K G Q M Y R T F Q R D S A F
CATCCGGAAAGAGGAAAAAACGGAGTCTTTGTCTAAAGAAATGCGTTGAGAAAGGGCAGATGTATAGAACCTTTCAACGAGATAGTGCTTT
P S G K R K K R S L C L K K C V E K G Q M Y R T F Q R D S A F
CATCCGGAAAGAGGAAAAAACGGAGTCTTTGTCTAAAGAAATGCGTTGAGAAAGGGCAGATGTATAGAACCTTTCAACGAGATAGTGCTTT
P S G K R K K R S L C L K K C V E K G Q M Y R T F Q R D S A F
CATCCGGAAAGAGGAAAAAACGGAGTCTTTGTCTAAAGAAATGCGTTGAGAAAGGGCAGATGTATAGAACCTTTCAACGAGATAGTGCTTT
P S G K R K K R S L C L K K C V E K G Q M Y R T F Q R D S A F
CATCCGGAAAGAGGAAAAAACGGAGTCTTTGTCTAAAGAAATGCGTTGAGAAAGGGCAGATGTATAGAACCTTTCAACGAGATAGTGCTTT
P S G K R K K R S L C L K K C V E K G Q M Y R T F Q R D S A F
CATCCGGAAAGAGGAAAAAACGGAGTCTTTGTCTAAAGAAATGCGTTGAGAAAGGGCAGATGTATAGAACCTTTCAACGAGATAGTGCTTT
P S G K R K K R S L C L K K C V E K G Q M Y R T F Q R D S A F

3,730 3,740 3,750 3,760 3,770 3,780 3,790 3,800

```
TTCAAATCTCTCAAAATGGAATCTGTTCCAAACATATATGCCATGGTTCCTTACTTCGACAGGGTGCAAATATCTCAATTTC

TTCAAATCTCTCAAAATGGAATCTGTTCCAAACATATATGCCATGGTTCCTTACTTCGACAGGGTGCAAATATCTCAATTTC
 S  N  L  S  K  W  N  L  F  Q  T  Y  M  P  W  F  L  T  S  T  G  C  K  Y  L  N  F
TTCAAATCTCTCAAAATGGAATCTGTTCCAAACATATATGCCATGGTTCCTTACTTCGACAGGGTGCAAATATCTCAATTTC
 S  N  L  S  K  W  N  L  F  Q  T  Y  M  P  W  F  L  T  S  T  G  C  K  Y  L  N  F
TTCAAATCTCTCAAAATGGAATCTGTTCCAAACATATATGCCATGGTTCCTTACTTCGACAGGGTGCAAATATCTCAATTTC
 S  N  L  S  K  W  N  L  F  Q  T  Y  M  P  W  F  L  T  S  T  G  C  K  Y  L  N  F
TTCAAATCTCTCAAAATGGAATCTGTTCCAAACATATATGCCATGGTTCCTTACTTCGACAGGGTGCAAATATCTCAATTTC
 S  N  L  S  K  W  N  L  F  Q  T  Y  M  P  W  F  L  T  S  T  G  C  K  Y  L  N  F
TTCAAATCTCTCAAAATGGAATCTGTTCCAAACATATATGCCATGGTTCCTTACTTCGACAGGGTGCAAATATCTCAATTTC
 S  N  L  S  K  W  N  L  F  Q  T  Y  M  P  W  F  L  T  S  T  G  C  K  Y  L  N  F
TTCAAATCTCTCAAAATGGAATCTGTTCCAAACATATATGCCATGGTTCCTTACTTCGACAGGGTGCAAATATCTCAATTTC
 S  N  L  S  K  W  N  L  F  Q  T  Y  M  P  W  F  L  T  S  T  G  C  K  Y  L  N  F
TTCAAATCTCTCAAAATGGAATCTGTTCCAAACATATATGCCATGGTTCCTTACTTCGACAGGGTGCAAATATCTCAATTTC
 S  N  L  S  K  W  N  L  F  Q  T  Y  M  P  W  F  L  T  S  T  G  C  K  Y  L  N  F
TTCAAATCTCTCAAAATGGAATCTGTTCCAAACATATATGCCATGGTTCCTTACTTCGACAGGGTGCAAATATCTCAATTTC
 S  N  L  S  K  W  N  L  F  Q  T  Y  M  P  W  F  L  T  S  T  G  C  K  Y  L  N  F
TTCAAATCTCTCAAAATGGAATCTGTTCCAAACATATATGCCATGGTTCCTTACTTCGACAGGGTGCAAATATCTCAATTTC
 S  N  L  S  K  W  N  L  F  Q  T  Y  M  P  W  F  L  T  S  T  G  C  K  Y  L  N  F
TTCAAATCTCTCAAAATGGAATCTGTTCCAAACATATATGCCATGGTTCCTTACTTCGACAGGGTGCAAATATCTCAATTTC
 S  N  L  S  K  W  N  L  F  Q  T  Y  M  P  W  F  L  T  S  T  G  C  K  Y  L  N  F
TTCAAATCTCTCAAAATGGAATCTGTTCCAAACATATATGCCATGGTTCCTTACTTCGACAGGGTGCAAATATCTCAATTTC
 S  N  L  S  K  W  N  L  F  Q  T  Y  M  P  W  F  L  T  S  T  G  C  K  Y  L  N  F
TTCAAATCTCTCAAAATGGAATCTGTTCCAAACATATATGCCATGGTTCCTTACTTCGACAGGGTGCAAATATCTCAATTTC
 S  N  L  S  K  W  N  L  F  Q  T  Y  M  P  W  F  L  T  S  T  G  C  K  Y  L  N  F
TTCAAATCTCTCAAAATGGAATCTGTTCCAAACATATATGCCATGGTTCCTTACTTCGACAGGGTGCAAATATCTCAATTTC
 S  N  L  S  K  W  N  L  F  Q  T  Y  M  P  W  F  L  T  S  T  G  C  K  Y  L  N  F
TTCAAATCTCTCAAAATGGAATCTGTTCCAAACATATATGCCATGGTTCCTTACTTCGACAGGGTGCAAATATCTCAATTTC
 S  N  L  S  K  W  N  L  F  Q  T  Y  M  P  W  F  L  T  S  T  G  C  K  Y  L  N  F
TTCAAATCTCTCAAAATGGAATCTGTTCCAAACATATATGCCATGGTTCCTTACTTCGACAGGGTGCAAATATCTCAATTTC
 S  N  L  S  K  W  N  L  F  Q  T  Y  M  P  W  F  L  T  S  T  G  C  K  Y  L  N  F
TTCAAATCTCTCAAAATGGAATCTGTTCCAAACATATATGCCATGGTTCCTTACTTCGACAGGGTGCAAATATCTCAATTTC
 S  N  L  S  K  W  N  L  F  Q  T  Y  M  P  W  F  L  T  S  T  G  C  K  Y  L  N  F
TTCAAATCTCTCAAAATGGAATCTGTTCCAAACATATATGCCATGGTTCCTTACTTCGACAGGGTGCAAATATCTCAATTTC
 S  N  L  S  K  W  N  L  F  Q  T  Y  M  P  W  F  L  T  S  T  G  C  K  Y  L  N  F
TTCAAATCTCTCAAAATGGAATCTGTTCCAAACATATATGCCATCGTTCCTTACTTCGACAGGGTGCAAATATCTCAATTTC
 S  N  L  S  K  W  N  L  F  Q  T  Y  M  P  S  F  L  T  S  T  G  C  K  Y  L  N  F
TTCAAATCTCTCAAAATGGAATCTGTTCCAAACATATATGCCATGGTTCCTTACTTCGACAGGGTGCAAATATCTCAATTTC
 S  N  L  S  K  W  N  L  F  Q  T  Y  M  P  W  F  L  T  S  T  G  C  K  Y  L  N  F
TTCAAATCTCTCAAAATGGAATCTGTTCCAAACATATATGCCATGGTTCCTTACTTCGACAGGGTGCAAATATCTCAATTTC
 S  N  L  S  K  W  N  L  F  Q  T  Y  M  P  W  F  L  T  S  T  G  C  K  Y  L  N  F
TTCAAATCTCTCAAAATGGAATCTGTTCCAAACATATATGCCATGGTTCCTTACTTCGACAGGGTGCAAATATCTCAATTTC
 S  N  L  S  K  W  N  L  F  Q  T  Y  M  P  W  F  L  T  S  T  G  C  K  Y  L  N  F
TTCAAATCTCTCAAAATGGAATCTGTTCCAAACATATATGCCATGGTTCCTTACTTCGACAGGGTGCAAATATCTCAATTTC
 S  N  L  S  K  W  N  L  F  Q  T  Y  M  P  W  F  L  T  S  T  G  C  K  Y  L  N  F
TTCAAATCTCTCAAAATGGAATCTGTTCCAAACATATATGCCATGGTTCCTTACTTCGACAGGGTGCAAATATCTCAATTTC
 S  N  L  S  K  W  N  L  F  Q  T  Y  M  P  W  F  L  T  S  T  G  C  K  Y  L  N  F
```

```
3,810     3,820     3,830     3,840     3,850     3,860     3,870     3,880     3,890
ACCCTTTTAGATACTCTTTCAGACCCATTGCCGATACTGAGTAGTAGTCAAAAATTTGTATCCATTTTTCATGATATGATGCATG

ACCCTTTTAGATACTCTTTCAGACCCATTGCCGATACTGAGTAGTAGTCAAAAATTTGTATCCATTTTTCATGATATGATGCATG
T L L D T L S D P L P I L S S S Q K F V S I F H D M M H
ACCCTTTTAGATACTCTTTCAGACCCATTGCCGATACTGAGTAGTAGTCAAAAATTTGTATCCATTTTTCATGATATGATGCATG
T L L D T L S D P L P I L S S S Q K F V S I F H D M M H
ACCCTTTTAGATACTCTTTCAGACCCATTGCCGATACTGAGTAGTAGTCAAAAATTTGTATCCATTTTTCATGATATGATGCATG
T L L D T L S D P L P I L S S S Q K F V S I F H D M M H
ACCCTTTTAGATACTCTTTCAGACCCATTGCCGATACTGAGTAGTAGTCAAAAATTTGTATCCATTTTTCATGATATGATGCATG
T L L D T L S D P L P I L S S S Q K F V S I F H D M M H
ACCCTTTTAGATACTCTTTCAGACCCATTGCCGATACTGAGTAGTAGTCAAAAATTTGTATCCATTTTTCATGATATGATGCATG
T L L D T L S D P L P I L S S S Q K F V S I F H D M M H
ACCCTTTTAGATACTCTTTCAGACCCATTGCCGATACTGAGTAGTAGTCAAAAATTTGTATCCATTTTTCATGATATGATGCATG
T L L D T L S D P L P I L S S S Q K F V S I F H D M M H
ACCCTTTTAGATACTCTTTCAGACCCATTGCCGATACTGAGTAGTAGTCAAAAATTTGTATCCATTTTTCATGATATGATGCATG
T L L D T L S D P L P I L S S S Q K F V S I F H D M M H
ACCCTTTTAGATACTCTTTCAGACCCATTGCCGATACTGAGTAGTAGTCAAAAATTTGTATCCATTTTTCATGATATGATGCATG
T L L D T L S D P L P I L S S S Q K F V S I F H D M M H
ACCCTTTTAGATACTCTTTCAGACCCATTGCCGATACTGAGTAGTAGTCAAAAATTTGTATCCATTTTTCATGATATGATGCATG
T L L D T L S D P L P I L S S S Q K F V S I F H D M M H
ACCCTTTTAGATACTCTTTCAGACCCATTGCCGATACTGAGTAGTAGTCAAAAATTTGTATCCATTTTTCATGATATGATGCATG
T L L D T L S D P L P I L S S S Q K F V S I F H D M M H
ACCCTTTTAGATACTCTTTCAGACCCATTGCCGATACTGAGTAGTAGTCAAAAATTTGTATCCATTTTTCATGATATGATGCATG
T L L D T L S D P L P I L S S S Q K F V S I F H D M M H
ACCCTTTTAGATACTCTTTCAGACCCATTGCCGATACTGAGTAGTAGTCAAAAATTTGTATCCATTTTTCATGATATGATGCATG
T L L D T L S D P L P I L S S S Q K F V S I F H D M M H
ACCCTTTTAGATACTCTTTCAGACCCATTGCCGATACTGAGTAGTAGTCAAAAATTTGTATCCATTTTTCATGATATGATGCATG
T L L D T L S D P L P I L S S S Q K F V S I F H D M M H
ACCCTTTTAGATACTCTTTCAGACCCATTGCCGATACTGAGTAGTAGTCAAAAATTTGTATCCATTTTTCATGATATGATGCATG
T L L D T L S D P L P I L S S S Q K F V S I F H D M M H
ACCCTTTTAGATACTCTTTCAGACCCATTGCCGATACTGAGTAGTAGTCAAAAATTTGTATCCATTTTTCATGATATGATGCATG
T L L D T L S D P L P I L S S S Q K F V S I F H D M M H
ACCCTTTTAGATACTCTTTCAGACCCATTGCCGATACTGAGTAGTAGTCAAAAATTTGTATCCATTTTTCATGATATGATGCATG
T L L D T L S D P L P I L S S S Q K F V S I F H D M M H
ACCCTTTTAGATACTCTTTCAGACCCATTGCCGATACTGAGTAGTAGTCAAAAATTTGTATCCATTTTTCATGATATGATGCATG
T L L D T L S D P L P I L S S S Q K F V S I F H D M M H
ACCCTTTTAGATACTCTTTCAGACCCATTGCCGATACTGAGTAGTAGTCAAAAATTTGTATCCATTTTTCATGATATGATGCATG
T L L D T L S D P L P I L S S S Q K F V S I F H D M M H
ACCCTTTTAGATACTCTTTCAGACCCATTGCCGATACTGAGTAGTAGTCAAAAATTTGTATCCATTTTTCATGATATGATGCATG
T L L D T L S D P L P I L S S S Q K F V S I F H D M M H
ACCCTTTTAGATACTCTTTCAGACCCATTGCCGATACTGAGTAGTAGTCAAAAATTTGTATCCATTTTTCATGATATGATGCATG
T L L D T L S D P L P I L S S S Q K F V S I F H D M M H
ACCCTTTTAGATACTCTTTCAGACCCATTGCCGATACTGAGTAGTAGTCAAAAATTTGTATCCATTTTTCATGATATGATGCATG
T L L D T L S D P L P I L S S S Q K F V S I F H D M M H
ACCCTTTTAGATACTCTTTCAGACCCATTGCCGATACTGAGTAGTAGTCAAAAATTTGTATCCATTTTTCATGATATGATGCATG
T L L D T L S D P L P I L S S S Q K F V S I F H D M M H
ACCCTTTTAGATACTCTTTCAGACCCATTGCCGATACTGAGTAGTAGTCAAAAATTTGTATCCATTTTTCATGATATGATGCATG
T L L D T L S D P L P I L S S S Q K F V S I F H D M M H
```

```
3,900 3,910 3,920 3,930 3,940 3,950 3,960 3,970
GATCAGATATATCACGGCCAATTCCTCAGAAGATTCTTCCACAATGGACTCTGATAAGTGAGATTTCGAGTCAATGTTTACA

GATCAGATATATCACGGCCAATTCCTCAGAAGATTCTTCCACAATGGACTCTGATAAGTGAGATTTCGAGTCAATGTTTACA
G S D I S R P I P Q K I L P Q W T L I S E I S S Q C L Q
GATCAGATATATCACGGCCAATTCCTCAGAAGATTCTTCCACAATGGACTCTGATAAGTGAGATTTCGAGTCAATGTTTACA
G S D I S R P I P Q K I L P Q W T L I S E I S S Q C L Q
GATCAGATATATCACGGCCAATTCCTCAGAAGATTCTTCCACAATGGACTCTGATAAGTGAGATTTCGAGTCAATGTTTACA
G S D I S R P I P Q K I L P Q W T L I S E I S S Q C L Q
GATCAGATATATCACGGCCAATTCCTCAGAAGATTCTTCCACAATGGACTCTGATAAGTGAGATTTCGAGTCAATGTTTACA
G S D I S R P I P Q K I L P Q W T L I S E I S S Q C L Q
GATCAGATATATCACGGCCAATTCCTCAGAAGATTCTTCCACAATGGACTCTGATAAGTGAGATTTCGAGTCAATGTTTACA
G S D I S R P I P Q K I L P Q W T L I S E I S S Q C L Q
GATCAGATATATCACGGCCAATTCCTCAGAAGATTCTTCCACAATGGACTCTGATAAGTGAGATTTCGAGTCAATGTTTACA
G S D I S R P I P Q K I L P Q W T L I S E I S S Q C L Q
GATCAGATATATCACGGCCAATTCCTCAGAAGATTCTTCCACAATGGACTCTGATAAGTGAGATTTCGAGTCAATGTTTACA
G S D I S R P I P Q K I L P Q W T L I S E I S S Q C L Q
GATCAGATATATCACGGCCAATTCCTCAGAAGATTCTTCCACAATGGACTCTGATAAGTGAGATTTCGAGTCAATGTTTACA
G S D I S R P I P Q K I L P Q W T L I S E I S S Q C L Q
GATCAGATATATCACGGCCAATTCCTCAGAAGATTCTTCCACAATGGACTCTGATAAGTGAGATTTCGAGTCAATGTTTACA
G S D I S R P I P Q K I L P Q W T L I S E I S S Q C L Q
GATCAGATATATCACGGCCAATTCCTCAGAAGATTCTTCCACAATGGACTCTGATAAGTGAGATTTCGAGTCAATGTTTACA
G S D I S R P I P Q K I L P Q W T L I S E I S S Q C L Q
GATCAGATATATCACGGCCAATTCCTCAGAAGATTCTTCCACAATGGACTCTGATAAGTGAGATTTCGAGTCAATGTTTACA
G S D I S R P I P Q K I L P Q W T L I S E I S S Q C L Q
GATCAGATATATCACGGCCAATTCCTCAGAAGATTCTTCCACAATGGACTCTGATAAGTGAGATTTCGAGTCAATGTTTACA
G S D I S R P I P Q K I L P Q W T L I S E I S S Q C L Q
GATCAGATATATCACGGCCAATTCCTCAGAAGATTCTTCCACAATGGACTCTGATAAGTGAGATTTCGAGTCAATGTTTACA
G S D I S R P I P Q K I L P Q W T L I S E I S S Q C L Q
GATCAGATATATCACGGCCAATTCCTCAGAAGATTCTTCCACAATGGACTCTGATAAGTGAGATTTCGAGTCAATGTTTACA
G S D I S R P I P Q K I L P Q W T L I S E I S S Q C L Q
GATCAGATATATCACGGCCAATTCCTCAGAAGATTCTTCCACAATGGACTCTGATAAGTGAGATTTCGAGTCAATGTTTACA
G S D I S R P I P Q K I L P Q W T L I S E I S S Q C L Q
GATCAGATATATCACGGCCAATTCCTCAGAAGATTCTTCCACAATGGACTCTGATAAGTGAGATTTCGAGTCAATGTTTACA
G S D I S R P I P Q K I L P Q W T L I S E I S S Q C L Q
GATCAGATATATCACGGCCAATTCCTCAGAAGATTCTTCCACAATGGACTCTGATAAGTGAGATTTCGAGTCAATGTTTACA
G S D I S R P I P Q K I L P Q W T L I S E I S S Q C L Q
GATCAGATATATCACGGCCAATTCCTCAGAAGATTCTTCCACAATGGACTCTGATAAGTGAGATTTCGAGTCAATGTTTACA
G S D I S R P I P Q K I L P Q W T L I S E I S S Q C L Q
GATCAGATATATCACGGCCAATTCCTCAGAAGATTCTTCCACAATGGACTCTGATAAGTGAGATTTCGAGTCAATGTTTACA
G S D I S R P I P Q K I L P Q W T L I S E I S S Q C L Q
GATCAGATATATCACGGCCAATTCCTCAGAAGATTCTTCCACAATGGACTCTGATAAGTGAGATTTCGAGTCAATGTTTACA
G S D I S R P I P Q K I L P Q W T L I S E I S S Q C L Q
GATCAGATATATCACGGCCAATTCCTCAGAAGATTCTTCCACAATGGACTCTGATAAGTGAGATTTCGAGTCAATGTTTACA
G S D I S R P I P Q K I L P Q W T L I S E I S S Q C L Q
GATCAGATATATCACGGCCAATTCCTCAGAAGATTCTTCCACAATGGACTCTGATAAGTGAGATTTCGAGTCAATGTTTACA
G S D I S R P I P Q K I L P Q W T L I S E I S S Q C L Q
GATCAGATATATCACGGCCAATTCCTCAGAAGATTCTTCCACAATGGACTCTGATAAGTGAGATTTCGAGTCAATGTTTACA
G S D I S R P I P Q K I L P Q W T L I S E I S S Q C L Q
```

3,980 3,990 4,000 4,010 4,020 4,030 4,040 4,050

GAATCTTCTTCTGTCCGAAGAAATGATTCATCGAAATAATGAGTCACCCGTTCCATTGATATGGGCACATCTGAGATCAAC

GAATCTTCTTCTGTCCGAAGAAATGATTCATCGAAATAATGAGTCACCCGTTCCATTGATATGGGCACATCTGAGATCAAC
N L L L S E E M I H R N N E S P V P L I W A H L R S T

GAATCTTCTTCTGTCCGAAGAAATGATTCATCGAAATAATGAGTCACCCGTTCCATTGATATGGGCACATCTGAGATCAAC
N L L L S E E M I H R N N E S P V P L I W A H L R S T

GAATCTTCTTCTGTCCGAAGAAATGATTCATCGAAATAATGAGTCACCCGTTCCATTGATATGGGCACATCTGAGATCAAC
N L L L S E E M I H R N N E S P V P L I W A H L R S T

GAATCTTCTTCTGTCCGAAGAAATGATTCATCGAAATAATGAGTCACCCGTTCCATTGATATGGGCACATCTGAGATCAAC
N L L L S E E M I H R N N E S P V P L I W A H L R S T

GAATCTTCTTCTGTCCGAAGAAATGATTCATCGAAATAATGAGTCACCCGTTCCATTGATATGGGCACATCTGAGATCAAC
N L L L S E E M I H R N N E S P V P L I W A H L R S T

GAATCTTCTTCTGTCCGAAGAAATGATTCATCGAAATAATGAGTCACCCGTTCCATTGATATGGGCACATCTGAGATCAAC
N L L L S E E M I H R N N E S P V P L I W A H L R S T

GAATCTTCTTCTGTCCGAAGAAATGATTCATCGAAATAATGAGTCACCCGTTCCATTGATATGGGCACATCTGAGATCAAC
N L L L S E E M I H R N N E S P V P L I W A H L R S T

GAATCTTCTTCTGTCCGAAGAAATGATTCATCGAAATAATGAGTCACCCGTTCCATTGATATGGGCACATCTGAGATCAAC
N L L L S E E M I H R N N E S P V P L I W A H L R S T

GAATCTTCTTCTGTCCGAAGAAATGATTCATCGAAATAATGAGTCACCCGTTCCATTGATATGGGCACATCTGAGATCAAC
N L L L S E E M I H R N N E S P V P L I W A H L R S T

GAATCTTCTTCTGTCCGAAGAAATGATTCATCGAAATAATGAGTCACCCGTTCCATTGATATGGGCACATCTGAGATCAAC
N L L L S E E M I H R N N E S P V P L I W A H L R S T

GAATCTTCTTCTGTCCGAAGAAATGATTCATCGAAATAATGAGTCACCCGTTCCATTGATATGGGCACATCTGAGATCAAC
N L L L S E E M I H R N N E S P V P L I W A H L R S T

GAATCTTCTTCTGTCCGAAGAAATGATTCATCGAAATAATGAGTCACCCGTTCCATTGATATGGGCACATCTGAGATCAAC
N L L L S E E M I H R N N E S P V P L I W A H L R S T

GAATCTTCTTCTGTCCGAAGAAATGATTCATCGAAATAATGAGTCACCCGTTCCATTGATATGGGCACATCTGAGATCAAC
N L L L S E E M I H R N N E S P V P L I W A H L R S T

GAATCTTCTTCTGTCCGAAGAAATGATTCATCGAAATAATGAGTCACCCGTTCCATTGATATGGGCACATCTGAGATCAAC
N L L L S E E M I H R N N E S P V P L I W A H L R S T

GAATCTTCTTCTGTCCGAAGAAATGATTCATCGAAATAATGAGTCACCCGTTCCATTGATATGGGCACATCTGAGATCAAC
N L L L S E E M I H R N N E S P V P L I W A H L R S T

GAATCTTCTTCTGTCCGAAGAAATGATTCATCGAAATAATGAGTCACCCGTTCCATTGATATGGGCACATCTGAGATCAAC
N L L L S E E M I H R N N E S P V P L I W A H L R S T

GAATCTTCTTCTGTCCGAAGAAATGATTCATCGAAATAATGAGTCACCCGTTCCATTGATATGGGCACATCTGAGATCAAC
N L L L S E E M I H R N N E S P V P L I W A H L R S T

GAATCTTCTTCTGTCCGAAGAAATGATTCATCGAAATAATGAGTCACCCGTTCCATTGATATGGGCACATCTGAGATCAAC
N L L L S E E M I H R N N E S P V P L I W A H L R S T

GAATCTTCTTCTGTCCGAAGAAATGATTCATCGAAATAATGAGTCACCCGTTCCATTGATATGGGCACATCTGAGATCAAC
N L L L S E E M I H R N N E S P V P L I W A H L R S T

GAATCTTCTTCTGTCCGAAGAAATGATTCATCGAAATAATGAGTCACCCGTTCCATTGATATGGGCACATCTGAGATCAAC
N L L L S E E M I H R N N E S P V P L I W A H L R S T

GAATCTTCTTCTGTCCGAAGAAATGATTCATCGAAATAATGAGTCACCCGTTCCATTGATATGGGCACATCTGAGATCAAC
N L L L S E E M I H R N N E S P V P L I W A H L R S T

GAATCTTCTTCTGTCCGAAGAAATGATTCATCGAAATAATGAGTCACCCGTTCCATTGATATGGGCACATCTGAGATCAAC
N L L L S E E M I H R N N E S P V P L I W A H L R S T

GAATCTTCTTCTGTCCGAAGAAATGATTCATCGAAATAATGAGTCACCCGTTCCATTGATATGGGCACATCTGAGATCAAC
N L L L S E E M I H R N N E S P V P L I W A H L R S T

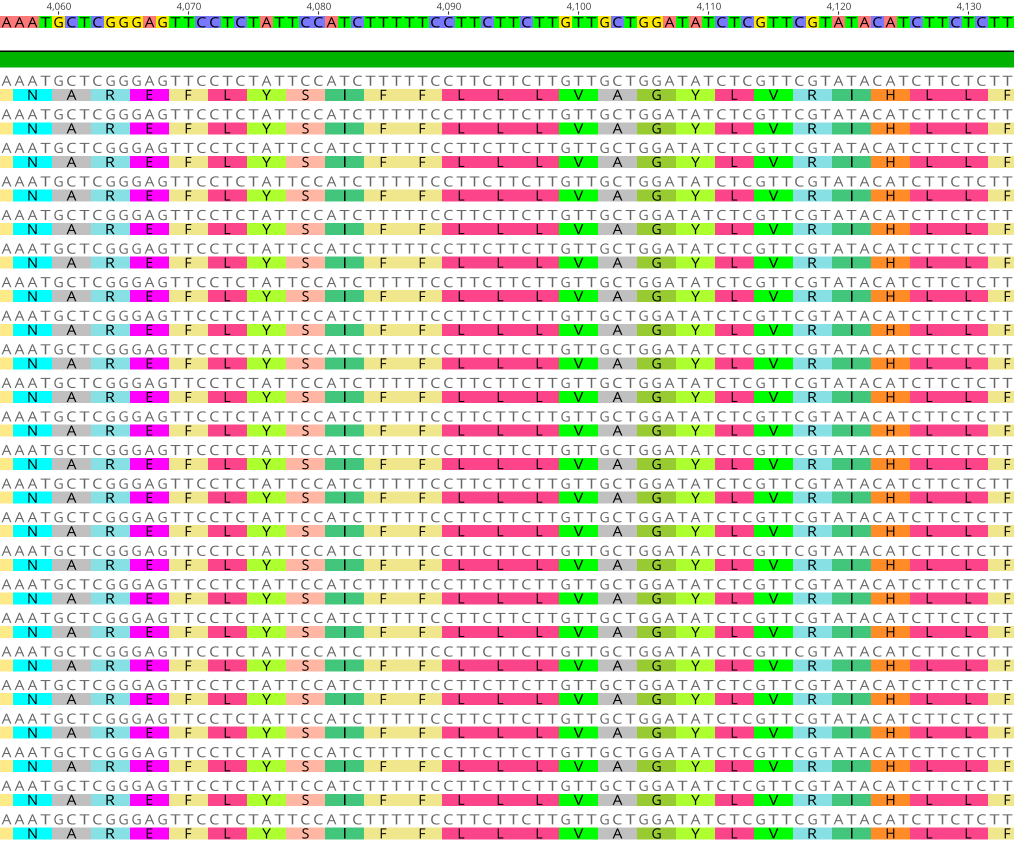
4,060 4,070 4,080 4,090 4,100 4,110 4,120 4,130
AAATGCTCGGGAGTTCCTCTATTCCATCTTTTTCCTTCTTCTTGTTGCTGGATATCTCGTTCGTATACATCTTCTCTT
AAATGCTCGGGAGTTCCTCTATTCCATCTTTTTCCTTCTTCTTGTTGCTGGATATCTCGTTCGTATACATCTTCTCTT
N A R E F L Y S I F F L L L V A G Y L V R I H L L F
AAATGCTCGGGAGTTCCTCTATTCCATCTTTTTCCTTCTTCTTGTTGCTGGATATCTCGTTCGTATACATCTTCTCTT
N A R E F L Y S I F F L L L V A G Y L V R I H L L F
AAATGCTCGGGAGTTCCTCTATTCCATCTTTTTCCTTCTTCTTGTTGCTGGATATCTCGTTCGTATACATCTTCTCTT
N A R E F L Y S I F F L L L V A G Y L V R I H L L F
AAATGCTCGGGAGTTCCTCTATTCCATCTTTTTCCTTCTTCTTGTTGCTGGATATCTCGTTCGTATACATCTTCTCTT
N A R E F L Y S I F F L L L V A G Y L V R I H L L F
AAATGCTCGGGAGTTCCTCTATTCCATCTTTTTCCTTCTTCTTGTTGCTGGATATCTCGTTCGTATACATCTTCTCTT
N A R E F L Y S I F F L L L V A G Y L V R I H L L F
AAATGCTCGGGAGTTCCTCTATTCCATCTTTTTCCTTCTTCTTGTTGCTGGATATCTCGTTCGTATACATCTTCTCTT
N A R E F L Y S I F F L L L V A G Y L V R I H L L F
AAATGCTCGGGAGTTCCTCTATTCCATCTTTTTCCTTCTTCTTGTTGCTGGATATCTCGTTCGTATACATCTTCTCTT
N A R E F L Y S I F F L L L V A G Y L V R I H L L F
AAATGCTCGGGAGTTCCTCTATTCCATCTTTTTCCTTCTTCTTGTTGCTGGATATCTCGTTCGTATACATCTTCTCTT
N A R E F L Y S I F F L L L V A G Y L V R I H L L F
AAATGCTCGGGAGTTCCTCTATTCCATCTTTTTCCTTCTTCTTGTTGCTGGATATCTCGTTCGTATACATCTTCTCTT
N A R E F L Y S I F F L L L V A G Y L V R I H L L F
AAATGCTCGGGAGTTCCTCTATTCCATCTTTTTCCTTCTTCTTGTTGCTGGATATCTCGTTCGTATACATCTTCTCTT
N A R E F L Y S I F F L L L V A G Y L V R I H L L F
AAATGCTCGGGAGTTCCTCTATTCCATCTTTTTCCTTCTTCTTGTTGCTGGATATCTCGTTCGTATACATCTTCTCTT
N A R E F L Y S I F F L L L V A G Y L V R I H L L F
AAATGCTCGGGAGTTCCTCTATTCCATCTTTTTCCTTCTTCTTGTTGCTGGATATCTCGTTCGTATACATCTTCTCTT
N A R E F L Y S I F F L L L V A G Y L V R I H L L F
AAATGCTCGGGAGTTCCTCTATTCCATCTTTTTCCTTCTTCTTGTTGCTGGATATCTCGTTCGTATACATCTTCTCTT
N A R E F L Y S I F F L L L V A G Y L V R I H L L F
AAATGCTCGGGAGTTCCTCTATTCCATCTTTTTCCTTCTTCTTGTTGCTGGATATCTCGTTCGTATACATCTTCTCTT
N A R E F L Y S I F F L L L V A G Y L V R I H L L F
AAATGCTCGGGAGTTCCTCTATTCCATCTTTTTCCTTCTTCTTGTTGCTGGATATCTCGTTCGTATACATCTTCTCTT
N A R E F L Y S I F F L L L V A G Y L V R I H L L F
AAATGCTCGGGAGTTCCTCTATTCCATCTTTTTCCTTCTTCTTGTTGCTGGATATCTCGTTCGTATACATCTTCTCTT
N A R E F L Y S I F F L L L V A G Y L V R I H L L F
AAATGCTCGGGAGTTCCTCTATTCCATCTTTTTCCTTCTTCTTGTTGCTGGATATCTCGTTCGTATACATCTTCTCTT
N A R E F L Y S I F F L L L V A G Y L V R I H L L F
AAATGCTCGGGAGTTCCTCTATTCCATCTTTTTCCTTCTTCTTGTTGCTGGATATCTCGTTCGTATACATCTTCTCTT
N A R E F L Y S I F F L L L V A G Y L V R I H L L F
AAATGCTCGGGAGTTCCTCTATTCCATCTTTTTCCTTCTTCTTGTTGCTGGATATCTCGTTCGTATACATCTTCTCTT
N A R E F L Y S I F F L L L V A G Y L V R I H L L F
AAATGCTCGGGAGTTCCTCTATTCCATCTTTTTCCTTCTTCTTGTTGCTGGATATCTCGTTCGTATACATCTTCTCTT
N A R E F L Y S I F F L L L V A G Y L V R I H L L F
AAATGCTCGGGAGTTCCTCTATTCCATCTTTTTCCTTCTTCTTGTTGCTGGATATCTCGTTCGTATACATCTTCTCTT
N A R E F L Y S I F F L L L V A G Y L V R I H L L F
AAATGCTCGGGAGTTCCTCTATTCCATCTTTTTCCTTCTTCTTGTTGCTGGATATCTCGTTCGTATACATCTTCTCTT
N A R E F L Y S I F F L L L V A G Y L V R I H L L F
AAATGCTCGGGAGTTCCTCTATTCCATCTTTTTCCTTCTTCTTGTTGCTGGATATCTCGTTCGTATACATCTTCTCTT
N A R E F L Y S I F F L L L V A G Y L V R I H L L F

```
4,140     4,150     4,160     4,170     4,180     4,190     4,200     4,210
TGTTTCCCGAGCCTCTAGTGAGTTACAGACAGAGTTAGAAAAGATCAAATCTTTGATGATTCCATCATACATGATGGAATT

TGTTTCCCGAGCCTCTAGTGAGTTACAGACAGAGTTAGAAAAGATCAAATCTTTGATGATTCCATCATACATGATGGAATT
 V  S  R  A  S  S  E  L  Q  T  E  L  E  K  I  K  S  L  M  I  P  S  Y  M  M  E  F
TGTTTCCCGAGCCTCTAGTGAGTTACAGACAGAGTTAGAAAAGATCAAATCTTTGATGATTCCATCATACATGATGGAATT
 V  S  R  A  S  S  E  L  Q  T  E  L  E  K  I  K  S  L  M  I  P  S  Y  M  M  E  F
TGTTTCCCGAGCCTCTAGTGAGTTACAGACAGAGTTAGAAAAGATCAAATCTTTGATGATTCCATCATACATGATGGAATT
 V  S  R  A  S  S  E  L  Q  T  E  L  E  K  I  K  S  L  M  I  P  S  Y  M  M  E  F
TGTTTCCCGAGCCTCTAGTGAGTTACAGACAGAGTTAGAAAAGATCAAATCTTTGATGATTCCATCATACATGATGGAATT
 V  S  R  A  S  S  E  L  Q  T  E  L  E  K  I  K  S  L  M  I  P  S  Y  M  M  E  F
TGTTTCCCGAGCCTCTAGTGAGTTACAGACAGAGTTAGAAAAGATCAAATCTTTGATGATTCCATCATACATGATGGAATT
 V  S  R  A  S  S  E  L  Q  T  E  L  E  K  I  K  S  L  M  I  P  S  Y  M  M  E  F
TGTTTCCCGAGCCTCTAGTGAGTTACAGACAGAGTTAGAAAAGATCAAATCTTTGATGATTCCATCATACATGATGGAATT
 V  S  R  A  S  S  E  L  Q  T  E  L  E  K  I  K  S  L  M  I  P  S  Y  M  M  E  F
TGTTTCCCGAGCCTCTAGTGAGTTACAGACAGAGTTAGAAAAGATCAAATCTTTGATGATTCCATCATACATGATGGAATT
 V  S  R  A  S  S  E  L  Q  T  E  L  E  K  I  K  S  L  M  I  P  S  Y  M  M  E  F
TGTTTCCCGAGCCTCTAGTGAGTTACAGACAGAGTTAGAAAAGATCAAATCTTTGATGATTCCATCATACATGATGGAATT
 V  S  R  A  S  S  E  L  Q  T  E  L  E  K  I  K  S  L  M  I  P  S  Y  M  M  E  F
TGTTTCCCGAGCCTCTAGTGAGTTACAGACAGAGTTAGAAAAGATCAAATCTTTGATGATTCCATCATACATGATGGAATT
 V  S  R  A  S  S  E  L  Q  T  E  L  E  K  I  K  S  L  M  I  P  S  Y  M  M  E  F
TGTTTCCCGAGCCTCTAGTGAGTTACAGACAGAGTTAGAAAAGATCAAATCTTTGATGATTCCATCATACATGATGGAATT
 V  S  R  A  S  S  E  L  Q  T  E  L  E  K  I  K  S  L  M  I  P  S  Y  M  M  E  F
TGTTTCCCGAGCCTCTAGTGAGTTACAGACAGAGTTAGAAAAGATCAAATCTTTGATGATTCCATCATACATGATGGAATT
 V  S  R  A  S  S  E  L  Q  T  E  L  E  K  I  K  S  L  M  I  P  S  Y  M  M  E  F
TGTTTCCCGAGCCTCTAGTGAGTTACAGACAGAGTTAGAAAAGATCAAATCTTTGATGATTCCATCATACATGATGGAATT
 V  S  R  A  S  S  E  L  Q  T  E  L  E  K  I  K  S  L  M  I  P  S  Y  M  M  E  F
TGTTTCCCGAGCCTCTAGTGAGTTACAGACAGAGTTAGAAAAGATCAAATCTTTGATGATTCCATCATACATGATGGAATT
 V  S  R  A  S  S  E  L  Q  T  E  L  E  K  I  K  S  L  M  I  P  S  Y  M  M  E  F
TGTTTCCCGAGCCTCTAGTGAGTTACAGACAGAGTTAGAAAAGATCAAATCTTTGATGATTCCATCATACATGATGGAATT
 V  S  R  A  S  S  E  L  Q  T  E  L  E  K  I  K  S  L  M  I  P  S  Y  M  M  E  F
TGTTTCCCGAGCCTCTAGTGAGTTACAGACAGAGTTAGAAAAGATCAAATCTTTGATGATTCCATCATACATGATGGAATT
 V  S  R  A  S  S  E  L  Q  T  E  L  E  K  I  K  S  L  M  I  P  S  Y  M  M  E  F
TGTTTCCCGAGCCTCTAGTGAGTTACAGACAGAGTTAGAAAAGATCAAATCTTTGATGATTCCATCATACATGATGGAATT
 V  S  R  A  S  S  E  L  Q  T  E  L  E  K  I  K  S  L  M  I  P  S  Y  M  M  E  F
TGTTTCCCGAGCCTCTAGTGAGTTACAGACAGAGTTAGAAAAGATCAAATCTTTGATGATTCCATCATACATGATGGAATT
 V  S  R  A  S  S  E  L  Q  T  E  L  E  K  I  K  S  L  M  I  P  S  Y  M  M  E  F
TGTTTCCCGAGCCTCTAGTGAGTTACAGACAGAGTTAGAAAAGATCAAATCTTTGATGATTCCATCATACATGATGGAATT
 V  S  R  A  S  S  E  L  Q  T  E  L  E  K  I  K  S  L  M  I  P  S  Y  M  M  E  F
TGTTTCCCGAGCCTCTAGTGAGTTACAGACAGAGTTAGAAAAGATCAAATCTTTGATGATTCCATCATACATGATGGAATT
 V  S  R  A  S  S  E  L  Q  T  E  L  E  K  I  K  S  L  M  I  P  S  Y  M  M  E  F
TGTTTCCCGAGCCTCTAGTGAGTTACAGACAGAGTTAGAAAAGATCAAATCTTTGATGATTCCATCATACATGATGGAATT
 V  S  R  A  S  S  E  L  Q  T  E  L  E  K  I  K  S  L  M  I  P  S  Y  M  M  E  F
TGTTTCCCGAGCCTCTAGTGAGTTACAGACAGAGTTAGAAAAGATCAAATCTTTGATGATTCCATCATACATGATGGAATT
 V  S  R  A  S  S  E  L  Q  T  E  L  E  K  I  K  S  L  M  I  P  S  Y  M  M  E  F
TGTTTCCCGAGCCTCTAGTGAGTTACAGACAGAGTTAGAAAAGATCAAATCTTTGATGATTCCATCATACATGATGGAATT
 V  S  R  A  S  S  E  L  Q  T  E  L  E  K  I  K  S  L  M  I  P  S  Y  M  M  E  F
TGTTTCCCGAGCCTCTAGTGAGTTACAGACAGAGTTAGAAAAGATCAAATCTTTGATGATTCCATCATACATGATGGAATT
 V  S  R  A  S  S  E  L  Q  T  E  L  E  K  I  K  S  L  M  I  P  S  Y  M  M  E  F
```

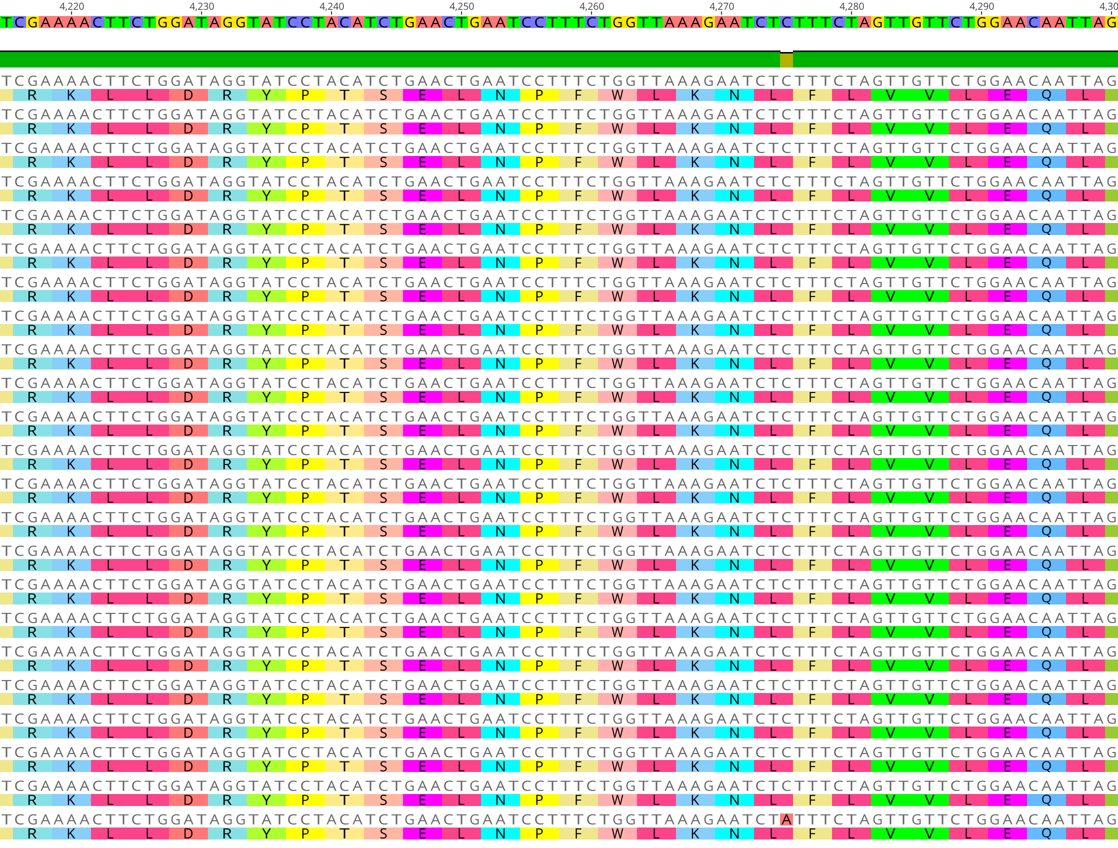
4,220 4,230 4,240 4,250 4,260 4,270 4,280 4,290 4,30
TCGAAAACTTCTGGATAGGTATCCTACATCTGAACTGAATCCTTTCTGGTTAAAGAATCTCTTTCTAGTTGTTCTGGAACAATTAG
R K L L D R Y P T S E L N P F W L K N L F L V V L E Q L
TCGAAAACTTCTGGATAGGTATCCTACATCTGAACTGAATCCTTTCTGGTTAAAGAATCTATTTCTAGTTGTTCTGGAACAATTAG

```
0        4,310     4,320     4,330     4,340     4,350     4,360     4,370     4,380
GAGATTCTCTGGAAGAAATACGGGGTTCTGCTTCTGGTGGCAACATGCTATTGGGTGGTGGTCCCGCTTATGGGGTCAAATCAATAC

GAGATTCTCTGGAAGAAATACGGGGTTCTGCTTCTGGTGGCAACATGCTATTGGGTGGTGGTCCCGCTTATGGGGTCAAATCAATAC
G  D  S  L  E  E  I  R  G  S  A  S  G  G  N  M  L  L  G  G  G  P  A  Y  G  V  K  S  I
GAGATTCTCTGGAAGAAATACGGGGTTCTGCTTCTGGTGGCAACATGCTATTGGGTGGTGGTCCCGCTTATGGGGTCAAATCAATAC
G  D  S  L  E  E  I  R  G  S  A  S  G  G  N  M  L  L  G  G  G  P  A  Y  G  V  K  S  I
GAGATTCTCTGGAAGAAATACGGGGTTCTGCTTCTGGTGGCAACATGCTATTGGGTGGTGGTCCCGCTTATGGGGTCAAATCAATAC
G  D  S  L  E  E  I  R  G  S  A  S  G  G  N  M  L  L  G  G  G  P  A  Y  G  V  K  S  I
GAGATTCTCTGGAAGAAATACGGGGTTCTGCTTCTGGTGGCAACATGCTATTGGGTGGTGGTCCCGCTTATGGGGTCAAATCAATAC
G  D  S  L  E  E  I  R  G  S  A  S  G  G  N  M  L  L  G  G  G  P  A  Y  G  V  K  S  I
GAGATTCTCTGGAAGAAATACGGGGTTCTGCTTCTGGTGGCAACATGCTATTGGGTGGTGGTCCCGCTTATGGGGTCAAATCAATAC
G  D  S  L  E  E  I  R  G  S  A  S  G  G  N  M  L  L  G  G  G  P  A  Y  G  V  K  S  I
GAGATTCTCTGGAAGAAATACGGGGTTCTGCTTCTGGTGGCAACATGCTATTGGGTGGTGGTCCCGCTTATGGGGTCAAATCAATAC
G  D  S  L  E  E  I  R  G  S  A  S  G  G  N  M  L  L  G  G  G  P  A  Y  G  V  K  S  I
GAGATTCTCTGGAAGAAATACGGGGTTCTGCTTCTGGTGGCAACATGCTATTGGGTGGTGGTCCCGCTTATGGGGTCAAATCAATAC
G  D  S  L  E  E  I  R  G  S  A  S  G  G  N  M  L  L  G  G  G  P  A  Y  G  V  K  S  I
GAGATTCTCTGGAAGAAATACGGGGTTCTGCTTCTGGTGGCAACATGCTATTGGGTGGTGGTCCCGCTTATGGGGTCAAATCAATAC
G  D  S  L  E  E  I  R  G  S  A  S  G  G  N  M  L  L  G  G  G  P  A  Y  G  V  K  S  I
GAGATTCTCTGGAAGAAATACGGGGTTCTGCTTCTGGTGGCAACATGCTATTGGGTGGTGGTCCCGCTTATGGGGTCAAATCAATAC
G  D  S  L  E  E  I  R  G  S  A  S  G  G  N  M  L  L  G  G  G  P  A  Y  G  V  K  S  I
GAGATTCTCTGGAAGAAATACGGGGTTCTGCTTCTGGTGGCAACATGCTATTGGGTGGTGGTCCCGCTTATGGGGTCAAATCAATAC
G  D  S  L  E  E  I  R  G  S  A  S  G  G  N  M  L  L  G  G  G  P  A  Y  G  V  K  S  I
GAGATTCTCTGGAAGAAATACGGGGTTCTGCTTCTGGTGGCAACATGCTATTGGGTGGTGGTCCCGCTTATGGGGTCAAATCAATAC
G  D  S  L  E  E  I  R  G  S  A  S  G  G  N  M  L  L  G  G  G  P  A  Y  G  V  K  S  I
GAGATTCTCTGGAAGAAATACGGGGTTCTGCTTCTGGTGGCAACATGCTATTGGGTGGTGGTCCCGCTTATGGGGTCAAATCAATAC
G  D  S  L  E  E  I  R  G  S  A  S  G  G  N  M  L  L  G  G  G  P  A  Y  G  V  K  S  I
GAGATTCTCTGGAAGAAATACGGGGTTCTGCTTCTGGTGGCAACATGCTATTGGGTGGTGGTCCCGCTTATGGGGTCAAATCAATAC
G  D  S  L  E  E  I  R  G  S  A  S  G  G  N  M  L  L  G  G  G  P  A  Y  G  V  K  S  I
GAGATTCTCTGGAAGAAATACGGGGTTCTGCTTCTGGTGGCAACATGCTATTGGGTGGTGGTCCCGCTTATGGGGTCAAATCAATAC
G  D  S  L  E  E  I  R  G  S  A  S  G  G  N  M  L  L  G  G  G  P  A  Y  G  V  K  S  I
GAGATTCTCTGGAAGAAATACGGGGTTCTGCTTCTGGTGGCAACATGCTATTGGGTGGTGGTCCCGCTTATGGGGTCAAATCAATAC
G  D  S  L  E  E  I  R  G  S  A  S  G  G  N  M  L  L  G  G  G  P  A  Y  G  V  K  S  I
GAGATTCTCTGGAAGAAATACGGGGTTCTGCTTCTGGTGGCAACATGCTATTGGGTGGTGGTCCCGCTTATGGGGTCAAATCAATAC
G  D  S  L  E  E  I  R  G  S  A  S  G  G  N  M  L  L  G  G  G  P  A  Y  G  V  K  S  I
GAGATTCTCTGGAAGAAATACGGGGTTCTGCTTCTGGTGGCAACATGCTATTGGGTGGTGGTCCCGCTTATGGGGTCAAATCAATAC
G  D  S  L  E  E  I  R  G  S  A  S  G  G  N  M  L  L  G  G  G  P  A  Y  G  V  K  S  I
GAGATTCTCTGGAAGAAATACGGGGTTCTGCTTCTGGTGGCAACATGCTATTGGGTGGTGGTCCCGCTTATGGGGTCAAATCAATAC
G  D  S  L  E  E  I  R  G  S  A  S  G  G  N  M  L  L  G  G  G  P  A  Y  G  V  K  S  I
GAGATTCTCTGGAAGAAATACGGGGTTCTGCTTCTGGTGGCAACATGCTATTGGGTGGTGGTCCCGCTTATGGGGTCAAATCAATAC
G  D  S  L  E  E  I  R  G  S  A  S  G  G  N  M  L  L  G  G  G  P  A  Y  G  V  K  S  I
GAGATTCTCTGGAAGAAATACGGGGTTCTGCTTCTGGTGGCAACATGCTATTGGGTGGTGGTCCCGCTTATGGGGTCAAATCAATAC
G  D  S  L  E  E  I  R  G  S  A  S  G  G  N  M  L  L  G  G  G  P  A  Y  G  V  K  S  I
GAGATTCTCTGGAAGAAATACGGGGTTCTGCTTCTGGTGGCAACATGCTATTGGGTGGTGGTCCCGCTTATGGGGTCAAATCAATAC
G  D  S  L  E  E  I  R  G  S  A  S  G  G  N  M  L  L  G  G  G  P  A  Y  G  V  K  S  I
GAGATTCTCTGGAAGAAATACGGGGTTCTGCTTCTGGTGGCAACATGCTATTGGGTGGTGGTCCCGCTTATGGGGTCAAATCAATAC
G  D  S  L  E  E  I  R  G  S  A  S  G  G  N  M  L  L  G  G  G  P  A  Y  G  V  K  S  I
GAGATTCTCTGGAAGAAATACGGGGTTCTGCTTCTGGTGGCAACATGCTATTGGGTGGTGGTCCCGCTTATGGGGTCAAATCAATAC
G  D  S  L  E  E  I  R  G  S  A  S  G  G  N  M  L  L  G  G  G  P  A  Y  G  V  K  S  I
GAGATTCTCTGGAAGAAATACGGGGTTCTGCTTCTGGTGGCAACATGCTATTGGGTGGTGGTCCCGCTTATGGGGTCAAATCAATAC
G  D  S  L  E  E  I  R  G  S  A  S  G  G  N  M  L  L  G  G  G  P  A  Y  G  V  K  S  I
GAGATTCTCTGGAAGAAATACGGGGTTCTGCTTCTGGTGGCAACATGCTATTGGGTGGTGGTCCCGCTTATGGGGTCAAATCAATAC
G  D  S  L  E  E  I  R  G  S  A  S  G  G  N  M  L  L  G  G  G  P  A  Y  G  V  K  S  I
```

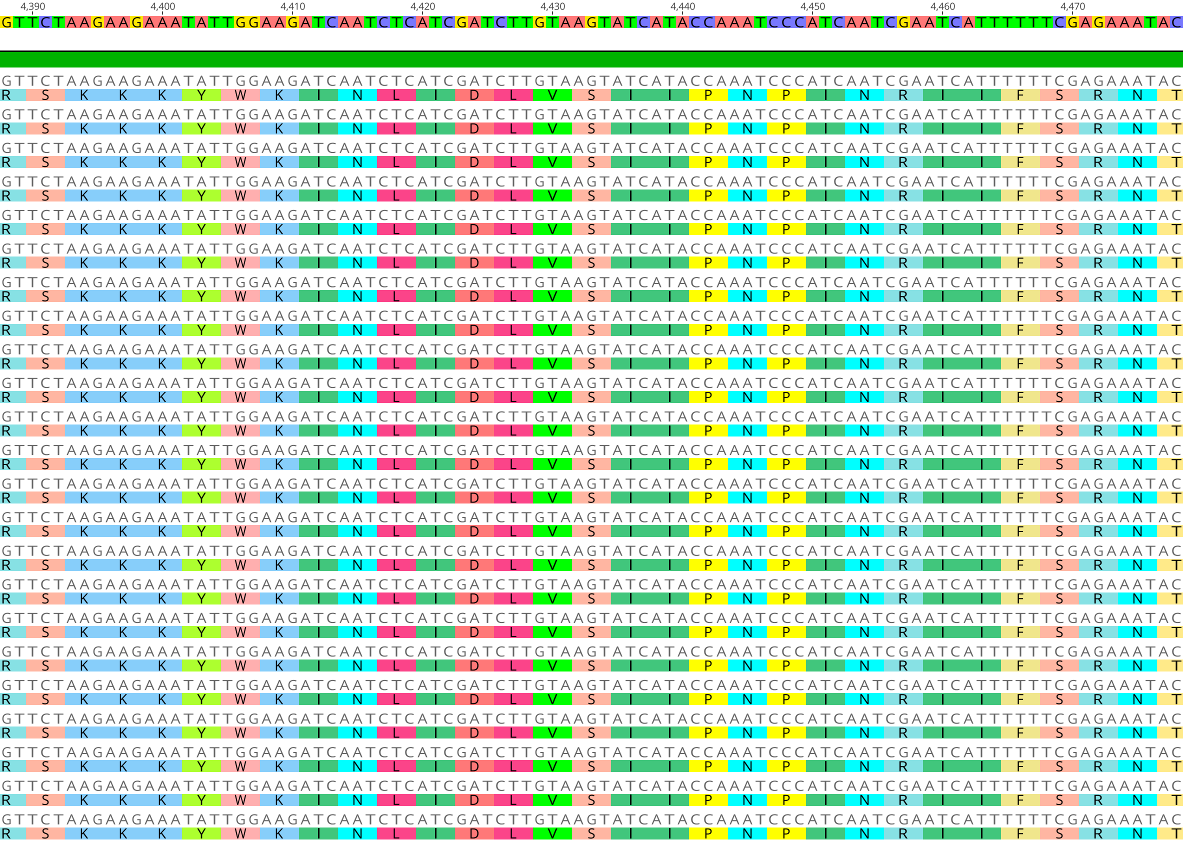
4,390 4,400 4,410 4,420 4,430 4,440 4,450 4,460 4,470
GTTCTAAGAAGAAATATTGGAAGATCAATCTCATCGATCTTGTAAGTATCATACCAAATCCCATCAATCGAATCATTTTTTCGAGAAATAC
GTTCTAAGAAGAAATATTGGAAGATCAATCTCATCGATCTTGTAAGTATCATACCAAATCCCATCAATCGAATCATTTTTTCGAGAAATAC
R S K K K Y W K I N L I D L V S I I P N P I N R I I F S R N T
GTTCTAAGAAGAAATATTGGAAGATCAATCTCATCGATCTTGTAAGTATCATACCAAATCCCATCAATCGAATCATTTTTTCGAGAAATAC
R S K K K Y W K I N L I D L V S I I P N P I N R I I F S R N T
GTTCTAAGAAGAAATATTGGAAGATCAATCTCATCGATCTTGTAAGTATCATACCAAATCCCATCAATCGAATCATTTTTTCGAGAAATAC
R S K K K Y W K I N L I D L V S I I P N P I N R I I F S R N T
GTTCTAAGAAGAAATATTGGAAGATCAATCTCATCGATCTTGTAAGTATCATACCAAATCCCATCAATCGAATCATTTTTTCGAGAAATAC
R S K K K Y W K I N L I D L V S I I P N P I N R I I F S R N T
GTTCTAAGAAGAAATATTGGAAGATCAATCTCATCGATCTTGTAAGTATCATACCAAATCCCATCAATCGAATCATTTTTTCGAGAAATAC
R S K K K Y W K I N L I D L V S I I P N P I N R I I F S R N T
GTTCTAAGAAGAAATATTGGAAGATCAATCTCATCGATCTTGTAAGTATCATACCAAATCCCATCAATCGAATCATTTTTTCGAGAAATAC
R S K K K Y W K I N L I D L V S I I P N P I N R I I F S R N T
GTTCTAAGAAGAAATATTGGAAGATCAATCTCATCGATCTTGTAAGTATCATACCAAATCCCATCAATCGAATCATTTTTTCGAGAAATAC
R S K K K Y W K I N L I D L V S I I P N P I N R I I F S R N T
GTTCTAAGAAGAAATATTGGAAGATCAATCTCATCGATCTTGTAAGTATCATACCAAATCCCATCAATCGAATCATTTTTTCGAGAAATAC
R S K K K Y W K I N L I D L V S I I P N P I N R I I F S R N T
GTTCTAAGAAGAAATATTGGAAGATCAATCTCATCGATCTTGTAAGTATCATACCAAATCCCATCAATCGAATCATTTTTTCGAGAAATAC
R S K K K Y W K I N L I D L V S I I P N P I N R I I F S R N T
GTTCTAAGAAGAAATATTGGAAGATCAATCTCATCGATCTTGTAAGTATCATACCAAATCCCATCAATCGAATCATTTTTTCGAGAAATAC
R S K K K Y W K I N L I D L V S I I P N P I N R I I F S R N T
GTTCTAAGAAGAAATATTGGAAGATCAATCTCATCGATCTTGTAAGTATCATACCAAATCCCATCAATCGAATCATTTTTTCGAGAAATAC
R S K K K Y W K I N L I D L V S I I P N P I N R I I F S R N T
GTTCTAAGAAGAAATATTGGAAGATCAATCTCATCGATCTTGTAAGTATCATACCAAATCCCATCAATCGAATCATTTTTTCGAGAAATAC
R S K K K Y W K I N L I D L V S I I P N P I N R I I F S R N T
GTTCTAAGAAGAAATATTGGAAGATCAATCTCATCGATCTTGTAAGTATCATACCAAATCCCATCAATCGAATCATTTTTTCGAGAAATAC
R S K K K Y W K I N L I D L V S I I P N P I N R I I F S R N T
GTTCTAAGAAGAAATATTGGAAGATCAATCTCATCGATCTTGTAAGTATCATACCAAATCCCATCAATCGAATCATTTTTTCGAGAAATAC
R S K K K Y W K I N L I D L V S I I P N P I N R I I F S R N T
GTTCTAAGAAGAAATATTGGAAGATCAATCTCATCGATCTTGTAAGTATCATACCAAATCCCATCAATCGAATCATTTTTTCGAGAAATAC
R S K K K Y W K I N L I D L V S I I P N P I N R I I F S R N T
GTTCTAAGAAGAAATATTGGAAGATCAATCTCATCGATCTTGTAAGTATCATACCAAATCCCATCAATCGAATCATTTTTTCGAGAAATAC
R S K K K Y W K I N L I D L V S I I P N P I N R I I F S R N T
GTTCTAAGAAGAAATATTGGAAGATCAATCTCATCGATCTTGTAAGTATCATACCAAATCCCATCAATCGAATCATTTTTTCGAGAAATAC
R S K K K Y W K I N L I D L V S I I P N P I N R I I F S R N T
GTTCTAAGAAGAAATATTGGAAGATCAATCTCATCGATCTTGTAAGTATCATACCAAATCCCATCAATCGAATCATTTTTTCGAGAAATAC
R S K K K Y W K I N L I D L V S I I P N P I N R I I F S R N T
GTTCTAAGAAGAAATATTGGAAGATCAATCTCATCGATCTTGTAAGTATCATACCAAATCCCATCAATCGAATCATTTTTTCGAGAAATAC
R S K K K Y W K I N L I D L V S I I P N P I N R I I F S R N T
GTTCTAAGAAGAAATATTGGAAGATCAATCTCATCGATCTTGTAAGTATCATACCAAATCCCATCAATCGAATCATTTTTTCGAGAAATAC
R S K K K Y W K I N L I D L V S I I P N P I N R I I F S R N T
GTTCTAAGAAGAAATATTGGAAGATCAATCTCATCGATCTTGTAAGTATCATACCAAATCCCATCAATCGAATCATTTTTTCGAGAAATAC
R S K K K Y W K I N L I D L V S I I P N P I N R I I F S R N T
GTTCTAAGAAGAAATATTGGAAGATCAATCTCATCGATCTTGTAAGTATCATACCAAATCCCATCAATCGAATCATTTTTTCGAGAAATAC
R S K K K Y W K I N L I D L V S I I P N P I N R I I F S R N T

```
4,480     4,490     4,500     4,510     4,520     4,530     4,540     4,550     4,560
GAGACATCTAAGTCGTACAAGTAAAGAGATCTATTCATTGATAAGAAAAAGAAAAAACGTGAACGGTGATTGGATTGATGAGAAAATAGAA

 1  GAGACATCTAAGTCGTACAAGTAAAGAGATCTATTCATTGATAAGAAAAAGAAAAAACGTGAACGGTGATTGGATTGATGAGAAAATAGAA
     R  H  L  S  R  T  S  K  E  I  Y  S  L  I  R  K  R  K  N  V  N  G  D  W  I  D  E  K  I  E
 2  GAGACATCTAAGTCGTACAAGTAAAGAGATCTATTCATTGATAAGAAAAAGAAAAAACGTGAACGGTGATTGGATTGATGAGAAAATAGAA
     R  H  L  S  R  T  S  K  E  I  Y  S  L  I  R  K  R  K  N  V  N  G  D  W  I  D  E  K  I  E
 3  GAGACATCTAAGTCGTACAAGTAAAGAGATCTATTCATTGATAAGAAAAAGAAAAAACGTGAACGGTGATTGGATTGATGAGAAAATAGAA
     R  H  L  S  R  T  S  K  E  I  Y  S  L  I  R  K  R  K  N  V  N  G  D  W  I  D  E  K  I  E
 4  GAGACATCTAAGTCGTACAAGTAAAGAGATCTATTCATTGATAAGAAAAAGAAAAAACGTGAACGGTGATTGGATTGATGAGAAAATAGAA
     R  H  L  S  R  T  S  K  E  I  Y  S  L  I  R  K  R  K  N  V  N  G  D  W  I  D  E  K  I  E
 5  GAGACATCTAAGTCGTACAAGTAAAGAGATCTATTCATTGATAAGAAAAAGAAAAAACGTGAACGGTGATTGGATTGATGAGAAAATAGAA
     R  H  L  S  R  T  S  K  E  I  Y  S  L  I  R  K  R  K  N  V  N  G  D  W  I  D  E  K  I  E
 6  GAGACATCTAAGTCGTACAAGTAAAGAGATCTATTCATTGATAAGAAAAAGAAAAAACGTGAACGGTGATTGGATTGATGATAAAATAGAA
     R  H  L  S  R  T  S  K  E  I  Y  S  L  I  R  K  R  K  N  V  N  G  D  W  I  D  D  K  I  E
 7  GAGACATCTAAGTCGTACAAGTAAAGAGATCTATTCATTGATAAGAAAAAGAAAAAACGTGAACGGTGATTGGATTGATGATAAAATAGAA
     R  H  L  S  R  T  S  K  E  I  Y  S  L  I  R  K  R  K  N  V  N  G  D  W  I  D  D  K  I  E
 8  GAGACATCTAAGTCGTACAAGTAAAGAGATCTATTCATTGATAAGAAAAAGAAAAAACGTGAACGGTGATTGGATTGATGATAAAATAGAA
     R  H  L  S  R  T  S  K  E  I  Y  S  L  I  R  K  R  K  N  V  N  G  D  W  I  D  D  K  I  E
 9  GAGACATCTAAGTCGTACAAGTAAAGAGATCTATTCATTGATAAGAAAAAGAAAAAACGTGAACGGTGATTGGATTGATGAGAAAATAGAA
     R  H  L  S  R  T  S  K  E  I  Y  S  L  I  R  K  R  K  N  V  N  G  D  W  I  D  E  K  I  E
10  GAGACATCTAAGTCGTACAAGTAAAGAGATCTATTCATTGATAAGAAAAAGAAAAAACGTGAACGGTGATTGGATTGATGAGAAAATAGAA
     R  H  L  S  R  T  S  K  E  I  Y  S  L  I  R  K  R  K  N  V  N  G  D  W  I  D  E  K  I  E
11  GAGACATCTAAGTCGTACAAGTAAAGAGATCTATTCATTGATAAGAAAAAGAAAAAACGTGAACGGTGATTGGATTGATGAGAAAATAGAA
     R  H  L  S  R  T  S  K  E  I  Y  S  L  I  R  K  R  K  N  V  N  G  D  W  I  D  E  K  I  E
12  GAGACATCTAAGTCGTACAAGTAAAGAGATCTATTCATTGATAAGAAAAAGAAAAAACGTGAACGGTGATTGGATTGATGAGAAAATAGAA
     R  H  L  S  R  T  S  K  E  I  Y  S  L  I  R  K  R  K  N  V  N  G  D  W  I  D  E  K  I  E
13  GAGACATCTAAGTCGTACAAGTAAAGAGATCTATTCATTGATAAGAAAAAGAAAAAACGTGAACGGTGATTGGATTGATGATAAAATAGAA
     R  H  L  S  R  T  S  K  E  I  Y  S  L  I  R  K  R  K  N  V  N  G  D  W  I  D  D  K  I  E
14  GAGACATCTAAGTCGTACAAGTAAAGAGATCTATTCATTGATAAGAAAAAGAAAAAACGTGAACGGTGATTGGATTGATGATAAAATAGAA
     R  H  L  S  R  T  S  K  E  I  Y  S  L  I  R  K  R  K  N  V  N  G  D  W  I  D  D  K  I  E
15  GAGACATCTAAGTCGTACAAGTAAAGAGATCTATTCATTGATAAGAAAAAGAAAAAACGTGAACGGTGATTGGATTGATGATAAAATAGAA
     R  H  L  S  R  T  S  K  E  I  Y  S  L  I  R  K  R  K  N  V  N  G  D  W  I  D  D  K  I  E
16  GAGACATCTAAGTCGTACAAGTAAAGAGATCTATTCATTGATAAGAAAAAGAAAAAACGTGAACGGTGATTGGATTGATGATAAAATAGAA
     R  H  L  S  R  T  S  K  E  I  Y  S  L  I  R  K  R  K  N  V  N  G  D  W  I  D  D  K  I  E
17  GAGACATCTAAGTCGTACAAGTAAAGAGATCTATTCATTGATAAGAAAAAGAAAAAACGTGAACGGTGATTGGATTGATGATAAAATAGAA
     R  H  L  S  R  T  S  K  E  I  Y  S  L  I  R  K  R  K  N  V  N  G  D  W  I  D  D  K  I  E
18  GAGACATCTAAGTCGTACAAGTAAAGAGATCTATTCATTGATAAGAAAAAGAAAAAACGTGAACGGTGATTGGATTGATGAGAAAATAGAA
     R  H  L  S  R  T  S  K  E  I  Y  S  L  I  R  K  R  K  N  V  N  G  D  W  I  D  E  K  I  E
19  GAGACATCTAAGTCGTACAAGTAAAGAGATCTATTCATTGATAAGAAAAAGAAAAAACGTGAACGGTGATTGGATTGATGAGAAAATAGAA
     R  H  L  S  R  T  S  K  E  I  Y  S  L  I  R  K  R  K  N  V  N  G  D  W  I  D  E  K  I  E
20  GAGACATCTAAGTCGTACAAGTAAAGAGATCTATTCATTGATAAGAAAAAGAAAAAACGTGAACGGTGATTGGATTGATGAGAAAATAGAA
     R  H  L  S  R  T  S  K  E  I  Y  S  L  I  R  K  R  K  N  V  N  G  D  W  I  D  E  K  I  E
21  GAGACATCTAAGTCGTACAAGTAAAGAGATCTATTCATTGATAAGAAAAAGAAAAAACGTGAACGGTGATTGGATTGATGATAAAATAGAA
     R  H  L  S  R  T  S  K  E  I  Y  S  L  I  R  K  R  K  N  V  N  G  D  W  I  D  D  K  I  E
22  GAGACATCTAAGTCGTACAAGTAAAGAGATCTATTCATTGATAAGAAAAAGAAAAAACGTGAACGGTGATTGGATTGATGATAAAATAGAA
     R  H  L  S  R  T  S  K  E  I  Y  S  L  I  R  K  R  K  N  V  N  G  D  W  I  D  D  K  I  E
23  GAGACATCTAAGTCGTACAAGTAAAGAGATCTATTCATTGATAAGAAAAATAAAAAACGTGAACGGTGATTGGATTGATGATAAAATAGAA
     R  H  L  S  R  T  S  K  E  I  Y  S  L  I  R  K  I  K  N  V  N  G  D  W  I  D  D  K  I  E
```

,570 4,580 4,590 4,600 4,610 4,620 4,630 4,640

TTCTGGGTCGCGAACAGTGATTCGATTGATGATGAAGAAAGAGAATTCTTGGTTCAGTTCTCCACCTTAACGAC

TTCTGGGTCGCGAACAGTGATTCGATTGATGATGAAGAAAGAGAATTCTTGGTTCAGTTCTCCACCTTAACGAC
F W V A N S D S I D D E E R E F L V Q F S T L T T

TTCTGGGTCGCGAACAGTGATTCGATTGATGATGAAGAAAGAGAATTCTTGGTTCAGTTCTCCACCTTAACGAC
F W V A N S D S I D D E E R E F L V Q F S T L T T

TTCTGGGTCGCGAACAGTGATTCGATTGATGATGAAGAAAGAGAATTCTTGGTTCAGTTCTCCACCTTAACGAC
F W V A N S D S I D D E E R E F L V Q F S T L T T

TTCTGGGTCGCGAACAGTGATTCGATTGATGATGAAGAAAGAGAATTCTTGGTTCAGTTCTCCACCTTAACGAC
F W V A N S D S I D D E E R E F L V Q F S T L T T

TTCTGGGTCGCGAACAGTGATTCGATTGATGATGAAGAAAGAGAATTCTTGGTTCAGTTCTCCACCTTAACGAC
F W V A N S D S I D D E E R E F L V Q F S T L T T

TTCTGGGTCGCGAACAGTGATTCGATTGATGATGAAGAAAGAGAATTCTTGGTTCAGTTCTCCACCTTAACGAC
F W V A N S D S I D D E E R E F L V Q F S T L T T

TTCTGGGTCGCGAACAGTGATTCGATTGATGATGAAGAAAGAGAATTCTTGGTTCAGTTCTCCACCTTAACGAC
F W V A N S D S I D D E E R E F L V Q F S T L T T

TTCTGGGTCGCGAACAGTGATTCGATTGATGATGAAGAAAGAGAATTCTTGGTTCAGTTCTCCACCTTAACGAC
F W V A N S D S I D D E E R E F L V Q F S T L T T

TTCTGGGTCGCGAACAGTGATTCGATTGATGATGAAGAAAGAGAATTCTTGGTTCAGTTCTCCACCTTAACGAC
F W V A N S D S I D D E E R E F L V Q F S T L T T

TTCTGGGTCGCGAACAGTGATTCGATTGATGATGAAGAAAGAGAATTCTTGGTTCAGTTCTCCACCTTAACGAC
F W V A N S D S I D D E E R E F L V Q F S T L T T

TTCTGGGTCGCGAACAGTGATTCGATTGATGATGAAGAAAGAGAATTCTTGGTTCAGTTCTCCACCTTAACGAC
F W V A N S D S I D D E E R E F L V Q F S T L T T

TTCTGGGTCGCGAACAGTGATTCGATTGATGATGAAGAAAGAGAATTCTTGGTTCAGTTCTCCACCTTAACGAC
F W V A N S D S I D D E E R E F L V Q F S T L T T

TTCTGGGTCGCGAACAGTGATTCGATTGATGATGAAGAAAGAGAATTCTTGGTTCAGTTCTCCACCTTAACGAC
F W V A N S D S I D D E E R E F L V Q F S T L T T

TTCTGGGTCGCGAACAGTGATTCGATTGATGATGAAGAAAGAGAATTCTTGGTTCAGTTCTCCACCTTAACGAC
F W V A N S D S I D D E E R E F L V Q F S T L T T

TTCTGGGTCGCGAACAGTGATTCGATTGATGATGAAGAAAGAGAATTCTTGGTTCAGTTCTCCACCTTAACGAC
F W V A N S D S I D D E E R E F L V Q F S T L T T

TTCTGGGTCGCGAACAGTGATTCGATTGATGATGAAGAAAGAGAATTCTTGGTTCAGTTCTCCACCTTAACGAC
F W V A N S D S I D D E E R E F L V Q F S T L T T

TTCTGGGTCGCGAACAGTGATTCGATTGATGATGAAGAAAGAGAATTCTTGGTTCAGTTCTCCACCTTAACGAC
F W V A N S D S I D D E E R E F L V Q F S T L T T

TTCTGGGTCGCGAACAGTGATTCGATTGATGATGAAGAAAGAGAATTCTTGGTTCAGTTCTCCACCTTAACGAC
F W V A N S D S I D D E E R E F L V Q F S T L T T

TTCTGGGTCGCGAACAGTGATTCGATTGATGATGAAGAAAGAGAATTCTTGGTTCAGTTCTCCACCTTAACGAC
F W V A N S D S I D D E E R E F L V Q F S T L T T

TTCTGGGTCGCGAACAGTGATTCGATTGATGATGAAGAAAGAGAATTCTTGGTTCAGTTCTCCACCTTAACGAC
F W V A N S D S I D D E E R E F L V Q F S T L T T

TTCTGGGTCGCGAACAGTGATTCGATTGATGATGAAGAAAGAGAATTCTTGGTTCAGTTCTCCACCTTAACGAC
F W V A N S D S I D D E E R E F L V Q F S T L T T

TTCTGGGTCGCGAACAGTGATTCGATTGATGATGAAGAAAGAGAATTCTTGGTTCAGTTCTCCACCTTAACGAC
F W V A N S D S I D D E E R E F L V Q F S T L T T

TTCTGGGTCGCGAACAGTGATTCGATTGATGATGAAGAAAGAGAATTCTTGGTTCAGTTCTCCACCTTAACGAC
F W V A N S D S I D D E E R E F L V Q F S T L T T

4,650 4,660 4,670 4,680 4,690 4,700 4,710 4,720 4,730

AGAAAAAAGGATTGATCAAATTCTATTGAGTCTGACTCATAGTGATCATTTATCAAAGAATGACTCTGGTTATCAAATGATTGAACAACC

AGAAAAAAGGATTGATCAAATTCTATTGAGTCTGACTCATAGTGATCATTTATCAAAGAATGACTCTGGTTATCAAATGATTGAACAACC
E K R I D Q I L L S L T H S D H L S K N D S G Y Q M I E Q P

AGAAAAAAGGATTGATCAAATTCTATTGAGTCTGACTCATAGTGATCATTTATCAAAGAATGACTCTGGTTATCAAATGATTGAACAACC
E K R I D Q I L L S L T H S D H L S K N D S G Y Q M I E Q P

AGAAAAAAGGATTGATCAAATTCTATTGAGTCTGACTCATAGTGATCATTTATCAAAGAATGACTCTGGTTATCAAATGATTGAACAACC
E K R I D Q I L L S L T H S D H L S K N D S G Y Q M I E Q P

AGAAAAAAGGATTGATCAAATTCTATTGAGTCTGACTCATAGTGATCATTTATCAAAGAATGACTCTGGTTATCAAATGATTGAACAACC
E K R I D Q I L L S L T H S D H L S K N D S G Y Q M I E Q P

AGAAAAAAGGATTGATCAAATTCTATTGAGTCTGACTCATAGTGATCATTTATCAAAGAATGACTCTGGTTATCAAATGATTGAACAACC
E K R I D Q I L L S L T H S D H L S K N D S G Y Q M I E Q P

AGAAAAAAGGATTGATCAAATTCTATTGAGTCTGACTCATAGTGATCATTTATCAAAGAATGACTCTGGTTATCAAATGATTGAACAACC
E K R I D Q I L L S L T H S D H L S K N D S G Y Q M I E Q P

AGAAAAAAGGATTGATCAAATTCTATTGAGTCTGACTCATAGTGATCATTTATCAAAGAATGACTCTGGTTATCAAATGATTGAACAACC
E K R I D Q I L L S L T H S D H L S K N D S G Y Q M I E Q P

AGAAAAAAGGATTGATCAAATTCTATTGAGTCTGACTCATAGTGATCATTTATCAAAGAATGACTCTGGTTATCAAATGATTGAACAACC
E K R I D Q I L L S L T H S D H L S K N D S G Y Q M I E Q P

AGAAAAAAGGATTGATCAAATTCTATTGAGTCTGACTCATAGTGATCATTTATCAAAGAATGACTCTGGTTATCAAATGATTGAACAACC
E K R I D Q I L L S L T H S D H L S K N D S G Y Q M I E Q P

AGAAAAAAGGATTGATCAAATTCTATTGAGTCTGACTCATAGTGATCATTTATCAAAGAATGACTCTGGTTATCAAATGATTGAACAACC
E K R I D Q I L L S L T H S D H L S K N D S G Y Q M I E Q P

AGAAAAAAGGATTGATCAAATTCTATTGAGTCTGACTCATAGTGATCATTTATCAAAGAATGACTCTGGTTATCAAATGATTGAACAACC
E K R I D Q I L L S L T H S D H L S K N D S G Y Q M I E Q P

AGAAAAAAGGATTGATCAAATTCTATTGAGTCTGACTCATAGTGATCATTTATCAAAGAATGACTCTGGTTATCAAATGATTGAACAACC
E K R I D Q I L L S L T H S D H L S K N D S G Y Q M I E Q P

AGAAAAAAGGATTGATCAAATTCTATTGAGTCTGACTCATAGTGATCATTTATCAAAGAATGACTCTGGTTATCAAATGATTGAACAACC
E K R I D Q I L L S L T H S D H L S K N D S G Y Q M I E Q P

AGAAAAAAGGATTGATCAAATTCTATTGAGTCTGACTCATAGTGATCATTTATCAAAGAATGACTCTGGTTATCAAATGATTGAACAACC
E K R I D Q I L L S L T H S D H L S K N D S G Y Q M I E Q P

AGAAAAAAGGATTGATCAAATTCTATTGAGTCTGACTCATAGTGATCATTTATCAAAGAATGACTCTGGTTATCAAATGATTGAACAACC
E K R I D Q I L L S L T H S D H L S K N D S G Y Q M I E Q P

AGAAAAAAGGATTGATCAAATTCTATTGAGTCTGACTCATAGTGATCATTTATCAAAGAATGACTCTGGTTATCAAATGATTGAACAACC
E K R I D Q I L L S L T H S D H L S K N D S G Y Q M I E Q P

AGAAAAAAGGATTGATCAAATTCTATTGAGTCTGACTCATAGTGATCATTTATCAAAGAATGACTCTGGTTATCAAATGATTGAACAACC
E K R I D Q I L L S L T H S D H L S K N D S G Y Q M I E Q P

AGAAAAAAGGATTGATCAAATTCTATTGAGTCTGACTCATAGTGATCATTTATCAAAGAATGACTCTGGTTATCAAATGATTGAACAACC
E K R I D Q I L L S L T H S D H L S K N D S G Y Q M I E Q P

AGAAAAAAGGATTGATCAAATTCTATTGAGTCTGACTCATAGTGATCATTTATCAAAGAATGACTCTGGTTATCAAATGATTGAACAACC
E K R I D Q I L L S L T H S D H L S K N D S G Y Q M I E Q P

AGAAAAAAGGATTGATCAAATTCTATTGAGTCTGACTCATAGTGATCATTTATCAAAGAATGACTCTGGTTATCAAATGATTGAACAACC
E K R I D Q I L L S L T H S D H L S K N D S G Y Q M I E Q P

AGAAAAAAGGATTGATCAAATTCTATTGAGTCTGACTCATAGTGATCATTTATCAAAGAATGACTCTGGTTATCAAATGATTGAACAACC
E K R I D Q I L L S L T H S D H L S K N D S G Y Q M I E Q P

AGAAAAAAGGATTGATCAAATTCTATTGAGTCTGACTCATAGTGATCATTTATCAAAGAATGACTCTGGTTATCAAATGATTGAACAACC
E K R I D Q I L L S L T H S D H L S K N D S G Y Q M I E Q P

AGAAAAAAGGATTGATCAAATTCTATTGAGTCTGACTCATAGTGATCATTTATCAAAGAATGACTCTGGTTATCAAATGATTGAACAACC
E K R I D Q I L L S L T H S D H L S K N D S G Y Q M I E Q P

```
4,740 4,750 4,760 4,770 4,780 4,790 4,800 4,810 4,820
GGGATCAATTTCCTTACGATACTTAGTTGACATTCATCAAAAGGATCTAATGAATTATGAGTTCAATAGATCCTGTTTAGCAGAAAGACG

GGGATCAATTTCCTTACGATACTTAGTTGACATTCATCAAAAGGATCTAATGAATTATGAGTTCAATAGATCCTGTTTAGCAGAAAGACG
G S I S L R Y L V D I H Q K D L M N Y E F N R S C L A E R R
GGGATCAATTTCCTTACGATACTTAGTTGACATTCATCAAAAGGATCTAATGAATTATGAGTTCAATAGATCCTGTTTAGCAGAAAGACG
G S I S L R Y L V D I H Q K D L M N Y E F N R S C L A E R R
GGGATCAATTTCCTTACGATACTTAGTTGACATTCATCAAAAGGATCTAATGAATTATGAGTTCAATAGATCCTGTTTAGCAGAAAGACG
G S I S L R Y L V D I H Q K D L M N Y E F N R S C L A E R R
GGGATCAATTTCCTTACGATACTTAGTTGACATTCATCAAAAGGATCTAATGAATTATGAGTTCAATAGATCCTGTTTAGCAGAAAGACG
G S I S L R Y L V D I H Q K D L M N Y E F N R S C L A E R R
GGGATCAATTTCCTTACGATACTTAGTTGACATTCATCAAAAGGATCTAATGAATTATGAGTTCAATAGATCCTGTTTAGCAGAAAGACG
G S I S L R Y L V D I H Q K D L M N Y E F N R S C L A E R R
GGGATCAATTTCCTTACGATACTTAGTTGACATTCATCAAAAGGATCTAATGAATTATGAGTTCAATAGATCCTGTTTAGCAGAAAGACG
G S I S L R Y L V D I H Q K D L M N Y E F N R S C L A E R R
GGGATCAATTTCCTTACGATACTTAGTTGACATTCATCAAAAGGATCTAATGAATTATGAGTTCAATAGATCCTGTTTAGCAGAAAGACG
G S I S L R Y L V D I H Q K D L M N Y E F N R S C L A E R R
GGGATCAATTTCCTTACGATACTTAGTTGACATTCATCAAAAGGATCTAATGAATTATGAGTTCAATAGATCCTGTTTAGCAGAAAGACG
G S I S L R Y L V D I H Q K D L M N Y E F N R S C L A E R R
GGGATCAATTTCCTTACGATACTTAGTTGACATTCATCAAAAGGATCTAATGAATTATGAGTTCAATAGATCCTGTTTAGCAGAAAGACG
G S I S L R Y L V D I H Q K D L M N Y E F N R S C L A E R R
GGGATCAATTTCCTTACGATACTTAGTTGACATTCATCAAAAGGATCTAATGAATTATGAGTTCAATAGATCCTGTTTAGCAGAAAGACG
G S I S L R Y L V D I H Q K D L M N Y E F N R S C L A E R R
GGGATCAATTTCCTTACGATACTTAGTTGACATTCATCAAAAGGATCTAATGAATTATGAGTTCAATAGATCCTGTTTAGCAGAAAGACG
G S I S L R Y L V D I H Q K D L M N Y E F N R S C L A E R R
GGGATCAATTTCCTTACGATACTTAGTTGACATTCATCAAAAGGATCTAATGAATTATGAGTTCAATAGATCCTGTTTAGCAGAAAGACG
G S I S L R Y L V D I H Q K D L M N Y E F N R S C L A E R R
GGGATCAATTTCCTTACGATACTTAGTTGACATTCATCAAAAGGATCTAATGAATTATGAGTTCAATAGATCCTGTTTAGCAGAAAGACG
G S I S L R Y L V D I H Q K D L M N Y E F N R S C L A E R R
GGGATCAATTTCCTTACGATACTTAGTTGACATTCATCAAAAGGATCTAATGAATTATGAGTTCAATAGATCCTGTTTAGCAGAAAGACG
G S I S L R Y L V D I H Q K D L M N Y E F N R S C L A E R R
GGGATCAATTTCCTTACGATACTTAGTTGACATTCATCAAAAGGATCTAATGAATTATGAGTTCAATAGATCCTGTTTAGCAGAAAGACG
G S I S L R Y L V D I H Q K D L M N Y E F N R S C L A E R R
GGGATCAATTTCCTTACGATACTTAGTTGACATTCATCAAAAGGATCTAATGAATTATGAGTTCAATAGATCCTGTTTAGCAGAAAGACG
G S I S L R Y L V D I H Q K D L M N Y E F N R S C L A E R R
GGGATCAATTTCCTTACGATACTTAGTTGACATTCATCAAAAGGATCTAATGAATTATGAGTTCAATAGATCCTGTTTAGCAGAAAGACG
G S I S L R Y L V D I H Q K D L M N Y E F N R S C L A E R R
GGGATCAATTTCCTTACGATACTTAGTTGACATTCATCAAAAGGATCTAATGAATTATGAGTTCAATAGATCCTGTTTAGCAGAAAGACG
G S I S L R Y L V D I H Q K D L M N Y E F N R S C L A E R R
GGGATCAATTTCCTTACGATACTTAGTTGACATTCATCAAAAGGATCTAATGAATTATGAGTTCAATAGATCCTGTTTAGCAGAAAGACG
G S I S L R Y L V D I H Q K D L M N Y E F N R S C L A E R R
GGGATCAATTTCCTTACGATACTTAGTTGACATTCATCAAAAGGATCTAATGAATTATGAGTTCAATAGATCCTGTTTAGCAGAAAGACG
G S I S L R Y L V D I H Q K D L M N Y E F N R S C L A E R R
GGGATCAATTTCCTTACGATACTTAGTTGACATTCATCAAAAGGATCTAATGAATTATGAGTTCAATAGATCCTGTTTAGCAGAAAGACG
G S I S L R Y L V D I H Q K D L M N Y E F N R S C L A E R R
GGGATCAATTTCCTTACGATACTTAGTTGACATTCATCAAAAGGATCTAATGAATTATGAGTTCAATAGATCCTGTTTAGCAGAAAGACG
G S I S L R Y L V D I H Q K D L M N Y E F N R S C L A E R R
GGGATCAATTTACTTACGATACTTAGTTGACATTCATCAAAAGGATCTAATGAATTATGAGTTCAATAGATCCTGTTTAGCAGAAAGACG
G S I Y L R Y L V D I H Q K D L M N Y E F N R S C L A E R R
```

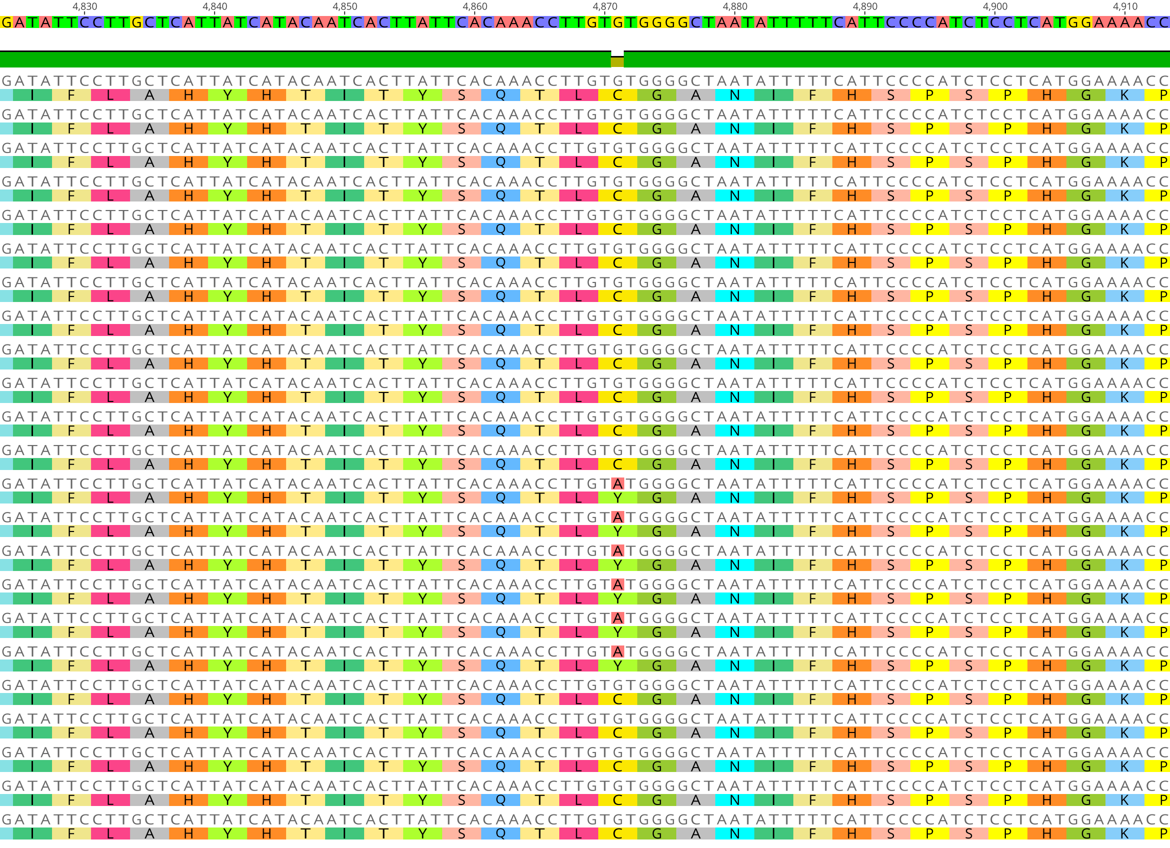
4,830 4,840 4,850 4,860 4,870 4,880 4,890 4,900 4,910
GATATTCCTTGCTCATTATCATACAATCACTTATTCACAAACCTTGTGTGGGGCTAATATTTTTCATTCCCCATCTCCTCATGGAAAACC
GATATTCCTTGCTCATTATCATACAATCACTTATTCACAAACCTTGTGTGGGGCTAATATTTTTCATTCCCCATCTCCTCATGGAAAACC
I F L A H Y H T I T Y S Q T L C G A N I F H S P S P H G K P
GATATTCCTTGCTCATTATCATACAATCACTTATTCACAAACCTTGTGTGGGGCTAATATTTTTCATTCCCCATCTCCTCATGGAAAACC
I F L A H Y H T I T Y S Q T L C G A N I F H S P S P H G K P
GATATTCCTTGCTCATTATCATACAATCACTTATTCACAAACCTTGTGTGGGGCTAATATTTTTCATTCCCCATCTCCTCATGGAAAACC
I F L A H Y H T I T Y S Q T L C G A N I F H S P S P H G K P
GATATTCCTTGCTCATTATCATACAATCACTTATTCACAAACCTTGTGTGGGGCTAATATTTTTCATTCCCCATCTCCTCATGGAAAACC
I F L A H Y H T I T Y S Q T L C G A N I F H S P S P H G K P
GATATTCCTTGCTCATTATCATACAATCACTTATTCACAAACCTTGTGTGGGGCTAATATTTTTCATTCCCCATCTCCTCATGGAAAACC
I F L A H Y H T I T Y S Q T L C G A N I F H S P S P H G K P
GATATTCCTTGCTCATTATCATACAATCACTTATTCACAAACCTTGTGTGGGGCTAATATTTTTCATTCCCCATCTCCTCATGGAAAACC
I F L A H Y H T I T Y S Q T L C G A N I F H S P S P H G K P
GATATTCCTTGCTCATTATCATACAATCACTTATTCACAAACCTTGTGTGGGGCTAATATTTTTCATTCCCCATCTCCTCATGGAAAACC
I F L A H Y H T I T Y S Q T L C G A N I F H S P S P H G K P
GATATTCCTTGCTCATTATCATACAATCACTTATTCACAAACCTTGTGTGGGGCTAATATTTTTCATTCCCCATCTCCTCATGGAAAACC
I F L A H Y H T I T Y S Q T L C G A N I F H S P S P H G K P
GATATTCCTTGCTCATTATCATACAATCACTTATTCACAAACCTTGTGTGGGGCTAATATTTTTCATTCCCCATCTCCTCATGGAAAACC
I F L A H Y H T I T Y S Q T L C G A N I F H S P S P H G K P
GATATTCCTTGCTCATTATCATACAATCACTTATTCACAAACCTTGTGTGGGGCTAATATTTTTCATTCCCCATCTCCTCATGGAAAACC
I F L A H Y H T I T Y S Q T L C G A N I F H S P S P H G K P
GATATTCCTTGCTCATTATCATACAATCACTTATTCACAAACCTTGTGTGGGGCTAATATTTTTCATTCCCCATCTCCTCATGGAAAACC
I F L A H Y H T I T Y S Q T L C G A N I F H S P S P H G K P
GATATTCCTTGCTCATTATCATACAATCACTTATTCACAAACCTTGTGTGGGGCTAATATTTTTCATTCCCCATCTCCTCATGGAAAACC
I F L A H Y H T I T Y S Q T L C G A N I F H S P S P H G K P
GATATTCCTTGCTCATTATCATACAATCACTTATTCACAAACCTTGTATGGGGCTAATATTTTTCATTCCCCATCTCCTCATGGAAAACC
I F L A H Y H T I T Y S Q T L Y G A N I F H S P S P H G K P
GATATTCCTTGCTCATTATCATACAATCACTTATTCACAAACCTTGTATGGGGCTAATATTTTTCATTCCCCATCTCCTCATGGAAAACC
I F L A H Y H T I T Y S Q T L Y G A N I F H S P S P H G K P
GATATTCCTTGCTCATTATCATACAATCACTTATTCACAAACCTTGTATGGGGCTAATATTTTTCATTCCCCATCTCCTCATGGAAAACC
I F L A H Y H T I T Y S Q T L Y G A N I F H S P S P H G K P
GATATTCCTTGCTCATTATCATACAATCACTTATTCACAAACCTTGTATGGGGCTAATATTTTTCATTCCCCATCTCCTCATGGAAAACC
I F L A H Y H T I T Y S Q T L Y G A N I F H S P S P H G K P
GATATTCCTTGCTCATTATCATACAATCACTTATTCACAAACCTTGTATGGGGCTAATATTTTTCATTCCCCATCTCCTCATGGAAAACC
I F L A H Y H T I T Y S Q T L Y G A N I F H S P S P H G K P
GATATTCCTTGCTCATTATCATACAATCACTTATTCACAAACCTTGTATGGGGCTAATATTTTTCATTCCCCATCTCCTCATGGAAAACC
I F L A H Y H T I T Y S Q T L Y G A N I F H S P S P H G K P
GATATTCCTTGCTCATTATCATACAATCACTTATTCACAAACCTTGTGTGGGGCTAATATTTTTCATTCCCCATCTCCTCATGGAAAACC
I F L A H Y H T I T Y S Q T L C G A N I F H S P S P H G K P
GATATTCCTTGCTCATTATCATACAATCACTTATTCACAAACCTTGTGTGGGGCTAATATTTTTCATTCCCCATCTCCTCATGGAAAACC
I F L A H Y H T I T Y S Q T L C G A N I F H S P S P H G K P
GATATTCCTTGCTCATTATCATACAATCACTTATTCACAAACCTTGTGTGGGGCTAATATTTTTCATTCCCCATCTCCTCATGGAAAACC
I F L A H Y H T I T Y S Q T L C G A N I F H S P S P H G K P
GATATTCCTTGCTCATTATCATACAATCACTTATTCACAAACCTTGTGTGGGGCTAATATTTTTCATTCCCCATCTCCTCATGGAAAACC
I F L A H Y H T I T Y S Q T L C G A N I F H S P S P H G K P
GATATTCCTTGCTCATTATCATACAATCACTTATTCACAAACCTTGTGTGGGGCTAATATTTTTCATTCCCCATCTCCTCATGGAAAACC
I F L A H Y H T I T Y S Q T L C G A N I F H S P S P H G K P

4,920 4,930 4,940 4,950 4,960 4,970 4,980 4,990

CTTTTCGCTCCGCTTAGCCCTATCCCCTTCTAGAGGTATTTTAGTGATAGGTTCTATAGGAACTGGACGATCCTGTTTG

```
CTTTTCGCTCCGCTTAGCCCTATCCCCTTCTAGAGGTATTTTAGTGATAGGTTCTATAGGAACTGGACGATCCTGTTTG
 F  S  L  R  L  A  L  S  P  S  R  G  I  L  V  I  G  S  I  G  T  G  R  S  C  L
CTTTTCGCTCCGCTTAGCCCTATCCCCTTCTAGAGGTATTTTAGTGATAGGTTCTATAGGAACTGGACGATCCTGTTTG
 F  S  L  R  L  A  L  S  P  S  R  G  I  L  V  I  G  S  I  G  T  G  R  S  C  L
CTTTTCGCTCCGCTTAGCCCTATCCCCTTCTAGAGGTATTTTAGTGATAGGTTCTATAGGAACTGGACGATCCTGTTTG
 F  S  L  R  L  A  L  S  P  S  R  G  I  L  V  I  G  S  I  G  T  G  R  S  C  L
CTTTTCGCTCCGCTTAGCCCTATCCCCTTCTAGAGGTATTTTAGTGATAGGTTCTATAGGAACTGGACGATCCTGTTTG
 F  S  L  R  L  A  L  S  P  S  R  G  I  L  V  I  G  S  I  G  T  G  R  S  C  L
CTTTTCGCTCCGCTTAGCCCTATCCCCTTCTAGAGGTATTTTAGTGATAGGTTCTATAGGAACTGGACGATCCTGTTTG
 F  S  L  R  L  A  L  S  P  S  R  G  I  L  V  I  G  S  I  G  T  G  R  S  C  L
CTTTTCGCTCCGCTTAGCCCTATCCCCTTCTAGAGGTATTTTAGTGATAGGTTCTATAGGAACTGGACGATCCTGTTTG
 F  S  L  R  L  A  L  S  P  S  R  G  I  L  V  I  G  S  I  G  T  G  R  S  C  L
CTTTTCGCTCCGCTTAGCCCTATCCCCTTCTAGAGGTATTTTAGTGATAGGTTCTATAGGAACTGGACGATCCTGTTTG
 F  S  L  R  L  A  L  S  P  S  R  G  I  L  V  I  G  S  I  G  T  G  R  S  C  L
CTTTTCGCTCCGCTTAGCCCTATCCCCTTCTAGAGGTATTTTAGTGATAGGTTCTATAGGAACTGGACGATCCTGTTTG
 F  S  L  R  L  A  L  S  P  S  R  G  I  L  V  I  G  S  I  G  T  G  R  S  C  L
CTTTTCGCTCCGCTTAGCCCTATCCCCTTCTAGAGGTATTTTAGTGATAGGTTCTATAGGAACTGGACGATCCTGTTTG
 F  S  L  R  L  A  L  S  P  S  R  G  I  L  V  I  G  S  I  G  T  G  R  S  C  L
CTTTTCGCTCCGCTTAGCCCTATCCCCTTCTAGAGGTATTTTAGTGATAGGTTCTATAGGAACTGGACGATCCTGTTTG
 F  S  L  R  L  A  L  S  P  S  R  G  I  L  V  I  G  S  I  G  T  G  R  S  C  L
CTTTTCGCTCCGCTTAGCCCTATCCCCTTCTAGAGGTATTTTAGTGATAGGTTCTATAGGAACTGGACGATCCTGTTTG
 F  S  L  R  L  A  L  S  P  S  R  G  I  L  V  I  G  S  I  G  T  G  R  S  C  L
CTTTTCGCTCCGCTTAGCCCTATCCCCTTCTAGAGGTATTTTAGTGATAGGTTCTATAGGAACTGGACGATCCTGTTTG
 F  S  L  R  L  A  L  S  P  S  R  G  I  L  V  I  G  S  I  G  T  G  R  S  C  L
CTTTTCGCTCCGCTTAGCCCTATCCCCTTCTAGAGGTATTTTAGTGATAGGTTCTATAGGAACTGGACGATCCTGTTTG
 F  S  L  R  L  A  L  S  P  S  R  G  I  L  V  I  G  S  I  G  T  G  R  S  C  L
CTTTTCGCTCCGCTTAGCCCTATCCCCTTCTAGAGGTATTTTAGTGATAGGTTCTATAGGAACTGGACGATCCTGTTTG
 F  S  L  R  L  A  L  S  P  S  R  G  I  L  V  I  G  S  I  G  T  G  R  S  C  L
CTTTTCGCTCCGCTTAGCCCTATCCCCTTCTAGAGGTATTTTAGTGATAGGTTCTATAGGAACTGGACGATCCTGTTTG
 F  S  L  R  L  A  L  S  P  S  R  G  I  L  V  I  G  S  I  G  T  G  R  S  C  L
CTTTTCGCTCCGCTTAGCCCTATCCCCTTCTAGAGGTATTTTAGTGATAGGTTCTATAGGAACTGGACGATCCTGTTTG
 F  S  L  R  L  A  L  S  P  S  R  G  I  L  V  I  G  S  I  G  T  G  R  S  C  L
CTTTTCGCTCCGCTTAGCCCTATCCCCTTCTAGAGGTATTTTAGTGATAGGTTCTATAGGAACTGGACGATCCTGTTTG
 F  S  L  R  L  A  L  S  P  S  R  G  I  L  V  I  G  S  I  G  T  G  R  S  C  L
CTTTTCGCTCCGCTTAGCCCTATCCCCTTCTAGAGGTATTTTAGTGATAGGTTCTATAGGAACTGGACGATCCTGTTTG
 F  S  L  R  L  A  L  S  P  S  R  G  I  L  V  I  G  S  I  G  T  G  R  S  C  L
CTTTTCGCTCCGCTTAGCCCTATCCCCTTCTAGAGGTATTTTAGTGATAGGTTCTATAGAAACTGGACGATCCTGTTTG
 F  S  L  R  L  A  L  S  P  S  R  G  I  L  V  I  G  S  I  E  T  G  R  S  C  L
CTTTTCGCTCCGCTTAGCCCTATCCCCTTCTAGAGGTATTTTAGTGATAGGTTCTATAGAAACTGGACGATCCTGTTTG
 F  S  L  R  L  A  L  S  P  S  R  G  I  L  V  I  G  S  I  E  T  G  R  S  C  L
CTTTTCGCTCCGCTTAGCCCTATCCCCTTCTAGAGGTATTTTAGTGATAGGTTCTATAGAAACTGGACGATCCTGTTTG
 F  S  L  R  L  A  L  S  P  S  R  G  I  L  V  I  G  S  I  E  T  G  R  S  C  L
CTTTTCGCTCCGCTTAGCCCTATCCCCTTCTAGAGGTATTTTAGTGATAGGTTCTATAGAAACTGGACGATCCTGTTTG
 F  S  L  R  L  A  L  S  P  S  R  G  I  L  V  I  G  S  I  E  T  G  R  S  C  L
CTTTTCGCTCCGCTTAGCCCTATCCCCTTCTAGAGGTATTTTAGTGATAGGTTCTATAGGAACTGGACGATCCTGTTTG
 F  S  L  R  L  A  L  S  P  S  R  G  I  L  V  I  G  S  I  G  T  G  R  S  C  L
```

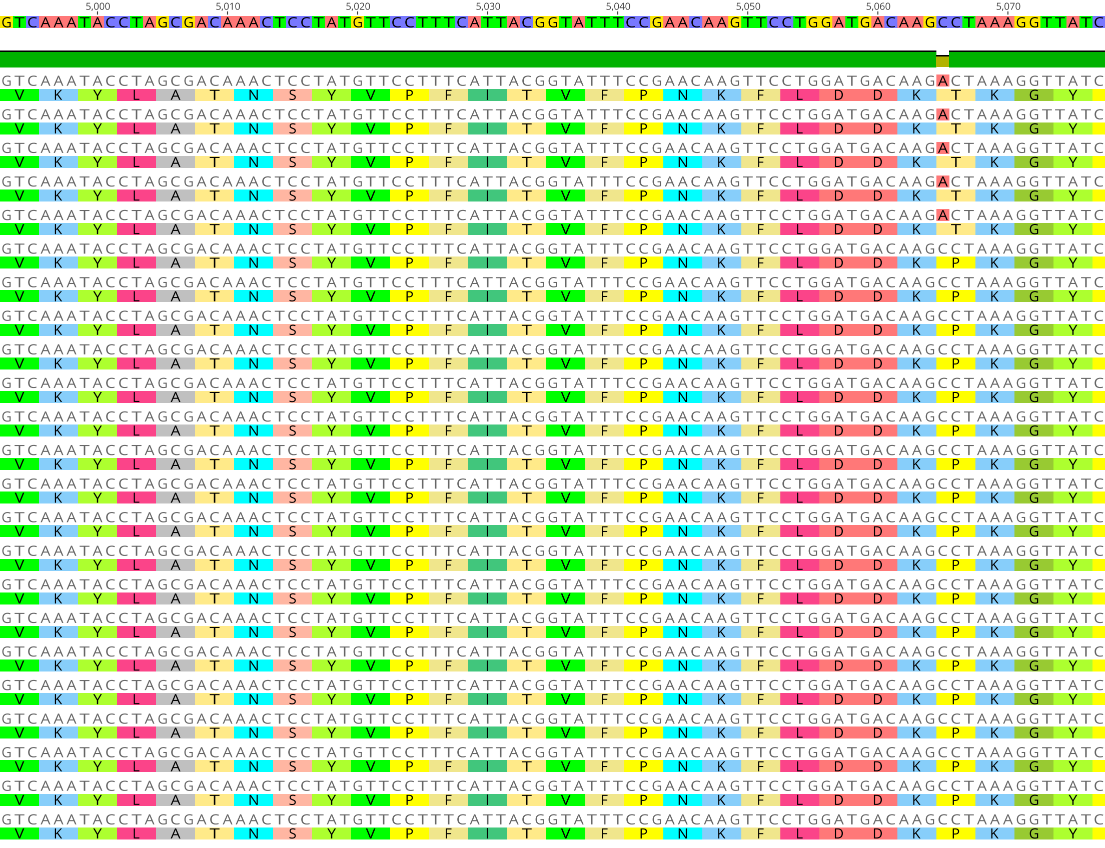

5,000 5,010 5,020 5,030 5,040 5,050 5,060 5,070
GTCAAATACCTAGCGACAAACTCCTATGTTCCTTTCATTACGGTATTTCCGAACAAGTTCCTGGATGACAAGCCTAAAGGTTATC
GTCAAATACCTAGCGACAAACTCCTATGTTCCTTTCATTACGGTATTTCCGAACAAGTTCCTGGATGACAAGACTAAAGGTTATC
V K Y L A T N S Y V P F I T V F P N K F L D D K T K G Y
GTCAAATACCTAGCGACAAACTCCTATGTTCCTTTCATTACGGTATTTCCGAACAAGTTCCTGGATGACAAGACTAAAGGTTATC
V K Y L A T N S Y V P F I T V F P N K F L D D K T K G Y
GTCAAATACCTAGCGACAAACTCCTATGTTCCTTTCATTACGGTATTTCCGAACAAGTTCCTGGATGACAAGACTAAAGGTTATC
V K Y L A T N S Y V P F I T V F P N K F L D D K T K G Y
GTCAAATACCTAGCGACAAACTCCTATGTTCCTTTCATTACGGTATTTCCGAACAAGTTCCTGGATGACAAGACTAAAGGTTATC
V K Y L A T N S Y V P F I T V F P N K F L D D K T K G Y
GTCAAATACCTAGCGACAAACTCCTATGTTCCTTTCATTACGGTATTTCCGAACAAGTTCCTGGATGACAAGACTAAAGGTTATC
V K Y L A T N S Y V P F I T V F P N K F L D D K T K G Y
GTCAAATACCTAGCGACAAACTCCTATGTTCCTTTCATTACGGTATTTCCGAACAAGTTCCTGGATGACAAGCCTAAAGGTTATC
V K Y L A T N S Y V P F I T V F P N K F L D D K P K G Y
GTCAAATACCTAGCGACAAACTCCTATGTTCCTTTCATTACGGTATTTCCGAACAAGTTCCTGGATGACAAGCCTAAAGGTTATC
V K Y L A T N S Y V P F I T V F P N K F L D D K P K G Y
GTCAAATACCTAGCGACAAACTCCTATGTTCCTTTCATTACGGTATTTCCGAACAAGTTCCTGGATGACAAGCCTAAAGGTTATC
V K Y L A T N S Y V P F I T V F P N K F L D D K P K G Y
GTCAAATACCTAGCGACAAACTCCTATGTTCCTTTCATTACGGTATTTCCGAACAAGTTCCTGGATGACAAGCCTAAAGGTTATC
V K Y L A T N S Y V P F I T V F P N K F L D D K P K G Y
GTCAAATACCTAGCGACAAACTCCTATGTTCCTTTCATTACGGTATTTCCGAACAAGTTCCTGGATGACAAGCCTAAAGGTTATC
V K Y L A T N S Y V P F I T V F P N K F L D D K P K G Y
GTCAAATACCTAGCGACAAACTCCTATGTTCCTTTCATTACGGTATTTCCGAACAAGTTCCTGGATGACAAGCCTAAAGGTTATC
V K Y L A T N S Y V P F I T V F P N K F L D D K P K G Y
GTCAAATACCTAGCGACAAACTCCTATGTTCCTTTCATTACGGTATTTCCGAACAAGTTCCTGGATGACAAGCCTAAAGGTTATC
V K Y L A T N S Y V P F I T V F P N K F L D D K P K G Y
GTCAAATACCTAGCGACAAACTCCTATGTTCCTTTCATTACGGTATTTCCGAACAAGTTCCTGGATGACAAGCCTAAAGGTTATC
V K Y L A T N S Y V P F I T V F P N K F L D D K P K G Y
GTCAAATACCTAGCGACAAACTCCTATGTTCCTTTCATTACGGTATTTCCGAACAAGTTCCTGGATGACAAGCCTAAAGGTTATC
V K Y L A T N S Y V P F I T V F P N K F L D D K P K G Y
GTCAAATACCTAGCGACAAACTCCTATGTTCCTTTCATTACGGTATTTCCGAACAAGTTCCTGGATGACAAGCCTAAAGGTTATC
V K Y L A T N S Y V P F I T V F P N K F L D D K P K G Y
GTCAAATACCTAGCGACAAACTCCTATGTTCCTTTCATTACGGTATTTCCGAACAAGTTCCTGGATGACAAGCCTAAAGGTTATC
V K Y L A T N S Y V P F I T V F P N K F L D D K P K G Y
GTCAAATACCTAGCGACAAACTCCTATGTTCCTTTCATTACGGTATTTCCGAACAAGTTCCTGGATGACAAGCCTAAAGGTTATC
V K Y L A T N S Y V P F I T V F P N K F L D D K P K G Y
GTCAAATACCTAGCGACAAACTCCTATGTTCCTTTCATTACGGTATTTCCGAACAAGTTCCTGGATGACAAGCCTAAAGGTTATC
V K Y L A T N S Y V P F I T V F P N K F L D D K P K G Y
GTCAAATACCTAGCGACAAACTCCTATGTTCCTTTCATTACGGTATTTCCGAACAAGTTCCTGGATGACAAGCCTAAAGGTTATC
V K Y L A T N S Y V P F I T V F P N K F L D D K P K G Y
GTCAAATACCTAGCGACAAACTCCTATGTTCCTTTCATTACGGTATTTCCGAACAAGTTCCTGGATGACAAGCCTAAAGGTTATC
V K Y L A T N S Y V P F I T V F P N K F L D D K P K G Y
GTCAAATACCTAGCGACAAACTCCTATGTTCCTTTCATTACGGTATTTCCGAACAAGTTCCTGGATGACAAGCCTAAAGGTTATC
V K Y L A T N S Y V P F I T V F P N K F L D D K P K G Y
GTCAAATACCTAGCGACAAACTCCTATGTTCCTTTCATTACGGTATTTCCGAACAAGTTCCTGGATGACAAGCCTAAAGGTTATC
V K Y L A T N S Y V P F I T V F P N K F L D D K P K G Y
GTCAAATACCTAGCGACAAACTCCTATGTTCCTTTCATTACGGTATTTCCGAACAAGTTCCTGGATGACAAGCCTAAAGGTTATC
V K Y L A T N S Y V P F I T V F P N K F L D D K P K G Y
GTCAAATACCTAGCGACAAACTCCTATGTTCCTTTCATTACGGTATTTCCGAACAAGTTCCTGGATGACAAGCCTAAAGGTTATC
V K Y L A T N S Y V P F I T V F P N K F L D D K P K G Y

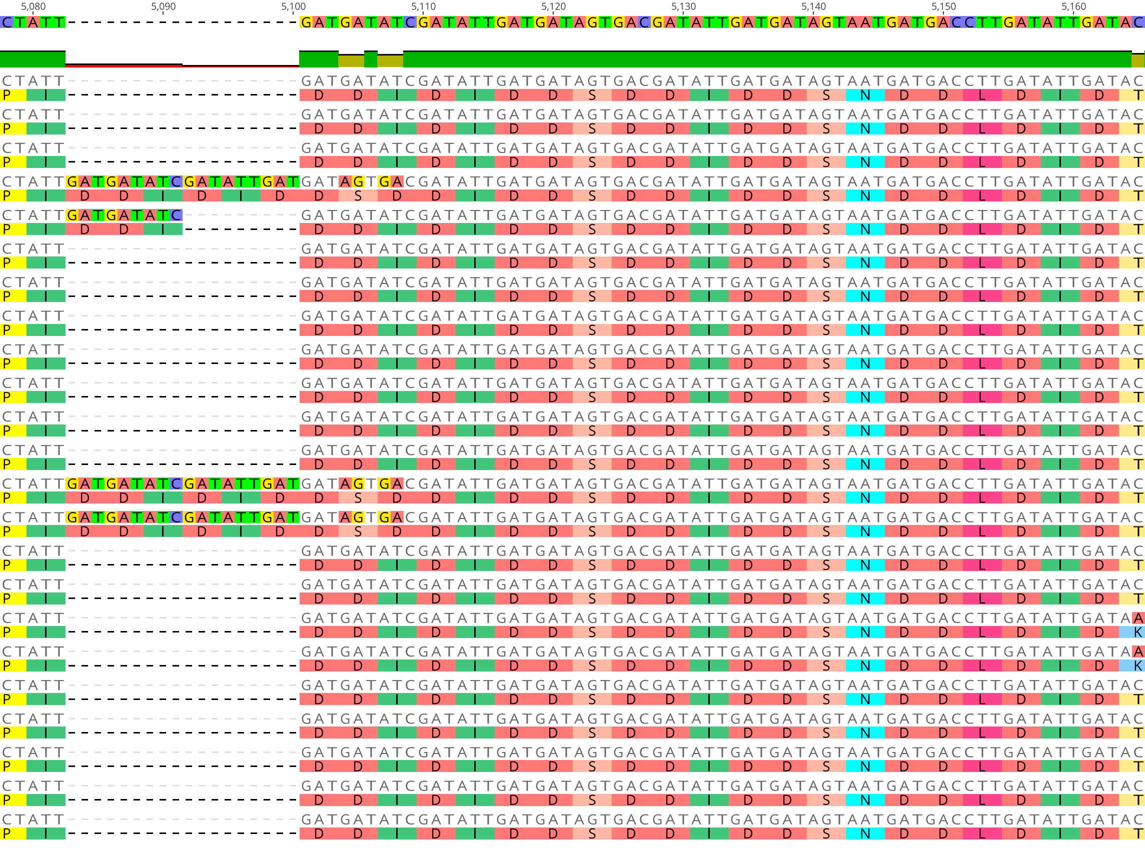

5,080
5,090
5,100
5,110
5,120
5,130
5,140
5,150
5,160
CTATT
GATGATATCGATATTGATGATAGTGACGATATTGATGATAGTAATGATGACCTTGATATTGATAC

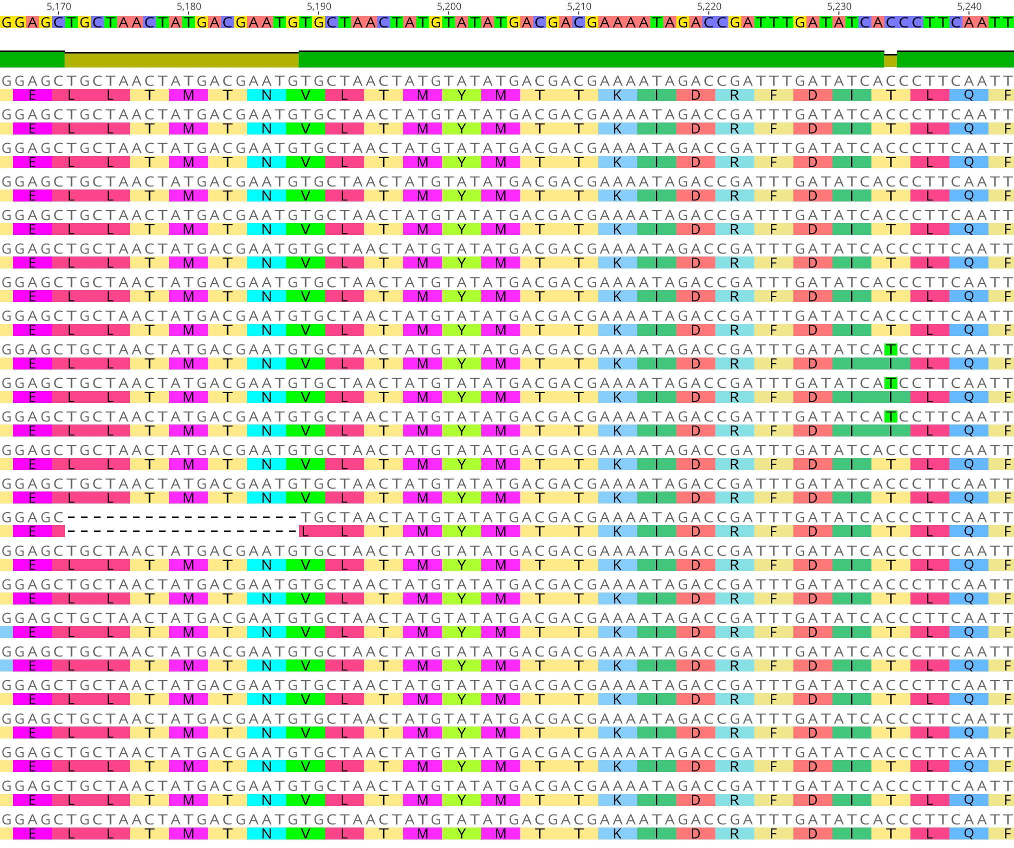

5,170 5,180 5,190 5,200 5,210 5,220 5,230 5,240
GGAGCTGCTAACTATGACGAATGTGCTAACTATGTATATGACGACGAAAATAGACCGATTTGATATCACCCTTCAATT
GGAGCTGCTAACTATGACGAATGTGCTAACTATGTATATGACGACGAAAATAGACCGATTTGATATCACCCTTCAATT
E L L T M T N V L T M Y M T T K I D R F D I T L Q F
GGAGCTGCTAACTATGACGAATGTGCTAACTATGTATATGACGACGAAAATAGACCGATTTGATATCACCCTTCAATT
E L L T M T N V L T M Y M T T K I D R F D I T L Q F
GGAGCTGCTAACTATGACGAATGTGCTAACTATGTATATGACGACGAAAATAGACCGATTTGATATCACCCTTCAATT
E L L T M T N V L T M Y M T T K I D R F D I T L Q F
GGAGCTGCTAACTATGACGAATGTGCTAACTATGTATATGACGACGAAAATAGACCGATTTGATATCACCCTTCAATT
E L L T M T N V L T M Y M T T K I D R F D I T L Q F
GGAGCTGCTAACTATGACGAATGTGCTAACTATGTATATGACGACGAAAATAGACCGATTTGATATCACCCTTCAATT
E L L T M T N V L T M Y M T T K I D R F D I T L Q F
GGAGCTGCTAACTATGACGAATGTGCTAACTATGTATATGACGACGAAAATAGACCGATTTGATATCACCCTTCAATT
E L L T M T N V L T M Y M T T K I D R F D I T L Q F
GGAGCTGCTAACTATGACGAATGTGCTAACTATGTATATGACGACGAAAATAGACCGATTTGATATCACCCTTCAATT
E L L T M T N V L T M Y M T T K I D R F D I T L Q F
GGAGCTGCTAACTATGACGAATGTGCTAACTATGTATATGACGACGAAAATAGACCGATTTGATATCACCCTTCAATT
E L L T M T N V L T M Y M T T K I D R F D I T L Q F
GGAGCTGCTAACTATGACGAATGTGCTAACTATGTATATGACGACGAAAATAGACCGATTTGATATCATCCTTCAATT
E L L T M T N V L T M Y M T T K I D R F D I I L Q F
GGAGCTGCTAACTATGACGAATGTGCTAACTATGTATATGACGACGAAAATAGACCGATTTGATATCATCCTTCAATT
E L L T M T N V L T M Y M T T K I D R F D I I L Q F
GGAGCTGCTAACTATGACGAATGTGCTAACTATGTATATGACGACGAAAATAGACCGATTTGATATCATCCTTCAATT
E L L T M T N V L T M Y M T T K I D R F D I I L Q F
GGAGCTGCTAACTATGACGAATGTGCTAACTATGTATATGACGACGAAAATAGACCGATTTGATATCACCCTTCAATT
E L L T M T N V L T M Y M T T K I D R F D I T L Q F
GGAGCTGCTAACTATGACGAATGTGCTAACTATGTATATGACGACGAAAATAGACCGATTTGATATCACCCTTCAATT
E L L T M T N V L T M Y M T T K I D R F D I T L Q F
GGAGC------------------TGCTAACTATGTATATGACGACGAAAATAGACCGATTTGATATCACCCTTCAATT
E ------------------ L L T M Y M T T K I D R F D I T L Q F
GGAGCTGCTAACTATGACGAATGTGCTAACTATGTATATGACGACGAAAATAGACCGATTTGATATCACCCTTCAATT
E L L T M T N V L T M Y M T T K I D R F D I T L Q F
GGAGCTGCTAACTATGACGAATGTGCTAACTATGTATATGACGACGAAAATAGACCGATTTGATATCACCCTTCAATT
E L L T M T N V L T M Y M T T K I D R F D I T L Q F
GGAGCTGCTAACTATGACGAATGTGCTAACTATGTATATGACGACGAAAATAGACCGATTTGATATCACCCTTCAATT
E L L T M T N V L T M Y M T T K I D R F D I T L Q F
GGAGCTGCTAACTATGACGAATGTGCTAACTATGTATATGACGACGAAAATAGACCGATTTGATATCACCCTTCAATT
E L L T M T N V L T M Y M T T K I D R F D I T L Q F
GGAGCTGCTAACTATGACGAATGTGCTAACTATGTATATGACGACGAAAATAGACCGATTTGATATCACCCTTCAATT
E L L T M T N V L T M Y M T T K I D R F D I T L Q F
GGAGCTGCTAACTATGACGAATGTGCTAACTATGTATATGACGACGAAAATAGACCGATTTGATATCACCCTTCAATT
E L L T M T N V L T M Y M T T K I D R F D I T L Q F
GGAGCTGCTAACTATGACGAATGTGCTAACTATGTATATGACGACGAAAATAGACCGATTTGATATCACCCTTCAATT
E L L T M T N V L T M Y M T T K I D R F D I T L Q F
GGAGCTGCTAACTATGACGAATGTGCTAACTATGTATATGACGACGAAAATAGACCGATTTGATATCACCCTTCAATT
E L L T M T N V L T M Y M T T K I D R F D I T L Q F
GGAGCTGCTAACTATGACGAATGTGCTAACTATGTATATGACGACGAAAATAGACCGATTTGATATCACCCTTCAATT
E L L T M T N V L T M Y M T T K I D R F D I T L Q F

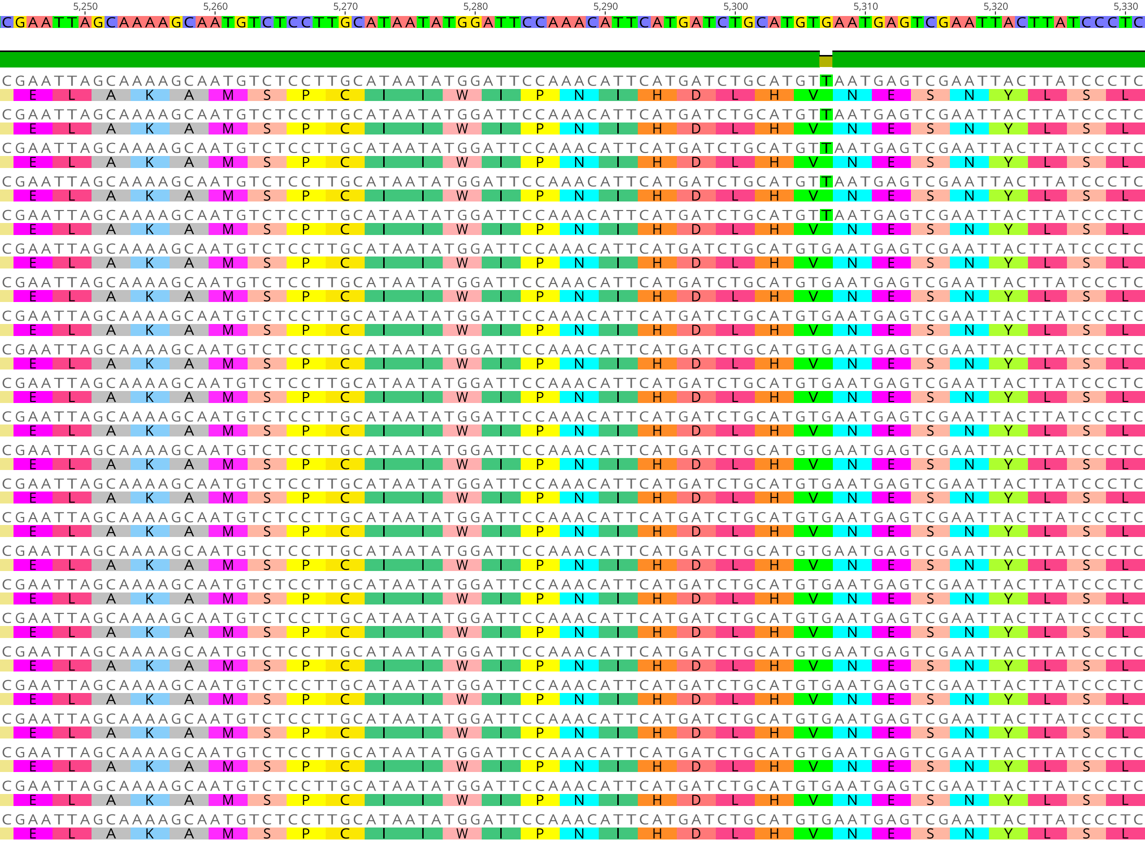

5,250 5,260 5,270 5,280 5,290 5,300 5,310 5,320 5,330
CGAATTAGCAAAAGCAATGTCTCCTTGCATAATATGGATTCCAAACATTCATGATCTGCATGTGAATGAGTCGAATTACTTATCCCTC
CGAATTAGCAAAAGCAATGTCTCCTTGCATAATATGGATTCCAAACATTCATGATCTGCATGTTAATGAGTCGAATTACTTATCCCTC
E L A K A M S P C I I W I P N I H D L H V N E S N Y L S L
CGAATTAGCAAAAGCAATGTCTCCTTGCATAATATGGATTCCAAACATTCATGATCTGCATGTTAATGAGTCGAATTACTTATCCCTC
E L A K A M S P C I I W I P N I H D L H V N E S N Y L S L
CGAATTAGCAAAAGCAATGTCTCCTTGCATAATATGGATTCCAAACATTCATGATCTGCATGTTAATGAGTCGAATTACTTATCCCTC
E L A K A M S P C I I W I P N I H D L H V N E S N Y L S L
CGAATTAGCAAAAGCAATGTCTCCTTGCATAATATGGATTCCAAACATTCATGATCTGCATGTTAATGAGTCGAATTACTTATCCCTC
E L A K A M S P C I I W I P N I H D L H V N E S N Y L S L
CGAATTAGCAAAAGCAATGTCTCCTTGCATAATATGGATTCCAAACATTCATGATCTGCATGTTAATGAGTCGAATTACTTATCCCTC
E L A K A M S P C I I W I P N I H D L H V N E S N Y L S L
CGAATTAGCAAAAGCAATGTCTCCTTGCATAATATGGATTCCAAACATTCATGATCTGCATGTGAATGAGTCGAATTACTTATCCCTC
E L A K A M S P C I I W I P N I H D L H V N E S N Y L S L
CGAATTAGCAAAAGCAATGTCTCCTTGCATAATATGGATTCCAAACATTCATGATCTGCATGTGAATGAGTCGAATTACTTATCCCTC
E L A K A M S P C I I W I P N I H D L H V N E S N Y L S L
CGAATTAGCAAAAGCAATGTCTCCTTGCATAATATGGATTCCAAACATTCATGATCTGCATGTGAATGAGTCGAATTACTTATCCCTC
E L A K A M S P C I I W I P N I H D L H V N E S N Y L S L
CGAATTAGCAAAAGCAATGTCTCCTTGCATAATATGGATTCCAAACATTCATGATCTGCATGTGAATGAGTCGAATTACTTATCCCTC
E L A K A M S P C I I W I P N I H D L H V N E S N Y L S L
CGAATTAGCAAAAGCAATGTCTCCTTGCATAATATGGATTCCAAACATTCATGATCTGCATGTGAATGAGTCGAATTACTTATCCCTC
E L A K A M S P C I I W I P N I H D L H V N E S N Y L S L
CGAATTAGCAAAAGCAATGTCTCCTTGCATAATATGGATTCCAAACATTCATGATCTGCATGTGAATGAGTCGAATTACTTATCCCTC
E L A K A M S P C I I W I P N I H D L H V N E S N Y L S L
CGAATTAGCAAAAGCAATGTCTCCTTGCATAATATGGATTCCAAACATTCATGATCTGCATGTGAATGAGTCGAATTACTTATCCCTC
E L A K A M S P C I I W I P N I H D L H V N E S N Y L S L
CGAATTAGCAAAAGCAATGTCTCCTTGCATAATATGGATTCCAAACATTCATGATCTGCATGTGAATGAGTCGAATTACTTATCCCTC
E L A K A M S P C I I W I P N I H D L H V N E S N Y L S L
CGAATTAGCAAAAGCAATGTCTCCTTGCATAATATGGATTCCAAACATTCATGATCTGCATGTGAATGAGTCGAATTACTTATCCCTC
E L A K A M S P C I I W I P N I H D L H V N E S N Y L S L
CGAATTAGCAAAAGCAATGTCTCCTTGCATAATATGGATTCCAAACATTCATGATCTGCATGTGAATGAGTCGAATTACTTATCCCTC
E L A K A M S P C I I W I P N I H D L H V N E S N Y L S L
CGAATTAGCAAAAGCAATGTCTCCTTGCATAATATGGATTCCAAACATTCATGATCTGCATGTGAATGAGTCGAATTACTTATCCCTC
E L A K A M S P C I I W I P N I H D L H V N E S N Y L S L
CGAATTAGCAAAAGCAATGTCTCCTTGCATAATATGGATTCCAAACATTCATGATCTGCATGTGAATGAGTCGAATTACTTATCCCTC
E L A K A M S P C I I W I P N I H D L H V N E S N Y L S L
CGAATTAGCAAAAGCAATGTCTCCTTGCATAATATGGATTCCAAACATTCATGATCTGCATGTGAATGAGTCGAATTACTTATCCCTC
E L A K A M S P C I I W I P N I H D L H V N E S N Y L S L
CGAATTAGCAAAAGCAATGTCTCCTTGCATAATATGGATTCCAAACATTCATGATCTGCATGTGAATGAGTCGAATTACTTATCCCTC
E L A K A M S P C I I W I P N I H D L H V N E S N Y L S L
CGAATTAGCAAAAGCAATGTCTCCTTGCATAATATGGATTCCAAACATTCATGATCTGCATGTGAATGAGTCGAATTACTTATCCCTC
E L A K A M S P C I I W I P N I H D L H V N E S N Y L S L
CGAATTAGCAAAAGCAATGTCTCCTTGCATAATATGGATTCCAAACATTCATGATCTGCATGTGAATGAGTCGAATTACTTATCCCTC
E L A K A M S P C I I W I P N I H D L H V N E S N Y L S L
CGAATTAGCAAAAGCAATGTCTCCTTGCATAATATGGATTCCAAACATTCATGATCTGCATGTGAATGAGTCGAATTACTTATCCCTC
E L A K A M S P C I I W I P N I H D L H V N E S N Y L S L
CGAATTAGCAAAAGCAATGTCTCCTTGCATAATATGGATTCCAAACATTCATGATCTGCATGTGAATGAGTCGAATTACTTATCCCTC
E L A K A M S P C I I W I P N I H D L H V N E S N Y L S L
CGAATTAGCAAAAGCAATGTCTCCTTGCATAATATGGATTCCAAACATTCATGATCTGCATGTGAATGAGTCGAATTACTTATCCCTC
E L A K A M S P C I I W I P N I H D L H V N E S N Y L S L

5,340 5,350 5,360 5,370 5,380 5,390 5,400 5,410

GATCTATTAGAGAACTATCTCTCCAGGGATTGTGAAAGATGTTCCACTGGAAAGATTCTTGTTATTGCTTCGACTCATATTCC

GATCTATTAGAGAACTATCTCTCCAGGGATTGTGAAAGATGTTCCACTGGAAAGATTCTTGTTATTGCTTCGACTCATATTCC
D L L E N Y L S R D C E R C S T G K I L V I A S T H I P
GATCTATTAGAGAACTATCTCTCCAGGGATTGTGAAAGATGTTCCACTGGAAAGATTCTTGTTATTGCTTCGACTCATATTCC
D L L E N Y L S R D C E R C S T G K I L V I A S T H I P
GATCTATTAGAGAACTATCTCTCCAGGGATTGTGAAAGATGTTCCACTGGAAAGATTCTTGTTATTGCTTCGACTCATATTCC
D L L E N Y L S R D C E R C S T G K I L V I A S T H I P
GATCTATTAGAGAACTATCTCTCCAGGGATTGTGAAAGATGTTCCACTGGAAAGATTCTTGTTATTGCTTCGACTCATATTCC
D L L E N Y L S R D C E R C S T G K I L V I A S T H I P
GATCTATTAGAGAACTATCTCTCCAGGGATTGTGAAAGATGTTCCACTGGAAAGATTCTTGTTATTGCTTCGACTCATATTCC
D L L E N Y L S R D C E R C S T G K I L V I A S T H I P
GATCTATTAGAGAACTATCTCTCCAGGGATTGTGAAAGATGTTCCACTGGAAAGATTCTTGTTATTGCTTCGACTCATATTCC
D L L E N Y L S R D C E R C S T G K I L V I A S T H I P
GATCTATTAGAGAACTATCTCTCCAGGGATTGTGAAAGATGTTCCACTGGAAAGATTCTTGTTATTGCTTCGACTCATATTCC
D L L E N Y L S R D C E R C S T G K I L V I A S T H I P
GATCTATTAGAGAACTATCTCTCCAGGGATTGTGAAAGATGTTCCACTGGAAAGATTCTTGTTATTGCTTCGACTCATATTCC
D L L E N Y L S R D C E R C S T G K I L V I A S T H I P
GATCTATTAGAGAACTATCTCTCCAGGGATTGTGAAAGATGTTCCACTGGAAAGATTCTTGTTATTGCTTCGACTCATATTCC
D L L E N Y L S R D C E R C S T G K I L V I A S T H I P
GATCTATTAGAGAACTATCTCTCCAGGGATTGTGAAAGATGTTCCACTGGAAAGATTCTTGTTATTGCTTCGACTCATATTCC
D L L E N Y L S R D C E R C S T G K I L V I A S T H I P
GATCTATTAGAGAACTATCTCTCCAGGGATTGTGAAAGATGTTCCACTGGAAAGATTCTTGTTATTGCTTCGACTCATATTCC
D L L E N Y L S R D C E R C S T G K I L V I A S T H I P
GATCTATTAGAGAACTATCTCTCCAGGGATTGTGAAAGATGTTCCACTGGAAAGATTCTTGTTATTGCTTCGACTCATATTCC
D L L E N Y L S R D C E R C S T G K I L V I A S T H I P
GATCTATTAGAGAACTATCTCTCCAGGGATTGTGAAAGATGTTCCACTGGAAAGATTCTTGTTATTGCTTCGACTCATATTCC
D L L E N Y L S R D C E R C S T G K I L V I A S T H I P
GATCTATTAGAGAACTATCTCTCCAGGGATTGTGAAAGATGTTCCACTGGAAAGATTCTTGTTATTGCTTCGACTCATATTCC
D L L E N Y L S R D C E R C S T G K I L V I A S T H I P
GATCTATTAGAGAACTATCTCTCCAGGGATTGTGAAAGATGTTCCACTGGAAAGATTCTTGTTATTGCTTCGACTCATATTCC
D L L E N Y L S R D C E R C S T G K I L V I A S T H I P
GATCTATTAGAGAACTATCTCTCCAGGGATTGTGAAAGATGTTCCACTGGAAAGATTCTTGTTATTGCTTCGACTCATATTCC
D L L E N Y L S R D C E R C S T G K I L V I A S T H I P
GATCTATTAGAGAACTATCTCTCCAGGGATTGTGAAAGATGTTCCACTGGAAAGATTCTTGTTATTGCTTCGACTCATATTCC
D L L E N Y L S R D C E R C S T G K I L V I A S T H I P
GATCTATTAGAGAACTATCTCTCCAGGGATTGTGAAAGATGTTCCACTGGAAAGATTCTTGTTATTGCTTCGACTCATATTCC
D L L E N Y L S R D C E R C S T G K I L V I A S T H I P
GATCTATTAGAGAACTATCTCTCCAGGGATTGTGAAAGATGTTCCACTGGAAAGATTCTTGTTATTGCTTCGACTCATATTCC
D L L E N Y L S R D C E R C S T G K I L V I A S T H I P
GATCTATTAGAGAACTATCTCTCCAGGGATTGTGAAAGATGTTCCACTGGAAAGATTCTTGTTATTGCTTCGACTCATATTCC
D L L E N Y L S R D C E R C S T G K I L V I A S T H I P
GATCTATTAGAGAACTATCTCTCCAGGGATTGTGAAAGATGTTCCACTGGAAAGATTCTTGTTATTGCTTCGACTCATATTCC
D L L E N Y L S R D C E R C S T G K I L V I A S T H I P
GATCTATTAGAGAACTATCTCTCCAGGGATTGTGAAAGATGTTCCACTGGAAAGATTCTTGTTATTGCTTCGACTCATATTCC
D L L E N Y L S R D C E R C S T G K I L V I A S T H I P
GATCTATTAGAGAACTATCTCTCCAGGGATTGTGAAAGATGTTCCACTGGAAAGATTCTTGTTATTGCTTCGACTCATATTCC
D L L E N Y L S R D C E R C S T G K I L V I A S T H I P

```
5,420 5,430 5,440 5,450 5,460 5,470 5,480
CCAAAAGTGGATCCCGCTCTAATAGCTCCAAAGAAATTCAATACATGCATTAAGATACGAAGGCTTCTTCTTC

CCAAAAGTGGATCCCGCTCTAATAGCTCCAAAGAAATTCAATACATGCATTAAGATACGAAGGCTTCTTCTTC
Q K V D P A L I A P K K F N T C I K I R R L L L
CCAAAAGTGGATCCCGCTCTAATAGCTCCAAAGAAATTCAATACATGCATTAAGATACGAAGGCTTCTTCTTC
Q K V D P A L I A P K K F N T C I K I R R L L L
CCAAAAGTGGATCCCGCTCTAATAGCTCCAAAGAAATTCAATACATGCATTAAGATACGAAGGCTTCTTCTTC
Q K V D P A L I A P K K F N T C I K I R R L L L
CCAAAAGTGGATCCCGCTCTAATAGCTCCAAAGAAATTCAATACATGCATTAAGATACGAAGGCTTCTTCTTC
Q K V D P A L I A P K K F N T C I K I R R L L L
CCAAAAGTGGATCCCGCTCTAATAGCTCCAAATAAATTCAATACATGCATTAAGATACGAAGGCTTCTTCTTC
Q K V D P A L I A P N K F N T C I K I R R L L L
CCAAAAGTGGATCCCGCTCTAATAGCTCCAAAGAAATTCAATACATGCATTAAGATACGAAGGCTTCTTCTTC
Q K V D P A L I A P K K F N T C I K I R R L L L
CCAAAAGTGGATCCCGCTCTAATAGCTCCAAAGAAATTCAATACATGCATTAAGATACGAAGGCTTCTTCTTC
Q K V D P A L I A P K K F N T C I K I R R L L L
CCAAAAGTGGATCCCGCTCTAATAGCTCCAAAGAAATTCAATACATGCATTAAGATACGAAGGCTTCTTCTTC
Q K V D P A L I A P K K F N T C I K I R R L L L
CCAAAAGTGGATCCCGCTCTAATAGCTCCAAAGAAATTCAATACATGCATTAAGATACGAAGGCTTCTTCTTC
Q K V D P A L I A P K K F N T C I K I R R L L L
CCAAAAGTGGATCCCGCTCTAATAGCTCCAAAGAAATTCAATACATGCATTAAGATACGAAGGCTTCTTCTTC
Q K V D P A L I A P K K F N T C I K I R R L L L
CCAAAAGTGGATCCCGCTCTAATAGCTCCAAAGAAATTCAATACATGCATTAAGATACGAAGGCTTCTTCTTC
Q K V D P A L I A P K K F N T C I K I R R L L L
CCAAAAGTGGATCCCGCTCTAATAGCTCCAAAGAAATTCAATACATGCATTAAGATACGAAGGCTTCTTCTTC
Q K V D P A L I A P K K F N T C I K I R R L L L
CCAAAAGTGGATCCCGCTCTAATAGCTCCAAATAAATTCAATACATGCATTAAGATACGAAGGCTTCTTCTTC
Q K V D P A L I A P N K F N T C I K I R R L L L
CCAAAAGTGGATCCCGCTCTAATAGCTCCAAATAAATTCAATACATGCATTAAGATACGAAGGCTTCTTCTTC
Q K V D P A L I A P N K F N T C I K I R R L L L
CCAAAAGTGGATCCCGCTCTAATAGCTCCAAAGAAATTCAATACATGCATTAAGATACGAAGGCTTCTTCTTC
Q K V D P A L I A P K K F N T C I K I R R L L L
CCAAAAGTGGATCCCGCTCTAATAGCTCCAAAGAAATTCAATACATGCATTAAGATACGAAGGCTTCTTCTTC
Q K V D P A L I A P K K F N T C I K I R R L L L
CCAAAAGTGGATCCCGCTCTAATAGCTCCAAAGAAATTCAATACATGCATTAAGATACGAAGGCTTCTTCTTC
Q K V D P A L I A P K K F N T C I K I R R L L L
CCAAAAGTGGATCCCGCTCTAATAGCTCCAAAGAAATTCAATACATGCATTAAGATACGAAGGCTTCTTCTTC
Q K V D P A L I A P K K F N T C I K I R R L L L
CCAAAAGTGGATCCCGCTCTAATAGCTCCAAAGAAATTCAATACATGCATTAAGATACGAAGGCTTCTTCTTC
Q K V D P A L I A P K K F N T C I K I R R L L L
CCAAAAGTGGATCCCGCTCTAATAGCTCCAAAGAAATTCAATACATGCATTAAGATACGAAGGCTTCTTCTTC
Q K V D P A L I A P K K F N T C I K I R R L L L
CCAAAAGTGGATCCCGCTCTAATAGCTCCAAAGAAATTCAATACATGCATTAAGATACGAAGGCTTCTTCTTC
Q K V D P A L I A P K K F N T C I K I R R L L L
CCAAAAGTGGATCCCGCTCTAATAGCTCCAAAGAAATTCAATACATGCATTAAGATACGAAGGCTTCTTCTTC
Q K V D P A L I A P K K F N T C I K I R R L L L
CCAAAAGTGGATCCCGCTCTAATAGCTCCAAAGAAATTCAATACATGCATTAAGATACGAAGGCTTCTTCTTC
Q K V D P A L I A P K K F N T C I K I R R L L L
```

5,490 5,500 5,510 5,520 5,530 5,540 5,550 5,560 5,57

CACAACAACGAAAGCACTTTTTCATTCTTTCATATACTAGAGGATTTCACTTGGAAAAGAAGATGTTCCATACTAACGGATT

CACAACAACGAAAGCACTTTTTCATTCTTTCATATACTAGAGGATTTCACTTGGAAAAGAAGATGTTCCATACTAACGGATT
P Q Q R K H F F I L S Y T R G F H L E K K M F H T N G F
CACAACAACGAAAGCACTTTTTCATTCTTTCATATACTAGAGGATTTCACTTGGAAAAGAAGATGTTCCATACTAACGGATT
P Q Q R K H F F I L S Y T R G F H L E K K M F H T N G F
TACAACAACGAAAGCACTTTTTCATTCTTTCATATACTAGAGGATTTCACTTGGAAAAGAAGATGTTCCATACTAACGGATT
L Q Q R K H F F I L S Y T R G F H L E K K M F H T N G F
CACAACAACGAAAGCACTTTTTCATTCTTTCATATACTAGAGGATTTCACTTGGAAAAGAAGATGTTCCATACTAACGGATT
P Q Q R K H F F I L S Y T R G F H L E K K M F H T N G F
CACAACAACGAAAGCACTTTTTCATTCTTTCATATACTAGAGGATTTCACTTGGAAAAGAAGATGTTCCATACTAACGGATT
P Q Q R K H F F I L S Y T R G F H L E K K M F H T N G F
CACAACAACGAAAGCACTTTTTCATTCTTTCATATACTAGAGGATTTCACTTGGAAAAGAAGATGTTCCATACTAACGGATT
P Q Q R K H F F I L S Y T R G F H L E K K M F H T N G F
CACAACAACGAAAGCACTTTTTCATTCTTTCATATACTAGAGGATTTCACTTGGAAAAGAAGATGTTCCATACTAACGGATT
P Q Q R K H F F I L S Y T R G F H L E K K M F H T N G F
CACAACAACGAAAGCACTTTTTCATTCTTTCATATACTAGAGGATTTCACTTGGAAAAGAAGATGTTCCATACTAACGGATT
P Q Q R K H F F I L S Y T R G F H L E K K M F H T N G F
CACAACAACGAAAGCACTTTTTCATTCTTTCATATACTAGAGGATTTCACTTGGAAAAGAAGATGTTCCATACTAACGGATT
P Q Q R K H F F I L S Y T R G F H L E K K M F H T N G F
CACAACAACGAAAGCACTTTTTCATTCTTTCATATACTAGAGGATTTCACTTGGAAAAGAAGATGTTCCATACTAACGGATT
P Q Q R K H F F I L S Y T R G F H L E K K M F H T N G F
CACAACAACGAAAGCACTTTTTCATTCTTTCATATACTAGAGGATTTCACTTGGAAAAGAAGATGTTCCATACTAACGGATT
P Q Q R K H F F I L S Y T R G F H L E K K M F H T N G F
CACAACAACGAAAGCACTTTTTCATTCTTTCATATACTAGAGGATTTCACTTGGAAAAGAAGATGTTCCATACTAACGGATT
P Q Q R K H F F I L S Y T R G F H L E K K M F H T N G F
CACAACAACGAAAGCACTTTTTCATTCTTTCATATACTAGAGGATTTCACTTGGAAAAGAAGATGTTCCATACTAACGGATT
P Q Q R K H F F I L S Y T R G F H L E K K M F H T N G F
CACAACAACGAAAGCACTTTTTCATTCTTTCATATACTAGAGGATTTCACTTGGAAAAGAAGATGTTCCATACTAACGGATT
P Q Q R K H F F I L S Y T R G F H L E K K M F H T N G F
CACAACAACGAAAGCACTTTTTCATTCTTTCATATACTAGAGGATTTCACTTGGAAAAGAAGATGTTCCATACTAACGGATT
P Q Q R K H F F I L S Y T R G F H L E K K M F H T N G F
CACAACAACGAAAGCACTTTTTCATTCTTTCATATACTAGAGGATTTCACTTGGAAAAGAAGATGTTCCATACTAACGGATT
P Q Q R K H F F I L S Y T R G F H L E K K M F H T N G F
CACAACAACGAAAGCACTTTTTCATTCTTTCATATACTAGAGGATTTCACTTGGAAAAGAAGATGTTCCATACTAACGGATT
P Q Q R K H F F I L S Y T R G F H L E K K M F H T N G F
CACAACAACGAAAGCACTTTTTCATTCTTTCATATACTAGAGGATTTCACTTGGAAAAGAAGATGTTCCATACTAACGGATT
P Q Q R K H F F I L S Y T R G F H L E K K M F H T N G F
CACAACAACGAAAGCACTTTTTCATTCTTTCATATACTAGAGGATTTCACTTGGAAAAGAAGATGTTCCATACTAACGGATT
P Q Q R K H F F I L S Y T R G F H L E K K M F H T N G F
CACAACAACGAAAGCACTTTTTCATTCTTTCATATACTAGAGGATTTCACTTGGAAAAGAAGATGTTCCATACTAACGGATT
P Q Q R K H F F I L S Y T R G F H L E K K M F H T N G F
CACAACAACGAAAGCACTTTTTCATTCTTTCATATACTAGAGGATTTCACTTGGAAAAGAAGATGTTCCATACTAACGGATT
P Q Q R K H F F I L S Y T R G F H L E K K M F H T N G F
CACAACAACGAAAGCACTTTTTCATTCTTTCATATACTAGAGGATTTCACTTGGAAAAGAAGATGTTCCATACTAACGGATT
P Q Q R K H F F I L S Y T R G F H L E K K M F H T N G F
CACAACAACGAAAGCACTTTTTCATTCTTTCATATACTAGAGGATTTCACTTGGAAAAGAAGATGTTCCATACTAACGGATT
P Q Q R K H F F I L S Y T R G F H L E K K M F H T N G F

70 5,580 5,590 5,600 5,610 5,620 5,630 5,640

CGGGTCCATAACCATGGGTTCCAATGCGCGAGATCTTGTAGCACTTATCAATGAGGCCCTATCAATTAGTATTA

CGGGTCCATAACCATGGGTTCCAATGCGCGAGATCTTGTAGCACTTATCAATGAGGCCCTATCAATTAGTATTA
G S I T M G S N A R D L V A L I N E A L S I S I

CGGGTCCATAACCATGGGTTCCAATGCGCGAGATCTTGTAGCACTTATCAATGAGGCCCTATCAATTAGTATTA
G S I T M G S N A R D L V A L I N E A L S I S I

CGGGTCCATAACCATGGGTTCCAATGCGCGAGATCTTGTAGCACTTATCAATGAGGCCCTATCAATTAGTATTA
G S I T M G S N A R D L V A L I N E A L S I S I

CGGGTCCATAACCATGGGTTCCAATGCGCGAGATCTTGTAGCACTTATCAATGAGGCCCTATCAATTAGTATTA
G S I T M G S N A R D L V A L I N E A L S I S I

CGGGTCCATAACCATGGGTTCCAATGCGCGAGATCTTGTAGCACTTATCAATGAGGCCCTATCAATTAGTATTA
G S I T M G S N A R D L V A L I N E A L S I S I

CGGGTCCATAACCATGGGTTCCAATGCGCGAGATCTTGTAGCACTTATCAATGAGGCCCTATCAATTAGTATTA
G S I T M G S N A R D L V A L I N E A L S I S I

CGGGTCCATAACCATGGGTTCCAATGCGCGAGATCTTGTAGCACTTATCAATGAGGCCCTATCAATTAGTATTA
G S I T M G S N A R D L V A L I N E A L S I S I

CGGGTCCATAACCATGGGTTCCAATGCGCGAGATCTTGTAGCACTTATCAATGAGGCCCTATCAATTAGTATTA
G S I T M G S N A R D L V A L I N E A L S I S I

CGGGTCCATAACCATGGGTTCCAATGCGCGAGATCTTGTAGCACTTATCAATGAGGCCCTATCAATTAGTATTA
G S I T M G S N A R D L V A L I N E A L S I S I

CGGGTCCATAACCATGGGTTCCAATGCGCGAGATCTTGTAGCACTTATCAATGAGGCCCTATCAATTAGTATTA
G S I T M G S N A R D L V A L I N E A L S I S I

CGGGTCCATAACCATGGGTTCCAATGCGCGAGATCTTGTAGCACTTATCAATGAGGCCCTATCAATTAGTATTA
G S I T M G S N A R D L V A L I N E A L S I S I

CGGGTCCATAACCATGGGTTCCAATGCGCGAGATCTTGTAGCACTTATCAATGAGGCCCTATCAATTAGTATTA
G S I T M G S N A R D L V A L I N E A L S I S I

CGGGTCCATAACCATGGGTTCCAATGCGCGAGATCTTGTAGCACTTATCAATGAGGCCCTATCAATTAGTATTA
G S I T M G S N A R D L V A L I N E A L S I S I

CGGGTCCATAACCATGGGTTCCAATGCGCGAGATCTTGTAGCACTTATCAATGAGGCCCTATCAATTAGTATTA
G S I T M G S N A R D L V A L I N E A L S I S I

CGGGTCCATAACCATGGGTTCCAATGCGCGAGATCTTGTAGCACTTATCAATGAGGCCCTATCAATTAGTATTA
G S I T M G S N A R D L V A L I N E A L S I S I

CGGGTCCATAACCATGGGTTCCAATGCGCGAGATCTTGTAGCACTTATCAATGAGGCCCTATCAATTAGTATTA
G S I T M G S N A R D L V A L I N E A L S I S I

CGGGTCCATAACCATGGGTTCCAATGCGCGAGATCTTGTAGCACTTATCAATGAGGCCCTATCAATTAGTATTA
G S I T M G S N A R D L V A L I N E A L S I S I

CGGGTCCATAACCATGGGTTCCAATGCGCGAGATCTTGTAGCACTTATCAATGAGGCCCTATCAATTAGTATTA
G S I T M G S N A R D L V A L I N E A L S I S I

CGGGTCCATAACCATGGGTTCCAATGCGCGAGATCTTGTAGCACTTATCAATGAGGCCCTATCAATTAGTATTA
G S I T M G S N A R D L V A L I N E A L S I S I

CGGGTCCATAACCATGGGTTCCAATGCGCGAGATCTTGTAGCACTTATCAATGAGGCCCTATCAATTAGTATTA
G S I T M G S N A R D L V A L I N E A L S I S I

CGGGTCCATAACCATGGGTTCCAATGCGCGAGATCTTGTAGCACTTATCAATGAGGCCCTATCAATTAGTATTA
G S I T M G S N A R D L V A L I N E A L S I S I

CGGGTCCATAACCATGGGTTCCAATGCGCGAGATCTTGTAGCACTTATCAATGAGGCCCTATCAATTAGTATTA
G S I T M G S N A R D L V A L I N E A L S I S I

CGGGTCCATAACCATGGGTTCCAATGCGCGAGATCTTGTAGCACTTATCAATGAGGCCCTATCAATTAGTATTA
G S I T M G S N A R D L V A L I N E A L S I S I

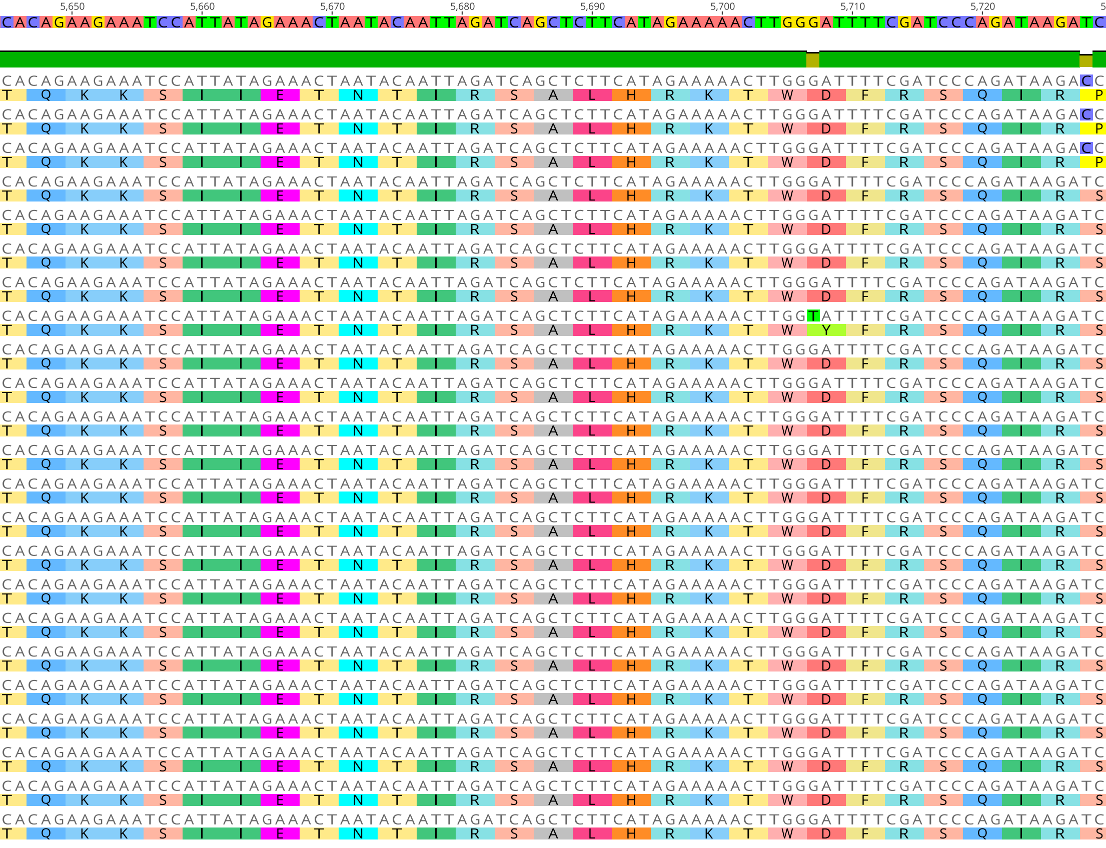
5,650 5,660 5,670 5,680 5,690 5,700 5,710 5,720 5
CACAGAAGAAATCCATTATAGAAACTAATACAATTAGATCAGCTCTTCATAGAAAAACTTGGGATTTTCGATCCCAGATAAGATC
CACAGAAGAAATCCATTATAGAAACTAATACAATTAGATCAGCTCTTCATAGAAAAACTTGGGATTTTCGATCCCAGATAAGACC
T Q K K S I I E T N T I R S A L H R K T W D F R S Q I R P
CACAGAAGAAATCCATTATAGAAACTAATACAATTAGATCAGCTCTTCATAGAAAAACTTGGGATTTTCGATCCCAGATAAGACC
T Q K K S I I E T N T I R S A L H R K T W D F R S Q I R P
CACAGAAGAAATCCATTATAGAAACTAATACAATTAGATCAGCTCTTCATAGAAAAACTTGGGATTTTCGATCCCAGATAAGAC
T Q K K S I I E T N T I R S A L H R K T W D F R S Q I R P
CACAGAAGAAATCCATTATAGAAACTAATACAATTAGATCAGCTCTTCATAGAAAAACTTGGGATTTTCGATCCCAGATAAGATC
T Q K K S I I E T N T I R S A L H R K T W D F R S Q I R S
CACAGAAGAAATCCATTATAGAAACTAATACAATTAGATCAGCTCTTCATAGAAAAACTTGGGATTTTCGATCCCAGATAAGATC
T Q K K S I I E T N T I R S A L H R K T W D F R S Q I R S
CACAGAAGAAATCCATTATAGAAACTAATACAATTAGATCAGCTCTTCATAGAAAAACTTGGGATTTTCGATCCCAGATAAGATC
T Q K K S I I E T N T I R S A L H R K T W D F R S Q I R S
CACAGAAGAAATCCATTATAGAAACTAATACAATTAGATCAGCTCTTCATAGAAAAACTTGGGATTTTCGATCCCAGATAAGATC
T Q K K S I I E T N T I R S A L H R K T W D F R S Q I R S
CACAGAAGAAATCCATTATAGAAACTAATACAATTAGATCAGCTCTTCATAGAAAAACTTGGTATTTTCGATCCCAGATAAGATC
T Q K K S I I E T N T I R S A L H R K T W Y F R S Q I R S
CACAGAAGAAATCCATTATAGAAACTAATACAATTAGATCAGCTCTTCATAGAAAAACTTGGGATTTTCGATCCCAGATAAGATC
T Q K K S I I E T N T I R S A L H R K T W D F R S Q I R S
CACAGAAGAAATCCATTATAGAAACTAATACAATTAGATCAGCTCTTCATAGAAAAACTTGGGATTTTCGATCCCAGATAAGATC
T Q K K S I I E T N T I R S A L H R K T W D F R S Q I R S
CACAGAAGAAATCCATTATAGAAACTAATACAATTAGATCAGCTCTTCATAGAAAAACTTGGGATTTTCGATCCCAGATAAGATC
T Q K K S I I E T N T I R S A L H R K T W D F R S Q I R S
CACAGAAGAAATCCATTATAGAAACTAATACAATTAGATCAGCTCTTCATAGAAAAACTTGGGATTTTCGATCCCAGATAAGATC
T Q K K S I I E T N T I R S A L H R K T W D F R S Q I R S
CACAGAAGAAATCCATTATAGAAACTAATACAATTAGATCAGCTCTTCATAGAAAAACTTGGGATTTTCGATCCCAGATAAGATC
T Q K K S I I E T N T I R S A L H R K T W D F R S Q I R S
CACAGAAGAAATCCATTATAGAAACTAATACAATTAGATCAGCTCTTCATAGAAAAACTTGGGATTTTCGATCCCAGATAAGATC
T Q K K S I I E T N T I R S A L H R K T W D F R S Q I R S
CACAGAAGAAATCCATTATAGAAACTAATACAATTAGATCAGCTCTTCATAGAAAAACTTGGGATTTTCGATCCCAGATAAGATC
T Q K K S I I E T N T I R S A L H R K T W D F R S Q I R S
CACAGAAGAAATCCATTATAGAAACTAATACAATTAGATCAGCTCTTCATAGAAAAACTTGGGATTTTCGATCCCAGATAAGATC
T Q K K S I I E T N T I R S A L H R K T W D F R S Q I R S
CACAGAAGAAATCCATTATAGAAACTAATACAATTAGATCAGCTCTTCATAGAAAAACTTGGGATTTTCGATCCCAGATAAGATC
T Q K K S I I E T N T I R S A L H R K T W D F R S Q I R S
CACAGAAGAAATCCATTATAGAAACTAATACAATTAGATCAGCTCTTCATAGAAAAACTTGGGATTTTCGATCCCAGATAAGATC
T Q K K S I I E T N T I R S A L H R K T W D F R S Q I R S
CACAGAAGAAATCCATTATAGAAACTAATACAATTAGATCAGCTCTTCATAGAAAAACTTGGGATTTTCGATCCCAGATAAGATC
T Q K K S I I E T N T I R S A L H R K T W D F R S Q I R S
CACAGAAGAAATCCATTATAGAAACTAATACAATTAGATCAGCTCTTCATAGAAAAACTTGGGATTTTCGATCCCAGATAAGATC
T Q K K S I I E T N T I R S A L H R K T W D F R S Q I R S
CACAGAAGAAATCCATTATAGAAACTAATACAATTAGATCAGCTCTTCATAGAAAAACTTGGGATTTTCGATCCCAGATAAGATC
T Q K K S I I E T N T I R S A L H R K T W D F R S Q I R S
CACAGAAGAAATCCATTATAGAAACTAATACAATTAGATCAGCTCTTCATAGAAAAACTTGGGATTTTCGATCCCAGATAAGATC
T Q K K S I I E T N T I R S A L H R K T W D F R S Q I R S
CACAGAAGAAATCCATTATAGAAACTAATACAATTAGATCAGCTCTTCATAGAAAAACTTGGGATTTTCGATCCCAGATAAGATC
T Q K K S I I E T N T I R S A L H R K T W D F R S Q I R S

,730 5,740 5,750 5,760 5,770 5,780 5,790 5,800 5,810

GGTTCAGGATCATGGGATCCTTTTCTATCAGATAGGAAGGGCTGTTACACAAAATGTACTTCTAAGTAATTGCCCCATAGATC

GGTTCAGGATCATGGGATCCTTTTCTATCAGATAGGAAGGGCTGTTACACAAAATGTACTTCTAAGTAATTGCCCCATAGATC
V Q D H G I L F Y Q I G R A V T Q N V L L S N C P I D

GGTTCAGGATCATGGGATCCTTTTCTATCAGATAGGAAGGGCTGTTACACAAAATGTACTTCTAAGTAATTGCCCCATAGATC
V Q D H G I L F Y Q I G R A V T Q N V L L S N C P I D

GGTTCAGGATCATGGGATCCTTTTCTATCAGATAGGAAGGGCTGTTACACAAAATGTACTTCTAAGTAATTGCCCCATAGATC
V Q D H G I L F Y Q I G R A V T Q N V L L S N C P I D

GGTTCAGGATCATGGGATCCTTTTCTATCAGATAGGAAGGGCTGTTACACAAAATGTACTTCTAAGTAATTGCCCCATAGATC
V Q D H G I L F Y Q I G R A V T Q N V L L S N C P I D

GGTTCAGGATCATGGGATCCTTTTCTATCAGATAGGAAGGGCTGTTACACAAAATGTACTTCTAAGTAATTGCCCCATAGATC
V Q D H G I L F Y Q I G R A V T Q N V L L S N C P I D

GGTTCAGGATCATGGGATCCTTTTCTATCAGATAGGAAGGGCTGTTACACAAAATGTACTTCTAAGTAATTGCCCCATAGATC
V Q D H G I L F Y Q I G R A V T Q N V L L S N C P I D

GGTTCAGGATCATGGGATCCTTTTCTATCAGATAGGAAGGGCTGTTACACAAAATGTACTTCTAAGTAATTGCCCCATAGATC
V Q D H G I L F Y Q I G R A V T Q N V L L S N C P I D

GGTTCAGGATCATGGGATCCTTTTCTATCAGATAGGAAGGGCTGTTACACAAAATGTACTTCTAAGTAATTGCCCCATAGATC
V Q D H G I L F Y Q I G R A V T Q N V L L S N C P I D

GGTTCAGGATCATGGGATCCTTTTCTATCAGATAGGAAGGGCTGTTACACAAAATGTACTTCTAAGTAATTGCCCCATAGATC
V Q D H G I L F Y Q I G R A V T Q N V L L S N C P I D

GGTTCAGGATCATGGGATCCTTTTCTATCAGATAGGAAGGGCTGTTACACAAAATGTACTTCTAAGTAATTGCCCCATAGATC
V Q D H G I L F Y Q I G R A V T Q N V L L S N C P I D

GGTTCAGGATCATGGGATCCTTTTCTATCAGATAGGAAGGGCTGTTACACAAAATGTACTTCTAAGTAATTGCCCCATAGATC
V Q D H G I L F Y Q I G R A V T Q N V L L S N C P I D

GGTTCAGGATCATGGGATCCTTTTCTATCAGATAGGAAGGGCTGTTACACAAAATGTACTTCTAAGTAATTGCCCCATAGATC
V Q D H G I L F Y Q I G R A V T Q N V L L S N C P I D

GGTTCAGGATCATGGGATCCTTTTCTATCAGATAGGAAGGGCTGTTACACAAAATGTACTTCTAAGTAATTGCCCCATAGATC
V Q D H G I L F Y Q I G R A V T Q N V L L S N C P I D

GGTTCAGGATCATGGGATCCTTTTCTATCAGATAGGAAGGGCTGTTACACAAAATGTACTTCTAAGTAATTGCCCCATAGATC
V Q D H G I L F Y Q I G R A V T Q N V L L S N C P I D

GGTTCAGGATCATGGGATCCTTTTCTATCAGATAGGAAGGGCTGTTACACAAAATGTACTTCTAAGTAATTGCCCCATAGATC
V Q D H G I L F Y Q I G R A V T Q N V L L S N C P I D

GGTTCAGGATCATGGGATCCTTTTCTATCAGATAGGAAGGGCTGTTACACAAAATGTACTTCTAAGTAATTGCCCCATAGATC
V Q D H G I L F Y Q I G R A V T Q N V L L S N C P I D

GGTTCAGGATCATGGGATCCTTTTCTATCAGATAGGAAGGGCTGTTACACAAAATGTACTTCTAAGTAATTGCCCCATAGATC
V Q D H G I L F Y Q I G R A V T Q N V L L S N C P I D

GGTTCAGGATCATGGGATCCTTTTCTATCAGATAGGAAGGGCTGTTACACAAAATGTACTTCTAAGTAATTGCCCCATAGATC
V Q D H G I L F Y Q I G R A V T Q N V L L S N C P I D

GGTTCAGGATCATGGGATCCTTTTCTATCAGATAGGAAGGGCTGTTACACAAAATGTACTTCTAAGTAATTGCCCCATAGATC
V Q D H G I L F Y Q I G R A V T Q N V L L S N C P I D

GGTTCAGGATCATGGGATCCTTTTCTATCAGATAGGAAGGGCTGTTACACAAAATGTACTTCTAAGTAATTGCCCCATAGATC
V Q D H G I L F Y Q I G R A V T Q N V L L S N C P I D

GGTTCAGGATCATGGGATCCTTTTCTATCAGATAGGAAGGGCTGTTACACAAAATGTACTTCTAAGTAATTGCCCCATAGATC
V Q D H G I L F Y Q I G R A V T Q N V L L S N C P I D

GGTTCAGGATCATGGGATCCTTTTCTATCAGATAGGAAGGGCTGTTACACAAAATGTACTTCTAAGTAATTGCCCCATAGATC
V Q D H G I L F Y Q I G R A V T Q N V L L S N C P I D

GGTTCAGGATCATGGGATCCTTTTCTATCAGATAGGAAGGGCTGTTACACAAAATGTACTTCTAAGTAATTGCCCCATAGATC
V Q D H G I L F Y Q I G R A V T Q N V L L S N C P I D

5,820 5,830 5,840 5,850 5,860 5,870 5,880 5,890

CTATATCTATCTATATGAAGAAGAAATCATGTAAGGGAGGGGATTCTTATTTGTACAAATGGTACTTCGAACTTGGAAC

CTATATCTATCTATATGAAGAAGAAATCATGTAAGGGAGGGGATTCTTATTTGTACAAATGGTACTTCGAACTTGGAAC
P I S I Y M K K K S C K G G D S Y L Y K W Y F E L G T

CTATATCTATCTATATGAAGAAGAAATCATGTAAGGGAGGGGATTCTTATTTGTACAAATGGTACTTCGAACTTGGAAC
P I S I Y M K K K S C K G G D S Y L Y K W Y F E L G T

CTATATCTATCTATATGAAGAAGAAATCATGTAAGGGAGGGGATTCTTATTTGTACAAATGGTACTTCGAACTTGGAAC
P I S I Y M K K K S C K G G D S Y L Y K W Y F E L G T

CTATATCTATCTATATGAAGAAGAAATCATGTAAGGGAGGGGATTCTTATTTGTACAAATGGTACTTCGAACTTGGAAC
P I S I Y M K K K S C K G G D S Y L Y K W Y F E L G T

CTATATCTATCTATATGAAGAAGAAATCATGTAAGGGAGGGGATTCTTATTTGTACAAATGGTACTTCGAACTTGGAAC
P I S I Y M K K K S C K G G D S Y L Y K W Y F E L G T

CTATATCTATCTATATGAAGAAGAAATCATGTAAGGGAGGGGATTCTTATTTGTACAAATGGTACTTCGAACTTGGAAC
P I S I Y M K K K S C K G G D S Y L Y K W Y F E L G T

CTATATCTATCTATATGAAGAAGAAATCATGTAAGGGAGGGGATTCTTATTTGTACAAATGGTACTTCGAACTTGGAAC
P I S I Y M K K K S C K G G D S Y L Y K W Y F E L G T

CTATATCTATCTATATGAAGAAGAAATCATGTAAGGGAGGGGATTCTTATTTGTACAAATGGTACTTCGAACTTGGAAC
P I S I Y M K K K S C K G G D S Y L Y K W Y F E L G T

CTATATCTATCTATATGAAGAAGAAATCATGTAAGGGAGGGGATTCTTATTTGTACAAATGGTACTTCGAACTTGGAAC
P I S I Y M K K K S C K G G D S Y L Y K W Y F E L G T

CTATATCTATCTATATGAAGAAGAAATCATGTAAGGGAGGGGATTCTTATTTGTACAAATGGTACTTCGAACTTGGAAC
P I S I Y M K K K S C K G G D S Y L Y K W Y F E L G T

CTATATCTATCTATATGAAGAAGAAATCATGTAAGGGAGGGGATTCTTATTTGTACAAATGGTACTTCGAACTTGGAAC
P I S I Y M K K K S C K G G D S Y L Y K W Y F E L G T

CTATATCTATCTATATGAAGAAGAAATCATGTAAGGGGGGGGATTCTTATTTGTACAAATGGTACTTCGAACTTGGAAC
P I S I Y M K K K S C K G G D S Y L Y K W Y F E L G T

CTATATCTATCTATATGAAGAAGAAATCATGTAAGGGAGGGGATTCTTATTTGTACAAATGGTACTTCGAACTTGGAAC
P I S I Y M K K K S C K G G D S Y L Y K W Y F E L G T

CTATATCTATCTATATGAAGAAGAAATCATGTAAGGGAGGGGATTCTTATTTGTACAAATGGTACTTCGAACTTGGAAC
P I S I Y M K K K S C K G G D S Y L Y K W Y F E L G T

CTATATCTATCTATATGAAGAAGAAATCATGTAAGGGAGGGGATTCTTATTTGTACAAATGGTACTTCGAACTTGGAAC
P I S I Y M K K K S C K G G D S Y L Y K W Y F E L G T

CTATATCTATCTATATGAAGAAGAAATCATGTAAGGGAGGGGATTCTTATTTGTACAAATGGTACTTCGAACTTGGAAC
P I S I Y M K K K S C K G G D S Y L Y K W Y F E L G T

CTATATCTATCTATATGAAGAAGAAATCATGTAAGGGAGGGGATTCTTATTTGTACAAATGGTACTTCGAACTTGGAAC
P I S I Y M K K K S C K G G D S Y L Y K W Y F E L G T

CTATATCTATCTATATGAAGAAGAAATCATGTAAGGGAGGGGATTCTTATTTGTACAAATGGTACTTCGAACTTGGAAC
P I S I Y M K K K S C K G G D S Y L Y K W Y F E L G T

CTATATCTATCTATATGAAGAAGAAATCATGTAAGGGAGGGGATTCTTATTTGTACAAATGGTACTTCGAACTTGGAAC
P I S I Y M K K K S C K G G D S Y L Y K W Y F E L G T

CTATATCTATCTATATGAAGAAGAAATCATGTAAGGGAGGGGATTCTTATTTGTACAAATGGTACTTCGAACTTGGAAC
P I S I Y M K K K S C K G G D S Y L Y K W Y F E L G T

CTATATCTATCTATATGAAGAAGAAATCATGTAAGGGAGGGGATTCTTATTTGTACAAATGGTACTTCGAACTTGGAAC
P I S I Y M K K K S C K G G D S Y L Y K W Y F E L G T

CTATATCTATCTATATGAAGAAGAAATCATGTAAGGGAGGGGATTCTTATTTGTACAAATGGTACTTCGAACTTGGAAC
P I S I Y M K K K S C K G G D S Y L Y K W Y F E L G T

CTATATCTATCTATATGAAGAAGAAATCATGTAAGGGAGGGGATTCTTATTTGTACAAATGGTACTTCGAACTTGGAAC
P I S I Y M K K K S C K G G D S Y L Y K W Y F E L G T

5,900 5,910 5,920 5,930 5,940 5,950 5,960 5,970

GAGCATGAAGAAATTAACGATACTTCTTTATCTTTTGAGTTGTTCTGCCGGATCGGTCGCTCAAGATCTTTGGTCTTCATC

GAGCATGAAGAAATTAACGATACTTCTTTATCTTTTGAGTTGTTCTGCCGGATCGGTCGCTCAAGATCTTTGGTCTTCATC
S M K K L T I L L Y L L S C S A G S V A Q D L W S S S

GAGCATGAAGAAATTAACGATACTTCTTTATCTTTTGAGTTGTTCTGCCGGATCGGTCGCTCAAGATCTTTGGTCTTCATC
S M K K L T I L L Y L L S C S A G S V A Q D L W S S S

GAGCATGAAGAAATTAACGATACTTCTTTATCTTTTGAGTTGTTCTGCCGGATCGGTCGCTCAAGATCTTTGGTCTTCATC
S M K K L T I L L Y L L S C S A G S V A Q D L W S S S

GAGCATGAAGAAATTAACGATACTTCTTTATCTTTTGAGTTGTTCTGCCGGATCGGTCGCTCAAGATCTTTGGTCTTCATC
S M K K L T I L L Y L L S C S A G S V A Q D L W S S S

GAGCATGAAGAAATTAACGATACTTCTTTATCTTTTGAGTTGTTCTGCCGGATCGGTCGCTCAAGATCTTTGGTCTTCATC
S M K K L T I L L Y L L S C S A G S V A Q D L W S S S

GAGCATGAAGAAATTAACGATACTTCTTTATCTTTTGAGTTGTTCTGCCGGATCGGTCGCTCAAGATCTTTGGTCTTCATC
S M K K L T I L L Y L L S C S A G S V A Q D L W S S S

GAGCATGAAGAAATTAACGATACTTCTTTATCTTTTGAGTTGTTCTGCCGGATCGGTCGCTCAAGATCTTTGGTCTTCATC
S M K K L T I L L Y L L S C S A G S V A Q D L W S S S

GAGCATGAAGAAATTAACGATACTTCTTTATCTTTTGAGTTGTTCTGCCGGATCGGTCGCTCAAGATCTTTGGTCTTCATC
S M K K L T I L L Y L L S C S A G S V A Q D L W S S S

GAGCATGAAGAAATTAACGATACTTCTTTATCTTTTGAGTTGTTCTGCCGGATCGGTCGCTCAAGATCTTTGGTCTTCATC
S M K K L T I L L Y L L S C S A G S V A Q D L W S S S

GAGCATGAAGAAATTAACGATACTTCTTTATCTTTTGAGTTGTTCTGCCGGATCGGTCGCTCAAGATCTTTGGTCTTCATC
S M K K L T I L L Y L L S C S A G S V A Q D L W S S S

GAGCATGAAGAAATTAACGATACTTCTTTATCTTTTGAGTTGTTCTGCCGGATCGGTCGCTCAAGATCTTTGGTCTTCATC
S M K K L T I L L Y L L S C S A G S V A Q D L W S S S

GAGCATGAAGAAATTAACGATACTTCTTTATCTTTTGAGTTGTTCTGCCGGATCGGTCGCTCAAGATCTTTGGTCTTCATC
S M K K L T I L L Y L L S C S A G S V A Q D L W S S S

GAGCATGAAGAAATTAACGATACTTCTTTATCTTTTGAGTTGTTCTGCCGGATCGGTCGCTCAAGATCTTTGGTCTTCATC
S M K K L T I L L Y L L S C S A G S V A Q D L W S S S

GAGCATGAAGAAATTAACGATACTTCTTTATCTTTTGAGTTGTTCTGCCGGATCGGTCGCTCAAGATCTTTGGTCTTCATC
S M K K L T I L L Y L L S C S A G S V A Q D L W S S S

GAGCATGAAGAAATTAACGATACTTCTTTATCTTTTGAGTTGTTCTGCCGGATCGGTCGCTCAAGATCTTTGGTCTTCATC
S M K K L T I L L Y L L S C S A G S V A Q D L W S S S

GAGCATGAAGAAATTAACGATACTTCTTTATCTTTTGAGTTGTTCTGCCGGATCGGTCGCTCAAGATCTTTGGTCTTCATC
S M K K L T I L L Y L L S C S A G S V A Q D L W S S S

GAGCATGAAGAAATTAACGATACTTCTTTATCTTTTGAGTTGTTCTGCCGGATCGGTCGCTCAAGATCTTTGGTCTTCATC
S M K K L T I L L Y L L S C S A G S V A Q D L W S S S

GAGCATGAAGAAATTAACGATACTTCTTTATCTTTTGAGTTGTTCTGCCGGATCGGTCGCTCAAGATCTTTGGTCTTCATC
S M K K L T I L L Y L L S C S A G S V A Q D L W S S S

GAGCATGAAGAAATTAACGATACTTCTTTATCTTTTGAGTTGTTCTGCCGGATCGGTCGCTCAAGATCTTTGGTCTTCATC
S M K K L T I L L Y L L S C S A G S V A Q D L W S S S

GAGCATGAAGAAATTAACGATACTTCTTTATCTTTTGAGTTGTTCTGCCGGATCGGTCGCTCAAGATCTTTGGTCTTCATC
S M K K L T I L L Y L L S C S A G S V A Q D L W S S S

GAGCATGAAGAAATTAACGATACTTCTTTATCTTTTGAGTTGTTCTGCCGGATCGGTCGCTCAAGATCTTTGGTCTTCATC
S M K K L T I L L Y L L S C S A G S V A Q D L W S S S

GAGCATGAAGAAATTAACGATACTTCTTTATCTTTTGAGTTGTTCTGCCGGATCGGTCGCTCAAGATCTTTGGTCTTCATC
S M K K L T I L L Y L L S C S A G S V A Q D L W S S S

GAGCATGAAGAAATTAACGATACTTCTTTATCTTTTGAGTTGTTCTGCCGGATCGGTCGCTCAAGATCTTTGGTCTTCATC
S M K K L T I L L Y L L S C S A G S V A Q D L W S S S

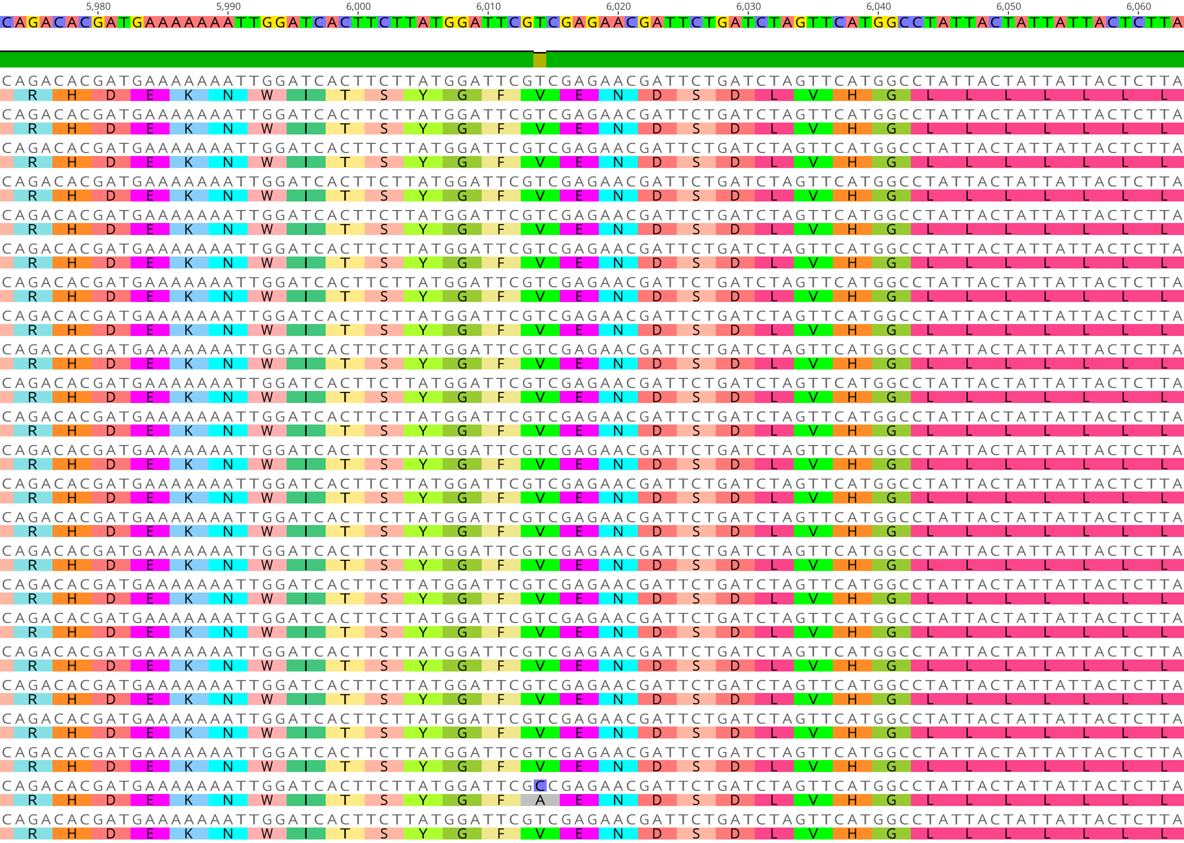
5,980 5,990 6,000 6,010 6,020 6,030 6,040 6,050 6,060
CAGACACGATGAAAAAAATTGGATCACTTCTTATGGATTCGTCGAGAACGATTCTGATCTAGTTCATGGCCTATTACTATTATTACTCTTA
CAGACACGATGAAAAAAATTGGATCACTTCTTATGGATTCGTCGAGAACGATTCTGATCTAGTTCATGGCCTATTACTATTATTACTCTTA
R H D E K N W I T S Y G F V E N D S D L V H G L L L L L L L
CAGACACGATGAAAAAAATTGGATCACTTCTTATGGATTCGTCGAGAACGATTCTGATCTAGTTCATGGCCTATTACTATTATTACTCTTA
R H D E K N W I T S Y G F V E N D S D L V H G L L L L L L L
CAGACACGATGAAAAAAATTGGATCACTTCTTATGGATTCGTCGAGAACGATTCTGATCTAGTTCATGGCCTATTACTATTATTACTCTTA
R H D E K N W I T S Y G F V E N D S D L V H G L L L L L L L
CAGACACGATGAAAAAAATTGGATCACTTCTTATGGATTCGTCGAGAACGATTCTGATCTAGTTCATGGCCTATTACTATTATTACTCTTA
R H D E K N W I T S Y G F V E N D S D L V H G L L L L L L L
CAGACACGATGAAAAAAATTGGATCACTTCTTATGGATTCGTCGAGAACGATTCTGATCTAGTTCATGGCCTATTACTATTATTACTCTTA
R H D E K N W I T S Y G F V E N D S D L V H G L L L L L L L
CAGACACGATGAAAAAAATTGGATCACTTCTTATGGATTCGTCGAGAACGATTCTGATCTAGTTCATGGCCTATTACTATTATTACTCTTA
R H D E K N W I T S Y G F V E N D S D L V H G L L L L L L L
CAGACACGATGAAAAAAATTGGATCACTTCTTATGGATTCGTCGAGAACGATTCTGATCTAGTTCATGGCCTATTACTATTATTACTCTTA
R H D E K N W I T S Y G F V E N D S D L V H G L L L L L L L
CAGACACGATGAAAAAAATTGGATCACTTCTTATGGATTCGTCGAGAACGATTCTGATCTAGTTCATGGCCTATTACTATTATTACTCTTA
R H D E K N W I T S Y G F V E N D S D L V H G L L L L L L L
CAGACACGATGAAAAAAATTGGATCACTTCTTATGGATTCGTCGAGAACGATTCTGATCTAGTTCATGGCCTATTACTATTATTACTCTTA
R H D E K N W I T S Y G F V E N D S D L V H G L L L L L L L
CAGACACGATGAAAAAAATTGGATCACTTCTTATGGATTCGTCGAGAACGATTCTGATCTAGTTCATGGCCTATTACTATTATTACTCTTA
R H D E K N W I T S Y G F V E N D S D L V H G L L L L L L L
CAGACACGATGAAAAAAATTGGATCACTTCTTATGGATTCGTCGAGAACGATTCTGATCTAGTTCATGGCCTATTACTATTATTACTCTTA
R H D E K N W I T S Y G F V E N D S D L V H G L L L L L L L
CAGACACGATGAAAAAAATTGGATCACTTCTTATGGATTCGTCGAGAACGATTCTGATCTAGTTCATGGCCTATTACTATTATTACTCTTA
R H D E K N W I T S Y G F V E N D S D L V H G L L L L L L L
CAGACACGATGAAAAAAATTGGATCACTTCTTATGGATTCGTCGAGAACGATTCTGATCTAGTTCATGGCCTATTACTATTATTACTCTTA
R H D E K N W I T S Y G F V E N D S D L V H G L L L L L L L
CAGACACGATGAAAAAAATTGGATCACTTCTTATGGATTCGTCGAGAACGATTCTGATCTAGTTCATGGCCTATTACTATTATTACTCTTA
R H D E K N W I T S Y G F V E N D S D L V H G L L L L L L L
CAGACACGATGAAAAAAATTGGATCACTTCTTATGGATTCGTCGAGAACGATTCTGATCTAGTTCATGGCCTATTACTATTATTACTCTTA
R H D E K N W I T S Y G F V E N D S D L V H G L L L L L L L
CAGACACGATGAAAAAAATTGGATCACTTCTTATGGATTCGTCGAGAACGATTCTGATCTAGTTCATGGCCTATTACTATTATTACTCTTA
R H D E K N W I T S Y G F V E N D S D L V H G L L L L L L L
CAGACACGATGAAAAAAATTGGATCACTTCTTATGGATTCGTCGAGAACGATTCTGATCTAGTTCATGGCCTATTACTATTATTACTCTTA
R H D E K N W I T S Y G F V E N D S D L V H G L L L L L L L
CAGACACGATGAAAAAAATTGGATCACTTCTTATGGATTCGTCGAGAACGATTCTGATCTAGTTCATGGCCTATTACTATTATTACTCTTA
R H D E K N W I T S Y G F V E N D S D L V H G L L L L L L L
CAGACACGATGAAAAAAATTGGATCACTTCTTATGGATTCGTCGAGAACGATTCTGATCTAGTTCATGGCCTATTACTATTATTACTCTTA
R H D E K N W I T S Y G F V E N D S D L V H G L L L L L L L
CAGACACGATGAAAAAAATTGGATCACTTCTTATGGATTCGTCGAGAACGATTCTGATCTAGTTCATGGCCTATTACTATTATTACTCTTA
R H D E K N W I T S Y G F V E N D S D L V H G L L L L L L L
CAGACACGATGAAAAAAATTGGATCACTTCTTATGGATTCGTCGAGAACGATTCTGATCTAGTTCATGGCCTATTACTATTATTACTCTTA
R H D E K N W I T S Y G F V E N D S D L V H G L L L L L L L
CAGACACGATGAAAAAAATTGGATCACTTCTTATGGATTCGCCGAGAACGATTCTGATCTAGTTCATGGCCTATTACTATTATTACTCTTA
R H D E K N W I T S Y G F A E N D S D L V H G L L L L L L L
CAGACACGATGAAAAAAATTGGATCACTTCTTATGGATTCGTCGAGAACGATTCTGATCTAGTTCATGGCCTATTACTATTATTACTCTTA
R H D E K N W I T S Y G F V E N D S D L V H G L L L L L L L

6,070 6,080 6,090 6,100 6,110 6,120 6,130 6,140

GAAGTAGAAGGCGCTCTGGCTCTGGCGGGATCCTCACGGACAGAAAAATCTTGCAGTCAGTTTGATAATAATCGAGTGA

GAAGTAGAAGGCGCTCTGGCTCTGGCGGGATCCTCACGGACAGAAAAATCTTGCAGTCAGTTTGATAATAATCGAGTGA
E V E G A L A L A G S S R T E K S C S Q F D N N R V

GAAGTAGAAGGCGCTCTGGCTCTGGCGGGATCCTCACGGACAGAAAAATCTTGCAGTCAGTTTGATAATAATCGAGTGA
E V E G A L A L A G S S R T E K S C S Q F D N N R V

GAAGTAGAAGGCGCTCTGGCTCTGGCGGGATCCTCACGGACAGAAAAATCTTGCAGTCAGTTTGATAATAATCGAGTGA
E V E G A L A L A G S S R T E K S C S Q F D N N R V

GAAGTAGAAGGCGCTCTGGCTCTGGCGGGATCCTCACGGACAGAAAAATCTTGCAGTCAGTTTGATAATAATCGAGTGA
E V E G A L A L A G S S R T E K S C S Q F D N N R V

GAAGTAGAAGGCGCTCTGGCTCTGGCGGGATCCTCACGGACAGAAAAATCTTGCAGTCAGTTTGATAATAATCGAGTGA
E V E G A L A L A G S S R T E K S C S Q F D N N R V

GAAGTAGAAGGCGCTCTGGCTCTGGCGGGATCCTCACGGACAGAAAAATCTTGCAGTCAGTTTGATAATAATCGAGTGA
E V E G A L A L A G S S R T E K S C S Q F D N N R V

GAAGTAGAAGGCGCTCTGGCTCTGGCGGGATCCTCACGGACAGAAAAATCTTGCAGTCAGTTTGATAATAATCGAGTGA
E V E G A L A L A G S S R T E K S C S Q F D N N R V

GAAGTAGAAGGCGCTCTGGCTCTGGCGGGATCCTCACGGACAGAAAAATCTTGCAGTCAGTTTGATAATAATCGAGTGA
E V E G A L A L A G S S R T E K S C S Q F D N N R V

GAAGTAGAAGGCGCTCTGGCTCTGGCGGGATCCTCACGGACAGAAAAATCTTGCAGTCAGTTTGATAATAATCGAGTGA
E V E G A L A L A G S S R T E K S C S Q F D N N R V

GAAGTAGAAGGCGCTCTGGCTCTGGCGGGATCCTCACGGACAGAAAAATCTTGCAGTCAGTTTGATAATAATCGAGTGA
E V E G A L A L A G S S R T E K S C S Q F D N N R V

GAAGTAGAAGGCGCTCTGGCTCTGGCGGGATCCTCACGGACAGAAAAATCTTGCAGTCAGTTTGATAATAATCGAGTGA
E V E G A L A L A G S S R T E K S C S Q F D N N R V

GAAGTAGAAGGCGCTCTGGCTCTGGCGGGATCCTCACGGACAGAAAAATCTTGCAGTCAGTTTGATAATAATCGAGTGA
E V E G A L A L A G S S R T E K S C S Q F D N N R V

GAAGTAGAAGGCGCTCTGGCTCTGGCGGGATCCTCACGGACAGAAAAATCTTGCAGTCAGTTTGATAATAATCGAGTGA
E V E G A L A L A G S S R T E K S C S Q F D N N R V

GAAGTAGAAGGCGCTCTGGCTCTGGCGGGATCCTCACGGACAGAAAAATCTTGCAGTCAGTTTGATAATAATCGAGTGA
E V E G A L A L A G S S R T E K S C S Q F D N N R V

GAAGTAGAAGGCGCTCTGGCTCTGGCGGGATCCTCACGGACAGAAAAATCTTGCAGTCAGTTTGATAATAATCGAGTGA
E V E G A L A L A G S S R T E K S C S Q F D N N R V

GAAGTAGAAGGCGCTCTGGCTCTGGCGGGATCCTCACGGACAGAAAAATCTTGCAGTCAGTTTGATAATAATCGAGTGA
E V E G A L A L A G S S R T E K S C S Q F D N N R V

GAAGTAGAAGGCGCTCTGGCTCTGGCGGGATCCTCACGGACAGAAAAATCTTGCAGTCAGTTTGATAATAATCGAGTGA
E V E G A L A L A G S S R T E K S C S Q F D N N R V

GAAGTAGAAGGCGCTCTGGCTCTGGCGGGATCCTCACGGACAGAAAAATCTTGCAGTCAGTTTGATAATAATCGAGTGA
E V E G A L A L A G S S R T E K S C S Q F D N N R V

GAAGTAGAAGGCGCTCTGGCTCTGGCGGGATCCTCACGGACAGAAAAATCTTGCAGTCAGTTTGATAATAATCGAGTGA
E V E G A L A L A G S S R T E K S C S Q F D N N R V

GAAGTAGAAGGCGCTCTGGCTCTGGCGGGATCCTCACGGACAGAAAAATCTTGCAGTCAGTTTGATAATAATCGAGTGA
E V E G A L A L A G S S R T E K S C S Q F D N N R V

GAAGTAGAAGGCGCTCTGGCTCTGGCGGGATCCTCACGGACAGAAAAATCTTGCAGTCAGTTTGATAATAATCGAGTGA
E V E G A L A L A G S S R T E K S C S Q F D N N R V

GAAGTAGAAGGCGCTCTGGCTCTGGCGGGATCCTCACGGACAGAAAAATCTTGCAGTCAGTTTGATAATAATCGAGTGA
E V E G A L A L A G S S R T E K S C S Q F D N N R V

GAAGTAGAAGGCGCTCTGGCTCTGGCGGGATCCTCACGGACAGAAAAATCTTGCAGTCAGTTTGATAATAATCGAGTGA
E V E G A L A L A G S S R T E K S C S Q F D N N R V

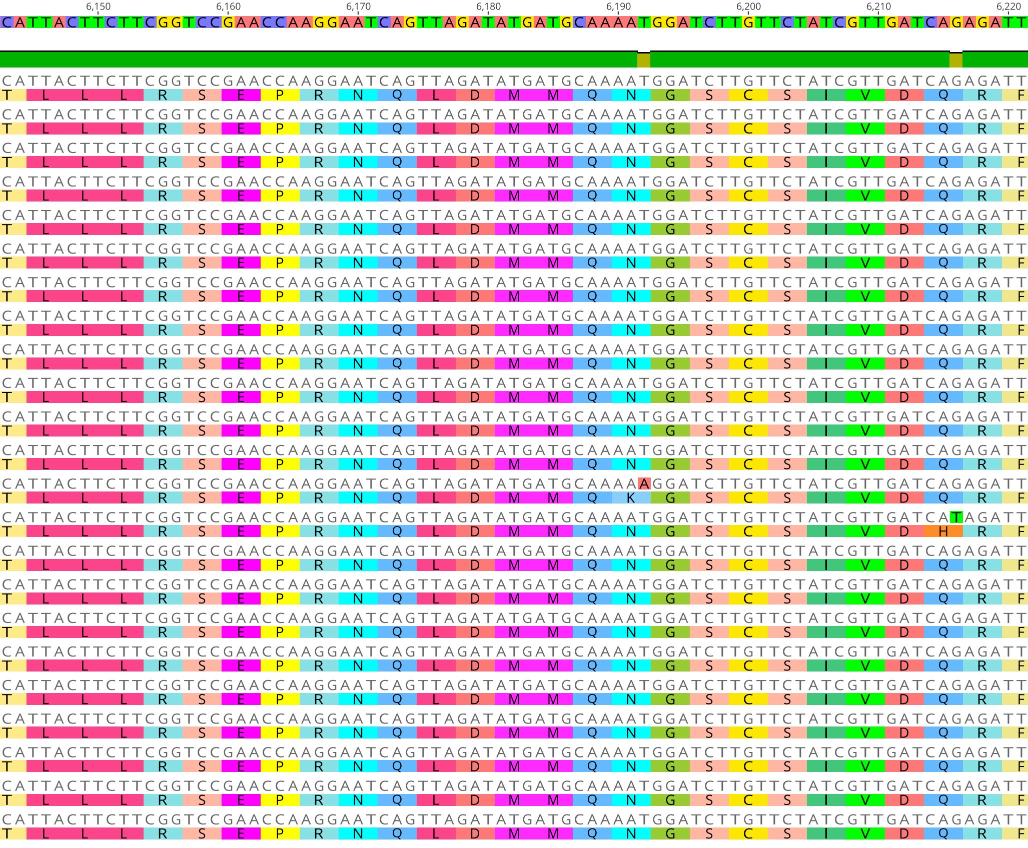

6,150 6,160 6,170 6,180 6,190 6,200 6,210 6,220
CATTACTTCTTCGGTCCGAACCAAGGAATCAGTTAGATATGATGCAAAATGGATCTTGTTCTATCGTTGATCAGAGATT
CATTACTTCTTCGGTCCGAACCAAGGAATCAGTTAGATATGATGCAAAATGGATCTTGTTCTATCGTTGATCAGAGATT
T L L L R S E P R N Q L D M M Q N G S C S I V D Q R F
CATTACTTCTTCGGTCCGAACCAAGGAATCAGTTAGATATGATGCAAAATGGATCTTGTTCTATCGTTGATCAGAGATT
T L L L R S E P R N Q L D M M Q N G S C S I V D Q R F
CATTACTTCTTCGGTCCGAACCAAGGAATCAGTTAGATATGATGCAAAATGGATCTTGTTCTATCGTTGATCAGAGATT
T L L L R S E P R N Q L D M M Q N G S C S I V D Q R F
CATTACTTCTTCGGTCCGAACCAAGGAATCAGTTAGATATGATGCAAAATGGATCTTGTTCTATCGTTGATCAGAGATT
T L L L R S E P R N Q L D M M Q N G S C S I V D Q R F
CATTACTTCTTCGGTCCGAACCAAGGAATCAGTTAGATATGATGCAAAATGGATCTTGTTCTATCGTTGATCAGAGATT
T L L L R S E P R N Q L D M M Q N G S C S I V D Q R F
CATTACTTCTTCGGTCCGAACCAAGGAATCAGTTAGATATGATGCAAAATGGATCTTGTTCTATCGTTGATCAGAGATT
T L L L R S E P R N Q L D M M Q N G S C S I V D Q R F
CATTACTTCTTCGGTCCGAACCAAGGAATCAGTTAGATATGATGCAAAATGGATCTTGTTCTATCGTTGATCAGAGATT
T L L L R S E P R N Q L D M M Q N G S C S I V D Q R F
CATTACTTCTTCGGTCCGAACCAAGGAATCAGTTAGATATGATGCAAAATGGATCTTGTTCTATCGTTGATCAGAGATT
T L L L R S E P R N Q L D M M Q N G S C S I V D Q R F
CATTACTTCTTCGGTCCGAACCAAGGAATCAGTTAGATATGATGCAAAATGGATCTTGTTCTATCGTTGATCAGAGATT
T L L L R S E P R N Q L D M M Q N G S C S I V D Q R F
CATTACTTCTTCGGTCCGAACCAAGGAATCAGTTAGATATGATGCAAAATGGATCTTGTTCTATCGTTGATCAGAGATT
T L L L R S E P R N Q L D M M Q N G S C S I V D Q R F
CATTACTTCTTCGGTCCGAACCAAGGAATCAGTTAGATATGATGCAAAATGGATCTTGTTCTATCGTTGATCAGAGATT
T L L L R S E P R N Q L D M M Q N G S C S I V D Q R F
CATTACTTCTTCGGTCCGAACCAAGGAATCAGTTAGATATGATGCAAAATGGATCTTGTTCTATCGTTGATCAGAGATT
T L L L R S E P R N Q L D M M Q N G S C S I V D Q R F
CATTACTTCTTCGGTCCGAACCAAGGAATCAGTTAGATATGATGCAAAAAGGATCTTGTTCTATCGTTGATCAGAGATT
T L L L R S E P R N Q L D M M Q K G S C S I V D Q R F
CATTACTTCTTCGGTCCGAACCAAGGAATCAGTTAGATATGATGCAAAATGGATCTTGTTCTATCGTTGATCATAGATT
T L L L R S E P R N Q L D M M Q N G S C S I V D H R F
CATTACTTCTTCGGTCCGAACCAAGGAATCAGTTAGATATGATGCAAAATGGATCTTGTTCTATCGTTGATCAGAGATT
T L L L R S E P R N Q L D M M Q N G S C S I V D Q R F
CATTACTTCTTCGGTCCGAACCAAGGAATCAGTTAGATATGATGCAAAATGGATCTTGTTCTATCGTTGATCAGAGATT
T L L L R S E P R N Q L D M M Q N G S C S I V D Q R F
CATTACTTCTTCGGTCCGAACCAAGGAATCAGTTAGATATGATGCAAAATGGATCTTGTTCTATCGTTGATCAGAGATT
T L L L R S E P R N Q L D M M Q N G S C S I V D Q R F
CATTACTTCTTCGGTCCGAACCAAGGAATCAGTTAGATATGATGCAAAATGGATCTTGTTCTATCGTTGATCAGAGATT
T L L L R S E P R N Q L D M M Q N G S C S I V D Q R F
CATTACTTCTTCGGTCCGAACCAAGGAATCAGTTAGATATGATGCAAAATGGATCTTGTTCTATCGTTGATCAGAGATT
T L L L R S E P R N Q L D M M Q N G S C S I V D Q R F
CATTACTTCTTCGGTCCGAACCAAGGAATCAGTTAGATATGATGCAAAATGGATCTTGTTCTATCGTTGATCAGAGATT
T L L L R S E P R N Q L D M M Q N G S C S I V D Q R F
CATTACTTCTTCGGTCCGAACCAAGGAATCAGTTAGATATGATGCAAAATGGATCTTGTTCTATCGTTGATCAGAGATT
T L L L R S E P R N Q L D M M Q N G S C S I V D Q R F
CATTACTTCTTCGGTCCGAACCAAGGAATCAGTTAGATATGATGCAAAATGGATCTTGTTCTATCGTTGATCAGAGATT
T L L L R S E P R N Q L D M M Q N G S C S I V D Q R F
CATTACTTCTTCGGTCCGAACCAAGGAATCAGTTAGATATGATGCAAAATGGATCTTGTTCTATCGTTGATCAGAGATT
T L L L R S E P R N Q L D M M Q N G S C S I V D Q R F

```
6,230 6,240 6,250 6,260 6,270 6,280 6,290 6
TCTATATGAAAAATACGAATCGGAGTTTGAAGAAGGGGAAGGGGCCCTCGATCCGCAACAGATAGAGGAGGATTTATT

TCTATATGAAAAATACGAATCGGAGTTTGAAGAAGGGGAAGGGGCCCTCGATCCGCAACAGATAGAGGAGGATTTATT
 L Y E K Y E S E F E E G E G A L D P Q Q I E E D L F
TCTATATGAAAAATACGAATCGGAGTTTGAAGAAGGGGAAGGGGCCCTCGATCCGCAACAGATAGAGGAGGATTTATT
 L Y E K Y E S E F E E G E G A L D P Q Q I E E D L F
TCTATATGAAAAATACGAATCGGAGTTTGAAGAAGGGGAAGGGGCCCTCGATCCGCAACAGATAGAGGAGGATTTATT
 L Y E K Y E S E F E E G E G A L D P Q Q I E E D L F
TCTATATGAAAAATACGAATCGGAGTTTGAAGAAGGGGAAGGGGCCCTCGATCCGCAACAGATAGAGGAGGATTTATT
 L Y E K Y E S E F E E G E G A L D P Q Q I E E D L F
TCTATATGAAAAATACGAATCGGAGTTTGAAGAAGGGGAAGGGGCCCTCGATCCGCAACAGATAGAGGAGGATTTATT
 L Y E K Y E S E F E E G E G A L D P Q Q I E E D L F
TCTATATGAAAAATACGAATCGGAGTTTGAAGAAGGGGAAGGGGCCCTCGATCCGCAACAGATAGAGGAGGATTTATT
 L Y E K Y E S E F E E G E G A L D P Q Q I E E D L F
TCTATATGAAAAATACGAATCGGAGTTTGAAGAAGGGGAAGGGGCCCTCGATCCGCAACAGATAGAGGAGGATTTATT
 L Y E K Y E S E F E E G E G A L D P Q Q I E E D L F
TCTATATGAAAAATACGAATCGGAGTTTGAAGAAGGGGAAGGGGCCCTCGATCCGCAACAGATAGAGGAGGATTTATT
 L Y E K Y E S E F E E G E G A L D P Q Q I E E D L F
TCTATATGAAAAATACGAATCGGAGTTTGAAGAAGGGGAAGGGGCCCTCGATCCGCAACAGATAGAGGAGGATTTATT
 L Y E K Y E S E F E E G E G A L D P Q Q I E E D L F
TCTATATGAAAAATACGAATCGGAGTTTGAAGAAGGGGAAGGGGCCCTCGATCCGCAACAGATAGAGGAGGATTTATT
 L Y E K Y E S E F E E G E G A L D P Q Q I E E D L F
TCTATATGAAAAATACGAATCGGAGTTTGAAGAAGGGGAAGGGGCCCTCGATCCGCAACAGATAGAGGAGGATTTATT
 L Y E K Y E S E F E E G E G A L D P Q Q I E E D L F
TCTATATGAAAAATACGAATCGGAGTTTGAAGAAGGGGAAGGGGCCCTCGATCCGCAACAGATAGAGGAGGATTTATT
 L Y E K Y E S E F E E G E G A L D P Q Q I E E D L F
TCTATATGAAAAATACGAATCGGAGTTTGAAGAAGGGGAAGGGGCCCTCGATCCGCAACAGATAGAGGAGGATTTATT
 L Y E K Y E S E F E E G E G A L D P Q Q I E E D L F
TCTATATGAAAAATACGAATCGGAGTTTGAAGAAGGGGAAGGGGGCCTCGATCCGCAACAGATAGAGGAGGATTTATT
 L Y E K Y E S E F E E G E G G L D P Q Q I E E D L F
TCTATATGAAAAATACGAATCGGAGTTTGAAGAAGGGGAAGGGGGCCTCGATCCGCAACAGATAGAGGAGGATTTATT
 L Y E K Y E S E F E E G E G G L D P Q Q I E E D L F
TCTATATGAAAAATACGAATCGGAGTTTGAAGAAGGGGAAGGGGGCCTCGATCCGCAACAGATAGAGGAGGATTTATT
 L Y E K Y E S E F E E G E G G L D P Q Q I E E D L F
TCTATATGAAAAATACGAATCGGAGTTTGAAGAAGGGGAAGGGGCCCTCGATCCGCAACAGATAGAGGAGGATTTATT
 L Y E K Y E S E F E E G E G A L D P Q Q I E E D L F
TCTATATGAAAAATACGAATCGGAGTTTGAAGAAGGGGAAGGGGCCCTCGATCCGCAACAGATAGAGGAGGATTTATT
 L Y E K Y E S E F E E G E G A L D P Q Q I E E D L F
TCTATATGAAAAATACGAATCGGAGTTTGAAGAAGGGGAAGGGGCCCTCGATCCGCAACAGATAGAGGAGGATTTCTT
 L Y E K Y E S E F E E G E G A L D P Q Q I E E D F F
TCTATATGAAAAATACGAATCGGAGTTTGAAGAAGGGGAAGGGGCCCTCGATCCGCAACAGATAGAGGAGGATTTCTT
 L Y E K Y E S E F E E G E G A L D P Q Q I E E D F F
TCTATATGAAAAATACGAATCGGAGTTTGAAGAAGGGGAAGGGGCCCTCGATCCGCAACAGATAGAGGAGGATTTCTT
 L Y E K Y E S E F E E G E G A L D P Q Q I E E D F F
TCTATATGAAAAATACGAATCGGAGTTTGAAGAAGGGGAAGGGGCCCTCGATCCGCAACAGATAGAGGAGGATTTCTT
 L Y E K Y E S E F E E G E G A L D P Q Q I E E D F F
TCTATATGAAAAATACGAATCGGAGTTTGAAGAAGGGGAAGGGGCCCTCGATCCGCAACAGATAGAGGAGGATTTATT
 L Y E K Y E S E F E E G E G A L D P Q Q I E E D L F
```

6,300 6,310 6,320 6,330 6,340 6,350 6,360 6,370 6,380

CAATCACATAGTTTGGGCTCCTAGAATATGGCGATTTGATTGTATCGAAAGGCCCACTGAATTGGGATTTCCCTATTGGACTGGGTCAT

CAATCACATAGTTTGGGCTCCTAGAATATGGCGATTTGATTGTATCGAAAGGCCCACTGAATTGGGATTTCCCTATTGGACTGGGTCAT
N H I V W A P R I W R F D C I E R P T E L G F P Y W T G S

CAATCACATAGTTTGGGCTCCTAGAATATGGCGATTTGATTGTATCGAAAGGCCCACTGAATTGGGATTTCCCTATTGGACTGGGTCAT
N H I V W A P R I W R F D C I E R P T E L G F P Y W T G S

CAATCACATAGTTTGGGCTCCTAGAATATGGCGATTTGATTGTATCGAAAGGCCCACTGAATTGGGATTTCCCTATTGGACTGGGTCAT
N H I V W A P R I W R F D C I E R P T E L G F P Y W T G S

CAATCACATAGTTTGGGCTCCTAGAATATGGCGATTTGATTGTATCGAAAGGCCCACTGAATTGGGATTTCCCTATTGGACTGGGTCAT
N H I V W A P R I W R F D C I E R P T E L G F P Y W T G S

CAATCACATAGTTTGGGCTCCTAGAATATGGCGATTTGATTGTATCGAAAGGCCCACTGAATTGGGATTTCCCTATTGGACTGGGTCAT
N H I V W A P R I W R F D C I E R P T E L G F P Y W T G S

CAATCACATAGTTTGGGCTCCTAGAATATGGCGATTTGATTGTATCGAAAGGCCCACTGAATTGGGATTTCCCTATTGGACTGGGTCAT
N H I V W A P R I W R F D C I E R P T E L G F P Y W T G S

CAATCACATAGTTTGGGCTCCTAGAATATGGCGATTTGATTGTATCGAAAGGCCCACTGAATTGGGATTTCCCTATTGGACTGGGTCAT
N H I V W A P R I W R F D C I E R P T E L G F P Y W T G S

CAATCACATAGTTTGGGCTCCTAGAATATGGCGATTTGATTGTATCGAAAGGCCCACTGAATTGGGATTTCCCTATTGGACTGGGTCAT
N H I V W A P R I W R F D C I E R P T E L G F P Y W T G S

CAATCACATAGTTTGGGCTCCTAGAATATGGCGATTTGATTGTATCGAAAGGCCCACTGAATTGGGATTTCCCTATTGGACTGGGTCAT
N H I V W A P R I W R F D C I E R P T E L G F P Y W T G S

CAATCACATAGTTTGGGCTCCTAGAATATGGCGATTTGATTGTATCGAAAGGCCCACTGAATTGGGATTTCCCTATTGGACTGGGTCAT
N H I V W A P R I W R F D C I E R P T E L G F P Y W T G S

CAATCACATAGTTTGGGCTCCTAGAATATGGCGATTTGATTGTATCGAAAGGCCCACTGAATTGGGATTTCCCTATTGGACTGGGTCAT
N H I V W A P R I W R F D C I E R P T E L G F P Y W T G S

CAATCACATAGTTTGGGCTCCTAGAATATGGCGATTTGATTGTATCGAAAGGCCCACTGAATTGGGATTTCCCTATTGGACTGGGTCAT
N H I V W A P R I W R F D C I E R P T E L G F P Y W T G S

CAATCACATAGTTTGGGCTCCTAGAATATGGCGATTTGATTGTATCGAAAGGCCCACTGAATTGGGATTTCCCTATTGGACTGGGTCAT
N H I V W A P R I W R F D C I E R P T E L G F P Y W T G S

CAATCACATAGTTTGGGCTCCTAGAATATGGCGATTCGATTGTATCGAAAGGTCCACTGAATTGGGATTTCCCTATTGGACTGGGTCAT
N H I V W A P R I W R F D C I E R S T E L G F P Y W T G S

CAATCACATAGTTTGGGCTCCTAGAATATGGCGATTTGATTGTATCGAAAGGCCCACTGAATTGGGATTTCCCTATTGGACTGGGTCAT
N H I V W A P R I W R F D C I E R P T E L G F P Y W T G S

CAATCACATAGTTTGGGCTCCTAGAATATGGCGATTTGATTGTATCGAAAGGCCCACTGAATTGGGATTTCCCTATTGGACTGGGTCAT
N H I V W A P R I W R F D C I E R P T E L G F P Y W T G S

CAATCACATAGTTTGGGCTCCTAGAATATGGCGATTTGATTGTATCGAAAGGCCCACTGAATTGGGATTTCCCTATTGGACTGGGTCAT
N H I V W A P R I W R F D C I E R P T E L G F P Y W T G S

CAATCACATAGTTTGGGCTCCTAGAATATGGCGATTTGATTGTATCGAAAGGCCCACTGAATTGGGATTTCCCTATTGGACTGGGTCAT
N H I V W A P R I W R F D C I E R P T E L G F P Y W T G S

CAATCACATAGTTTGGGCTCCTAGAATATGGCGATTTGATTGTATCGAAAGGCCCACTGAATTGGGATTTCCCTATTGGACTGGGTCAT
N H I V W A P R I W R F D C I E R P T E L G F P Y W T G S

CAATCACATAGTTTGGGCTCCTAGAATATGGCGATTTGATTGTATCGAAAGGCCCACTGAATTGGGATTTCCCTATTGGACTGGGTCAT
N H I V W A P R I W R F D C I E R P T E L G F P Y W T G S

CAATCACATAGTTTGGGCTCCTAGAATATGGCGATTTGATTGTATCGAAAGGCCCACTGAATTGGGATTTCCCTATTGGACTGGGTCAT
N H I V W A P R I W R F D C I E R P T E L G F P Y W T G S

CAATCACATAGTTTGGGCTCCTAGAATATGGCGATTTGATTGTATCGAAAGGCCCACTGAATTGGGATTTCCCTATTGGACTGGGTCAT
N H I V W A P R I W R F D C I E R P T E L G F P Y W T G S

CAATCACATAGTTTGGGCTCCTAGAATATGGCGATTTGATTGTATCGAAAGGCCCACTGAATTGGGATTTCCCTATTGGACTGGGTCAT
N H I V W A P R I W R F D C I E R P T E L G F P Y W T G S

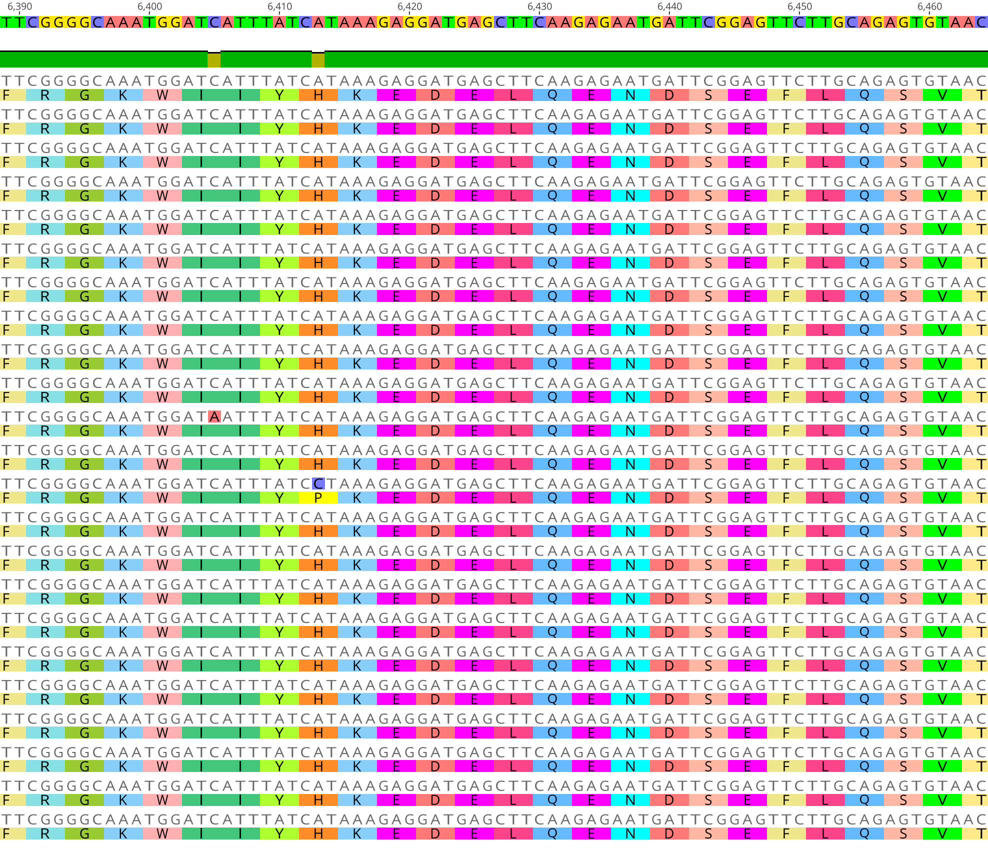
6,390 6,400 6,410 6,420 6,430 6,440 6,450 6,460
TTCGGGGCAAATGGATCATTTATCATAAAGAGGATGAGCTTCAAGAGAATGATTCGGAGTTCTTGCAGAGTGTAAC
TTCGGGGCAAATGGATCATTTATCATAAAGAGGATGAGCTTCAAGAGAATGATTCGGAGTTCTTGCAGAGTGTAAC
F R G K W I I Y H K E D E L Q E N D S E F L Q S V T
TTCGGGGCAAATGGATCATTTATCATAAAGAGGATGAGCTTCAAGAGAATGATTCGGAGTTCTTGCAGAGTGTAAC
F R G K W I I Y H K E D E L Q E N D S E F L Q S V T
TTCGGGGCAAATGGATCATTTATCATAAAGAGGATGAGCTTCAAGAGAATGATTCGGAGTTCTTGCAGAGTGTAAC
F R G K W I I Y H K E D E L Q E N D S E F L Q S V T
TTCGGGGCAAATGGATCATTTATCATAAAGAGGATGAGCTTCAAGAGAATGATTCGGAGTTCTTGCAGAGTGTAAC
F R G K W I I Y H K E D E L Q E N D S E F L Q S V T
TTCGGGGCAAATGGATCATTTATCATAAAGAGGATGAGCTTCAAGAGAATGATTCGGAGTTCTTGCAGAGTGTAAC
F R G K W I I Y H K E D E L Q E N D S E F L Q S V T
TTCGGGGCAAATGGATCATTTATCATAAAGAGGATGAGCTTCAAGAGAATGATTCGGAGTTCTTGCAGAGTGTAAC
F R G K W I I Y H K E D E L Q E N D S E F L Q S V T
TTCGGGGCAAATGGATCATTTATCATAAAGAGGATGAGCTTCAAGAGAATGATTCGGAGTTCTTGCAGAGTGTAAC
F R G K W I I Y H K E D E L Q E N D S E F L Q S V T
TTCGGGGCAAATGGATCATTTATCATAAAGAGGATGAGCTTCAAGAGAATGATTCGGAGTTCTTGCAGAGTGTAAC
F R G K W I I Y H K E D E L Q E N D S E F L Q S V T
TTCGGGGCAAATGGATCATTTATCATAAAGAGGATGAGCTTCAAGAGAATGATTCGGAGTTCTTGCAGAGTGTAAC
F R G K W I I Y H K E D E L Q E N D S E F L Q S V T
TTCGGGGCAAATGGATCATTTATCATAAAGAGGATGAGCTTCAAGAGAATGATTCGGAGTTCTTGCAGAGTGTAAC
F R G K W I I Y H K E D E L Q E N D S E F L Q S V T
TTCGGGGCAAATGGATAATTTATCATAAAGAGGATGAGCTTCAAGAGAATGATTCGGAGTTCTTGCAGAGTGTAAC
F R G K W I I Y H K E D E L Q E N D S E F L Q S V T
TTCGGGGCAAATGGATCATTTATCATAAAGAGGATGAGCTTCAAGAGAATGATTCGGAGTTCTTGCAGAGTGTAAC
F R G K W I I Y H K E D E L Q E N D S E F L Q S V T
TTCGGGGCAAATGGATCATTTATCCTAAAGAGGATGAGCTTCAAGAGAATGATTCGGAGTTCTTGCAGAGTGTAAC
F R G K W I I Y P K E D E L Q E N D S E F L Q S V T
TTCGGGGCAAATGGATCATTTATCATAAAGAGGATGAGCTTCAAGAGAATGATTCGGAGTTCTTGCAGAGTGTAAC
F R G K W I I Y H K E D E L Q E N D S E F L Q S V T
TTCGGGGCAAATGGATCATTTATCATAAAGAGGATGAGCTTCAAGAGAATGATTCGGAGTTCTTGCAGAGTGTAAC
F R G K W I I Y H K E D E L Q E N D S E F L Q S V T
TTCGGGGCAAATGGATCATTTATCATAAAGAGGATGAGCTTCAAGAGAATGATTCGGAGTTCTTGCAGAGTGTAAC
F R G K W I I Y H K E D E L Q E N D S E F L Q S V T
TTCGGGGCAAATGGATCATTTATCATAAAGAGGATGAGCTTCAAGAGAATGATTCGGAGTTCTTGCAGAGTGTAAC
F R G K W I I Y H K E D E L Q E N D S E F L Q S V T
TTCGGGGCAAATGGATCATTTATCATAAAGAGGATGAGCTTCAAGAGAATGATTCGGAGTTCTTGCAGAGTGTAAC
F R G K W I I Y H K E D E L Q E N D S E F L Q S V T
TTCGGGGCAAATGGATCATTTATCATAAAGAGGATGAGCTTCAAGAGAATGATTCGGAGTTCTTGCAGAGTGTAAC
F R G K W I I Y H K E D E L Q E N D S E F L Q S V T
TTCGGGGCAAATGGATCATTTATCATAAAGAGGATGAGCTTCAAGAGAATGATTCGGAGTTCTTGCAGAGTGTAAC
F R G K W I I Y H K E D E L Q E N D S E F L Q S V T
TTCGGGGCAAATGGATCATTTATCATAAAGAGGATGAGCTTCAAGAGAATGATTCGGAGTTCTTGCAGAGTGTAAC
F R G K W I I Y H K E D E L Q E N D S E F L Q S V T
TTCGGGGCAAATGGATCATTTATCATAAAGAGGATGAGCTTCAAGAGAATGATTCGGAGTTCTTGCAGAGTGTAAC
F R G K W I I Y H K E D E L Q E N D S E F L Q S V T
TTCGGGGCAAATGGATCATTTATCATAAAGAGGATGAGCTTCAAGAGAATGATTCGGAGTTCTTGCAGAGTGTAAC
F R G K W I I Y H K E D E L Q E N D S E F L Q S V T

6,470 6,480 6,490 6,500 6,510 6,520 6,530 6,540

CATGCAGTACCAGACACGAGATAGATCTTCCAAAGAACAAGGCTTTTTTCGAACAAGCCAATTCATTTGGGACCCTGCGGATC

CATGCAGTACCAGACACGAGATAGATCTTCCAAAGAACAAGGCTTTTTTCGAACAAGCCAATTCATTTGGGACCCTGCGGATC
M Q Y Q T R D R S S K E Q G F F R T S Q F I W D P A D

CATGCAGTACCAGACACGAGATAGATCTTCCAAAGAACAAGGCTTTTTTCGAACAAGCCAATTCATTTGGGACCCTGCGGATC
M Q Y Q T R D R S S K E Q G F F R T S Q F I W D P A D

CATGCAGTACCAGACACGAGATAGATCTTCCAAAGAACAAGGCTTTTTTCGAACAAGCCAATTCATTTGGGACCCTGCGGATC
M Q Y Q T R D R S S K E Q G F F R T S Q F I W D P A D

CATGCAGTACCAGACACGAGATAGATCTTCCAAAGAACAAGGCTTTTTTCGAACAAGCCAATTCATTTGGGACCCTGCGGATC
M Q Y Q T R D R S S K E Q G F F R T S Q F I W D P A D

CATGCAGTACCAGACACGAGATAGATCTTCCAAAGAACAAGGCTTTTTTCGAACAAGCCAATTCATTTGGGACCCTGCGGATC
M Q Y Q T R D R S S K E Q G F F R T S Q F I W D P A D

CATGCAGTACCAGACACGAGATAGATCTTCCAAAGAACAAGGCTTTTTTCGAACAAGCCAATTCATTTGGGACCCTGCGGATC
M Q Y Q T R D R S S K E Q G F F R T S Q F I W D P A D

CATGCAGTACCAGACACGAGATAGATCTTCCAAAGAACAAGGCTTTTTTCGAACAAGCCAATTCATTTGGGACCCTGCGGATC
M Q Y Q T R D R S S K E Q G F F R T S Q F I W D P A D

CATGCAGTACCAGACACGAGATAGATCTTCCAAAGAACAAGGCTTTTTTCGAACAAGCCAATTCATTTGGGACCCTGCGGATC
M Q Y Q T R D R S S K E Q G F F R T S Q F I W D P A D

CATGCAGTACCAGACACGAGATAGATCTTCCAAAGAACAAGGCTTTTTTCGAACAAGCCAATTCATTTGGGACCCTGCGGATC
M Q Y Q T R D R S S K E Q G F F R T S Q F I W D P A D

CATGCAGTACCAGACACGAGATAGATCTTCCAAAGAACAAGGCTTTTTTCGAACAAGCCAATTCATTTGGGACCCTGCGGATC
M Q Y Q T R D R S S K E Q G F F R T S Q F I W D P A D

CATGCAGTACCAGACACGAGATAGATCTTCCAAAGAACAAGGCTTTTTTCGAACAAGCCAATTCATTTGGGACCCTGCGGATC
M Q Y Q T R D R S S K E Q G F F R T S Q F I W D P A D

CATGCAGTACCAGACACGAGATAGATCTTCCAAAGAACAAGGCTTTTTTCGAACAAGCCAATTCATTTGGGACCCTGCGGATC
M Q Y Q T R D R S S K E Q G F F R T S Q F I W D P A D

CATGCAGTACCAGACACGAGATAGATCTTCCAAAGAACAAGGCTTTTTTCGAACAAGCCAATTCATTTGGGACCCTGCGGATC
M Q Y Q T R D R S S K E Q G F F R T S Q F I W D P A D

CATGCAGTACCAGACACGAGATAGATCTTCCAAAGAACAAGGCTTTTTTCGAACAAGCCAATTCATTTGGGACCCTGCGGATC
M Q Y Q T R D R S S K E Q G F F R T S Q F I W D P A D

CATGCAGTACCAGACACGAGATAGATCTTCCAAAGAACAAGGCTTTTTTCGAACAAGCCAATTCATTTGGGACCCTGCGGATC
M Q Y Q T R D R S S K E Q G F F R T S Q F I W D P A D

CATGCAGTACCAGACACGAGATAGATCTTCCAAAGAACAAGGCTTTTTTCGAACAAGCCAATTCATTTGGGACCCTGCGGATC
M Q Y Q T R D R S S K E Q G F F R T S Q F I W D P A D

CATGCAGTACCAGACACGAGATAGATCTTCCAAAGAACAAGGCTTTTTTCGAACAAGCCAATTCATTTGGGACCCTGCGGATC
M Q Y Q T R D R S S K E Q G F F R T S Q F I W D P A D

CATGCAGTACCAGACACGAGATAGATCTTCCAAAGAACAAGGCTTTTTTCGAACAAGCCAATTCATTTGGGACCCTGCGGATC
M Q Y Q T R D R S S K E Q G F F R T S Q F I W D P A D

CATGCAGTACCAGACACGAGATAGATCTTCCAAAGAACAAGGCTTTTTTCGAATAAGCCAATTCATTTGGGACCCTGCGGATC
M Q Y Q T R D R S S K E Q G F F R I S Q F I W D P A D

CATGCAGTACCAGACACGAGATAGATCTTCCAAAGAACAAGGCTTTTTTCGAATAAGCCAATTCATTTGGGACCCTGCGGATC
M Q Y Q T R D R S S K E Q G F F R I S Q F I W D P A D

CATGCAGTACCAGACACGAGATAGATCTTCCAAAGAACAAGGCTTTTTTCGAATAAGCCAATTCATTTGGGACCCTGCGGATC
M Q Y Q T R D R S S K E Q G F F R I S Q F I W D P A D

CATGCAGTACCAGACACGAGATAGATCTTCCAAAGAACAAGGCTTTTTTCGAACAAGCCAATTCATTTGGGACCCTGCGGATC
M Q Y Q T R D R S S K E Q G F F R T S Q F I W D P A D

CATGCAGTACCAGACACGAGATAGATCTTCCAAAGAACAAGGCTTTTTTCGAACAAGCCAATTCATTTGGGACCCTGCGGATC
M Q Y Q T R D R S S K E Q G F F R T S Q F I W D P A D

6,550 6,560 6,570 6,580 6,590 6,600 6,610 6,620 6,630

CATCCTTTTCCCTATTCAAAGATCAGCCCTCTGTCTCTGTGTTTTCACGTCGAGAATTCTTTGCAGATGAAGAGATGTCAAAGGGG

```
CATCCTTTTCCCTATTCAAAGATCAGCCCTCTGTCTCTGTGTTTTCACGTCGAGAATTCTTTGCAGATGAAGAGATGTCAAAGGGG
P S F S L F K D Q P S V S V F S R R E F F A D E E M S K G
CATCCTTTTCCCTATTCAAAGATCAGCCCTCTGTCTCTGTGTTTTCACGTCGAGAATTCTTTGCAGATGAAGAGATGTCAAAGGGG
P S F S L F K D Q P S V S V F S R R E F F A D E E M S K G
CATCCTTTTCCCTATTCAAAGATCAGCCCTCTGTCTCTGTGTTTTCACGTCGAGAATTCTTTGCAGATGAAGAGATGTCAAAGGGG
P S F S L F K D Q P S V S V F S R R E F F A D E E M S K G
CATCCTTTTCCCTATTCAAAGATAAGCCCTCTGTCTCTGTGTTTTCACGTCGAGAATTCTTTGCAGATGAAGAGATGTCAAAGGGG
P S F S L F K D K P S V S V F S R R E F F A D E E M S K G
CATCCTTTTCCCTATTCAAAGATCAGCCCTCTGTCTCTGTGTTTTCACGTCGAGAATTCTTTGCAGATGAAGAGATGTCAAAGGGG
P S F S L F K D Q P S V S V F S R R E F F A D E E M S K G
CATCCTTTTCCCTATTCAAAGATCAGCCCTCTGTCTCTGTGTTTTCACGTCGAGAATTCTTTGCAGATGAAGAGATGTCAAAGGGG
P S F S L F K D Q P S V S V F S R R E F F A D E E M S K G
CATCCTTTTCCCTATTCAAAGATCAGCCCTCTGTCTCTGTGTTTTCACGTCGAGAATTCTTTGCAGATGAAGAGATGTCAAAGGGG
P S F S L F K D Q P S V S V F S R R E F F A D E E M S K G
CATCCTTTTCCCTATTCAAAGATCAGCCCTCTGTCTCTGTGTTTTCACGTCGAGAATTCTTTGCAGATGAAGAGATGTCAAAGGGG
P S F S L F K D Q P S V S V F S R R E F F A D E E M S K G
CATCCTTTTCCCTATTCAAAGATCAGCCCTCTGTCTCTGTGTTTTCACGTCGAGAATTCTTTGCAGATGAAGAGATGTCAAAGGGG
P S F S L F K D Q P S V S V F S R R E F F A D E E M S K G
CATCCTTTTCCCTATTCAAAGATCAGCCCTCTGTCTCTGTGTTTTCACGTCGAGAATTCTTTGCAGATGAAGAGATGTCAAAGGGG
P S F S L F K D Q P S V S V F S R R E F F A D E E M S K G
CATCCTTTTCCCTATTCAAAGATCAGCCCTCTGTCTCTGTGTTTTCACGTCGAGAATTCTTTGCAGATGAAGAGATGTCAAAGGGG
P S F S L F K D Q P S V S V F S R R E F F A D E E M S K G
CATCCTTTTCCCTATTCAAAGATCAGCCCTCTGTCTCTGTGTTTTCACGTCGAGAATTCTTTGCAGATGAAGAGATGTCAAAGGGG
P S F S L F K D Q P S V S V F S R R E F F A D E E M S K G
CATCCTTTTCCCTATTCAAAGATCAGCCCTCTGTCTCTGTGTTTTCACGTCGAGAATTCTTTGCAGATGAAGAGATGTCAAAGGGG
P S F S L F K D Q P S V S V F S R R E F F A D E E M S K G
CATCCTTTTCCCTATTCAAAGATCAGCCCTCTGTCTCTGTGTTTTCACGTCGAGAATTCTTTGCAGATGAAGAGATGTCAAAGGGG
P S F S L F K D Q P S V S V F S R R E F F A D E E M S K G
CATCCTTTTCCCTATTCAAAGATCAGCCCTCTGTCTCTGTGTTTTCACGTCGAGAATTCTTTGCAGATGAAGAGATGTCAAAGGGG
P S F S L F K D Q P S V S V F S R R E F F A D E E M S K G
CATCCTTTTCCCTATTCAAAGATCAGCCCTCTGTCTCTGTGTTTTCACGTCGAGAATTCTTTGCAGATGAAGAGATGTCAAAGGGG
P S F S L F K D Q P S V S V F S R R E F F A D E E M S K G
CATCCTTTTCCCTATTCAAAGATCAGCCCTCTGTCTCTGTGTTTTCACGTCGAGAATTCTTTGCAGATGAAGAGATGTCAAAGGGG
P S F S L F K D Q P S V S V F S R R E F F A D E E M S K G
CATCCTTTTCCCTATTCAAAGATCAGCCCTCTGTCTCTGTGTTTTCACGTCGAGAATTCTTTGCAGATGAAGAGATGTCAAAGGGG
P S F S L F K D Q P S V S V F S R R E F F A D E E M S K G
CATCCTTTTCCCTATTCAAAGATCAGCCCTCTGTCTCTGTGTTTTCACGTCGAGAATTCTTTGCAGATGAAGAGATGTCAAAGGGG
P S F S L F K D Q P S V S V F S R R E F F A D E E M S K G
CATCCTTTTCCCTATTCAAAGATCAGCCCTCTGTCTCTGTGTTTTCACGTCGAGAATTCTTTGCAGATGAAGAGATGTCAAAGGGG
P S F S L F K D Q P S V S V F S R R E F F A D E E M S K G
CATCCTTTTCCCTATTCAAAGATCAGCCCTCTGTCTCTGTGTTTTCACGTCGAGAATTCTTTGCAGATGAAGAGATGTCAAAGGGG
P S F S L F K D Q P S V S V F S R R E F F A D E E M S K G
CATCCTTTTCCCTATTCAAAGATCAGCCCTCTGTCTCTGTGTTTTCACGTCGAGAATTCTTTGCAGATGAAGAGATGTCAAAGGGG
P S F S L F K D Q P S V S V F S R R E F F A D E E M S K G
CATCCTTTTCCCTATTCAAAGATCAGCCCTCTGTCTCTGTGTTTTCACGTCGAGAATTCTTTGCAGATGAAGAGATGTCAAAGGGG
P S F S L F K D Q P S V S V F S R R E F F A D E E M S K G
```

6,640 6,650 6,660 6,670 6,680 6,690 6,700 6,71

CTCATTGCTTCCCAAACAAATCCTCCTACATCTATATATAAACGCTGGTTCATCAAGAATACGCAAGAAAAGCACTT

CTCATTGCTTCCCAAACAAATCCTCCTACATCTATATATAAACGCTGGTTCATCAAGAATACGCAAGAAAAGCACTT
L I A S Q T N P P T S I Y K R W F I K N T Q E K H F

CTCATTGCTTCCCAAACAAATCCTCCTACATCTATATATAAACGCTGGTTCATCAAGAATACGCAAGAAAAGCACTT
L I A S Q T N P P T S I Y K R W F I K N T Q E K H F

CTCATTGCTTCCCAAACAAATCCTCCTACATCTATATATAAACGCTGGTTCATCAAGAATACGCAAGAAAAGCACTT
L I A S Q T N P P T S I Y K R W F I K N T Q E K H F

CTCATTGCTTCCCAAACAAATCCTCCTACATCTATATATAAACGCTGGTTCATCAAGAATACGCAAGAAAAGCACTT
L I A S Q T N P P T S I Y K R W F I K N T Q E K H F

CTCATTGCTTCCCAAACAAATCCTCCTACATCTATATATAAACGCTGGTTCATCAAGAATACGCAAGAAAAGCACTT
L I A S Q T N P P T S I Y K R W F I K N T Q E K H F

CTCATTGCTTCCCAAACAAATCCTCCTACATCTATATATAAACGCTGGTTCATCAAGAATACGCAAGAAAAGCACTT
L I A S Q T N P P T S I Y K R W F I K N T Q E K H F

CTCATTGCTTCCCAAACAAATCCTCCTACATCTATATATAAACGCTGGTTCATCAAGAATACGCAAGAAAAGCACTT
L I A S Q T N P P T S I Y K R W F I K N T Q E K H F

CTCATTGCTTCCCAAACAAATCCTCCTACATCTATATATAAACGCTGGTTCATCAAGAATACGCAAGAAAAGCACTT
L I A S Q T N P P T S I Y K R W F I K N T Q E K H F

CTCATTGCTTCCCAAACAAATCCTCCTACATCTATATATAAACGCTGGTTCATCAAGAATACGCAAGAAAAGCACTT
L I A S Q T N P P T S I Y K R W F I K N T Q E K H F

CTCATTGCTTCCCAAACAAATCCTCCTACATCTATATATAAACGCTGGTTCATCAAGAATACGCAAGAAAAGCACTT
L I A S Q T N P P T S I Y K R W F I K N T Q E K H F

CTCATTGCTTCCCAAACAAATCCTCCTACATCTATATATAAACGCTGGTTCATCAAGAATACGCAAGAAAAGCACTT
L I A S Q T N P P T S I Y K R W F I K N T Q E K H F

CTCATTGCTTCCCAAACAAATCCTCCTACATCTATATATAAACGCTGGTTCATCAAGAATACGCAAGAAAAGCACTT
L I A S Q T N P P T S I Y K R W F I K N T Q E K H F

CTCATTGCTTCCCAAACAAATCCTCCTACATCTATATATAAACGCTGGTTCATCAAGAATACGCAAGAAAAGCACTT
L I A S Q T N P P T S I Y K R W F I K N T Q E K H F

CTCATTGCTTCCCAAACAAATCCTCCTACATCTATATATAAACGCTGGTTCATCAAGAATACGCAAGAAAAGCACTT
L I A S Q T N P P T S I Y K R W F I K N T Q E K H F

CTCATTGCTTCCCAAACAAATCCTCCTACATCTATATATAAACGCTGGTTCATCAAGAATACGCAAGAAAAGCACTT
L I A S Q T N P P T S I Y K R W F I K N T Q E K H F

CTCATTGCTTCCCAAACAAATCCTCCTACATCTATATATAAACGCTGGTTCATCAAGAATACGCAAGAAAAGCACTT
L I A S Q T N P P T S I Y K R W F I K N T Q E K H F

CTCATTGCTTCCCAAACAAATCCTCCTACATCTATATATAAACGCTGGTTCATCAAGAATACGCAAGAAAAGCACTT
L I A S Q T N P P T S I Y K R W F I K N T Q E K H F

CTCATTGCTTCCCAAACAAATCCTCCTACATCTATATATAAACGCTGGTTCATCAAGAATACGCAAGAAAAGCACTT
L I A S Q T N P P T S I Y K R W F I K N T Q E K H F

CTCATTGCTTCCCAAACAAATCCTCCTACATCTATATATAAACGCTGGTTCATCAAGAATACGCAAGAAAAGCACTT
L I A S Q T N P P T S I Y K R W F I K N T Q E K H F

CTCATTGCTTCCCAAACAAATCCTCCTACATCTATATATAAACGCTGGTTCATCAAGAATACGCAAGAAAAGCACTT
L I A S Q T N P P T S I Y K R W F I K N T Q E K H F

CTCATTGCTTCCCAAACAAATCCTCCTACATCTATATATAAACGCTGGTTCATCAAGAATACGCAAGAAAAGCACTT
L I A S Q T N P P T S I Y K R W F I K N T Q E K H F

CTCATTGCTTCCCAAACAAATCCTCCTACATCTATATATAAACGCTGGTTCATCAAGAATACGCAAGAAAAGCACTT
L I A S Q T N P P T S I Y K R W F I K N T Q E K H F

CTCATTGCTTCCCAAACAAATCCTCCTACATCTATATATAAACGCTGGTTCATCAAGAATACGCAAGAAAAGCACTT
L I A S Q T N P P T S I Y K R W F I K N T Q E K H F

0 6,720 6,730 6,740 6,750 6,760 6,770 6,780

CGAATTGTTGATTCATCGCCAGAGATGGTTTAGAACCAATAGTTCATTATCTAATGGATCTTTCCGTTCTAATA

CGAATTGTTGATTCATCGCCAGAGATGGTTTAGAACCAATAGTTCATTATCTAATGGATCTTTCCGTTCTAATA
E L L I H R Q R W F R T N S S L S N G S F R S N

CGAATTGTTGATTCATCGCCAGAGATGGTTTAGAACCAATAGTTCATTATCTAATGGATCTTTCCGTTCTAATA
E L L I H R Q R W F R T N S S L S N G S F R S N

CGAATTGTTGATTCATCGCCAGAGATGGTTTAGAACCAATAGTTCATTATCTAATGGATCTTTCCGTTCTAATA
E L L I H R Q R W F R T N S S L S N G S F R S N

CGAATTGTTGATTCATCGCCAGAGATGGTTTAGAACCAATAGTTCATTATCTAATGGATCTTTCCGTTCTAATA
E L L I H R Q R W F R T N S S L S N G S F R S N

CGAATTGTTGATTCATCGCCAGAGATGGTTTAGAACCAATAGTTCATTATCTAATGGATCTTTCCGTTCTAATA
E L L I H R Q R W F R T N S S L S N G S F R S N

CGAATTGTTGATTCATCGCCAGAGATGGTTTAGAACCAATAGTTCATTATCTAATGGATCTTTCCGTTCTAATA
E L L I H R Q R W F R T N S S L S N G S F R S N

CGAATTGTTGATTCATCGCCAGAGATGGTTTAGAACCAATAGTTCATTATCTAATGGATCTTTCCGTTCTAATA
E L L I H R Q R W F R T N S S L S N G S F R S N

CGAATTGTTGATTCATCGCCAGAGATGGTTTAGAACCAATAGTTCATTATCTAATGGATCTTTCCGTTCTAATA
E L L I H R Q R W F R T N S S L S N G S F R S N

CGAATTGTTGATTCATCGCCAGAGATGGTTTAGAACCAATAGTTCATTATCTAATGGATCTTTCCGTTCTAATA
E L L I H R Q R W F R T N S S L S N G S F R S N

CGAATTGTTGATTCATCGCCAGAGATGGTTTAGAACCAATAGTTCATTATCTAATGGATCTTTCCGTTCTAATA
E L L I H R Q R W F R T N S S L S N G S F R S N

CGAATTGTTGATTCATCGCCAGAGATGGTTTAGAACCAATAGTTCATTATCTAATGGATCTTTCCGTTCTAATA
E L L I H R Q R W F R T N S S L S N G S F R S N

CGAATTGTTGATTCATCGCCAGAGATGGTTTAGAACCAATAGTTCATTATCTAATGGATCTTTCCGTTCTAATA
E L L I H R Q R W F R T N S S L S N G S F R S N

CGAATTGTTGATTCATCGCCAGAGATGGTTTAGAACCAATAGTTCATTATCTAATGGATCTTTCCGTTCTAATA
E L L I H R Q R W F R T N S S L S N G S F R S N

CGAATTGTTGATTCATCGCCAGAGATGGTTTAGAACCAATAGTTCATTATCTAATGGATCTTTCCGTTCTAATA
E L L I H R Q R W F R T N S S L S N G S F R S N

CGAATTGTTGATTCATCGCCAGAGATGGTTTAGAACCAATAGTTCATTATCTAATGGATCTTTCCGTTCTAATA
E L L I H R Q R W F R T N S S L S N G S F R S N

CGAATTGTTGATTCATCGCCAGAGATGGTTTAGAACCAATAGTTCATTATCTAATGGATCTTTCCGTTCTAATA
E L L I H R Q R W F R T N S S L S N G S F R S N

CGAATTGTTGATTCATCGCCAGAGATGGTTTAGAACCAATAGTTCATTATCTAATGGATCTTTCCGTTCTAATA
E L L I H R Q R W F R T N S S L S N G S F R S N

CGAATTGTTGATTCATCGCCAGAGATGGTTTAGAACCAATAGTTCATTATCTAATGGATCTTTCCGTTCTAATA
E L L I H R Q R W F R T N S S L S N G S F R S N

CGAATTGTTGATTCATCGCCAGAGATGGTTTAGAACCAATAGTTCATTATCTAATGGATCTTTCCGTTCTAATA
E L L I H R Q R W F R T N S S L S N G S F R S N

CGAATTGTTGATTCATCGCCAGAGATGGTTTAGAACCAATAGTTCATTATCTAATGGATCTTTCCGTTCTAATA
E L L I H R Q R W F R T N S S L S N G S F R S N

CGAATTGTTGATTCATCGCCAGAGATGGTTTAGAACCAATAGTTCATTATCTAATGGATCTTTCCGTTCTAATA
E L L I H R Q R W F R T N S S L S N G S F R S N

CGAATTGTTGATTCATCGCCAGAGATGGTTTAGAACCAATAGTTCATTATCTAATGGATCTTTCCGTTCTAATA
E L L I H R Q R W F R T N S S L S N G S F R S N

CGAATTGTTGATTCATCGCCAGAGATGGTTTAGAACCAATAGTTCATTATCTAATGGATCTTTCCGTTCTAATA
E L L I H R Q R W F R T N S S L S N G S F R S N

6,790 6,800 6,810 6,820 6,830 6,840 6,850 6

CTCTATCCGAGAGTTATCAGTATTTATCAAATCTGTTCCTATCTAACGGAACGCTATTGGATCAAATGACAAAGA

CTCTATCCGAGAGTTATCAGTATTTATCAAATCTGTTCCTATCTAACGGAACGCTATTGGATCAAATGACAAAGA
T L S E S Y Q Y L S N L F L S N G T L L D Q M T K

CTCTATCCGAGAGTTATCAGTATTTATCAAATCTGTTCCTATCTAACGGAACGCTATTGGATCAAATGACAAAGA
T L S E S Y Q Y L S N L F L S N G T L L D Q M T K

CTCTATCCGAGAGTTATCAGTATTTATCAAATCTGTTCCTATCTAACGGAACGCTATTGGATCAAATGACAAAGA
T L S E S Y Q Y L S N L F L S N G T L L D Q M T K

CTCTATCCGAGAGTTATCAGTATTTATCAAATCTGTTCCTATCTAACGGAACGCTATTGGATCAAATGACAAAGA
T L S E S Y Q Y L S N L F L S N G T L L D Q M T K

CTCTATCCGAGAGTTATCAGTATTTATCAAATCTGTTCCTATCTAACGGAACGCTATTGGATCAAATGACAAAGA
T L S E S Y Q Y L S N L F L S N G T L L D Q M T K

CTCTATCCGAGAGTTATCAGTATTTATCAAATCTGTTCCTATCTAACGGAACGCTATTGGATCAAATGACAAAGA
T L S E S Y Q Y L S N L F L S N G T L L D Q M T K

CTCTATCCGAGAGTTATCAGTATTTATCAAATCTGTTCCTATCTAACGGAACGCTATTGGATCAAATGACAAAGA
T L S E S Y Q Y L S N L F L S N G T L L D Q M T K

CTCTATCCGAGAGTTATCAGTATTTATCAAATCTGTTCCTATCTAACGGAACGCTATTGGATCAAATGACAAAGA
T L S E S Y Q Y L S N L F L S N G T L L D Q M T K

CTCTATCCGAGAGTTATCAGTATTTATCAAATCTGTTCCTATCTAACGGAACGCTATTGGATCAAATGACAAAGA
T L S E S Y Q Y L S N L F L S N G T L L D Q M T K

CTCTATCCGAGAGTTATCAGTATTTATCAAATCTGTTCCTATCTAACGGAACGCTATTGGATCAAATGACAAAGA
T L S E S Y Q Y L S N L F L S N G T L L D Q M T K

CTCTATCCGAGAGTTATCAGTATTTATCAAATCTGTTCCTATCTAACGGAACGCTATTGGATCAAATGACAAAGA
T L S E S Y Q Y L S N L F L S N G T L L D Q M T K

CTCTATCCGAGAGTTATCAGTATTTATCAAATCTGTTCCTATCTAACGGAACGCTATTGGATCAAATGACAAAGA
T L S E S Y Q Y L S N L F L S N G T L L D Q M T K

CTCTATCCGAGAGTTATCAGTATTTATCAAATCTGTTCCTATCTAACGGAACGCTATTGGATCAAATGACAAAGA
T L S E S Y Q Y L S N L F L S N G T L L D Q M T K

CTCTATCCGAGAGTTATCAGTATTTATCAAATCTGTTCCTATCTAACGGAACGCTATTGGATCAAATGACAAAGA
T L S E S Y Q Y L S N L F L S N G T L L D Q M T K

CTCTATCCGAGAGTTATCAGTATTTATCAAATCTGTTCCTATCTAACGGAACGCTATTGGATCAAATGACAAAGA
T L S E S Y Q Y L S N L F L S N G T L L D Q M T K

CTCTATCCGAGAGTTATCAGTATTTATCAAATCTGTTCCTATCTAACGGAACGCTATTGGATCAAATGACAAAGA
T L S E S Y Q Y L S N L F L S N G T L L D Q M T K

CTCTATCCGAGAGTTATCAGTATTTATCAAATCTGTTCCTATCTAACGGAACGCTATTGGATCAAATGACAAAGA
T L S E S Y Q Y L S N L F L S N G T L L D Q M T K

CTCTATCCGAGAGTTATCAGTATTTATCAAATCTGTTCCTATCTAACGGAACGCTATTGGATCAAATGACAAAGA
T L S E S Y Q Y L S N L F L S N G T L L D Q M T K

CTCTATCCGAGAGTTATCAGTATTTATCAAATCTGTTCCTATCTAACGGAACGCTATTGGATCAAATGACAAAGA
T L S E S Y Q Y L S N L F L S N G T L L D Q M T K

CTCTATCCGAGAGTTATCAGTATTTATCAAATCTGTTCCTATCTAACGGAACGCTATTGGATCAAATGACAAAGA
T L S E S Y Q Y L S N L F L S N G T L L D Q M T K

CTCTATCCGAGAGTTATCAGTATTTATCAAATCTGTTCCTATCTAACGGAACGCTATTGGATCAAATGACAAAGA
T L S E S Y Q Y L S N L F L S N G T L L D Q M T K

CTCTATCCGAGAGTTATCAGTATTTATCAAATCTGTTCCTATCTAACGGAACGCTATTGGATCAAATGACAAAGA
T L S E S Y Q Y L S N L F L S N G T L L D Q M T K

CTCTATCCGAGAGTTATCAGTATTTATCAAATCTGTTCCTATCTAACGGAACGCTATTGGATCAAATGACAAAGA
T L S E S Y Q Y L S N L F L S N G T L L D Q M T K

```
6,860     6,870     6,880     6,890     6,900     6,910     6,920     6,930     6,942
CATTGTTGAGAAAGAGATGGCTTTTCCCGGATGAAATGAAACATTTGATTCATGTAACAGGAGAAAGATTCCCCATTCCTTAG

CATTGTTGAGAAAGAGATGGCTTTTCCCGGATGAAATGAAACATTTGATTCATGTAACAGGAGAAAGATTCCCCATTCCTTAG
T L L R K R W L F P D E M K H L I H V T G E R F P I P *
CATTGTTGAGAAAGAGATGGCTTTTCCCGGATGAAATGAAACATTTGATTCATGTAACAGGAGAAAGATTCCCCATTCCTTAG
T L L R K R W L F P D E M K H L I H V T G E R F P I P *
CATTGTTGAGAAAGAGATGGCTTTTCCCGGATGAAATGAAACATTTGATTCATGTAACAGGAGAAAGATTCCCCATTCCTTAG
T L L R K R W L F P D E M K H L I H V T G E R F P I P *
CATTGTTGAGAAAGAGATGGCTTTTCCCGGATGAAATGAAACATTTGATTCATGTAACAGGAGAAAGATTCCCCATTCCTTAG
T L L R K R W L F P D E M K H L I H V T G E R F P I P *
CATTGTTGAGAAAGAGATGGCTTTTCCCGGATGAAATGAAACATTTGATTCATGTAACAGGAGAAAGATTCCCCATTCCTTAG
T L L R K R W L F P D E M K H L I H V T G E R F P I P *
CATTGTTGAGAAAGAGATGGCTTTTCCCGGATGAAATGAAACATTTGATTCATGTAACAGGAGAAAGATTCCCCATTCCTTAG
T L L R K R W L F P D E M K H L I H V T G E R F P I P *
CATTGTTGAGAAAGAGATGGCTTTTCCCGGATGAAATGAAACATTTGATTCATGTAACAGGAGAAAGATTCCCCATTCCTTAG
T L L R K R W L F P D E M K H L I H V T G E R F P I P *
CATTGTTGAGAAAGAGATGGCTTTTCCCGGATGAAATGAAACATTTGATTCATGTAACAGGAGAAAGATTCCCCATTCCTTAG
T L L R K R W L F P D E M K H L I H V T G E R F P I P *
CATTGTTGAGAAAGAGATGGCTTTTCCCGGATGAAATGAAACATTTGATTCATGTAACAGGAGAAAGATTCCCCATTCCTTAG
T L L R K R W L F P D E M K H L I H V T G E R F P I P *
CATTGTTGAGAAAGAGATGGCTTTTCCCGGATGAAATGAAACATTTGATTCATGTAACAGGAGAAAGATTCCCCATTCCTTAG
T L L R K R W L F P D E M K H L I H V T G E R F P I P *
CATTGTTGAGAAAGAGATGGCTTTTCCCGGATGAAATGAAACATTTGATTCATGTAACAGGAGAAAGATTCCCCATTCCTTAG
T L L R K R W L F P D E M K H L I H V T G E R F P I P *
CATTGTTGAGAAAGAGATGGCTTTTCCCGGATGAAATGAAACATTTGATTCATGTAACAGGAGAAAGATTCCCCATTCCTTAG
T L L R K R W L F P D E M K H L I H V T G E R F P I P *
CATTGTTGAGAAAGAGATGGCTTTTCCCGGATGAAATGAAACATTTGATTCATGTAACAGGAGAAAGATTCCCCATTCCTTAG
T L L R K R W L F P D E M K H L I H V T G E R F P I P *
CATTGTTGAGAAAGAGATGGCTTTTCCCGGATGAAATGAAACATTTGATTCATGTAACAGGAGAAAGATTCCCCATTCCTTAG
T L L R K R W L F P D E M K H L I H V T G E R F P I P *
CATTGTTGAGAAAGAGATGGCTTTTCCCGGATGAAATGAAACATTTGATTCATGTAACAGGAGAAAGATTCCCCATTCCTTAG
T L L R K R W L F P D E M K H L I H V T G E R F P I P *
CATTGTTGAGAAAGAGATGGCTTTTCCCGGATGAAATGAAACATTTGATTCATGTAACAGGAGAAAGATTCCCCATTCCTTAG
T L L R K R W L F P D E M K H L I H V T G E R F P I P *
CATTGTTGAGAAAGAGATGGCTTTTCCCGGATGAAATGAAACATTTGATTCATGTAACAGGAGAAAGATTCCCCATTCCTTAG
T L L R K R W L F P D E M K H L I H V T G E R F P I P *
CATTGTTGAGAAAGAGATGGCTTTTCCCGGATGAAATGAAACATTTGATTCATGTAACAGGAGAAAGATTCCCCATTCCTTAG
T L L R K R W L F P D E M K H L I H V T G E R F P I P *
CATTGTTGAGAAAGAGATGGCTTTTCCCGGATGAAATGAAACATTTGATTCATGTAACAGGAGAAAGATTCTCCATTCCTTAG
T L L R K R W L F P D E M K H L I H V T G E R F S I P *
CATTGTTGAGAAAGAGATGGCTTTTCCCGGATGAAATGAAACATTTGATTCATGTAACAGGAGAAAGATTCTCCATTCCTTAG
T L L R K R W L F P D E M K H L I H V T G E R F S I P *
CATTGTTGAGAAAGAGATGGCTTTTCCCGGATGAAATGAAACATTTGATTCATGTAACAGGAGAAAGATTCTCCATTCCTTAG
T L L R K R W L F P D E M K H L I H V T G E R F S I P *
CATTGTTGAGAAAGAGATGGCTTTTCCCGGATGAAATGAAACATTTGATTCATGTAACAGGAGAAAGATTCTCCATTCCTTAG
T L L R K R W L F P D E M K H L I H V T G E R F S I P *
CATTGTTGAGAAAGAGATGGCTTTTCCCGGATGAAATGAAACATTTGATTCATGTAACAGGAGAAAGATTCCCCATTCCTTAG
T L L R K R W L F P D E M K H L I H V T G E R F P I P *
```
