# Supplementary material for: Comparative plastid genomics of Gastrochilus (Orchidaceae) with insights into molecular markers and phylogeny
Source: BMC Genomics. 2026 Jun 26;27:777. doi: 10.1186/s12864-026-12698-8 (PMC13599090; doi:10.1186/s12864-026-12698-8)
Supplement: Supplementary file 1 [file 12864_2026_12698_MOESM2_ESM.zip › Supplementary material/Fig. S5.pdf]

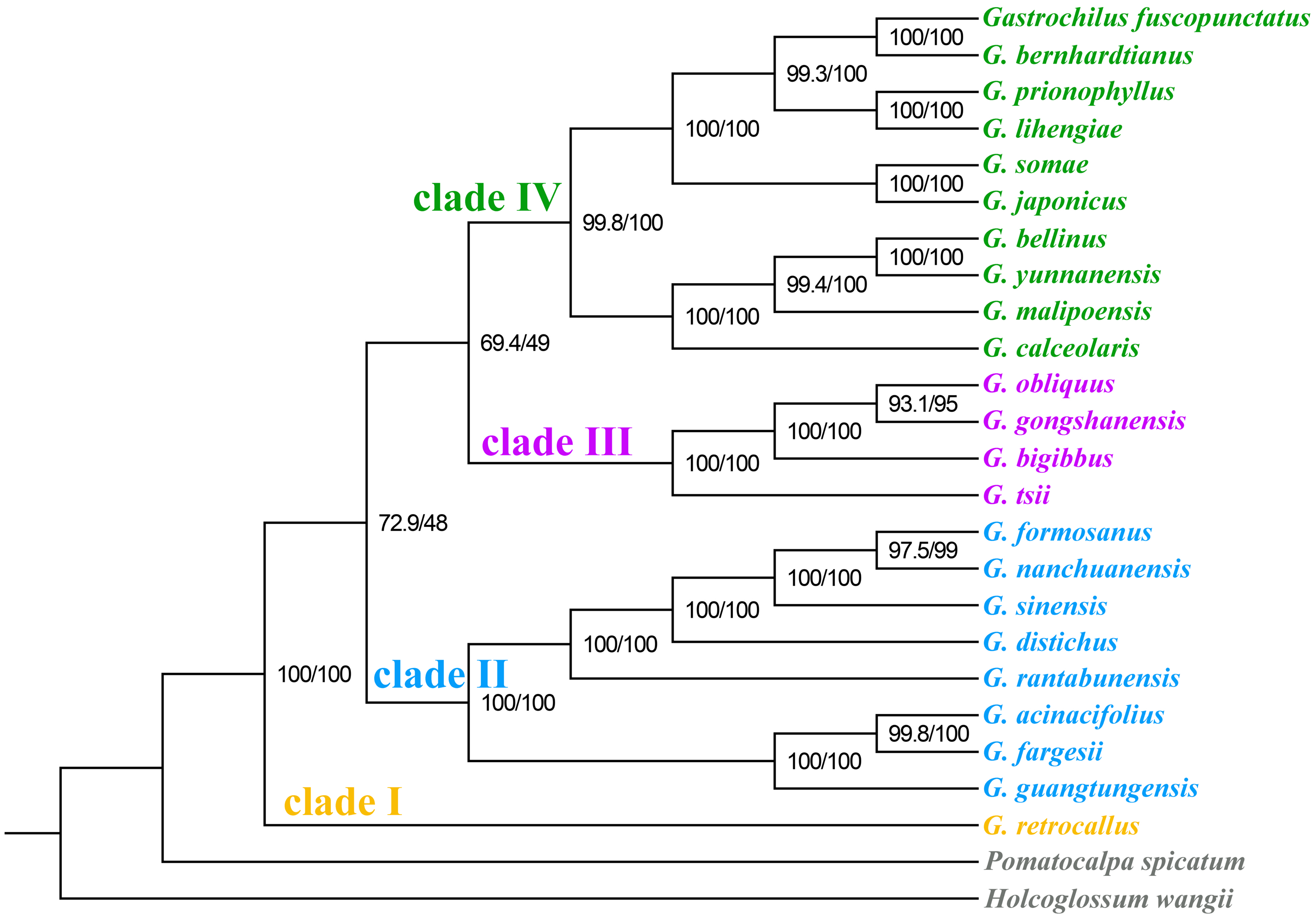
Gastrochilus fuscopunctatus
G. bernhardtianus
G. prionophyllus
G. lihengiae
G. somae
G. japonicus
G. bellinus
G. yunnanensis
G. malipoensis
G. calceolaris
G. obliquus
G. gongshanensis
G. bigibbus
G. tsii
G. formosanus
G. nanchuanensis
G. sinensis
G. distichus
G. rantabunensis
G. acinacifolius
G. fargesii
G. guangtungensis
G. retrocallus
Pomatocalpa spicatum
Holcoglossum wangii
clade IV
clade III
clade II
clade I
100/100
99.3/100
100/100
100/100
100/100
99.8/100
100/100
99.4/100
100/100
69.4/49
93.1/95
100/100
100/100
72.9/48
97.5/99
100/100
100/100
100/100
100/100
100/100
99.8/100
100/100
