# Supplementary material for: Comparative plastid genomics of Gastrochilus (Orchidaceae) with insights into molecular markers and phylogeny
Source: BMC Genomics. 2026 Jun 26;27:777. doi: 10.1186/s12864-026-12698-8 (PMC13599090; doi:10.1186/s12864-026-12698-8)
Supplement: Supplementary file 1 [file 12864_2026_12698_MOESM2_ESM.zip › Supplementary material/Supplementary_Figure_Legends.docx]

Fig. S1 Plastid genome maps of four *Gastrochilus* species. Genes are color-coded according to their functional categories and are plotted on the inner (transcribed counterclockwise) or outer (transcribed clockwise) circle. The inner gray ring visualizes the GC content across the genome. The regions between the circles depict the large single-copy (LSC), small single-copy (SSC), and the two inverted repeat (IRa and IRb) regions. For each species, the total genome size and overall GC content are annotated in the center of the map

Fig. S2 Mauve alignment of 23 *Gastrochilus* plastid genomes. Colored rectangles represent locally collinear blocks (LCBs), each indicating a set of homologous sequences conserved across genomes. Lines connecting LCBs show homologous relationships between species. Gene annotations appear in two rows below each LCB: the upper row for genes on the forward strand, the lower for the reverse strand. White rectangles denote protein-coding sequences (with introns shown as thin white lines); green and red rectangles represent tRNA and rRNA genes, respectively. The top x‑axis shows genome coordinates in base pairs (bp). The phylogenetic tree on the left is the maximum-likelihood (ML) tree inferred from complete plastid genomes

Fig. S3 Visualization of positively selected sites in the *rps16* coding sequences across 23 *Gastrochilus* species. For each species, the nucleotide sequence and its translated amino acid sequence are shown, as visualized using Geneious Prime v2021.1.1. The species order follows the phylogenetic relationships inferred from complete plastid genomes, allowing visual comparison of sequence divergence and selection patterns among species

Fig. S4 Visualization of positively selected sites in *ycf2* gene across 23 *Gastrochilus* species. For each species, the nucleotide sequence and its translated amino acid sequence are shown, as visualized using Geneious Prime v2021.1.1. The species order follows the phylogenetic relationships inferred from complete plastid genomes, allowing visual comparison of sequence divergence and selection patterns among species

Fig. S5 Maximum-likelihood phylogenetic tree of *Gastrochilus* based on 68 shared coding DNA sequences. Nodal support values are shown as UFBoot support / SH-aLRT test values
